# The Search for Herbal Antibiotics: An In-Silico Investigation of Antibacterial Phytochemicals

**DOI:** 10.3390/antibiotics5030030

**Published:** 2016-09-12

**Authors:** Mary Snow Setzer, Javad Sharifi-Rad, William N. Setzer

**Affiliations:** 1 Department of Chemistry, University of Alabama in Huntsville, Huntsville, AL 35899, USA; mary.setzer@uah.edu; 2 Zabol Medicinal Plants Research Center, Zabol University of Medical Sciences, Zabol 61615-585, Iran; javad.sharifirad@gmail.com; 3 Department of Pharmacognosy, Zabol University of Medical Sciences, Zabol 61615-585, Iran

**Keywords:** antibiotic resistance, antibacterial phytochemicals, molecular docking, bacterial protein targets

## Abstract

Recently, the emergence and spread of pathogenic bacterial resistance to many antibiotics (multidrug-resistant strains) have been increasing throughout the world. This phenomenon is of great concern and there is a need to find alternative chemotherapeutic agents to combat these antibiotic-resistant microorganisms. Higher plants may serve as a resource for new antimicrobials to replace or augment current therapeutic options. In this work, we have carried out a molecular docking study of a total of 561 antibacterial phytochemicals listed in the *Dictionary of Natural Products*, including 77 alkaloids (17 indole alkaloids, 27 isoquinoline alkaloids, 4 steroidal alkaloids, and 28 miscellaneous alkaloids), 99 terpenoids (5 monoterpenoids, 31 sesquiterpenoids, 52 diterpenoids, and 11 triterpenoids), 309 polyphenolics (87 flavonoids, 25 chalcones, 41 isoflavonoids, 5 neoflavonoids, 12 pterocarpans, 10 chromones, 7 condensed tannins, 11 coumarins, 30 stilbenoids, 2 lignans, 5 phenylpropanoids, 13 xanthones, 5 hydrolyzable tannins, and 56 miscellaneous phenolics), 30 quinones, and 46 miscellaneous phytochemicals, with six bacterial protein targets (peptide deformylase, DNA gyrase/topoisomerase IV, UDP-galactose mutase, protein tyrosine phosphatase, cytochrome P450 CYP121, and NAD^+^-dependent DNA ligase). In addition, 35 known inhibitors were docked with their respective targets for comparison purposes. Prenylated polyphenolics showed the best docking profiles, while terpenoids had the poorest. The most susceptible protein targets were peptide deformylases and NAD^+^-dependent DNA ligases.

## 1. Introduction

Recently, established antibiotics have become less effective against numerous infectious organisms, and the Centers for Disease Control and Prevention (CDC) has warned of a “post-antibiotic era” [1]. This concern is heightened by our tenuous ability to detect, contain, and prevent emerging infectious diseases. The emergence of pathogenic microbes with increased resistance to existing antibiotics provides a major incentive for the discovery of new antimicrobial agents. The problems of drug-resistant pathogens have been reviewed recently [2,3,4,5]; there is a pressing need for more effective antibacterial therapy. Based on several recent reports, pathogens of immediate concern are methicillin-resistant *Staphylococcus aureus* (MRSA), a common cause of hospital-acquired infections, and which is evolving a resistance to vancomycin [6]; *Pseudomonas aeruginosa* in which multidrug resistance has become problematic [7]; *Streptococcus pneumoniae*, a common respiratory pathogen in which multidrug resistance is spreading [8]; multidrug-resistant strains of *Mycobacterium tuberculosis* [9], which are causing an alarming increase in the incidence of tuberculosis; and virulent strains of *Escherichia coli*, which continue to emerge [10,11,12].

Virtual screening using cheminformatics, pharmacophore, or ligand- and structure-based target prediction methods [13] has emerged as an advantageous alternative to high-throughput screening for identification of potential lead structures or biological targets for anti-infective drug discovery. For example, Bernal and Coy-Barrera have used a combination of molecular docking and multivariate analysis to identify antifungal and antiviral xanthone lead compounds [14]. Rahimi and co-workers have used a structural similarity search along with molecular docking to identify potential *Shigella flexneri* DNA gyrase inhibitors [15]. Molecular docking has been used to identify bacterial peptidyl-tRNA hydrolase as an additional alternative target for known antibiotic drugs [16].

Until the beginning of the twentieth century, virtually all medicines were derived from natural sources, most often from plants, and plants continue to serve as sources of new medicines and provide lead compounds for drug development. These antimicrobial agents derived from higher plants have been reviewed recently [17,18]. In the discovery of new and complementary antibacterial agents, phytochemicals that show antibacterial activity can be examined for potential inhibition of bacterial target proteins such as peptide deformylase (PDF), topoisomerase IV (TopoIV), DNA gyrase B (GyrB), protein tyrosine phosphatase (Ptp), UDP-galactopyranose mutase (UGM), cytochrome P450 (CYP121), and NAD^+^-dependent DNA ligase, as well as phytochemical inhibitors of bacterial efflux pumps or quorum sensing proteins, or agents that enhance the immune system. In this work, we have carried out an in-silico screening of phytochemicals identified in the *Dictionary of Natural Products* [19] as showing antibacterial activity against several potential bacterial protein targets.

### 1.1. Peptide Deformylase

The process of bacterial protein synthesis is initiated with *N*-formylmethionine (f-Met-tRNAi), which is generated through the enzymatic transformylation of methionyl-tRNA (Met-tRNAi) by formyl methionyl transferase (f-Mett). The *N*-formyl group of the polypeptide that emerges from the ribosome after completion of the elongation process is removed by the sequential action of peptide deformylase (PDF) [20,21]. Methionine amino peptidase (MAP) then removes the *N*-terminal methionine depending on the nature of the second amino acid in the peptide chain [22]. Therefore, deformylation plays a pivotal role in bacterial protein maturation, growth, and survival; PDF is vital in a variety of pathogenic bacteria but it is not required for cytoplasmic protein synthesis in the eukaryotes. Hence, PDF has been identified as a potential antibacterial drug target [23]. Bacterial PDFs are metallohydrolases that use Fe^2+^ as the catalytic metal ion (which can be replaced with Ni^2+^ or Zn^2+^) that is tetrahedrally coordinated to two histidine residues, a cysteine residue, and a water molecule [24].

### 1.2. DNA Gyrase/Topoisomerase IV

Topoisomerase enzymes control the topological state of DNA within cells and are important for the essential process of protein translation and cell replication. Much attention in antibacterial drug discovery has been focused on the DNA gyrase (a type II topoisomerase) and topoisomerase IV. These types of topoisomerases are present in bacteria and plants, but not animals. DNA gyrase and topoisomerase IV share high structural and sequence similarity, yet play different necessary roles in the replication of DNA. Because of their vital nature and mechanisms of action, topoisomerases have become key drug targets for antibacterial drug discovery [25,26].

### 1.3. UDP-Galactopyranose Mutase

UDP-Galactopyranose mutase (UGM) is the only enzyme known to catalyze the isomerization of UDP-galactopyranose to UDP-galactofuranose. The enzyme has been identified in prokaryotes, including Gram-negative bacteria and mycobacteria, as well as eukaryotic parasites (*Leishmania major*, *Trypanosoma cruzi*), nematodes (*Caenorhabditis elegans*), and fungi (*Aspergillus fumigatus*, *Cryptococcus neoformans*), but have not been found in higher eukaryotes [27]. Galactofuranose residues are essential components of mycobacterial cell walls, and thus, UGM has been identified as a potential target for antimycobacterial chemotherapeutics.

### 1.4. Protein Tyrosine Phosphatase

Protein tyrosine phosphatases (Ptps) have been suggested to be major virulence determinants. These enzymes reverse the effect of tyrosine kinases by dephosphorylating the tyrosine residues of host cellular substrate proteins important in host cellular signaling, which attenuates host immune defenses [28,29]. Ptps are essential components for the interaction of mycobacteria with host cells, making them attractive protein targets; structural differences between mycobacterial Ptps and eukaryotic Ptps could allow for the discovery of selective mycobacterial Ptp inhibitors [28,29].

### 1.5. Cytochrome P450 CYP121

Several antifungal azole and triazole agents have been shown to inhibit mycobacterial cytochrome P450 CYP121 [30]. CYP121 is essential for *Mycobacterium tuberculosis* and there is a correlation between antimycobacterial activity and MtCYP121 binding, suggesting that MtCYP121 is the major target in *M. tuberculosis* [31]. There is low sequence similarity between MtCYP121 and mammalian P450s, which suggests that MtCYP121 is a promising antimycobacterial drug target [32].

### 1.6. NAD^+^-Dependent DNA Ligase

DNA ligases are involved in DNA replication, recombination, and repair pathways by joining adjacent 3′-hydroxyl and 5′-phosphoryl termini in DNA [33]. Bacterial DNA ligases use NAD^+^ as a cofactor, which differentiates them from eukaryotic DNA ligases, which use ATP [34]. The differences between bacterial NAD^+^-dependent DNA ligases (LigA) and mammalian ATP-dependent DNA ligases suggest that bacterial LigA should be excellent targets for antibacterial drug discovery [35].

## 2. Computational Methods

Protein-ligand docking studies were carried out based on the structures of verified bacterial protein drug targets.

Bacterial peptide deformylase: *Bacillus cereus* (BcPDF, PDB 2OKL [36]); *Escherichia coli* (EcPDF, PDB 1G2A and 1G27 [37], PDB 1LRU [38], PDB 2AI8 [39], PDB 2KMN [40], and PDB 3K6L [41]); *Mycobacterium tuberculosis* (MtPDF, PDB 3E3U [42]); *Pseudomonas aeruginosa* (PaPDF, PDB 1LRY [38], 1IX1 [43], and 1S17 [44]); and *Staphylococcus aureus* (SaPDF, PDB 1Q1Y [43], PDB 3U7K, 3U7M, and 3U7N [45]). In order to test for the selectivity toward bacterial PDF over human PDF, molecular docking of the phytochemical ligands was also carried out on human PDF (HsPDF, PDB 4JE7 and 4JE8 [46]).

Bacterial DNA gyrase B/topoisomerase IV: *E. coli* (EcGyrB, PDB 1AJ6 [47], EcTopoIVB, PDB 1S16 [48]), and *M. tuberculosis* (MtGyrB, PDB 3ZKB and 3ZKD [49]).

Bacterial protein tyrosine phosphatase: *M. tuberculosis* (MtPtpA, PDB 1U2Q [28], and MtPtpB, PDB 2OZ5 [29]). In order to compare *Mycobacterium* Ptp docking over human Ptp, molecular docking of the phytochemical ligands was also carried out with human PtpB (HsPtpB, PDB 2I4H and 2I5X [50]).

Mycobacterial UDP-galactopyranose mutase: *M. tuberculosis* (MtUGM, PDB 4RPG, 4RPH, 4RPJ, 4RPK, and 4RPL [51]).

Mycobacterial cytochrome P450 CYP121: *M. tuberculosis* (MtCYP121, PDB 4IPS [52], 4KTF [53], and 5IBE [32]).

Bacterial NAD^+^-Dependent DNA ligase: *E. coli* (EcLigA, PDB 2OWO [35] and 4GLX [54]), *M. tuberculosis* (MtLigA and PDB 1ZAU [55]), *S. aureus* (SaLigA, PDB 4CC5 and 4CC6 [56]), and *S. pneumoniae* (SpLigA and PDB 4GLW [54]).

Prior to docking, all solvent molecules and the co-crystallized ligands were removed from the structures. If co-factors were present, they were retained in each protein model (e.g., divalent metal ions in peptide deformylases, flavin adenine dinucleotide (FAD) in *M. tuberculosis* UDP-galactopyranose mutase, and heme in MtCYP121). Molecular docking calculations for all compounds with each of the proteins were undertaken using Molegro Virtual Docker (version 6.0, Molegro ApS, Aarhus, Denmark) [57], with a sphere (15 Å radius) large enough to accommodate the cavity centered on the binding sites of each protein structure in order to allow each ligand to search. If a co-crystallized inhibitor or substrate was present in the structure, then that site was chosen as the binding site. If no co-crystallized ligand was present, then suitably sized (>50 Å^3^) cavities were used as potential binding sites. Standard protonation states of the proteins based on neutral pH were used in the docking studies. Each protein was used as a rigid model structure; no relaxation of the protein was performed. Assignments of the charges on each protein were based on standard templates as part of the Molegro Virtual Docker program; no other charges were necessary to be set. Overall, 561 antibacterial phytochemicals have been docked. This molecule set was comprised of 77 alkaloids, 99 terpenoids, 190 flavonoids, 119 polyphenolic compounds, 30 quinones, and 46 miscellaneous phytochemicals. Each ligand structure was built using Spartan‘14 for Windows (version 1.1.0, Wavefunction Inc., Irvine, CA, USA). For each ligand, a conformational search and geometry optimization was carried out using the MMFF force field [58]. Flexible ligand models were used in the docking and subsequent optimization scheme. Variable orientations of each of the ligands were searched and ranked based on their re-rank score. For each docking simulation the maximum number of iterations for the docking algorithm was set to 1500, with a maximum population size of 50, and 100 runs per ligand. The RMSD threshold for multiple poses was set to 1.00 Å. The generated poses from each ligand were sorted by the calculated re-rank score.

## 3. Results and Discussion

**Figure 1:**
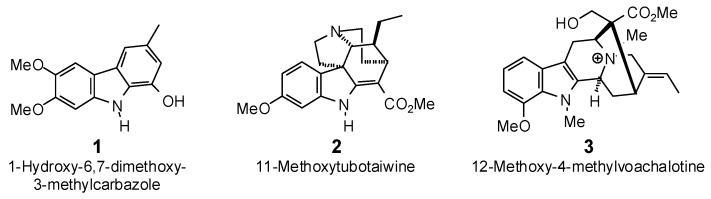

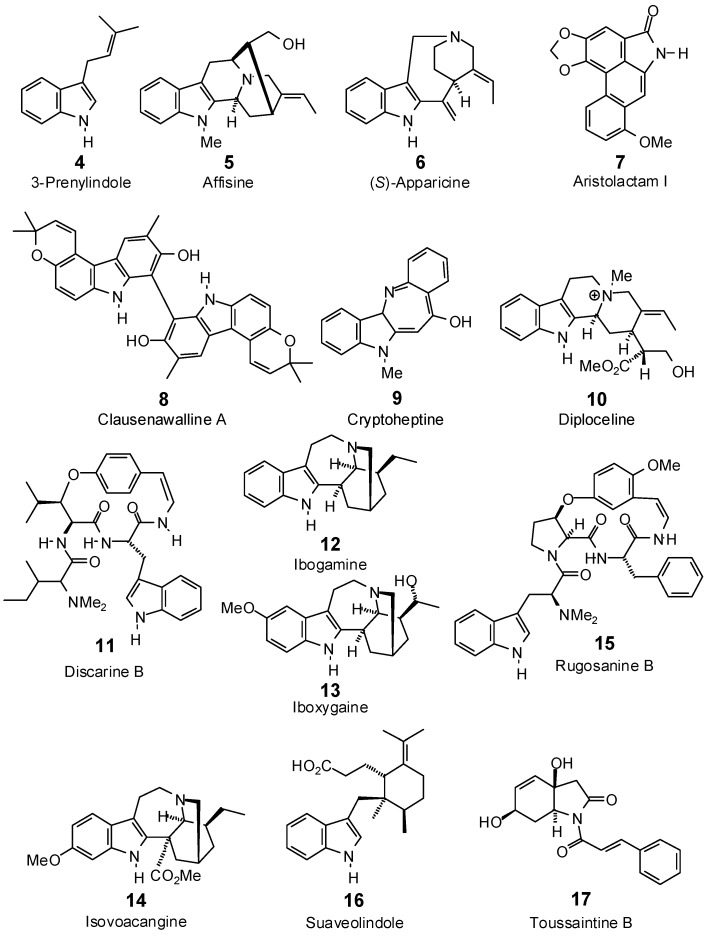
Indole alkaloids examined in this work.

**Figure 2:**
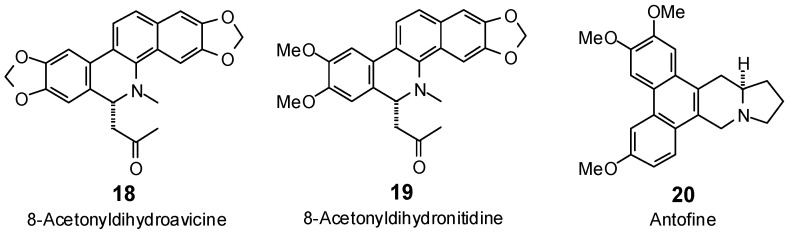

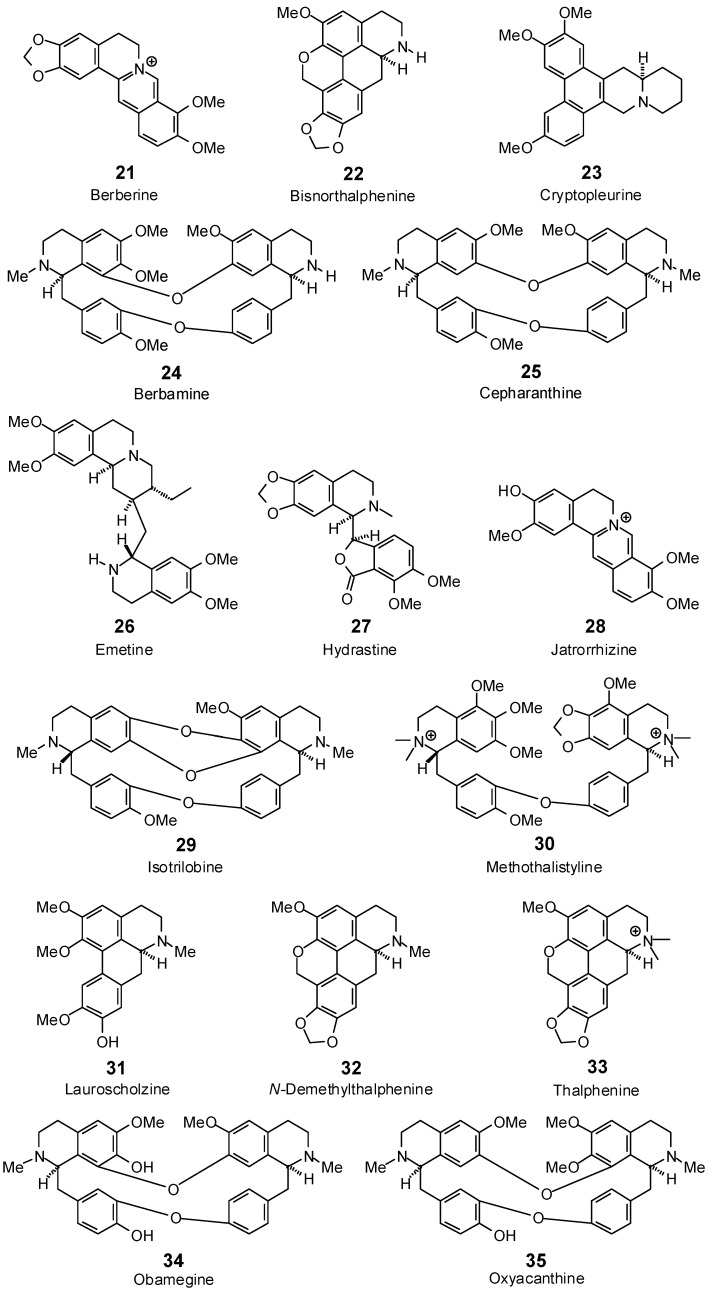

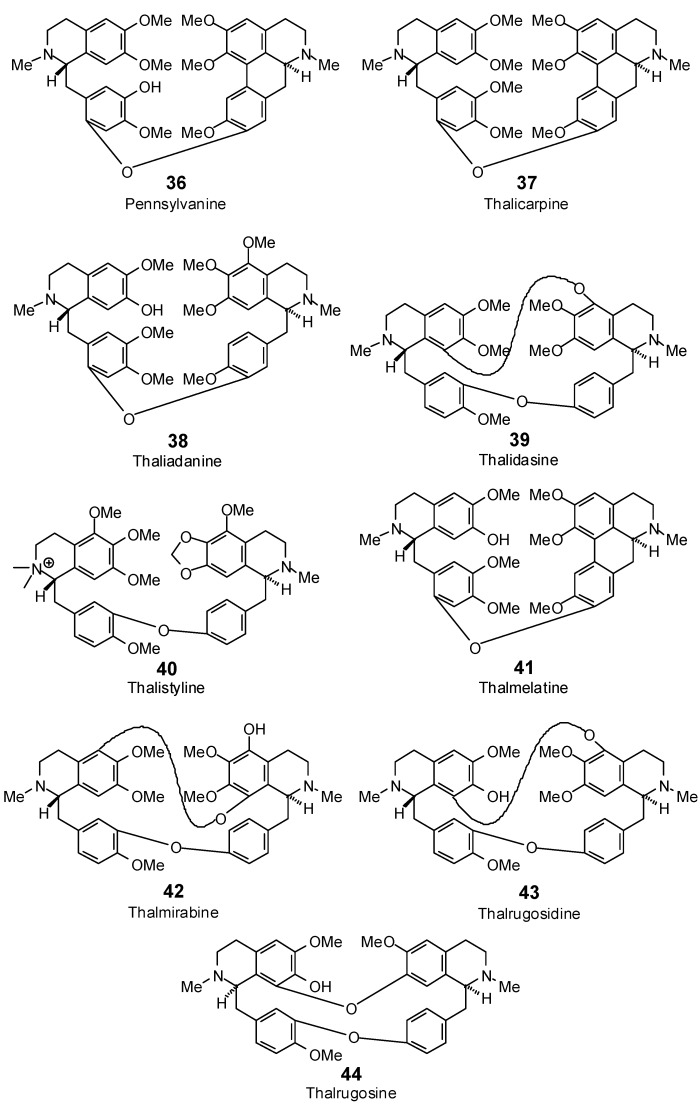
Isoquinoline alkaloids examined in this work.

**Figure 3:**
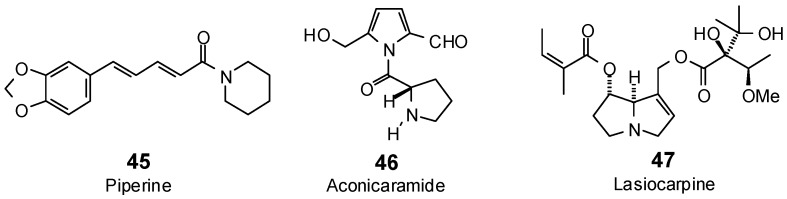

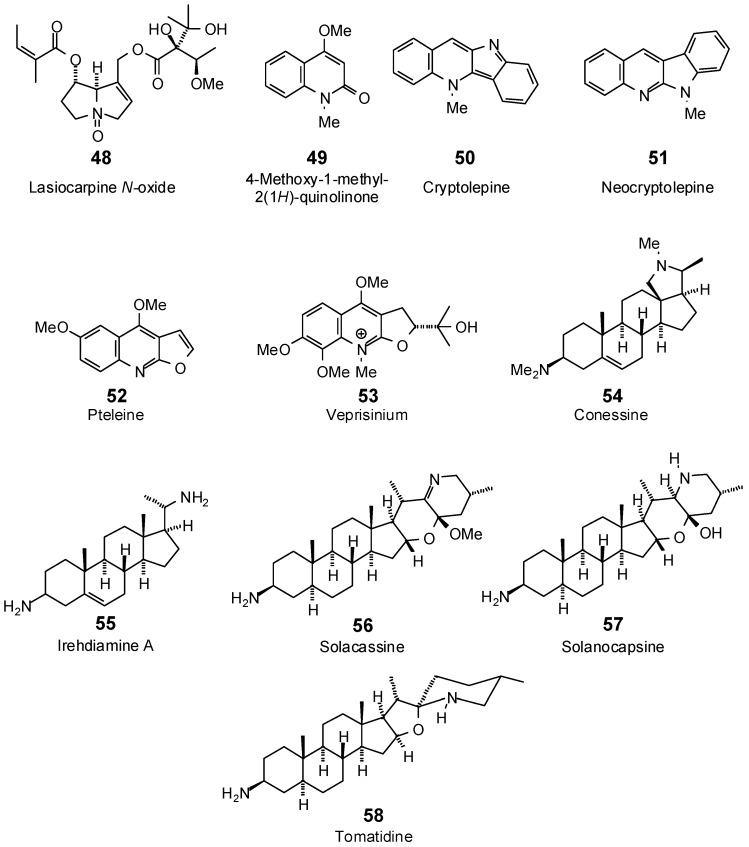
Piperidine, pyrrole, pyrrolizidine, quinoline, and steroidal alkaloids examined in this work.

**Figure 4:**
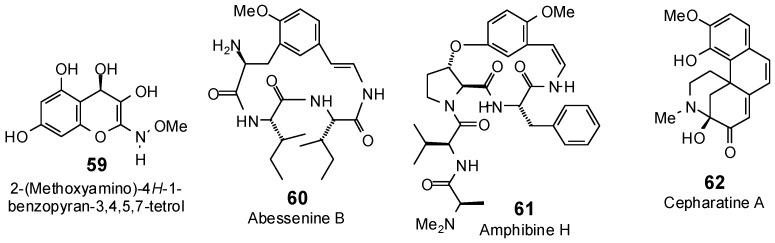

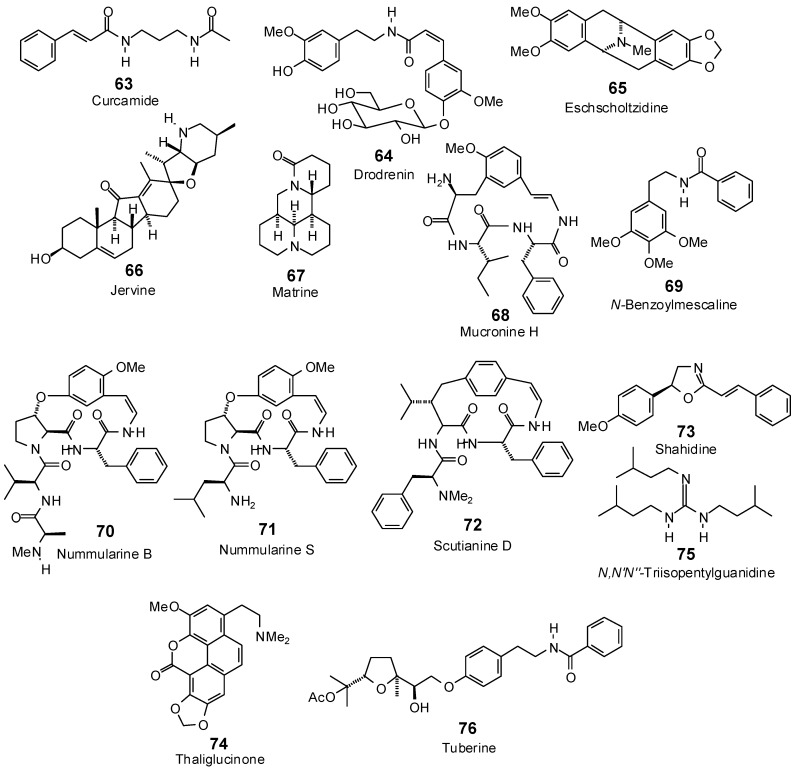
Miscellaneous alkaloids examined in this work.

**Figure 5:**
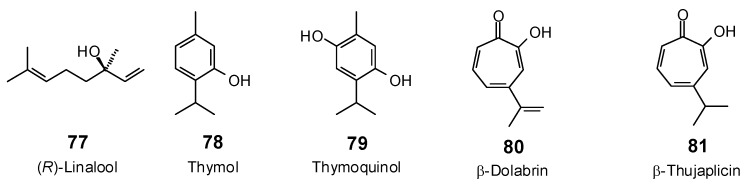
Monoterpenoids examined in this work.

**Figure 6:**
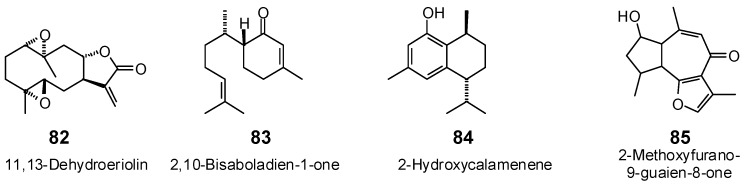

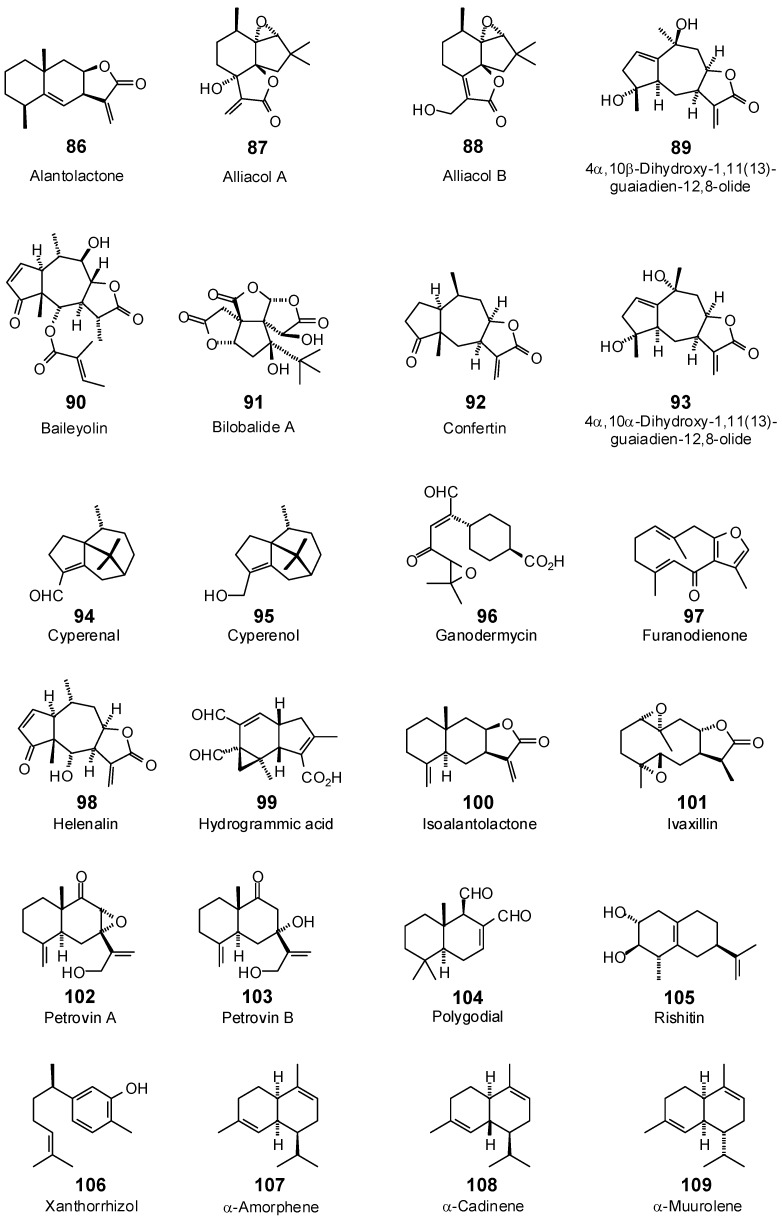

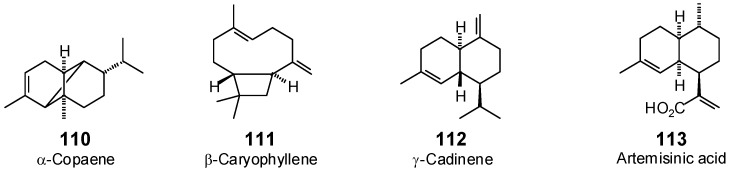
Sesquiterpenoids examined in this work.

**Figure 7:**
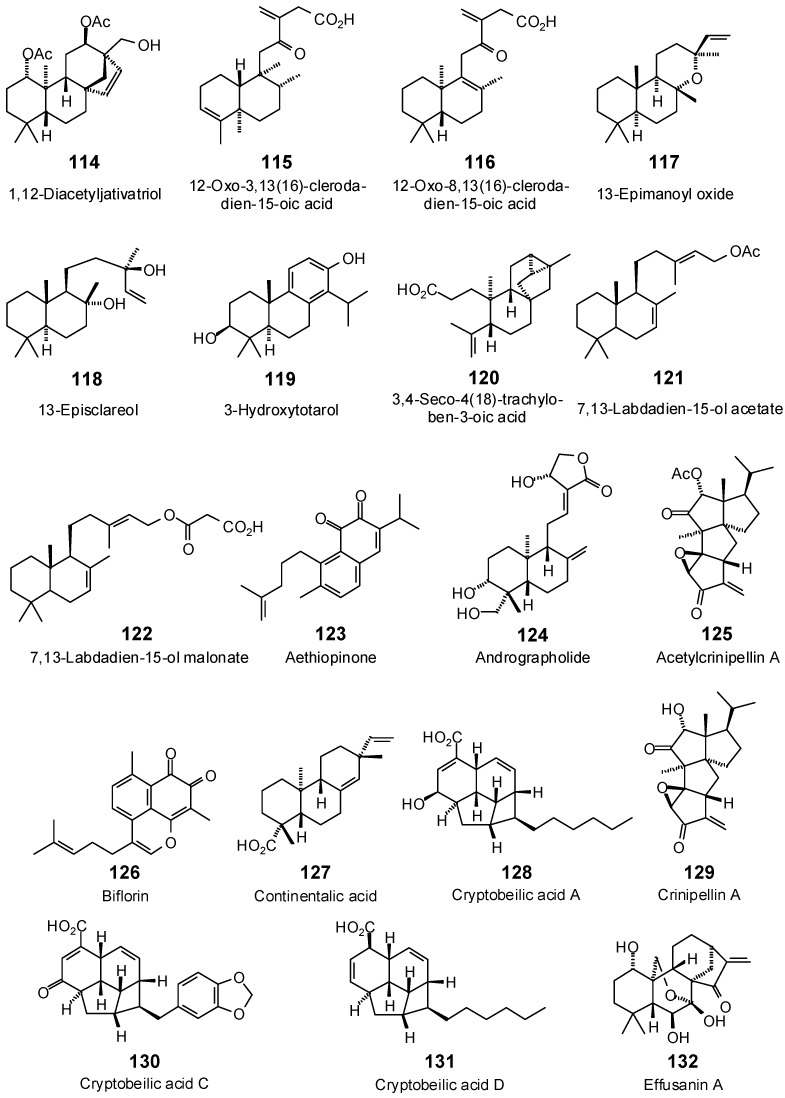

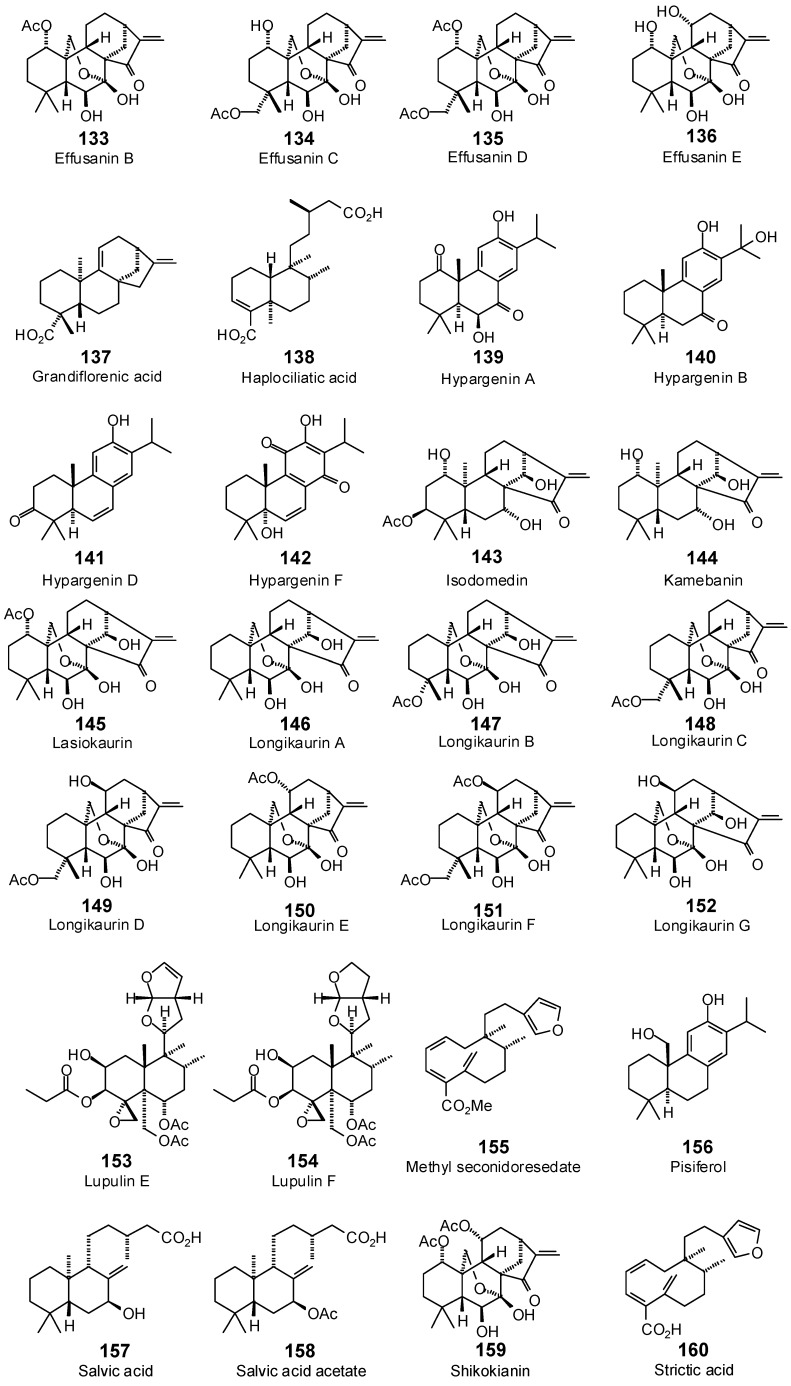

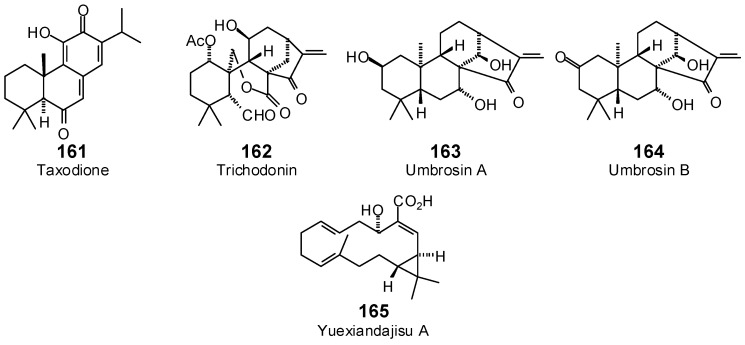
Diterpenoids examined in this work.

**Figure 8:**
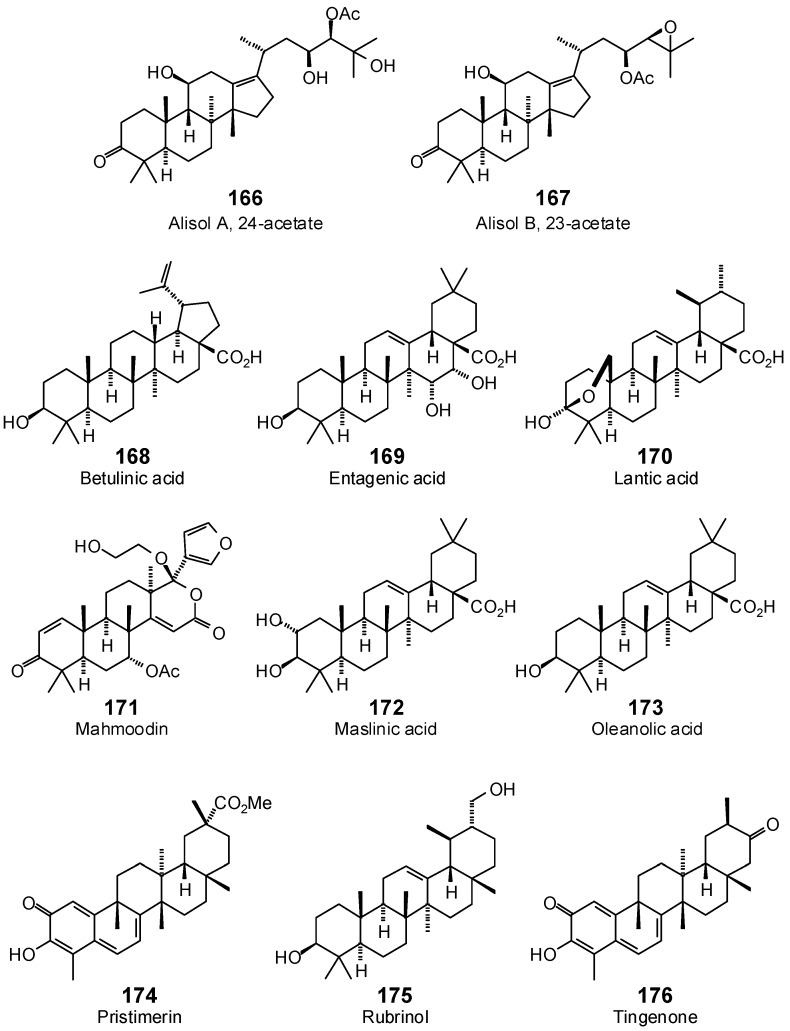
Triterpenoids examined in this work.

**Figure 9:**
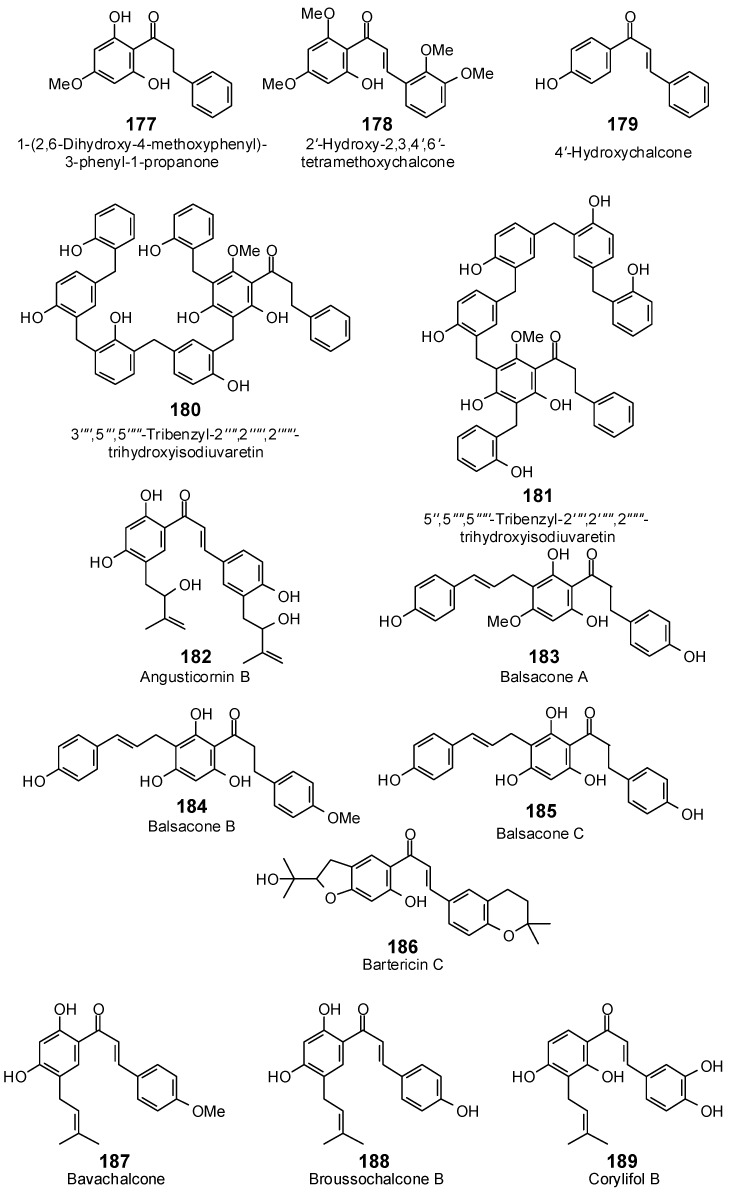

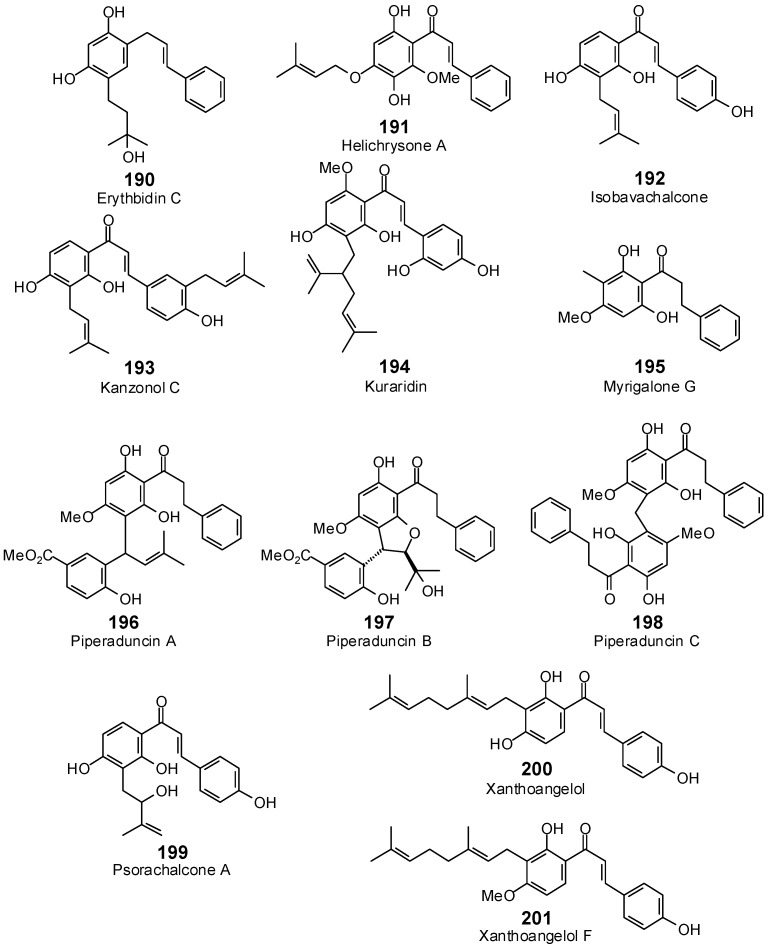
Chalcones examined in this work.

**Figure 10:**
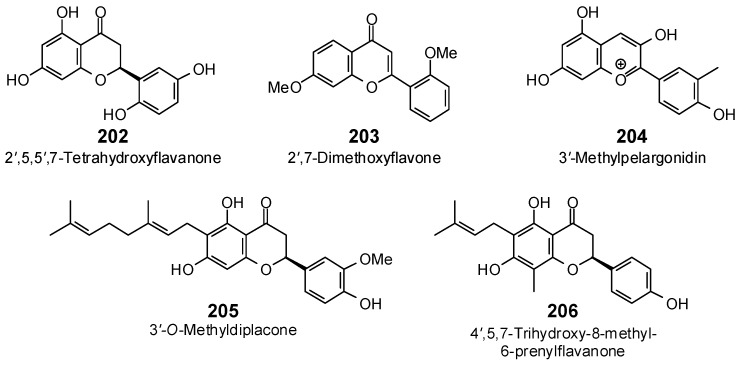

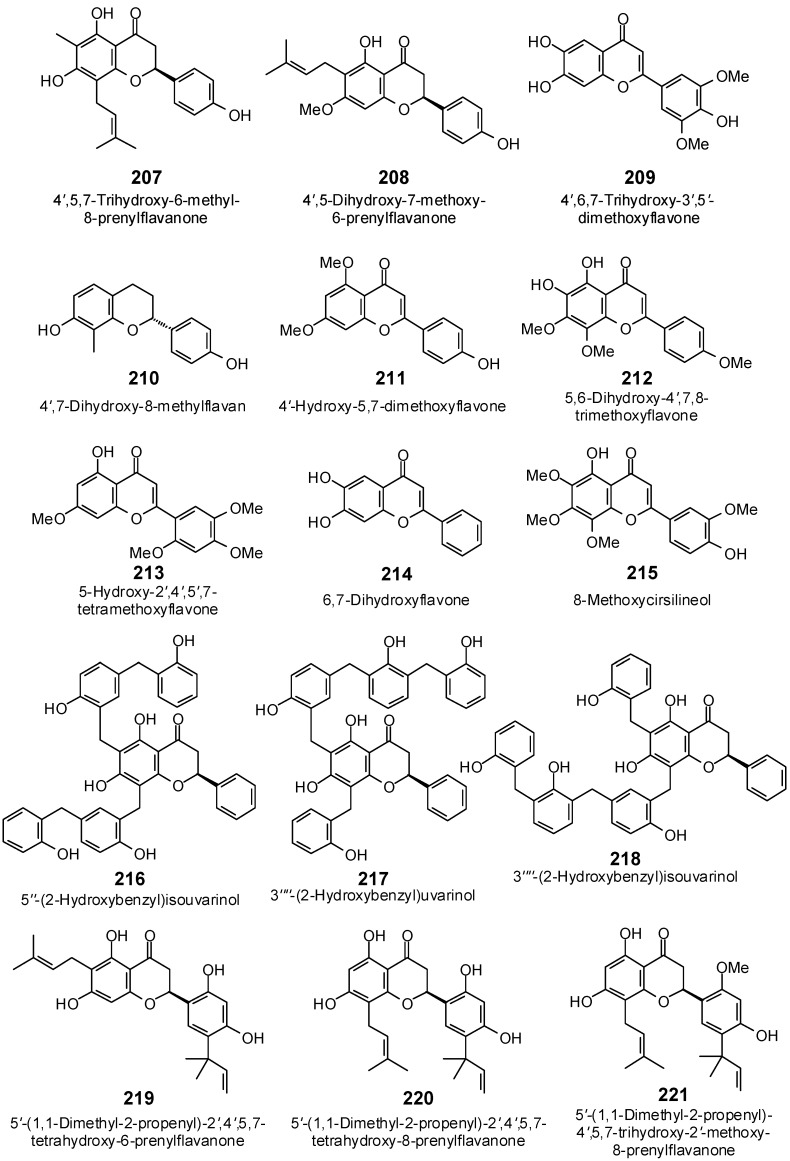

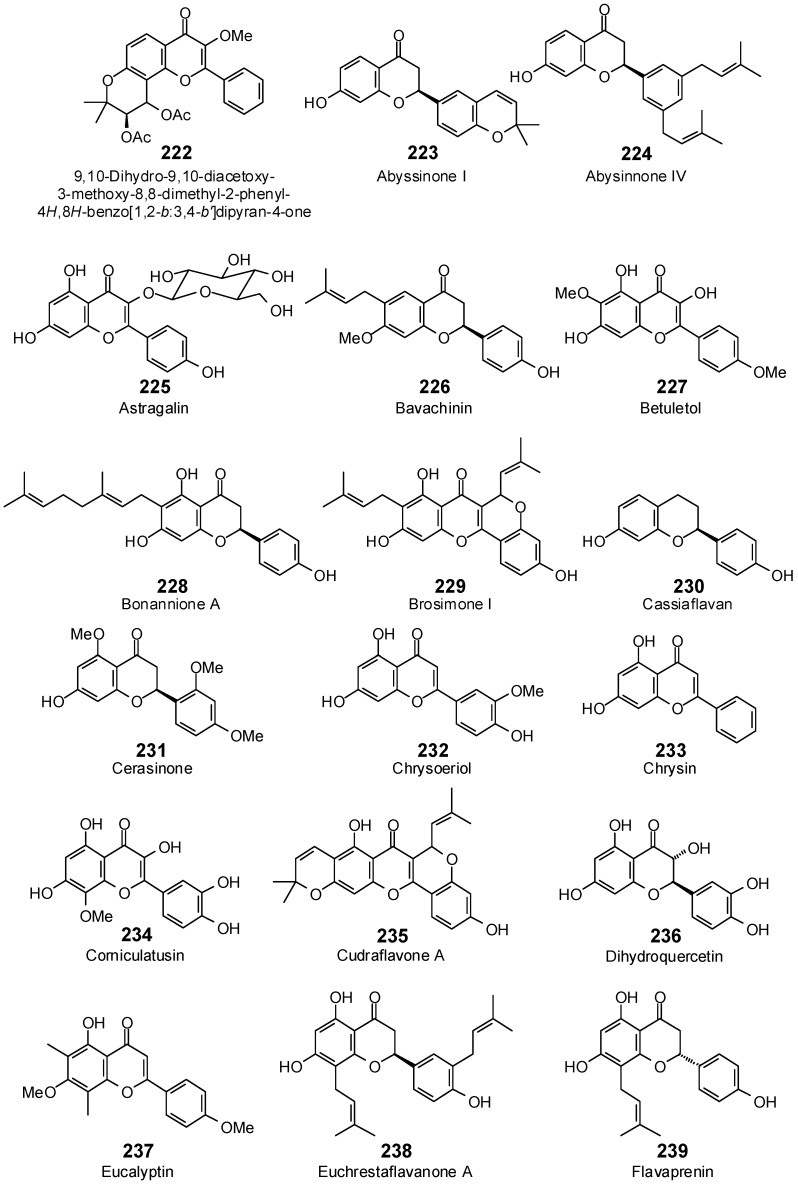

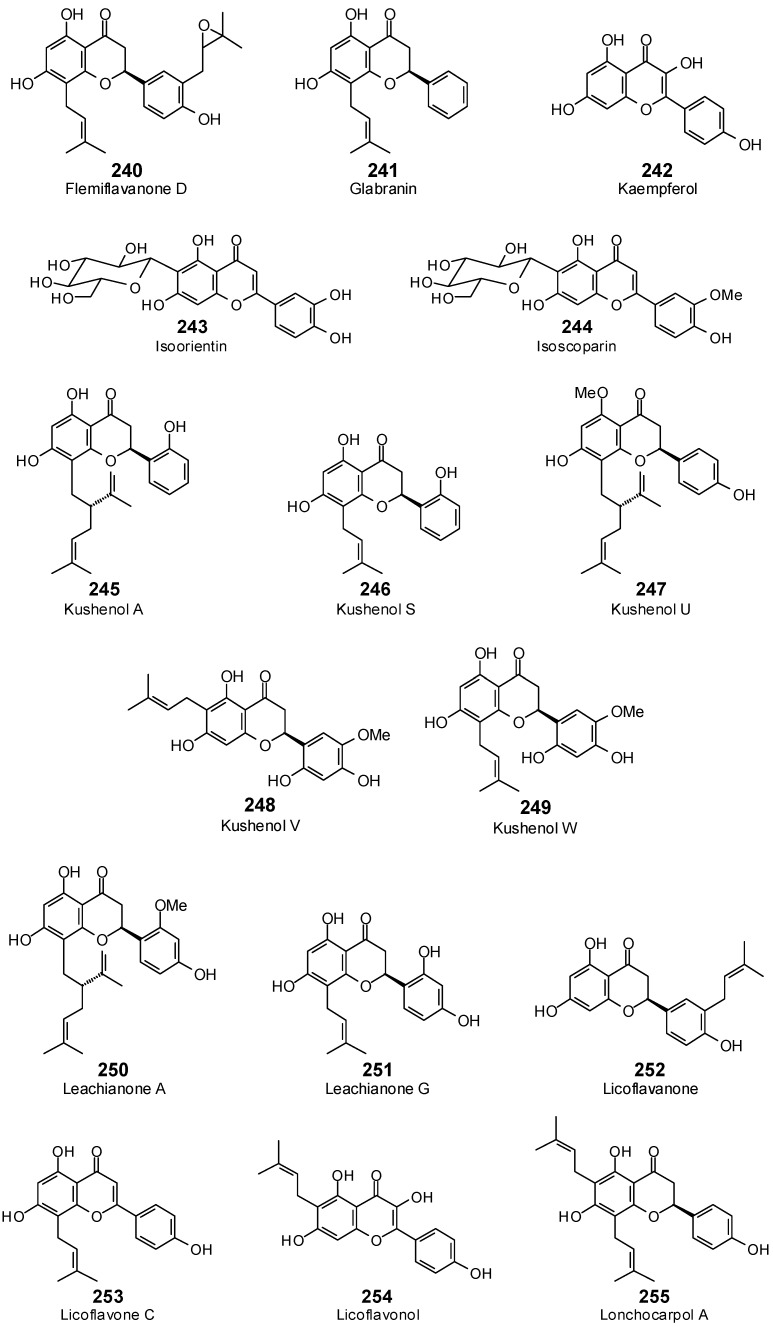

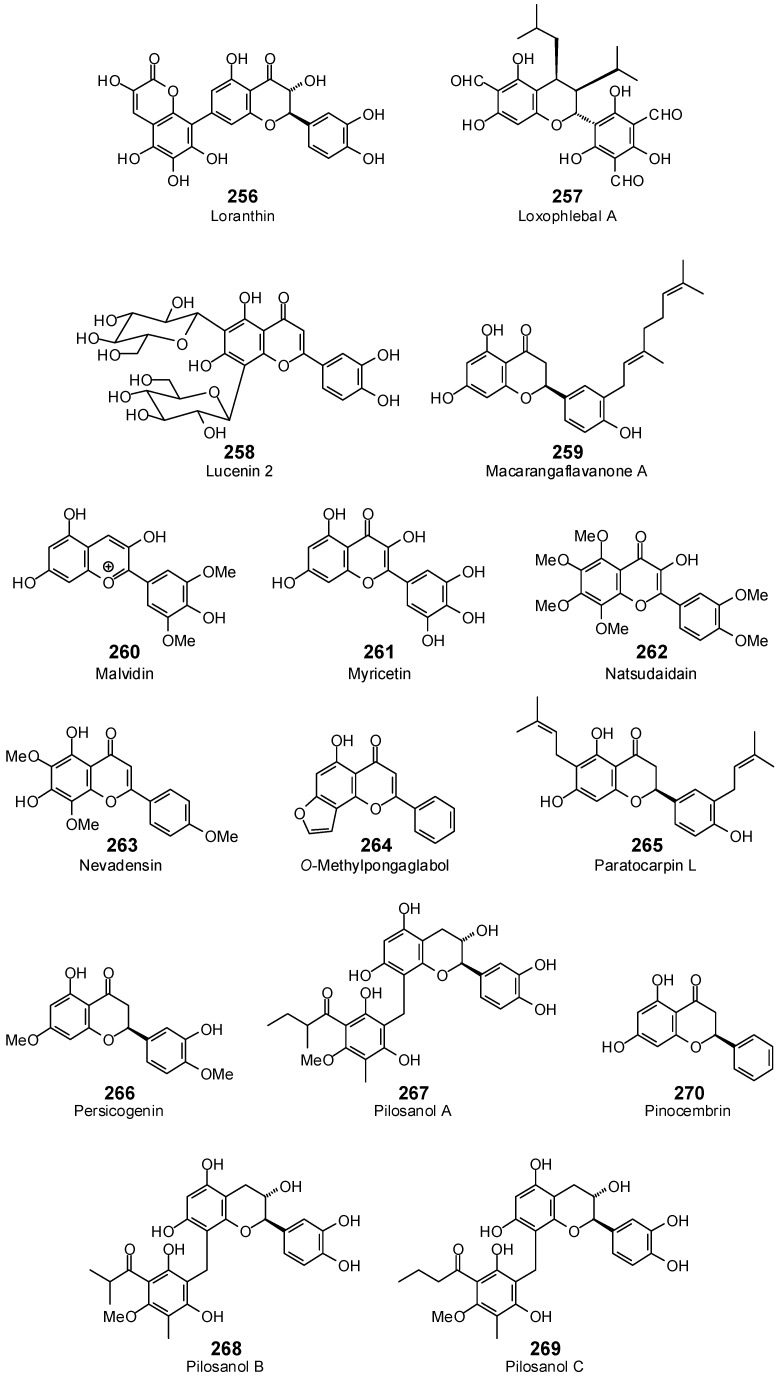

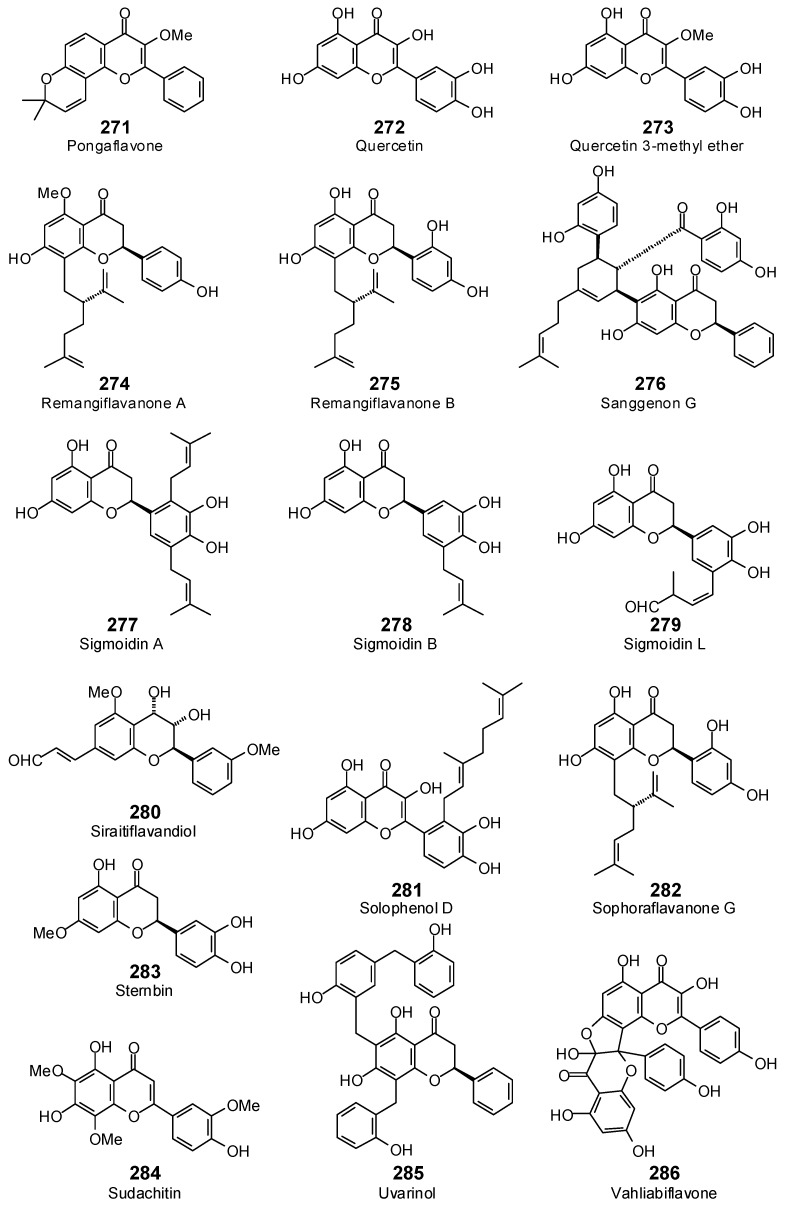

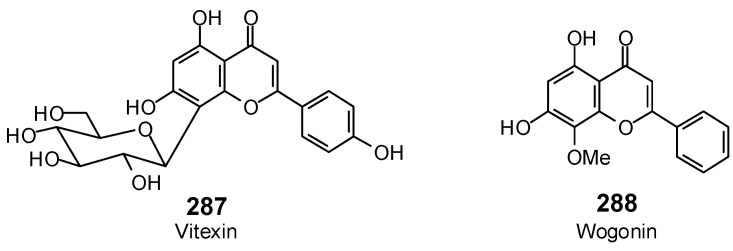
Flavonoids examined in this work.

**Figure 11:**
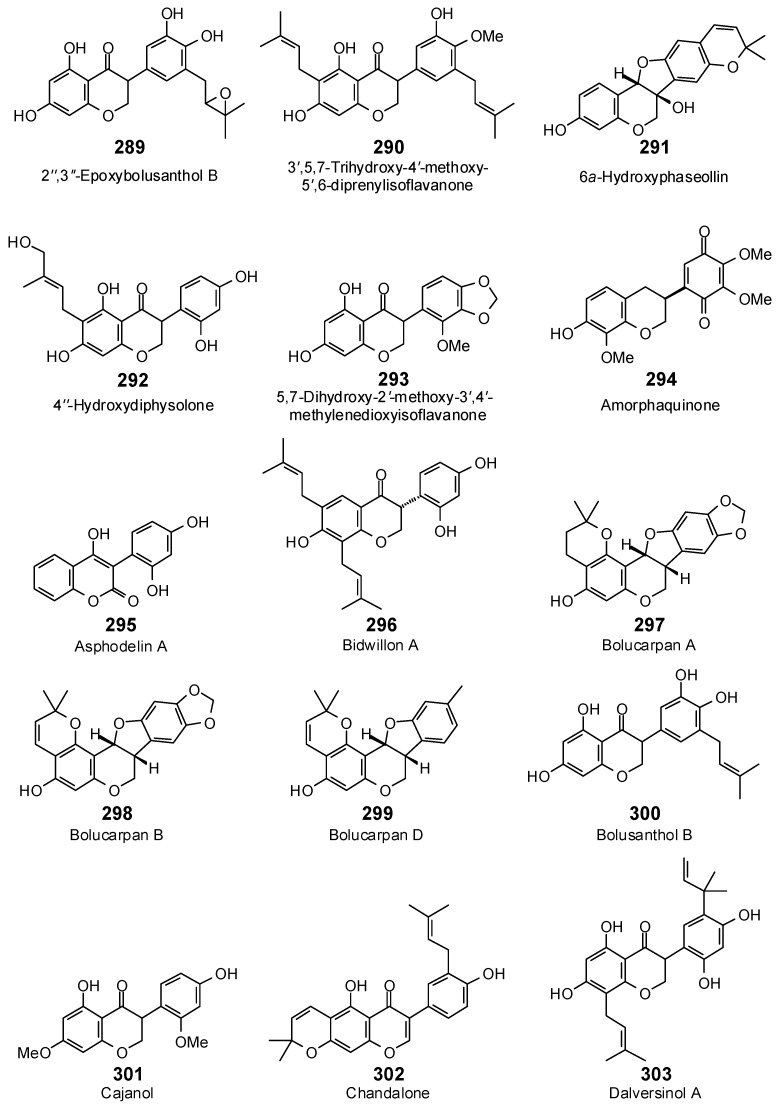

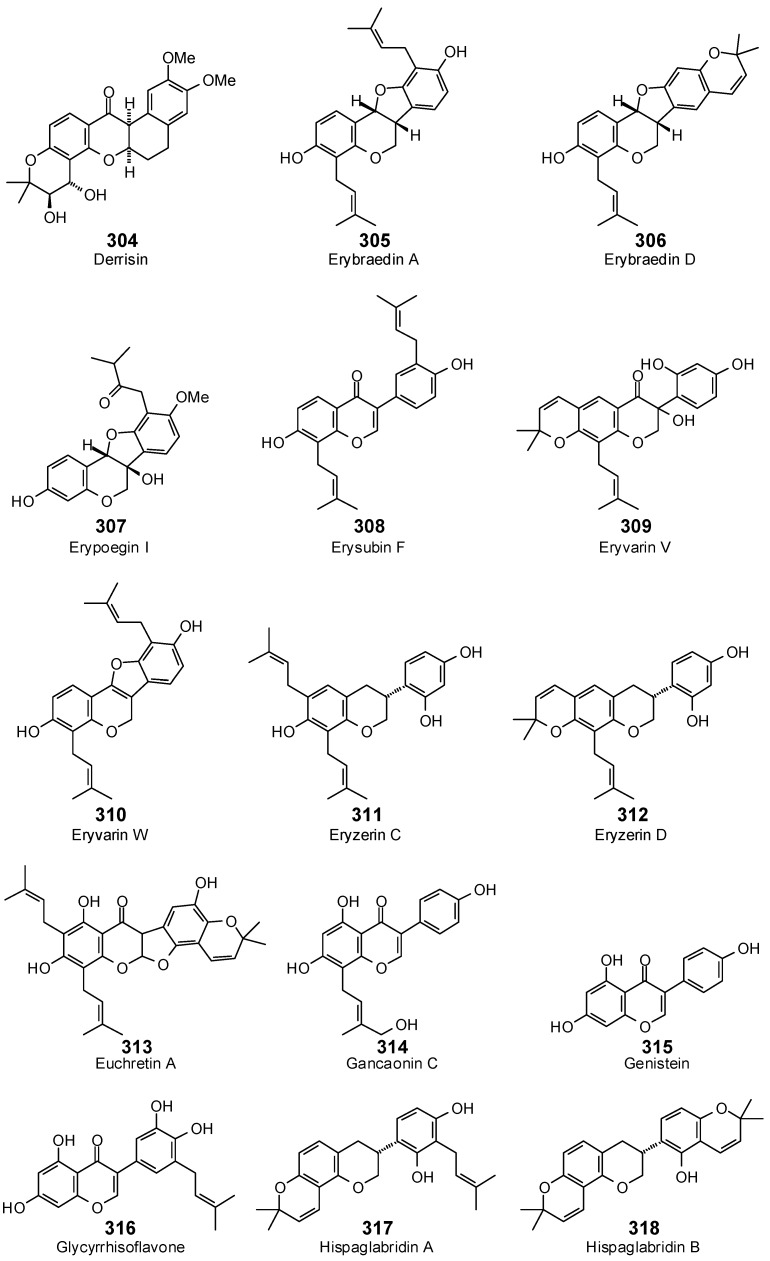

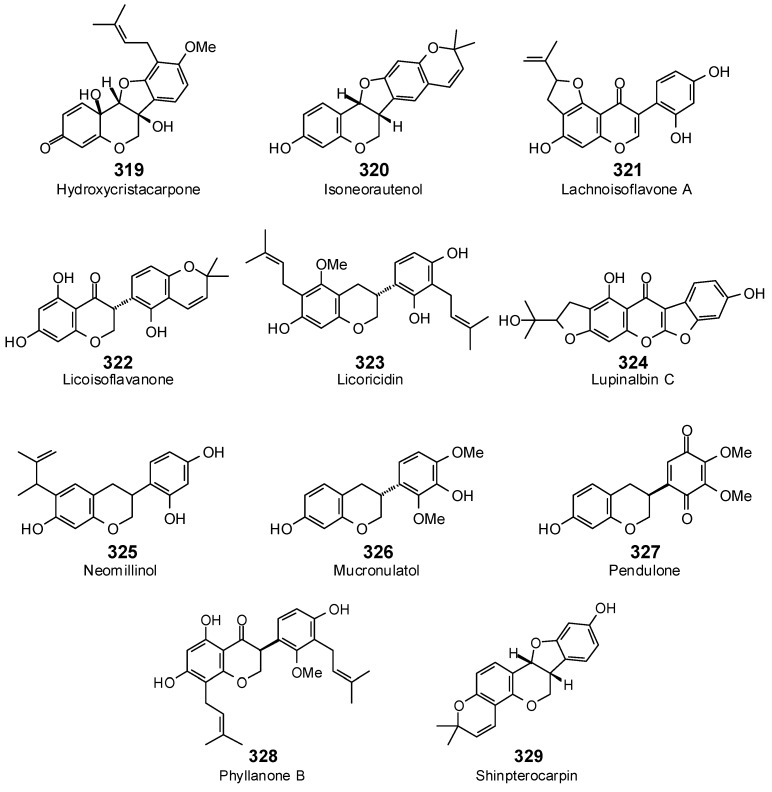
Isoflavonoids examined in this work.

**Figure 12:**
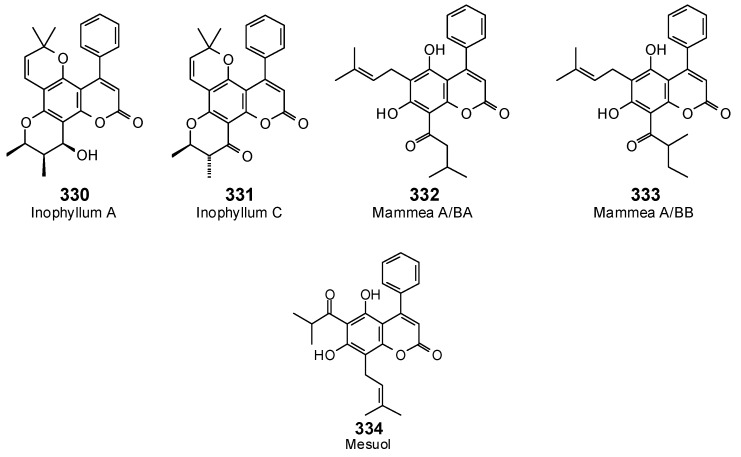
Neoflavonoids examined in this work.

**Figure 13:**
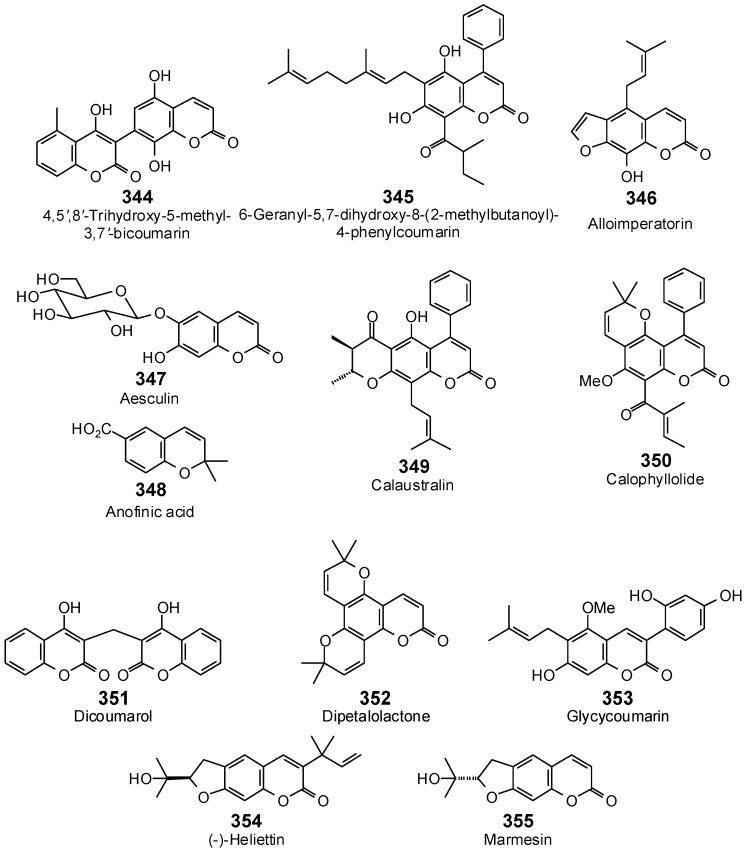
Coumarins examined in this work.

**Figure 14:**
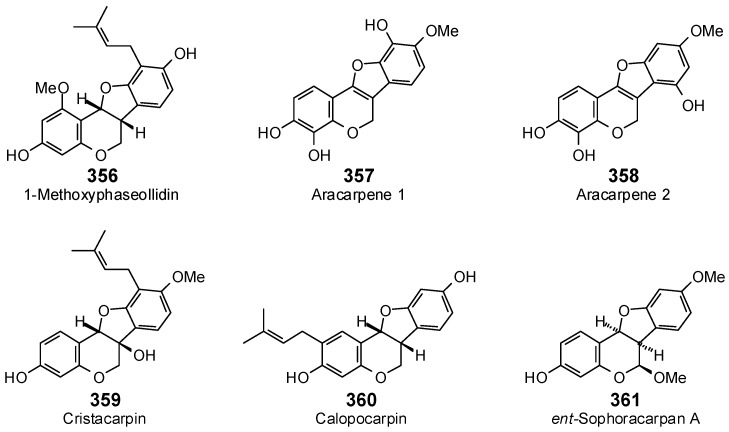

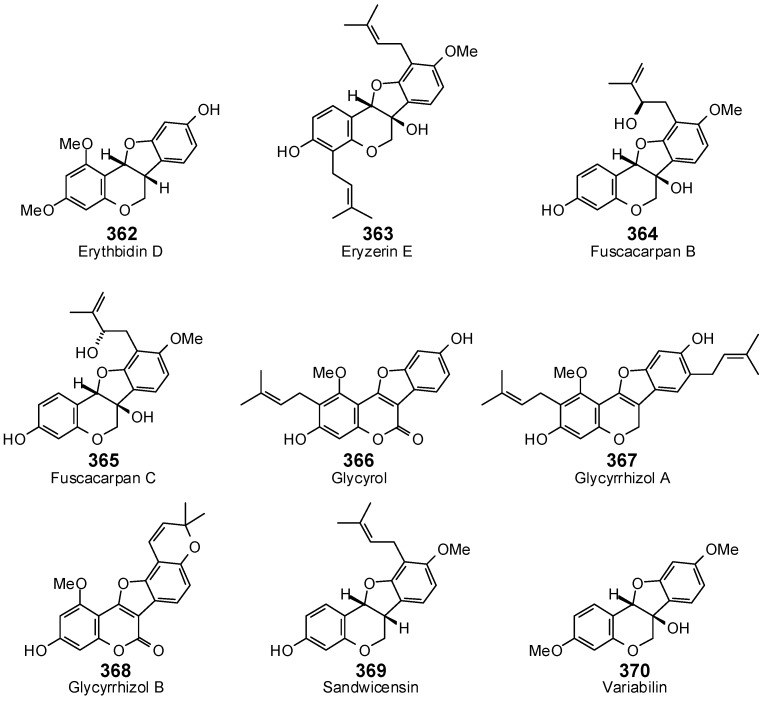
Pterocarpans examined in this work.

**Figure 15:**
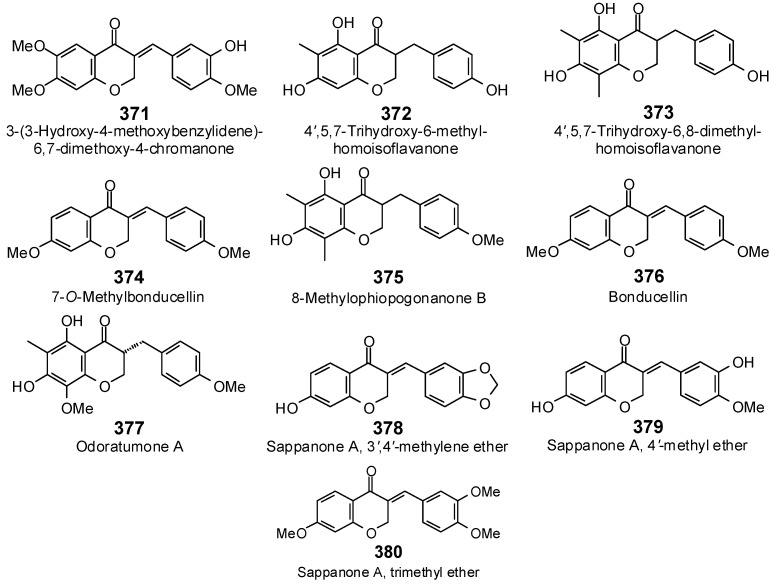
Chromones examined in this work.

**Figure 16:**
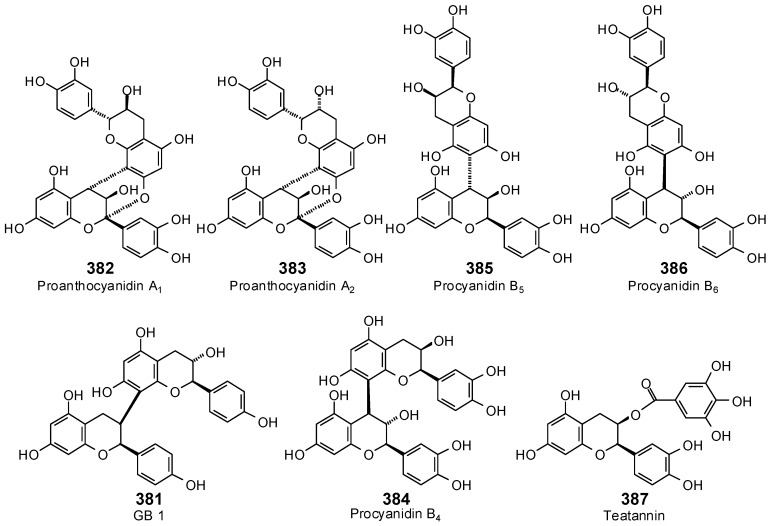
Condensed tannins examined in this work.

**Figure 17:**
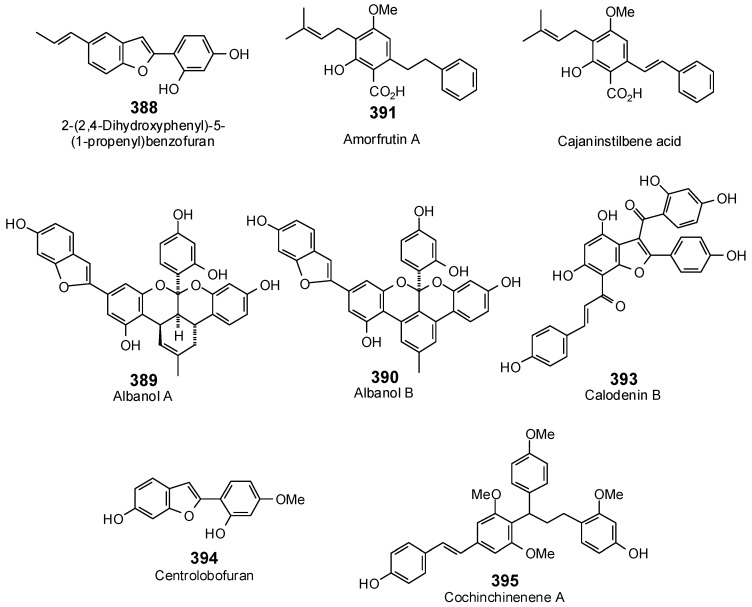

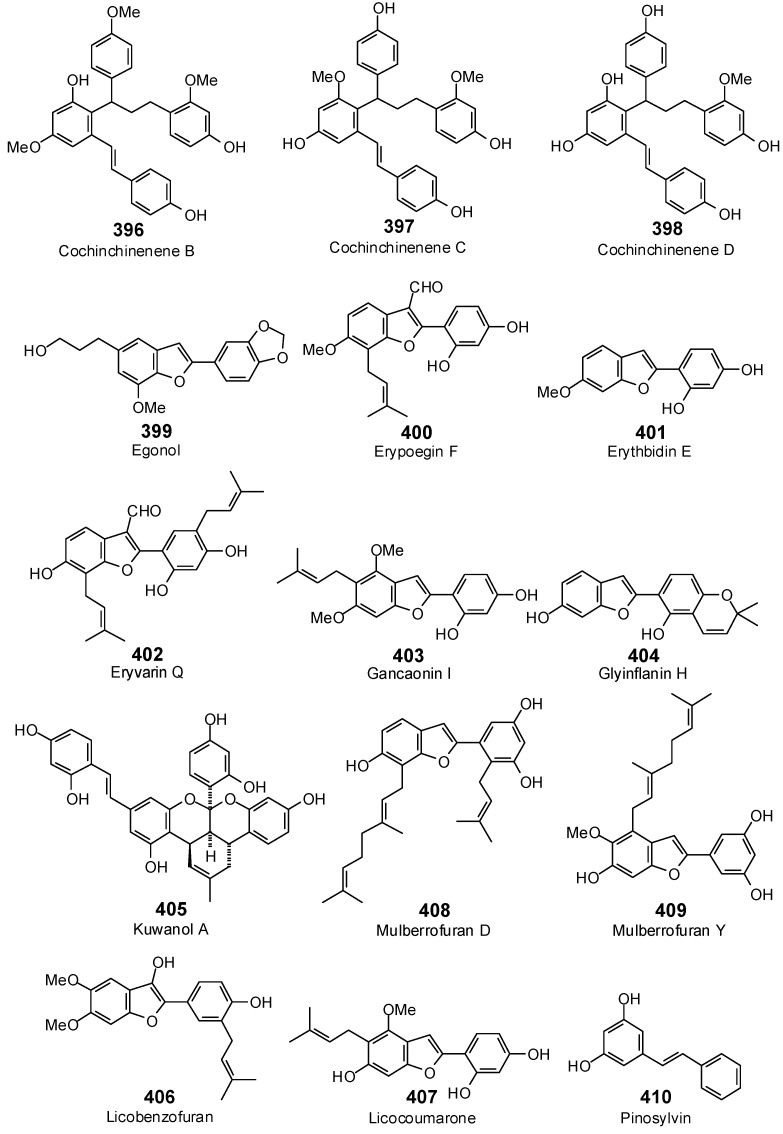

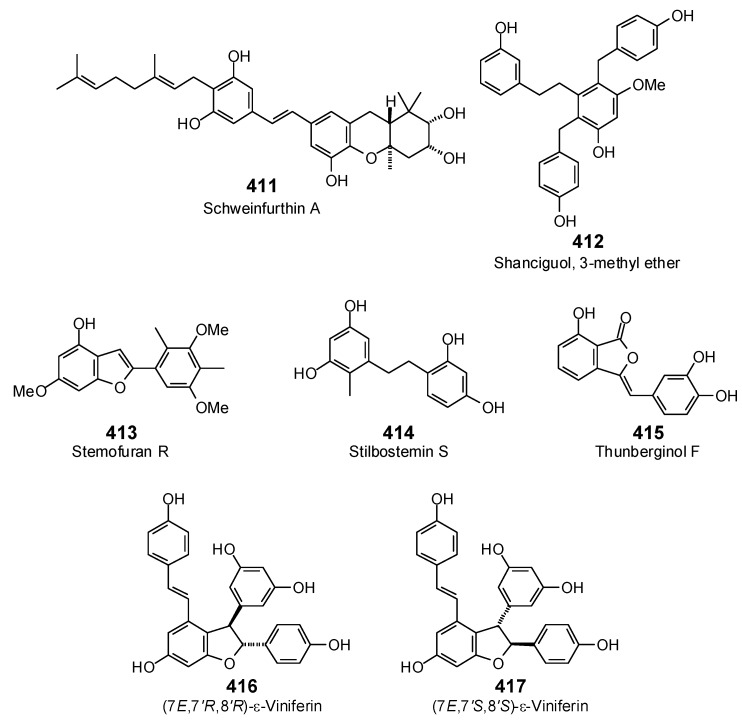
Stilbenoids examined in this work.

**Figure 18:**
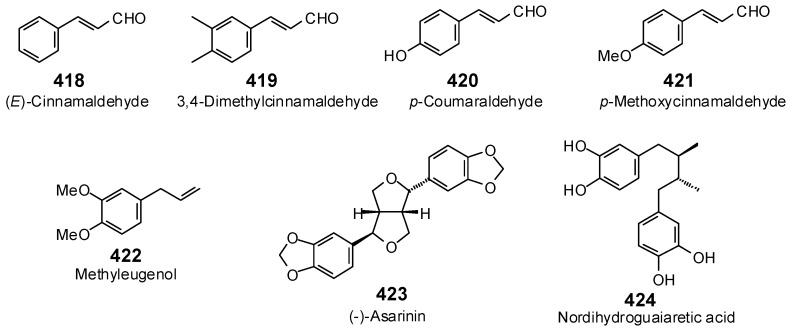
Phenylpropanoids and lignans examined in this work.

**Figure 19:**
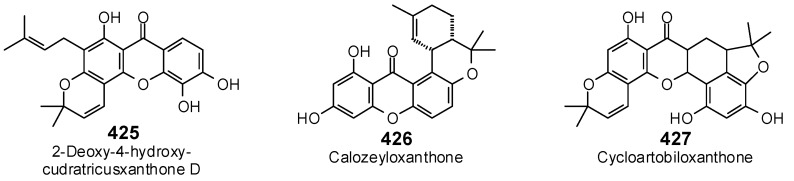

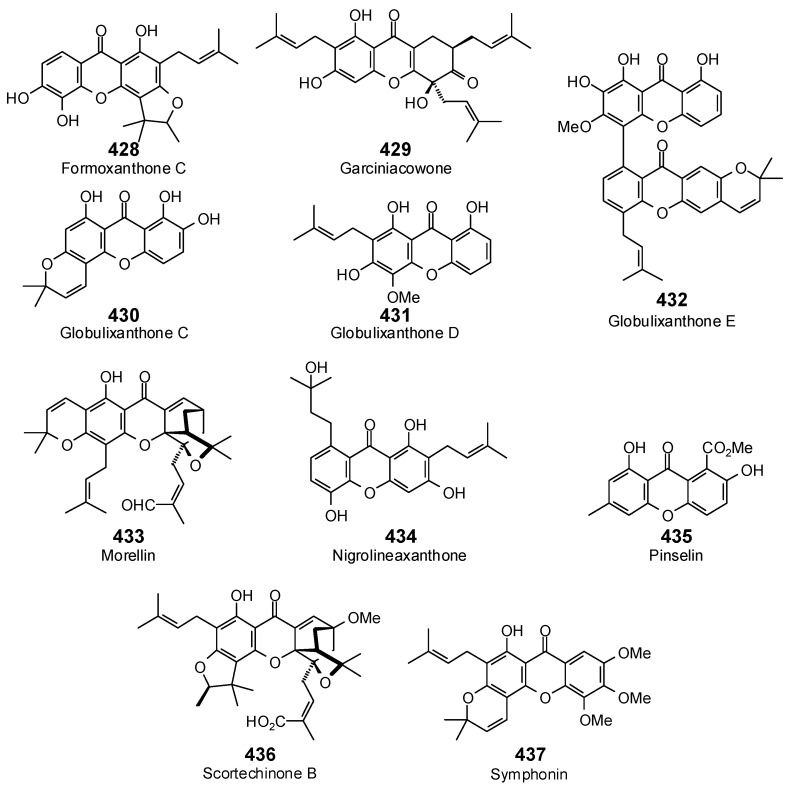
Xanthones examined in this work.

**Figure 20:**
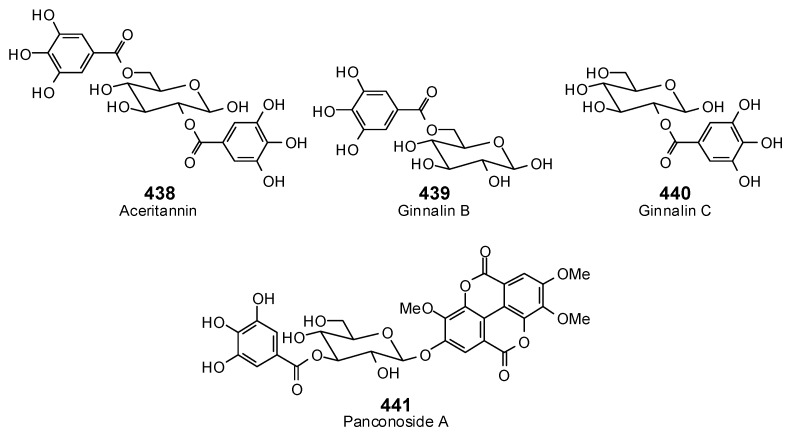
Hydrolyzable tannins examined in this work.

**Figure 21:**
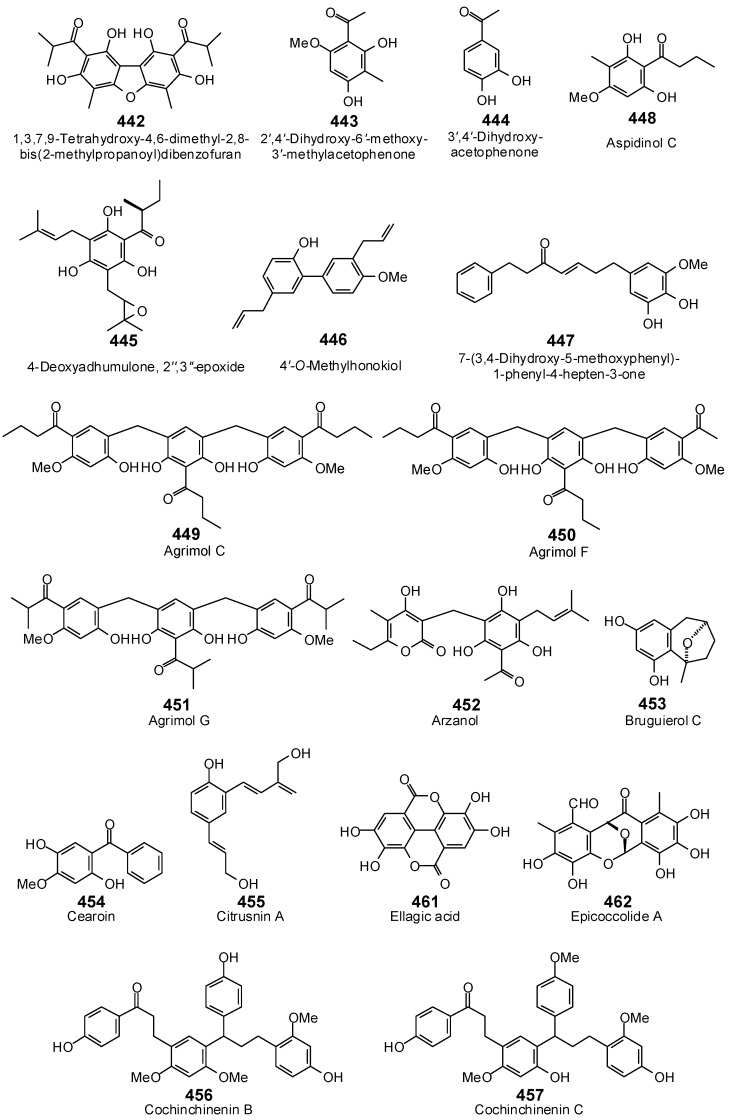

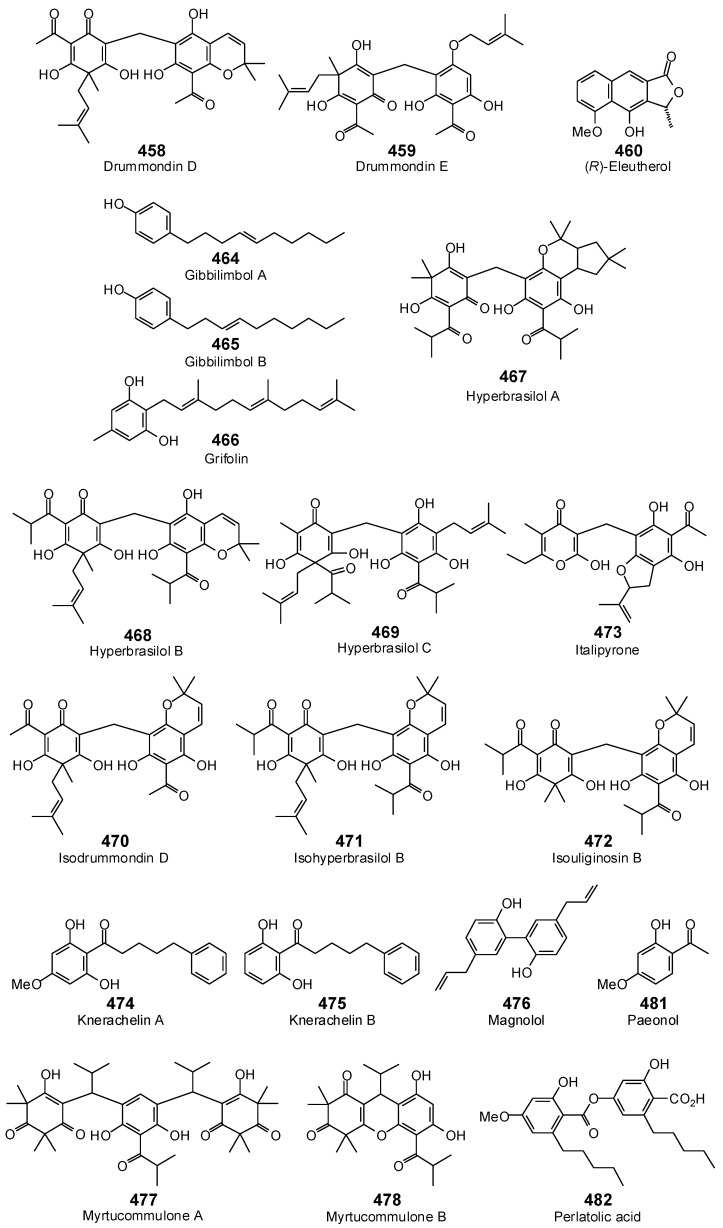

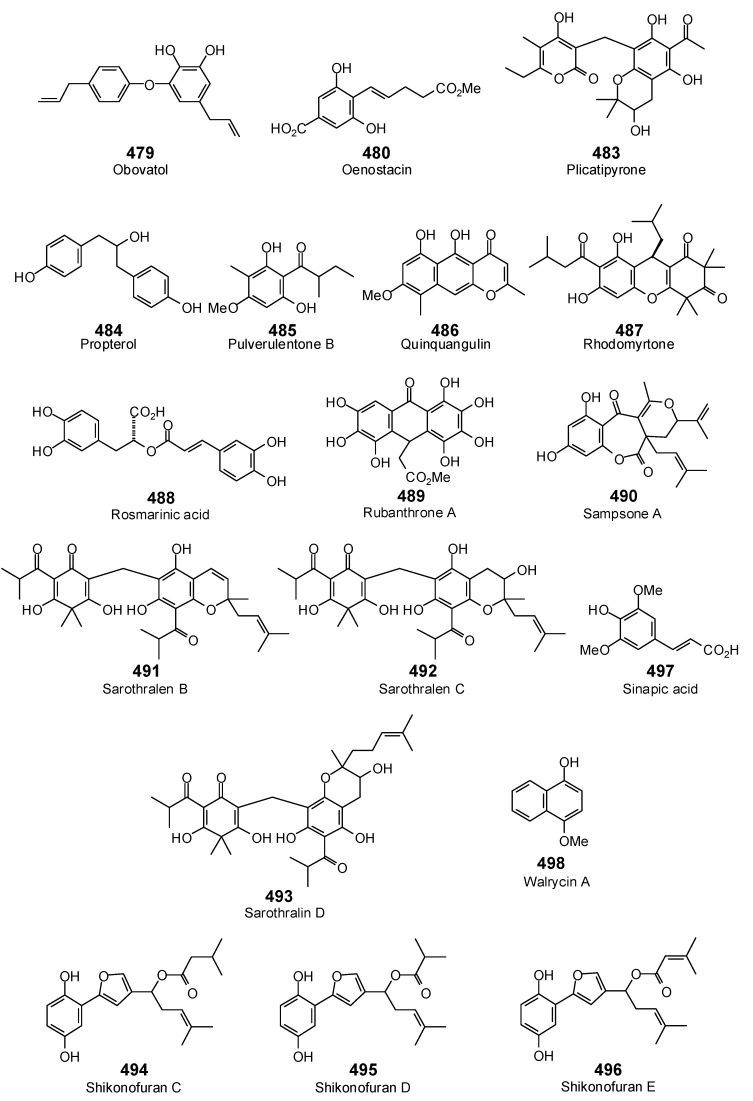
Miscellaneous phenolic phytochemicals examined in this work.

**Figure 22:**
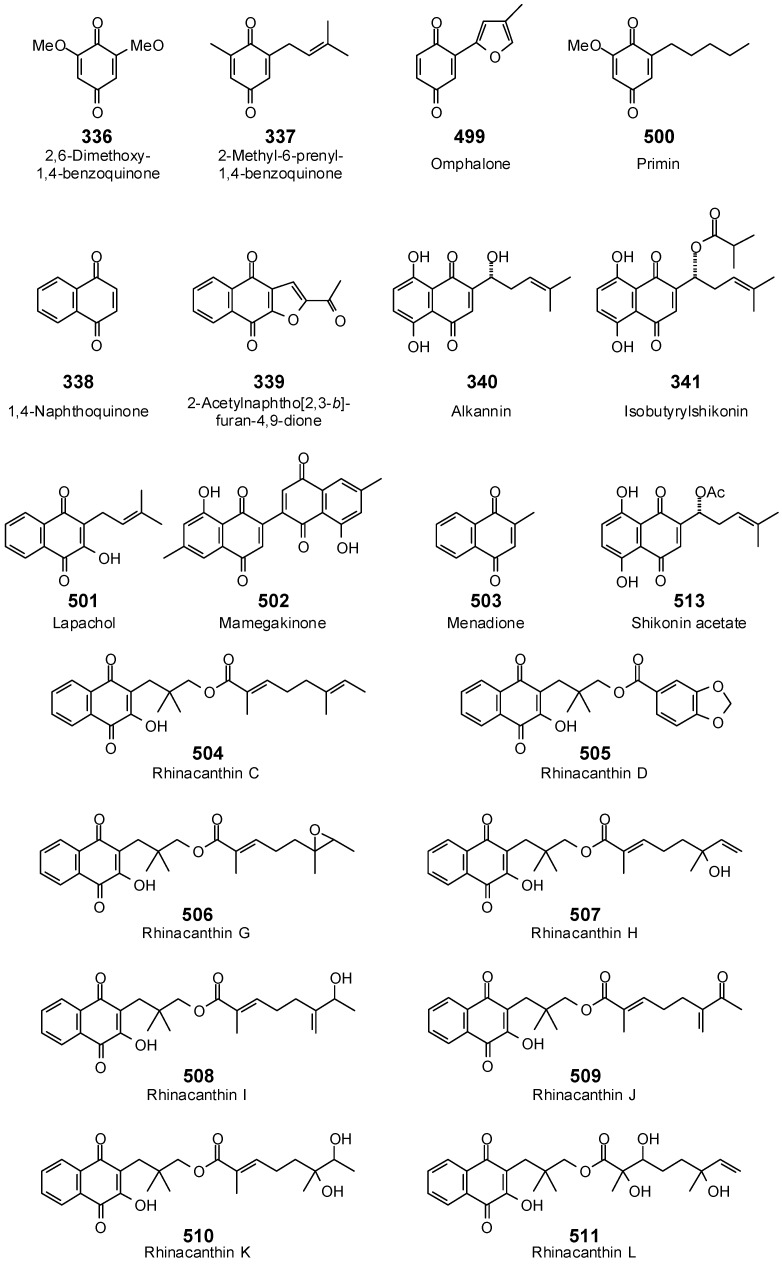

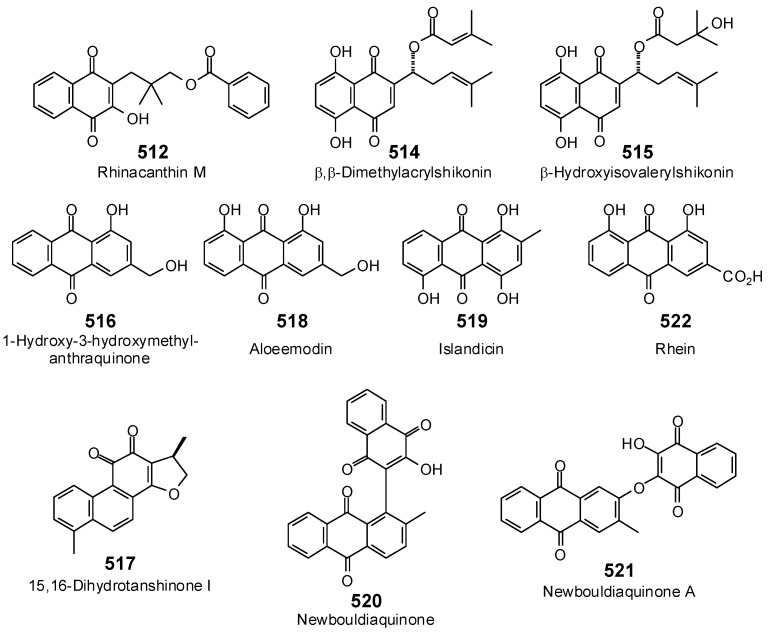
Quinones examined in this work.

**Figure 23:**
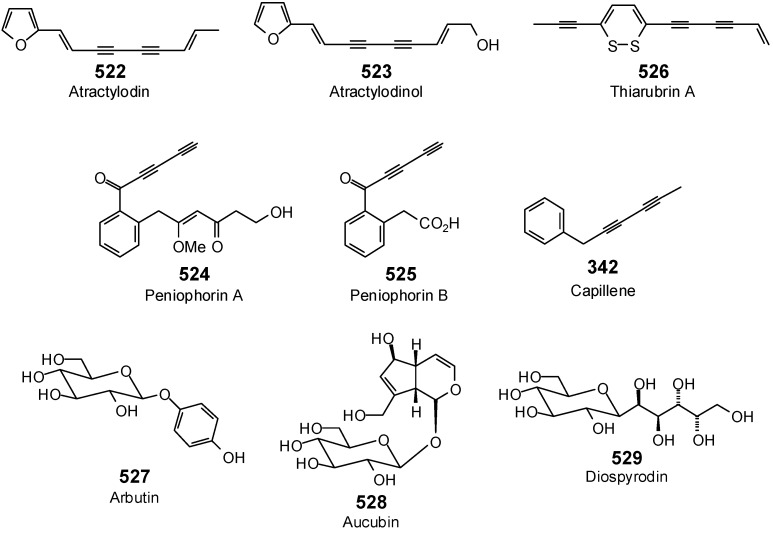

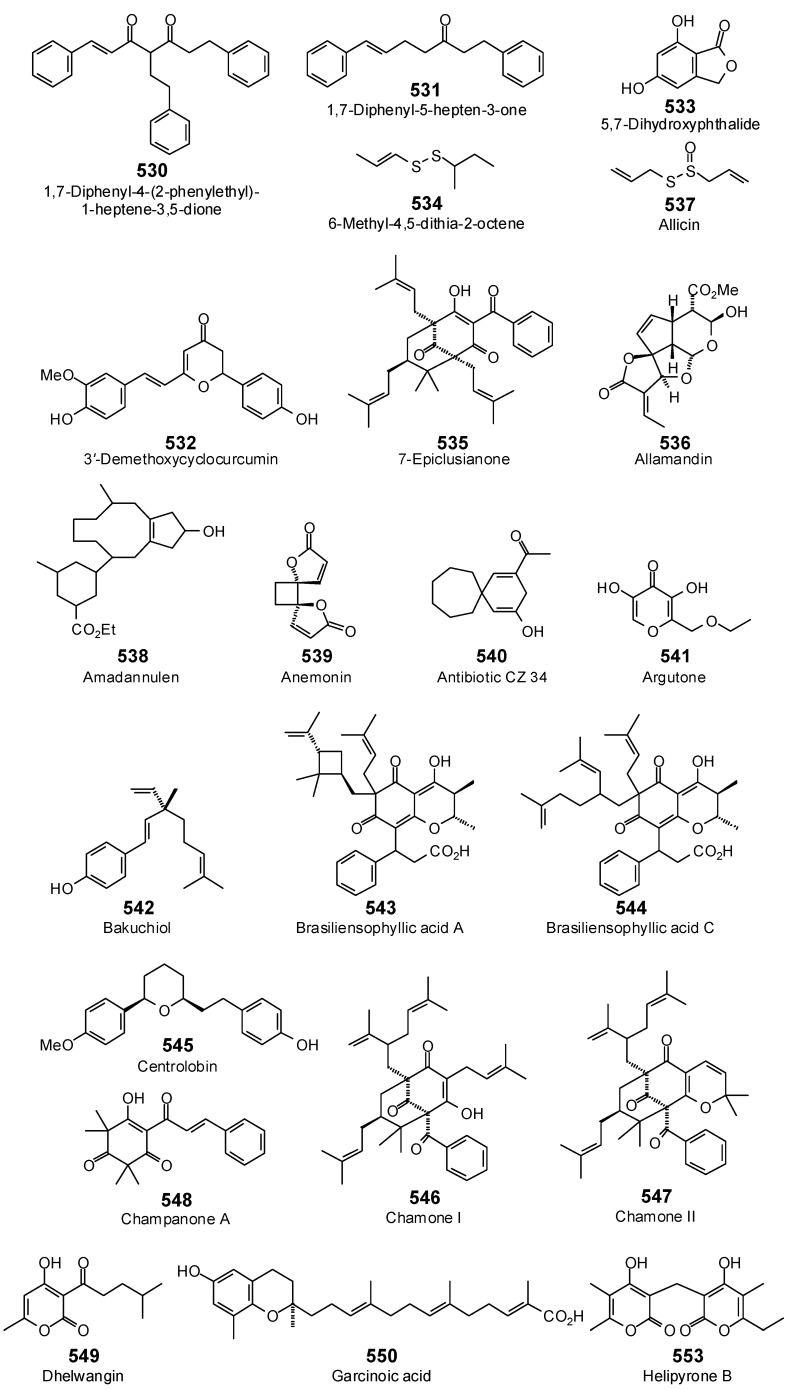

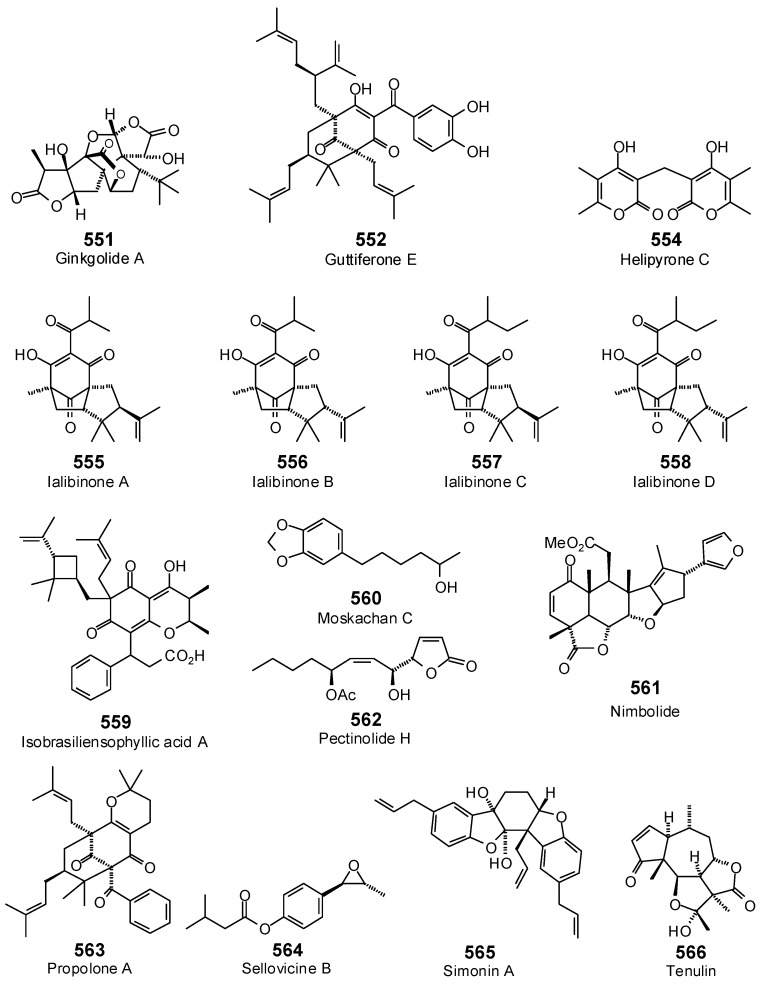
Acetylene, glucoside, and other miscellaneous phytochemicals examined in this work.

The Molegro Virtual Docking program [59,57] was used to carry out in-silico protein-ligand docking studies using known antibacterial phytochemicals with the structures of verified bacterial protein drug targets. A total of 561 antibacterial phytochemicals listed in the *Dictionary of Natural Products* [19] were studied, including 77 alkaloids (17 indole alkaloids, 27 isoquinoline alkaloids, 4 steroidal alkaloids, and 28 miscellaneous alkaloids), 99 terpenoids (5 monoterpenoids, 31 sesquiterpenoids, 52 diterpenoids, and 11 triterpenoids), 309 polyphenolics (87 flavonoids, 25 chalcones, 41 isoflavonoids, 5 neoflavonoids, 12 pterocarpans, 10 chromones, 7 condensed tannins, 11 coumarins, 30 stilbenoids, 2 lignans, 5 phenylpropanoids, 13 xanthones, 5 hydrolyzable tannins, and 56 miscellaneous phenolics), 30 quinones, and 46 miscellaneous phytochemicals (see Figure 1, Figure 2, Figure 3, Figure 4, Figure 5, Figure 6, Figure 7, Figure 8, Figure 9, Figure 10, Figure 11, Figure 12, Figure 13, Figure 14, Figure 15, Figure 16, Figure 17, Figure 18, Figure 19, Figure 20, Figure 21, Figure 22 and Figure 23), with six bacterial protein targets (peptide deformylase, DNA gyrase/topoisomerase IV, UDP-galactose mutase, protein tyrosine phosphatase, cytochrome P450 CYP121, and NAD^+^-dependent DNA ligase). As a test for docking accuracy, the co-crystallized ligands from each protein structure were re-docked into the proteins. The docking energies and root-mean squared deviations (RMSD) are summarized in Table 1. In order to correct for the known biasing of docking energies (E_dock_) with increasing molecular weight (MW) [60,61,62,63,64,65], we have also determined a normalized docking score (DS_norm_) based on the molecular weight: DS_norm_ = 7.2 × E_dock_/MW^⅓^.

### 3.1. Bacterial Peptide Deformylase

MolDock docking energies (E_dock_) and normalized docking scores (DS_norm_) of antibacterial phytochemical ligands with bacterial peptide deformylase enzyme structures are summarized in Table 2. There were very few alkaloids docking to the bacterial peptide deformylase protein targets with notable docking scores. Those alkaloids that had large exothermic docking energies usually violated Lipinski’s rule of five [66], with molecular weights >500 or hydrogen-bond acceptor atoms >10. Tuberine (76), however, did show excellent docking to *Escherichia coli* peptide deformylase (EcPDF) (E_dock_ = −136.7 kJ/mol; DS_norm_ = −126.5) compared to docking of the ligand with human PDF (HsPDF, E_dock_ = −121.7 kJ/mol) or compared with the docking energy of the co-crystallized ligand actinonin (E_dock_ = −111.8 kJ/mol). (+)-Tuberine (76), isolated from *Haplophyllum tuberculatum*, has shown antibacterial properties against *Staphylococcus aureus* and *Bacillus* subtilis, as well as *E. coli* [67,68].

Several chalcones exhibited particularly strong docking properties with bacterial PDFs. Most notably, angusticornin B (182) docked strongly with EcPDF (E_dock_ = −143 kJ/mol), *Mycobacterium tuberculosis* PDF (MtPDF, E_dock_ = −134.4 kJ/mol), *Pseudomonas aeruginosa* PDF (PaPDF, E_dock_ = −134.7 kJ/mol), and *Streptococcus pneumoniae* PDF (SpPDF, E_dock_ = −131.4 kJ/mol); more strongly than with HsPDF (−126.7 kJ/mol). Balsacone B (184) and balsacone C (185) docked well with SpPDF; kanzonol C (193) docked well with EcPDF, SpPDF, but also with HsPDF; piperaduncin B (197) docked well with EcPDF, SaPDF, and SpPDF, but also with HsPDF; xanthoangelol (200) showed remarkable docking properties with EcPDF, MtPDF, PaPDF, SaPDF (*Staphylococcus aureus* PDF), and SpPDF, but also with HsPDF; and xanthoangelol F (201) docked well with MtPDF. Angusticornin B (182) has shown activity against *E. coli* and *P. aeruginosa* [69]. Apparently the balsacones B and C (184, 185) were not screened for activity against *S. pneumoniae*, but these compounds have shown activity against Gram-positive *S. aureus* [70]. Kanzonol C (193) has shown broad spectrum antibacterial activity including inhibition of *E. coli* [71]. Although piperaduncum B (197) was active against *Micrococcus luteus* and *Bacillus subtilis*, it was inactive against *E. coli* [72]. Neither xanthoangelol (200) nor xanthoangelol F (201) showed activity against Gram-negative bacteria, but both were active against Gram-positive organisms [73,74].

Four antibacterial flavonoids, 3′-*O*-methyldiplacone (205), 5′-(1,1-dimethyl-2-propenyl)-2′,4′,5,7-tetrahydroxy-8-prenylflavanone (220), 5′-(1,1-dimethyl-2-propenyl)-4′,5,7-trihydroxy-2′-methoxy-8-prenylflavanone (221), and flemiflavanone D (240) showed excellent docking properties to EcPDF with E_dock_ < −130 kJ/mol. The geranylflavonoid 3′-*O*-methyldiplacone (205) has shown excellent antibacterial activity against Gram-positive bacteria (MIC 4–8 μg/mL), but was inactive against Gram-negative organisms, including *E. coli* [75]. Both 5′-(1,1-dimethyl-2-propenyl)-2′,4′,5,7-tetrahydroxy-8-prenylflavanone (220) and 5′-(1,1-dimethyl-2-propenyl)-4′,5,7-trihydroxy-2′-methoxy-8-prenylflavanone (221), isolated from the root extract of *Dalea scandens*, showed significant activity against both methicillin-susceptible and methicillin-resistant *S. aureus* [76]. Flemiflavanone D (240) has also shown activity against *S. aureus* [77]. The geranylflavonoid macarangaflavanone A (259), active against *E. coli* and *Micrococcus luteus* [78], showed excellent docking with SpPDF (E_dock_ = −130.7 kJ/mol). The prenylated flavanone, 5′-(1,1-dimethyl-2-propenyl)-2′,4′,5,7-tetrahydroxy-6-prenylflavanone (219), which had shown activity against oxacillin-sensitive and oxacillin-resistant *S. aureus*, docked well with PaPDF (E_dock_ = −132.2 kJ/mol).

Garcinoic acid (=*trans*-δ-tocotrienoloic acid) (550) has shown antibacterial activity against *B. cereus*, *S. aureus*, and *P. aeruginosa* [79]. This compound has also shown notable docking properties with EcPDF, PaPDF, and SaPDF, with docking energies of −134.6, −129.4, and −135.3 kJ/mol, respectively. Unfortunately, garcinoic acid (550) also docked well with human PDF (E_dock_ = −132.0 kJ/mol).

Rosmarinic acid (488), a relatively common caffeic acid ester, has been found in many plants, including *Rosmarinus officinalis*, *Melissa officinalis*, *Momordica balsamina*, *Mentha piperita*, *Salvia officinalis*, *Teucrium scorodonia*, *Sanicula europaea*, *Thymus* spp., *Hyptis verticillata*, *Lithospermum erythrorhizon*, and many other plant species [19]. The compound has a number of important biological activities such as antithrombotic, anti-inflammatory, antiviral, antifungal, and antibacterial effects [80]. Rosmarinic acid (488) showed strong docking to both EcPDF and PaPDF (−129.8 and −129.9 kJ/mol, respectively), and the compound is active against both *E. coli* [81] and *P. aeruginosa* [82,83].

The antibacterial hydroquinone derivatives shikonofuran C (494) and shikonofuran E (496) [84] both showed selective docking to EcPDF with docking energies of −130.8 kJ/mol. The naphthoquinones rhinacanthins G (506), H (507), I (508), K (510), and L (511) all showed remarkable docking to EcPDF (E_dock_ ranged from −130.3 kJ/mol to −136.0 kJ/mol). The docking energies were generally selective for EcPDF, but rhinacanthin I (508) did show comparable docking to human PDF (E_dock_ = −133.2 kJ/mol). Rhinacanthin-rich extracts have shown antibacterial activity [85].

Several stilbenoid derivatives showed particularly strong docking to bacterial PDFs. The chalcone stilbenoids cochinchinenene B (396) docked well with EcPDF (E_dock_ = −145.2 kJ/mol) and SaPDF (E_dock_ = −130.4 kJ/mol); cochinchinenene C (397) docked well with EcPDF (E_dock_ = −135.8 kJ/mol), MtPDF (E_dock_ = −133.2 kJ/mol), and SaPDF (E_dock_ = −133.7 kJ/mol); and cochinchinenene D (398) docked well with BcPDF (E_dock_ = −127.5 kJ/mol) and EcPDF (E_dock_ = −140.3 kJ/mol). However, these compounds also docked very well with human PDF (E_dock_ = −156.8, −138.2, and −245.0 kJ/mol, respectively). All three of the cochinchinenenes have shown antibacterial activity against *Helicobacter pylori* [87]. The geranylated benzofurans mulberrofuran D (408) and mulberrofuran Y (409) showed similar docking properties, docking strongly to EcPDF and PaPDF, but also to HsPDF. Both mulberrofuran D (408) and mulberrofuran Y (409) showed antibacterial activity against Gram-positive organisms, including MRSA [88]. Likewise, prenylated benzofuran eryvarin Q (402) docked strongly to EcPDF, PaPDF, SaPDF, and SpPDF, as well as HsPDF, and this compound has shown potent anti-MRSA activity [89]. ε-Viniferin (416, 417), a stilbenoidfuran derived from grape leaves, has shown anti-MRSA activity [90] and this compound docked well to EcPDF and HsPDF.

Condensed and hydrolyzable tannins showed strong docking to bacterial PDFs, but these compounds violate Lipinski’s rule of five [66], and are generally known to be non-selective protein complexing agents [91].

Gupta and Sahu have carried out molecular docking studies of 452 phytochemicals (308 antibacterial and 144 antiviral compounds) with *Leptospira interrogans* PDF using iGEMDOCK and AutoDock Vina [92]. These researchers found betulinic acid (168), carpaine, cycloartenol, ginkgolide A, glycyrrhetic acid, gossypol, nimbidin, oleanolic acid, procyanidins, quercetin (272), tomatidine (58), and ursolic acid to be strongly docking ligands. We found, using MolDock [59,57], the triterpenoid ligands betulinic acid (168), cycloartenol, glycyrrhetic acid, oleanolic acid (173), and ursolic acid to be much weaker docking ligands (E_dock_ ~ −85 to −99 kJ/mol) than polyphenolic ligands such as procyanidin B6 (386) (E_dock_ = −131.3 kJ/mol) or gossypol (E_dock_ = −128.4 kJ/mol), in apparent contradiction to the trend reported by Gupta and Sahu [92].

**Figure 24:**
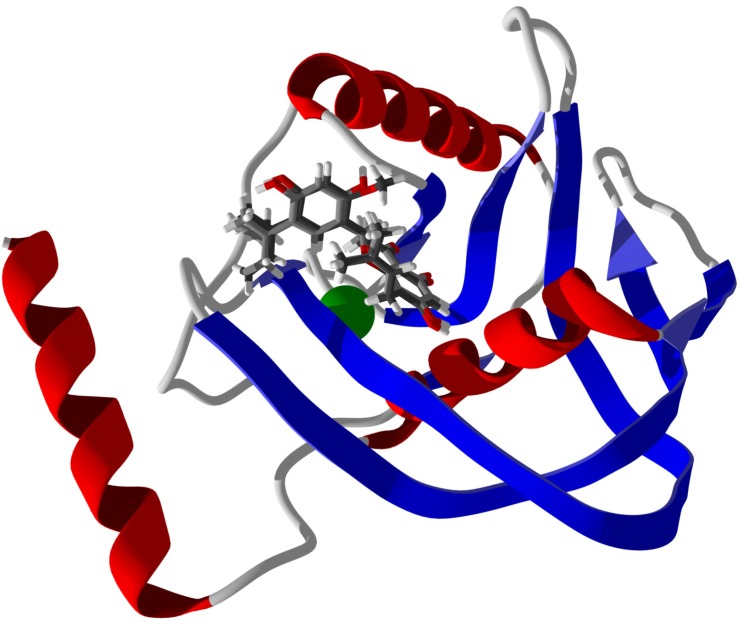
Lowest-energy docked poses for 5′-(1,1-dimethyl-2-propenyl)-2′,4′,5,7-tetrahydroxy-8-prenylflavanone (220) (grey carbon skeleton) and 5′-(1,1-dimethyl-2-propenyl)-4′,5,7-trihydroxy-2′-methoxy-8-prenylflavanone (221) (black carbon skeleton) with *Escherichia coli* peptide deformylase (EcPDF, PDB 2G2A). The Ni^2+^ cofactor in the catalytic site is shown as a green sphere.

Note that although there are several phytochemical ligands that showed strong docking to bacterial PDFs, most of these did not show selective docking to this protein target. There are two notable exceptions; the prenylated flavonoids 5′-(1,1-dimethyl-2-propenyl)-2′,4′,5,7-tetrahydroxy-8-prenylflavanone (220) and 5′-(1,1-dimethyl-2-propenyl)-4′,5,7-trihydroxy-2′-methoxy-8-prenylflavanone (221) both docked to EcPDF with docking energies (−131.3 and −132.8 kJ/mol) that were more exothermic than any other proteins examined. These two flavonoid ligands adopted very similar docking poses with EcPDF (see Figure 24).

**Figure 25:**
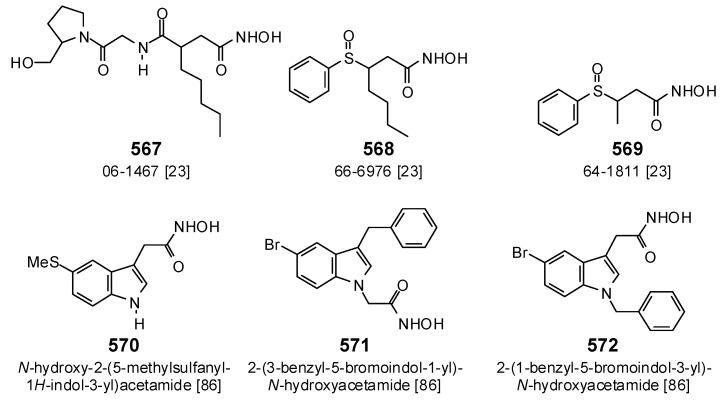
Structures of the synthetic bacterial peptide deformylase inhibitors.

For comparison, several synthetic bacterial PDF inhibitors were also investigated in this docking study. Compounds 06-1467 (567), 66-6976 (568), and 64-1811 (569) (Figure 25) have been shown to inhibit EcPDF with IC_50_ values of 0.006, 0.1, and 20 μM, respectively [23]. These compounds showed docking energies of −122.7, −101.4, and −86.3 kJ/mol, respectively, correlating with their PDF inhibitory activities. Importantly, compound 06-1467 (567) also docked well to PaPDF (E_dock_ = −129.8 kJ/mol), which compares favorably to the better docking phytochemical ligands in this study. Likewise, the synthetic indoles, *N*-hydroxy-2-(5-methylsulfanyl-1*H*-indol-3-yl)acetamide (570) (EcPDF IC_50_ > 1.0 μM), 2-(3-benzyl-5-bromoindol-1-yl)-*N*-hydroxyacetamide (571) (EcPDF IC_50_ = 0.312 μM), and 2-(1-benzyl-5-bromoindol-3-yl)-*N*-hydroxyacetamide (572) (EcPDF IC_50_ = 0.021 μM) [86], showed docking energies with EcPDF of −101.6, −117.5, and −112.6 kJ/mol, respectively (i.e., they do not correlate). However, the docking energies of these compounds with BcPDF, MtPDF, and SaPDF do correlate with EcPDF inhibition as well as with *Bacillus subtilis* antibacterial MIC values [86].

### 3.2. Bacterial Topoisomerase IV/Gyrase B

**Figure 26:**
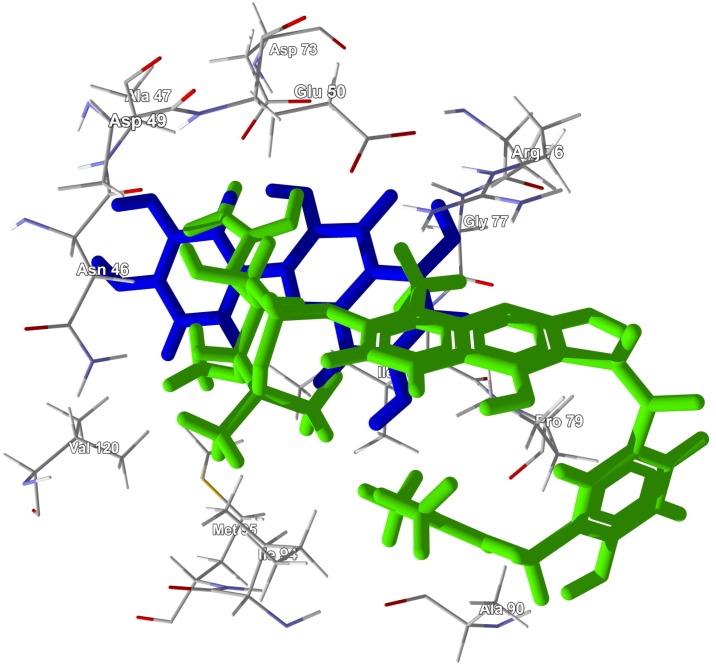
Lowest-energy docked pose of quercetin (272) (**blue**) in the novobiocin (**green**) binding site of *Escherichia coli* DNA gyrase B (PDB 1AJ6).

The MolDock docking energies for the phytochemical ligands with *E. coli* topoisomerase IV, *E. coli* gyrase B, and *M. tuberculosis* gyrase B are summarized in Table 3. The co-crystalized ligand for EcTopoIV and MtGyrB was phosphoaminophosphonic acid-adenylate ester, which crystallized in the ATP binding site of the proteins (E_dock_ ~ −176 kJ/mol). The co-crystallized ligand for EcGyrB was novobiocin (E_dock_ = −114.2 kJ/mol). (−)-Epicatechin gallate and (−)-epigallocatechin 3-gallate are known inhibitors of EcGyrB [93] and these compounds had docking energies of approximately −140 kJ/mol for EcTopoIV and MtGyrB (Table 3). There is a slight correlation between the docking energies of quercetin, epicatechin, epicatechin gallate, epigallocatechin, and epigallocatechin 3-gallate (−90.6, −87.6, −91.8, −90.8, and −94.1 kJ/mol, respectively) and the experimental dissociation constants (*K_d_*) with EcGyrB (54, 36, 34, 23, and 15 μM, respectively) [93]. Similarly, there is a correlation between the experimental IC_50_ values for quercetin (0.14 μM), norfloxacin (0.09 μM), and novobiocin (0.05 μM) [94] and the docking energies with EcGyrB (−90.6, −94.0, and −114.2 kJ/mol, respectively). Plaper and co-workers have carried out a binding study of quercetin with *E. coli* DNA gyrase [95]. These researchers found that quercetin (272) binds to EcGyrB with a *K_d_* of 15 μM. Furthermore, they carried out a molecular modeling analysis using InsightII v. 97. The final orientation of quercetin in the binding site of EcGyrB is very different from the orientation of the lowest-energy docked pose in this MolDock study (Figure 26).

6-Geranyl-5,7-dihydroxy-8(2-methylbutanoyl)-4-phenylcoumarin (345) showed strong, as well as selective, docking to the ATP sites of EcTopoIV and MtGyrB with docking energies of −154.6 and −166.5 kJ/mol, respectively. This compound has shown antibacterial activity against antibiotic resistant strains of *S. aureus* [96].

Several phytochemical ligands that showed strong docking to bacterial PDFs (see above) also docked strongly to the ATP-binding sites of EcTopoIV or MtGyrB. Angusticornin B (182), kanzonol C (193), and mulberrofuran D (408) docked well with both EcTopoIV (E_dock_ = −154.9, −151.6, and −150.0 kJ/mol, respectively) and MtGyrB (E_dock_ = −151.8, −159.8, and −151.2 kJ/mol, respectively). Likewise, piperaduncin B (197), garcinoic acid (550), and cochinchinenene B (396) docked to MtGyrB with E_dock_ = −152.4, −151.0, and −151.5 kJ/mol, respectively. Rhinacanthin H (507) and mulberrofuran Y (409) docked to EcTopoIV with docking energies of −155.9 and −153.9 kJ/mol, respectively. The Rhinacanthins showed general docking selectivity for the ATP site of either EcTopoIV or MtGyrB.

Wu and co-workers have examined the *E. coli* gyrase B inhibitory activity of several flavonoids [98]. Although none of the flavonoids were strong inhibitors, kaempferol (242) was the best with IC_50_ = 0.037 mg/mL, followed by quercetin (272) (IC_50_ = 0.076 mg/mL), chrysin (233) (IC_50_ = 0.18 mg/mL), and myricetin (261) (IC_50_ = 1.18 mg/mL). There is no correlation between these gyrase inhibitions and the docking energies to EcgyrB (E_dock_ = −90.1, −94.6, −87.1, and −99.3 kJ/mol, respectively), except that these compounds are all relatively poor docking flavonoids and are also weak EcGyrB inhibitors.

### 3.3. Protein Tyrosine Phosphatase

**Figure 27:**
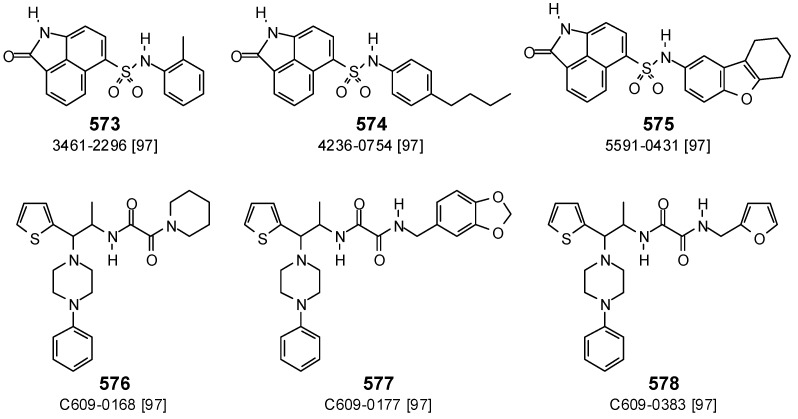
Structures of the synthetic protein tyrosine phosphatase inhibitors.

Docking scores for antibacterial phytochemical ligands with *M. tuberculosis* protein tyrosine phosphatase (Ptp) are summarized in Table 3. A number of synthetic *M. tuberculosis* Ptp inhibitors have previously been described [97]. Several of these compounds have been docked into the crystal structure of MtPtp (Figure 27, Table 3). The strongest docking of these was compound C609-0383 (578) (E_dock_ = −128.2 kJ/mol). Except for the outlier, compound 4236-0754 (574) (IC_50_ = 1.2 μM, E_dock_ = −107.7 kJ/mol), the docking scores for the ligands paralleled the experimental enzyme inhibitory data. Only two phytochemical ligands showed docking scores comparable to compound C609-0383 (578); angusticornin B (182) (E_dock_ = −127.3 kJ/mol) and garcinoic acid (550) (E_dock_ = −131.6 kJ/mol). Both of these ligands, however, are shown to be promiscuous docking compounds, strongly docking to many of the proteins investigated.

### 3.4. UDP-Galactopyranose Mutase

**Figure 28:**
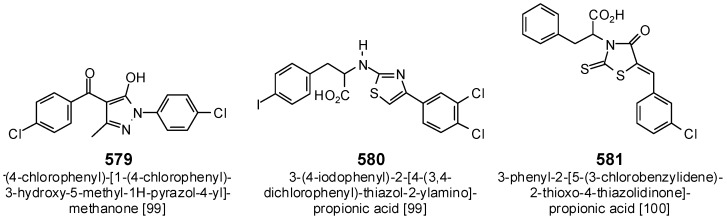
Structures of synthetic UDP-galactopyranose mutase inhibitors.

Three phenolic ligands showed strong, selective docking to *M. tuberculosis* UGM (Table 4). Drummondin D (458) and drummondin E (459) had docking energies of −134.4 and −138.3 kJ/mol, respectively, which were not as strong as the docking energy of the substrate (UDP-d-galactopyranose, E_dock_ = −162.1 kJ/mol), but were stronger than known synthetic inhibitors of MtUGM (Figure 28), (4-chlorophenyl)-[1-(4-chlorophenyl)-3-hydroxy-5-methyl-1*H*-pyrazol-4-yl]-methanone (579) (E_dock_ = −103.7 kJ/mol), 3-(4-iodophenyl)-2-[4-(3,4-dichlorophenyl)-thiazol-2-ylamino]-propionic acid (580) (E_dock_ = −112.3 kJ/mol) [99], and 3-phenyl-2-[5-(3-chlorobenzylidene)-2-thioxo-4-thiazolidinone]-propionic acid (581) (E_dock_ = −120.5 kJ/mol) [100]. Both drummondin D (458) and E (459) showed excellent antibacterial activities against *Staphylococcus aureus*, *Bacillus subtilis*, and *Mycobacterium smegmatis* [101]. Although it violates the rule-of-five for drug likeness [66], hyperbrasilol C (469) (MW = 554.67) also showed strong selective docking to MtUGM with a docking energy of −149.3 kJ/mol; this compound showed antibacterial activity against *Bacillus subtilis* [102].

### 3.5. Cytochrome P450 CYP121

**Figure 29:**
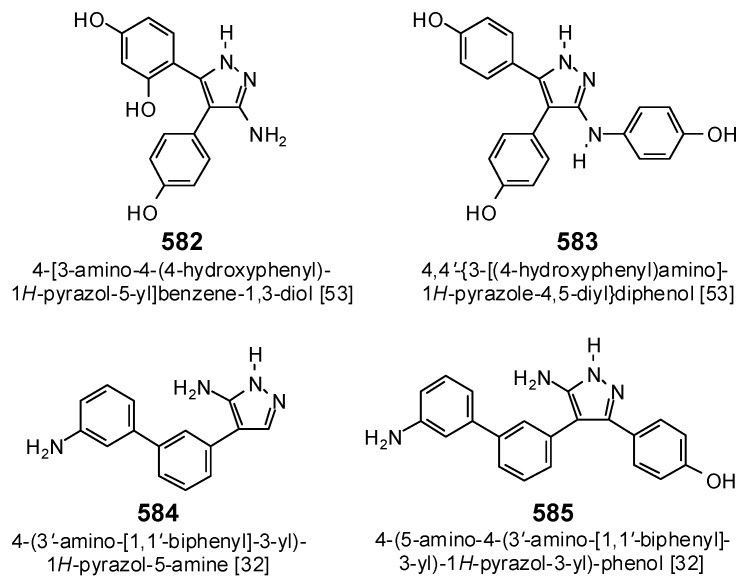
Structures of the synthetic cytochrome P450 CYP121 inhibitors.

ε-Viniferin (416, 417) showed good docking properties with MtCYP121 (E_dock_ = −134.4 and −134.6 kJ/mol, respectively), stronger than the co-crystallized ligand, 4-[5-amino-4-(3′-amino[1,1′-biphenyl]-3-yl)-1*H*-pyrazol-3-yl]phenol (585) (E_dock_ = −124.2 kJ/mol), or the synthetic MtCYP121 inhibitor, 4,4′-{3-[(4-hydroxyphenyl)-amino]-1*H*-pyrazole-4,5-diyl}diphenol (583) [53] (Figure 29, E_dock_ = −119.2 kJ/mol). However, these ε-viniferin diastereomers also docked well with EcPDF (Edock = −134.0 and −134.3 kJ/mol, respectively). 3′′′′-(2-Hydroxybenzyl)isouvarinol (218) was selective for MtCYP121 (Edock = −179.4 kJ/mol), but violates the rule-of-five (MW = 680.74, 6 hydroxyl groups).

### 3.6. NAD^+^-Dependent DNA Ligase

**Figure 30:**
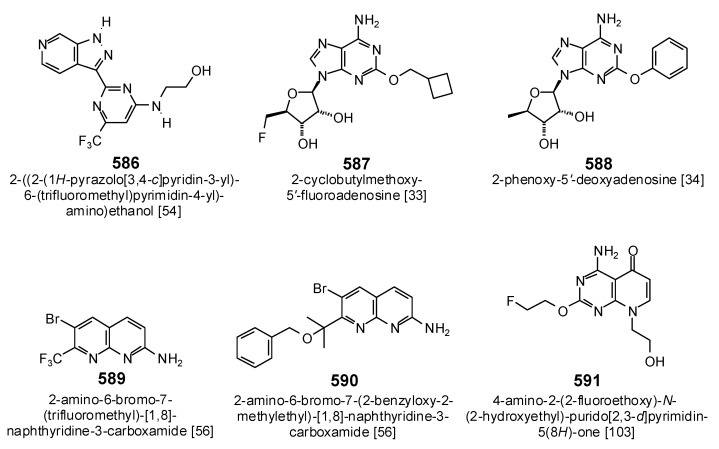

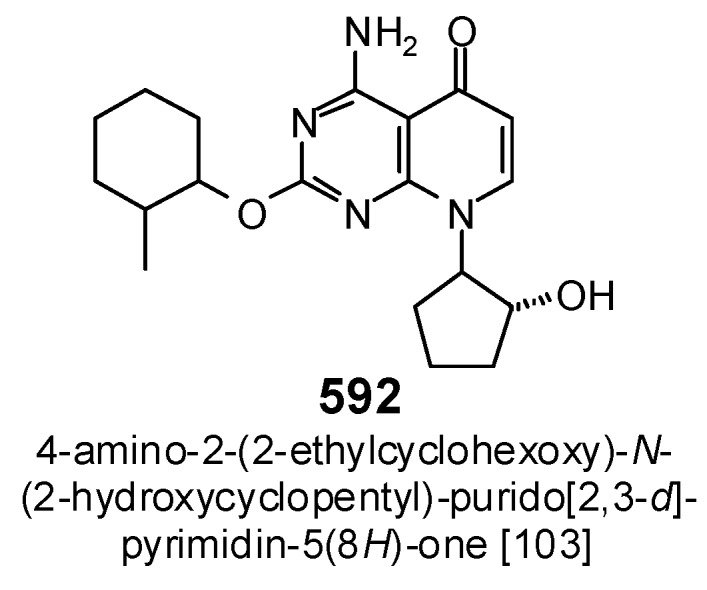
Structures of the synthetic NAD^+^-dependent DNA ligase inhibitors.

Several phytochemical ligands showed selective docking to bacterial DNA ligase (Table 4). Although they were found to be promiscuous docking ligands, the chalcones balsacone B (184) and balsacone C (185) did dock strongly to EcLigA (E_dock_ = −143.3 and −134.9 kJ/mol, respectively) and to SaLigA (E_dock_ = −143.3 and −134.9 kJ/mol, respectively). The prenylated chalcone kuraridin (194) also showed selective docking for EcLigA and SaLigA (E_dock_ = −148.8 and −150.7 kJ/mol, respectively). Piperaduncin A (196) (E_dock_ = −146.9) was selective for EcLigA, while xanthoangelol (200) selectively docked to SaLigA (E_dock_ = −153.0 kJ/mol). The prenylated neoflavonoid (4-phenylcoumarin) mesuol (334) showed docking selectivity for SaLigA (E_dock_ = −130.8 kJ/mol). The geranylated flavonoid 3′-*O*-methyldiplacone (205) docked strongly with EcLigA and SaLigA (E_dock_ = −155.1 and −143.7 kJ/mol, respectively). The prenylated flavonoids 5′-(1,1-dimethyl-2-propenyl)-2′,4′,5,7-tetrahydroxy-6-prenylflavanone (219) (E_dock_ = −136.9 kJ/mol), lonchocarpol A (255) (E_dock_ = −134.0 kJ/mol), paratocarpin L (265) (E_dock_ = −139.7 kJ/mol), and sigmoidin A (277) (E_dock_ = −135.0 kJ/mol) were selective for SaLigA. The epoxyprenylflavanoid flemiflavanone D (240) was selective for EcLigA (Edock = −139.5 kJ/mol). The prenylated xanthone garciniacowone (429) docked strongly to both EcLigA and SaLigA with docking energies of −139.0 and −146.0 kJ/mol, respectively. This compound showed excellent activity against methicillin-sensitive and methicillin-resistant *S. aureus* and moderate activity against *E. coli* [104]. Note that these phytochemical ligands had more exothermic docking energies than the co-crystallized ligands (Table 1) for the bacterial DNA ligases, and had comparable docking energies to known bacterial LigA inhibitors doxorubicin (E_dock_ = −144.2 kJ/mol with SaLigA), 2-cyclobutylmethoxy-5′-fluoroadenosine (587) (E_dock_ = −132.3 kJ/mol with SaLigA), or other synthetic LigA inhibitors [33,34,54,56,103] (Figure 30).

Prenylated flavonoids have previously shown promise as antimicrobial agents [105]. Kuraridin (194) has shown promising activity against methicillin sensitive and resistant strains of *S. aureus* [106], but was inactive against *E. coli* [107]. Piperaduncin A (196) showed antibacterial activity against *Bacillus subtilis* and *Micrococcus luteus*, but was also inactive against *E. coli* [72]. Lonchocarpol A (255) showed excellent antibacterial activity against methicillin-resistant *S. aureus* and vancomycin-resistant *Enterococcus faecium*, but was inactive against *Mycobacterium smegmatis* [108]. Paratocarpin L (syn. macarangaflavanone B) (265) has shown activity against both *E. coli* and *M. luteus* [78].

## 4. Conclusions

This docking study of 561 known antibacterial phytochemicals helps to elucidate the possible biochemical targets for these compounds and there are some notable trends. The poorest docking ligands to the bacterial protein targets in this investigation were the terpenoids, while the best docking ligands, those with large negative (exothermic) docking energies, were generally phenolics. The most susceptible protein targets, based upon docking energies, for phytochemical ligands were *E. coli* peptide deformylase (EcPDF), *E. coli* topoisomerase IV (EcTopoIV), and *E. coli* DNA ligase (EcLigA). As a class, the alkaloids showed excellent docking to EcPDF, as did the diterpenoids and miscellaneous phenolics. *S. aureus* DNA ligase (SaLigA) was a good target for chalcones, flavonoids, and especially stilbenoids, while flavonoids and isoflavonoids docked well to EcTopoIV. Prenylated chalcones and flavonoids generally showed excellent docking properties to bacterial peptide deformylases and to bacterial DNA ligases. In evaluating the ligand docking in this work, we considered the criteria of docking selectivity (promiscuous binding compounds are unlikely to be useful therapeutic agents) and whether the docking characteristics of the ligand were noticeably better than known inhibitors. In this analysis, we have also considered drug likeness. That is, we have generally overlooked those phytochemical ligands that violate Lipinski’s rule of five [66] (ligands with MW > 500 g/mol, hydrogen-bond-donating atoms > 5, hydrogen-bond-accepting atoms > 10, or ClogP > 5), even though they may have strong docking energies.

There are several limitations to in-silico docking results that should also be considered. Some of the phytochemicals examined may not be bioavailable due to limited solubility or poor bacterial cell wall permeability. In this study, we have examined the docking of the natural ligands (or their aglycones) and we did not take into account in vivo hydrolysis or other metabolic derivatization. The compounds examined have not been filtered for potential mammalian toxicity [109]. The docking studies also do not account for synergism in enzyme inhibition or antibacterial activity. The molecular docking method itself suffers from inherent limitations (e.g., the protein is modeled as a rigid structure without flexibility, solvation of the binding site and the ligand is excluded, and free-energy estimation of the protein-ligand complexes is largely ignored) [110,111]. Nevertheless, the results of this current study underscore the importance of natural products from higher plants in antibacterial drug discovery, and may provide potential avenues for the development of chemotherapeutic agents for the replacement of current antibiotic regimens or complementary management for bacterial infections.

## Figures and Tables

**Table 1:** MolDock docking energies of co-crystallized ligands and the root-mean-squared deviations between the co-crystallized ligand and the re-docked poses of the co-crystallized ligand with bacterial protein target crystal structures.

Protein Target	PDB Code	Co-Crystallized Ligand	E (kJ/mol)	RMSD (Å)
BcPDF	2OKL	Actinonin	−114.6	0.82
EcPDF	1G2A	Actinonin	−111.8	1.08
	1G27	2-[(Formyl-hydroxy-amino)-methyl]-hexanoic acid (1-dimethylcarbamoyl-2,2-dimethyl-propyl)-amide (BB-3497)	−90.2	0.49
	1LRU	Actinonin	−110.0	1.23
	2AI8	[Hydroxy(3-phenylpropyl)amino]methanol (SB-485345)	−56.1	0.71
	2KMN	Actinonin	−46.6	0.84
	3K6L	(2*S*,3*R*)-*N*4-[(1*S*)-1-(Dimethylcarbamoyl)-2,2-dimethylpropyl]-*N*1,2-dihydroxy-3-(2-methylpropyl)butanediamide (BB-2827)	−96.2	0.79
McPDF	3E3U	*N*-[(2*R*)-2-{[(2*S*)-2-(1,3-benzoxazol-2-yl)pyrrolidin-1-yl]carbonyl}-hexyl]-*N*-hydroxyformamide	−99.9	0.55
PaPDF	1LRY	Actinonin	−97.6	0.71
	1IX1	Actinonin	−127.2	0.81
	1S17	2-(3,4-Dihydro-3-oxo-2*H*-benzo[B][1,4]thiazin-2-yl)-*N*-hydroxyacetamide	−92.6	1.10
SaPDF	1Q1Y	Actinonin	−98.4	2.18
	3U7K	(*S*)-*N*-(Cyclopentylmethyl)-*N*-(2-(hydroxyamino)-2-oxoethyl)-2-[3-(2-methoxyphenyl)ureido]-3,3-dimethylbutanamide	−120.5	3.12
	3U7L	(*S*)-*N*-(Cyclopentylmethyl)-2-[3-(3,5-difluorophenyl)ureido]-*N*-(2-(hydroxyamino)-2-oxoethyl)-3,3-dimethylbutanamide	−111.9	3.25
	3U7M	*N*-[(2*R*,4*S*)-2-butyl-4-(3-(2-fluorophenyl)ureido)-5-methyl-3-oxohexyl]-*N*-hydroxyformamide	−93.9	4.94
	3U7N	*N*-[(2*R*,4*S*)-2-butyl-5-methyl-4-(3-(5-methylpyridin-2-yl)ureido)-3-oxohexyl]-*N*-hydroxyformamide	−82.5	5.00
SpPDF	2AI7	[Hydroxy(3-phenylpropyl)amino]methanol (SB-485345)	−58.8	1.07
	2AIA	2-(3-Benzoylphenoxy)ethyl(hydroxyl)formamide (SB-543668)	−113.2	5.83
	2AIE	Hydroxy[3-(6-methylpyridin-2-yl)propyl]formamide (SB-505684)	−73.1	1.06
HsPDF	4JE7	Actinonin	−105.0	0.71
	4JE8	Met-Ala-Ser	−78.3	0.85
EcTopoIV	1S16	Phosphoaminophosphonic acid-adenylate ester (ADPNP)	−175.9	0.59
EcGyrB	1AJ6	Novobiocin	−74.2	5.55
MtGyrB	3ZKB	Phosphoaminophosphonic acid-adenylate ester (ADPNP)	−158.4	1.04
	3ZKD	Phosphoaminophosphonic acid-adenylate ester (ADPNP)	−176.2	0.81
MtPtp	1U2Q	Glycerol	−29.9	4.43
	2OZ5	{(3-Chlorobenzyl)[(5-{[(3,3-diphenylpropyl)aminosulfonyl}-2-thienyl)methyl]-amino}(oxo)acetic acid	−148.3	4.92
HsPtp	1I4H	*N*-(*t*-Butoxycarbonyl)-l-tyrosyl-*N*-methyl-4-(sulfoamino)-l-phenylalaninamide	−121.2	4.81
	2I5X	(4-Ethylphenyl)sulfamic acid	−80.4	0.82
MtUGM	4RPG	UDP-d-Galactopyranose	−148.3	0.94
	4RPH	UDP-d-Galactopyranose	−162.1	0.34
	4RPJ	UDP	−132.0	0.68
	4RPK	(2*R*,5*S*)-5-[(1*R*)-1,2-Dihydroxyethyl]-3,3,4,4-tetrafluorotetrahydrofuran-2-yl[(2*R*,3*S*,4*R*,5*R*)-5-(2,4-dioxo-3,4-dihydropyrimidin-1(2*H*)-yl)-3,4-dihydroxytetrahydrofuran-2-yl]methyl dihydrogen diphosphate	−154.6	1.89
	4RPL	[(2*R*,3*S*,4*R*,5*R*)-5-(2,4-Dioxo-3,4-dihydropyrimidin-1(2*H*)-yl)-3,4-dihydroxytetrahydrofuran-2-yl]methyl(2*R*,5*S*,6*R*)-3,3,4,4-tetrafluoro-5-hydroxy-6-(hydroxymethyl)tetrahydro-2*H*-pyran-2-yl dihydrogen diphosphate	−124.9	1.67
MtCYP121	3G5H	(3*S*,6*S*)-3,6-Bis(4-hydroxybenzyl)piperazine-2,5-dioneCyclo(Tyr-Tyr)	−77.6	4.18
	4G44	3-(1*H*-1,2,4-Triazol-1-ylmethyl)aniline	−55.3	0.49
	4IPS	(3*S*,6*S*)-3,6-Bis(4-hydroxybenzyl)piperazin-2-one	−63.4	5.44
	4KTF	4,4′-(3-Amino-1*H*-pyrazole-4,5-diyl)diphenol	−80.4	8.19
	5IBE	4-[5-Amino-4-(3′-amino[1,1′-biphenyl]-3-yl)-1*H*-pyrazol-3-yl]phenol	−94.2	2.17
EcLigA	2OWO	none	-	-
	4GLX	2-Amino-6-bromo-7-(trifluoromethyl)-1,8-naphthyridine-3-carboxamide	−90.6	0.69
MtLigA	1ZAU	Adenosine monophosphate	−77.0	0.80
SaLigA	4CC5	2-Chloranyl-6-(1*H*-1,2,4-triazol-3-yl)pyrazine	−52.7	4.57
	4CC6	2-{[2-(1*H*-Pyrazolo[3,4-*c*]pyridin-3-yl)-6-(trifluoromethyl)pyridin-4-yl]amino}ethanol	−110.3	0.65
SpLigA	4GLW	7-Methoxy-6-methylpteridine-2,4-diamine	−84.0	0.41

**Table 2:** MolDock molecular docking energies (E_dock_, kJ/mol) and normalized docking scores (DS_norm_) for antibacterial phytochemical ligands with bacterial peptide deformylases.

Ligand	BcPDF		EcPDF		MtPDF		PaPDF		SaPDF		SpPDF		HsPDF	
	E_dock_	DS_norm_	E_dock_	DS_norm_	E_dock_	DS_norm_	E_dock_	DS_norm_	E_dock_	DS_norm_	E_dock_	DS_norm_	E_dock_	DS_norm_
**Indole Alkaloids**														
1-Hydroxy-6,7-dimethoxy-3-methylcarbazole (**1**)	−78.8	−89.1	−92.9	−105.0	−87.3	−98.6	−79.9	−90.3	−88.3	−99.8	−88.3	−99.8	−84.4	−95.4
11-Methoxytubotaiwine (**2**)	−83.8	−85.1	−94.6	−96.1	−96.6	−98.1	−90.9	−92.3	−94.6	−96.1	−92.3	−93.8	−100.7	−102.3
12-Methoxy-4-methyl-voachalotine (**3**)	−81.5	−78.8	−97.6	−94.4	−82.6	−79.8	−88.0	−85.0	−91.2	−88.2	−92.0	−89.0	−93.5	−90.4
3-Prenylindole (**4**)	−80.4	−101.4	−83.5	−105.3	−84.7	−106.8	−84.8	−106.9	−82.6	−104.1	−79.8	−100.7	−87.2	−110.0
Affinisine (**5**)	−74.1	−78.8	−92.8	−98.7	−88.9	−94.6	−82.6	−87.9	−83.8	−89.2	−84.6	−90.0	−86.3	−91.9
Apparicine (**6**)	−69.2	−77.5	−83.9	−94.0	−83.4	−93.4	−83.3	−93.3	−86.2	−96.6	−92.7	−103.9	−81.8	−91.6
Aristolactam I (**7**)	−90.4	−97.9	−104.7	−113.3	−98.1	−106.1	−99.5	−107.7	−105.6	−114.3	−102.3	−110.7	−100.9	−109.2
Clausenawalline A (**8**) ^a^	no dock	no dock	−128.8	−112.5	−40.1	−35.1	−120.0	−104.9	−74.0	−64.7	−102.2	−89.4	−114.5	−100.0
Cryptoheptine (**9**)	−77.6	−87.2	−96.0	−107.9	−79.8	−89.6	−91.3	−102.5	−95.5	−107.2	−98.4	−110.5	−86.8	−97.5
Diploceline (**10**)	−93.6	−93.8	−105.1	−105.3	−100.7	−100.9	−90.0	−90.2	−106.4	−106.6	−96.3	−96.5	−95.4	−95.6
Discarine B (**11**)	−109.8	−95.0	−126.2	−109.2	−122.3	−105.9	−115.8	−100.2	−126.0	−109.1	−126.7	−109.6	−126.0	−109.1
Ibogamine (**12**)	−67.3	−74.0	−91.1	−100.0	−87.8	−96.4	−90.5	−99.4	−78.9	−86.7	−75.4	−82.9	−94.6	−104.0
Iboxygaine (**13**)	−71.8	−75.0	−106.4	−111.1	−93.8	−98.0	−105.5	−110.1	−82.4	−86.0	−80.9	−84.5	−104.1	−108.7
Isovoacangine (**14**)	−90.6	−90.9	−100.7	−100.9	−89.1	−89.4	−94.5	−94.8	−83.1	−83.3	−96.9	−97.2	−89.8	−90.1
Rugosanine B (**15**)	−110.8	−93.3	−113.1	−95.3	−104.3	−87.9	−98.1	−82.7	−124.8	−105.1	−133.9	−112.8	−123.6	−104.1
Suaveolindole (**16**)	−103.0	−104.7	−108.1	−109.9	−98.5	−100.1	−108.1	−109.9	−103.2	−105.0	−103.8	−105.6	−109.0	−110.8
Toussaintine B (**17**)	−83.1	−89.3	−100.0	−107.5	−85.5	−91.9	−104.2	−112.0	−85.5	−91.9	−83.9	−90.2	−101.0	−108.6
**Isoquinoline Alkaloids**														
8-Acetonyldihydroavicine (**18**)	−99.8	−98.2	−106.8	−105.1	−96.4	−94.9	−96.6	−95.1	−98.6	−97.0	−100.0	−98.5	−104.4	−102.8
8-Acetonyldihydronitidine (**19**)	−85.7	−83.3	−108.1	−105.0	−91.5	−88.9	−104.2	−101.2	−101.3	−98.4	−102.1	−99.2	−103.8	−100.9
Antofine (**20**)	−85.5	−86.1	−107.4	−108.2	−99.1	−99.8	−93.3	−94.0	−97.5	−98.3	−91.9	−92.6	−99.7	−100.4
Berbamine (**24**)	−41.7	−35.4	−90.1	−76.5	−57.2	−48.5	−84.3	−71.5	−76.7	−65.1	−74.4	−63.2	−100.3	−85.1
Berberine (**21**)	−84.7	−87.6	−103.5	−107.0	−91.9	−95.0	−88.4	−91.4	−88.6	−91.6	−87.8	−90.7	−87.7	−90.7
Bisnorthalphenine (**22**)	−95.3	−99.8	−101.3	−106.1	−104.5	−109.5	−97.1	−101.7	−98.1	−102.8	−99.7	−104.4	−96.5	−101.0
Cepharanthine (**25**)	−86.7	−73.7	−92.5	−78.5	−74.4	−63.2	−93.4	−79.3	−114.5	−97.2	−121.5	−103.2	−96.8	−82.2
Cryptopleurine (**23**)	−80.6	−80.2	−105.6	−105.1	−98.9	−98.4	−98.5	−98.0	−96.1	−95.6	−89.7	−89.2	−99.9	−99.4
Emetine (**26**)	−111.2	−102.0	−119.4	−109.6	−117.4	−107.8	−120.8	−110.9	−120.2	−110.3	−109.9	−100.9	−110.9	−101.8
Hydrastine (**27**)	−80.2	−79.4	−112.6	−111.4	−101.6	−100.5	−94.6	−93.6	−97.9	−96.9	−102.0	−101.0	−109.5	−108.4
Isotrilobine (**29**)	−47.8	−41.3	−103.3	−89.3	−100.3	−86.6	−86.3	−74.5	−99.3	−85.7	−87.6	−75.6	−102.4	−88.4
Jatrorrhizine (**28**)	−86.4	−89.1	−101.5	−104.7	−95.1	−98.1	−92.0	−94.9	−93.2	−96.2	−92.3	−95.2	−94.7	−97.7
Lauroscholtzine (**31**)	−71.5	−73.6	−95.5	−98.2	−93.0	−95.6	−91.1	−93.7	−98.4	−101.2	−93.5	−96.2	−90.7	−93.3
Methothalistyline (**30**)	−76.6	−61.6	−112.1	−90.2	−104.5	−84.1	−115.7	−93.1	−108.1	−87.0	−127.9	−102.9	−132.7	−106.8
*N*-Demethylthalphenine (**32**)	−100.4	−103.7	−98.7	−101.9	−107.6	−111.1	−102.2	−105.6	−102.1	−105.4	−98.0	−101.2	−93.5	−96.6
Obamegine (**34**)	no dock	no dock	−83.1	−71.1	−19.4	−16.6	−67.9	−58.0	−83.2	−71.1	−83.7	−71.6	−102.0	−87.2
Oxyacanthine (**35**)	−39.7	−33.7	−93.7	−79.5	11.1	9.4	−85.3	−72.4	−69.8	−59.2	−85.8	−72.8	−94.1	−79.8
Pennsylvanine (**36**)	−102.4	−83.6	−131.0	−106.9	−113.2	−92.4	−108.7	−88.7	−126.2	−103.1	−129.9	−106.1	−137.8	−112.5
Thaliadanine (**38**)	−91.0	−73.2	−131.2	−105.5	−89.9	−72.3	−108.5	−87.2	−123.6	−99.4	−124.3	−100.0	−124.7	−100.3
Thalicarpine (**37**)	−87.8	−71.2	−128.3	−104.0	−105.2	−85.3	−105.9	−85.9	−130.1	−105.5	−115.1	−93.3	−114.6	−93.0
Thalidasine (**39**)	−64.5	−53.4	−82.5	−68.4	−67.3	−55.8	−92.8	−76.9	−82.9	−68.7	−103.5	−85.8	−102.2	−84.7
Thalistyline (**40**)	−84.1	−68.2	−119.3	−96.7	−96.0	−77.8	−111.9	−90.7	−118.2	−95.8	−117.8	−95.5	−120.6	−97.8
Thalmelatine (**41**)	−87.7	−71.6	−130.3	−106.4	−102.8	−83.9	−116.5	−95.1	−124.1	−101.3	−130.5	−106.5	−145.0	−118.4
Thalmirabine (**42**)	−76.5	−62.9	−85.1	−69.9	−39.2	−32.2	−73.6	−60.5	−111.1	−91.3	−100.2	−82.4	−97.4	−80.1
Thalphenine (**33**)	−88.5	−90.1	−102.6	−104.5	−91.2	−92.8	−94.6	−96.3	−93.8	−95.5	−91.6	−93.2	−93.0	−94.6
Thalrugosidine (**43**)	no dock	no dock	−90.7	−75.7	−42.4	−35.4	−89.4	−74.6	−97.2	−81.1	−106.9	−89.2	−96.1	−80.2
Thalrugosine (**44**)	no dock	no dock	−85.7	−72.7	−25.2	−21.4	−69.3	−58.8	−83.6	−70.9	−97.6	−82.8	−104.2	−88.4
**Piperidine, Pyrrole, Pyrrolizidine, Quinoline, and Steroidal Alkaloids**														
Aconicaramide (**46**)	−86.0	−102.0	−89.6	−106.3	−80.8	−96.0	−89.1	−105.8	−89.7	−106.5	−85.4	−101.4	−89.5	−106.2
Lasiocarpine (**47**)	−82.9	−80.2	−121.9	−117.8	−104.0	−100.5	−106.1	−102.6	−117.3	−113.4	−108.5	−104.9	−119.5	−115.6
Lasiocarpine *N*-oxide (**48**)	−94.5	−90.2	−119.1	−113.6	−110.0	−105.0	−104.5	−99.8	−106.8	−102.0	−108.2	−103.2	−117.7	−112.4
Piperine (**45**)	−88.8	−97.0	−107.3	−117.2	−89.5	−97.7	−104.9	−114.5	−95.0	−103.8	−94.8	−103.5	−104.3	−113.9
4-Methoxy-1-methyl-2(1*H*)-quinolinone (**49**)	−70.1	−87.8	−73.5	−92.0	−68.7	−86.0	−72.6	−90.9	−72.3	−90.6	−73.1	−91.5	−72.1	−90.3
Cryptolepine (**50**)	−74.4	−87.0	−85.3	−99.8	−79.1	−92.5	−82.8	−96.9	−86.8	−101.6	−88.4	−103.4	−80.9	−94.6
Neocryptolepine (**51**)	−71.2	−83.2	−83.5	−97.7	−77.5	−90.6	−79.9	−93.5	−87.6	−102.5	−87.7	−102.6	−80.4	−94.1
Pteleine (**52**)	−84.5	−99.2	−90.0	−105.8	−83.4	−98.0	−89.3	−104.9	−88.9	−104.4	−88.2	−103.6	−85.6	−100.6
Veprisinium (**53**)	−89.8	−93.0	−99.2	−102.8	−101.7	−105.4	−100.0	−103.6	−95.2	−98.6	−94.3	−97.7	−96.3	−99.8
Conessine (**54**)	−70.4	−71.4	−103.5	−104.9	−83.6	−84.8	−74.2	−75.3	−88.6	−89.8	−95.1	−96.4	−81.9	−83.0
Irehdiamine A (**55**)	−84.2	−88.9	−89.8	−94.8	−98.5	−104.0	−89.4	−94.3	−91.2	−96.2	−87.0	−91.8	−85.4	−90.0
Solacassine (**56**)	−75.6	−71.3	−92.6	−87.4	−83.1	−78.4	−86.5	−81.6	−89.6	−84.6	−92.4	−87.2	−86.7	−81.8
Solanocapsine (**57**)	−75.3	−71.7	−94.2	−89.6	−88.8	−84.6	−74.3	−70.7	−87.6	−83.4	−85.1	−81.0	−97.4	−92.7
Tomatidine (**58**)	−71.2	−68.6	−94.8	−91.4	−65.7	−63.3	−88.3	−85.1	−90.7	−87.4	−87.1	−83.9	−98.0	−94.5
**Miscellaneous Alkaloids**														
2-(Methoxyamino)-4*H*-1-benzo-pyran-3,4,5,7-tetrol (**59**)	−84.7	−97.8	−87.8	−101.4	−80.5	−93.0	−89.2	−103.0	−85.9	−99.2	−89.3	−103.1	−84.6	−97.7
Abyssenine C (**60**)	−78.7	−74.2	−102.5	−96.6	−111.3	−104.8	−106.6	−100.4	−103.1	−97.1	−101.2	−95.4	−103.7	−97.6
Amphibine H (**61**)	−64.2	−54.6	−115.2	−97.9	−123.8	−105.2	−118.6	−100.8	−119.9	−101.9	−117.1	−99.5	−121.1	−102.9
Cepharatine A (**62**)	−81.4	−86.2	−97.4	−103.1	−78.9	−83.5	−92.4	−97.8	−95.7	−101.3	−94.5	−100.1	−90.3	−95.6
Curcamide (**63**)	−93.8	−107.5	−98.8	−113.3	−93.0	−106.6	−100.5	−115.2	−97.4	−111.8	−95.5	−109.5	−102.7	−117.8
Drodrenin (**64**)	−113.2	−102.1	−148.6	−134.1	−123.6	−111.6	−135.5	−122.3	−130.6	−117.9	−135.6	−122.3	−132.2	−119.4
Eschscholtzidine (**65**)	−79.9	−82.3	−104.0	−107.2	−94.8	−97.7	−92.3	−95.2	−94.0	−96.9	−91.4	−94.3	−99.1	−102.1
Jervine (**66**)	−71.6	−68.5	−96.4	−92.1	−83.5	−79.8	−92.5	−88.4	−92.3	−88.3	−100.9	−96.4	−97.7	−93.4
Matrine (**67**)	−80.2	−91.7	−91.0	−104.1	−83.6	−95.7	−90.8	−103.9	−85.9	−98.3	−87.7	−100.3	−85.0	−97.3
Mucronine H (**68**)	−101.8	−93.6	−109.2	−100.4	−111.0	−102.0	−103.5	−95.1	−109.0	−100.2	−114.6	−105.3	−124.4	−114.3
*N*-Benzoylmescaline (**69**)	−101.3	−107.0	−109.7	−115.9	−100.9	−106.6	−112.4	−118.7	−103.4	−109.2	−99.0	−104.6	−109.9	−116.1
Nummularine B (**70**)	−107.3	−91.9	−126.2	−108.1	−127.8	−109.5	−108.7	−93.1	−129.4	−110.8	−122.9	−105.2	−119.4	−102.2
Nummularine S (**71**)	−84.2	−75.3	−120.7	−107.8	−116.1	−103.8	−112.8	−100.8	−124.0	−110.8	−119.0	−106.4	−130.6	−116.7
Scutianine B (**72**)	−90.1	−78.2	−128.5	−111.5	−103.5	−89.8	−124.6	−108.1	−118.8	−103.1	−118.3	−102.6	−126.8	−110.0
Shahidine (**73**)	−85.2	−93.7	−105.5	−116.1	−88.1	−96.9	−98.5	−108.4	−93.8	−103.1	−95.6	−105.2	−101.8	−112.0
Thaliglucinone (**74**)	−92.9	−93.4	−116.0	−116.6	−106.1	−106.7	−119.4	−120.1	−104.9	−105.5	−106.9	−107.5	−103.3	−103.8
Triisopenylguanidine (**75**)	−91.4	−101.7	−107.6	−119.8	−95.6	−106.4	−98.4	−109.6	−97.2	−108.2	−92.1	−102.6	−101.4	−112.9
Tuberine (**76**)	−92.7	−85.7	−136.7	−126.5	−103.7	−95.9	−105.6	−97.7	−121.9	−112.7	−121.1	−112.0	−121.7	−112.6
**Monoterpenoids**														
Linalool (**77**)	−78.7	−105.6	−81.4	−109.1	−75.9	−101.7	−80.0	−107.3	−77.0	−103.2	−77.9	−104.4	−80.4	−107.8
Thymol (**78**)	−68.6	−92.7	−71.0	−96.1	−65.8	−89.0	−67.5	−91.3	−69.5	−94.1	−69.0	−93.3	−68.6	−92.8
Thymoquinol (**79**)	−73.7	−96.4	−76.2	−99.6	−70.6	−92.4	−75.7	−98.9	−76.9	−100.5	−75.7	−99.0	−73.8	−96.5
β-Dolabrin (**80**)	−76.9	−101.3	−76.9	−101.4	−74.2	−97.8	−77.3	−102.0	−77.9	−102.7	−77.7	−102.4	−75.0	−98.9
β-Thujaplicin (**81**)	−74.9	−98.4	−75.8	−99.5	−73.4	−96.3	−75.6	−99.3	−76.2	−100.1	−76.9	−100.9	−74.7	−98.1
**Sesquiterpenoids**														
11,13-Dehydroeriolin (**82**)	−85.2	−95.4	−100.0	−112.0	−89.1	−99.8	−101.1	−113.2	−93.0	−104.2	−86.9	−97.4	−84.6	−94.8
2,10-Bisaboladien-1-one (**83**)	−84.5	−100.6	−91.0	−108.3	−94.6	−112.6	−86.0	−102.3	−93.0	−110.7	−89.4	−106.4	−86.2	−102.7
2-Hydroxycalamenene (**84**)	−81.1	−96.8	−85.0	−101.5	−81.5	−97.3	−84.5	−100.9	−83.6	−99.8	−82.8	−98.9	−78.0	−93.1
2-Methoxyfurano-9-guaien-8-one (**85**)	−91.7	−103.3	−100.3	−112.9	−93.2	−104.9	−102.4	−115.3	−102.9	−115.8	−101.3	−114.1	−95.4	−107.5
4α,10α-Dihydroxy-1,11(13)-guaiadien-12,8-olide (**93**)	−89.8	−100.6	−93.3	−104.5	−87.2	−97.7	−97.1	−108.7	−91.3	−102.3	−90.0	−100.8	−96.6	−108.2
4α,10β-Dihydroxy-1,11(13)-guaiadien-12,8-olide (**89**)	−94.0	−105.3	−99.8	−111.8	−104.1	−116.6	−98.7	−110.6	−102.0	−114.3	−93.3	−104.6	−96.7	−108.3
Alantolactone (**86**)	−78.9	−92.2	−80.7	−94.4	−77.3	−90.4	−82.9	−97.0	−86.2	−100.8	−83.2	−97.3	−78.2	−91.4
Alliacol A (**87**)	−45.9	−51.5	−75.8	−85.0	−67.2	−75.3	−77.4	−86.7	−73.1	−81.9	−72.7	−81.4	−77.0	−86.3
Alliacol B (**88**)	−78.5	−87.9	−82.2	−92.0	−90.5	−101.4	−81.8	−91.6	−85.3	−95.5	−80.3	−89.9	−81.7	−91.6
Artemisinic acid (**113**)	−84.4	−98.4	−85.0	−99.2	−86.2	−100.5	−83.8	−97.7	−88.3	−103.0	−86.0	−100.3	−81.4	−94.9
Baileyolin (**90**)	−98.8	−99.6	−103.1	−104.0	−111.4	−112.3	−105.2	−106.1	−113.3	−114.3	−108.2	−109.1	−103.2	−104.0
Bilobalide A (**91**)	−78.0	−81.4	−93.0	−97.1	−95.6	−99.9	−82.5	−86.2	−98.6	−103.0	−102.0	−106.5	−95.8	−100.0
Confertin (**92**)	−83.4	−95.4	−90.9	−104.0	−91.4	−104.6	−90.5	−103.5	−89.2	−102.0	−87.4	−100.0	−87.5	−100.1
Cyperenal (**94**)	−46.2	−55.2	−65.6	−78.3	−70.6	−84.3	−60.6	−72.4	−63.2	−75.4	−67.6	−80.7	−60.9	−72.7
Cyperenol (**95**)	−36.1	−43.0	−65.5	−77.9	−70.6	−84.0	−57.8	−68.8	−62.6	−74.5	−64.7	−77.1	−57.1	−68.0
Furanodienone (**97**)	−84.3	−98.9	−88.4	−103.7	−87.1	−102.1	−94.7	−111.1	−89.0	−104.4	−88.5	−103.8	−87.5	−102.6
Ganodermycin (**96**)	−111.8	−122.8	−109.5	−120.3	−115.7	−127.1	−109.3	−120.1	−113.6	−124.8	−110.9	−121.8	−105.4	−115.7
Helenalin (**98**)	−69.8	−78.4	−81.9	−92.0	−82.8	−93.0	−80.0	−89.8	−84.2	−94.6	−85.8	−96.4	−86.5	−97.2
Hydrogrammic acid (**99**)	−83.0	−93.5	−91.4	−103.0	−86.8	−97.7	−88.5	−99.6	−88.9	−100.1	−87.8	−98.8	−83.7	−94.3
Isoalantolactone (**100**)	−79.3	−92.8	−84.7	−99.0	−76.8	−89.8	−87.9	−102.9	−85.1	−99.5	−85.4	−99.9	−78.0	−91.2
Ivaxillin (**101**)	−81.1	−90.6	−98.4	−110.0	−90.3	−100.9	−100.6	−112.4	−93.6	−104.6	−90.4	−101.0	−82.8	−92.6
Petrovin A (**102**)	−84.1	−96.2	−88.9	−101.7	−84.5	−96.6	−90.1	−103.0	−88.5	−101.2	−86.0	−98.3	−86.2	−98.7
Petrovin B (**103**)	−84.8	−96.8	−87.5	−99.9	−80.5	−91.9	−89.9	−102.6	−86.6	−98.8	−84.7	−96.6	−85.9	−98.0
Polygodial (**104**)	−79.0	−92.1	−84.1	−98.1	−88.1	−102.7	−82.8	−96.5	−88.8	−103.6	−86.1	−100.4	−74.8	−87.2
Rishitin (**105**)	−75.0	−89.0	−80.0	−94.9	−77.0	−91.4	−81.6	−96.8	−79.3	−94.1	−80.8	−95.9	−81.0	−96.1
Xanthorrhizol (**106**)	−86.1	−102.8	−90.9	−108.5	−91.2	−108.9	−90.1	−107.5	−92.3	−110.3	−88.6	−105.8	−91.7	−109.4
α-Amorphene (**107**)	−75.5	−92.2	−79.5	−97.1	−80.2	−97.9	−77.9	−95.1	−78.1	−95.3	−73.8	−90.1	−72.0	−87.9
α-Cadinene (**108**)	−73.3	−89.5	−80.6	−98.4	−79.8	−97.4	−80.4	−98.1	−79.6	−97.2	−77.6	−94.8	−77.8	−94.9
α-Copaene (**110**)	−66.9	−83.6	−65.6	−81.9	−67.3	−84.1	−66.7	−83.3	−73.2	−91.5	−68.6	−85.7	−68.0	−85.0
α-Muurolene (**109**)	−76.9	−93.9	−79.1	−96.6	−75.9	−92.7	−78.2	−95.4	−78.7	−96.1	−77.3	−94.3	−79.0	−96.4
γ-Cadinene (**112**)	−77.2	−94.2	−81.4	−99.4	−77.5	−94.6	−81.9	−99.9	−81.4	−99.4	−79.7	−97.3	−79.7	−97.3
**Diterpenoids**														
1,12-Diacetyljativatriol (**114**)	−96.9	−94.2	−107.0	−104.0	−107.1	−104.1	−98.6	−95.9	−101.6	−98.8	−102.1	−99.3	−102.4	−99.5
12-Oxo-3,13(16)-clerodadien-15-oic acid (**115**)	−104.5	−110.0	−102.9	−108.3	−98.3	−103.5	−101.8	−107.1	−109.2	−114.9	−96.9	−102.0	−100.8	−106.1
12-Oxo-8,13(16)-clerodadien-15-oic acid (**116**)	−93.4	−98.3	−106.7	−112.3	−102.3	−107.8	−99.8	−105.1	−106.6	−112.3	−107.9	−113.6	−95.3	−100.3
13-Epimanoyl oxide (**117**)	−58.8	−63.8	−81.6	−88.6	−86.1	−93.5	−81.0	−88.0	−78.6	−85.3	−78.8	−85.6	−76.7	−83.3
13-Episclareol (**118**)	−88.1	−93.8	−101.5	−108.0	−103.0	−109.6	−99.6	−106.0	−99.1	−105.5	−94.6	−100.7	−95.7	−101.8
3,4-Seco-4(18)-trachyloben-3-oic acid (**120**)	−99.3	−106.4	−99.0	−106.1	−99.6	−106.7	−96.3	−103.2	−99.3	−106.4	−96.5	−103.3	−94.9	−101.6
3-Hydroxytotarol (**119**)	−82.6	−88.5	−87.0	−93.2	−83.0	−88.9	−89.1	−95.4	−86.2	−92.3	−81.7	−87.5	−84.8	−90.8
7,13-Labdadien-15-ol acetate (**121**)	−76.8	−79.7	−108.6	−112.7	−105.3	−109.3	−106.9	−110.9	−105.1	−109.0	−102.0	−105.8	−104.1	−108.1
7,13-Labdadien-15-ol malonate (**122**)	−99.8	−99.4	−121.2	−120.6	−111.4	−110.9	−115.9	−115.4	−115.9	−115.4	−122.7	−122.2	−105.3	−104.8
Acetylcrinipellin A (**125**)	−90.1	−90.1	−100.1	−100.0	−96.4	−96.4	−91.8	−91.7	−97.7	−97.7	−88.5	−88.4	−99.4	−99.4
Aethiopinone (**123**)	−89.4	−96.4	−104.4	−112.6	−92.8	−100.1	−105.6	−113.9	−92.5	−99.7	−94.5	−102.0	−100.0	−107.8
Andrographolide (**124**)	−89.7	−91.5	−102.9	−104.9	−101.3	−103.3	−102.6	−104.6	−106.9	−109.0	−104.6	−106.6	−99.9	−101.9
Biflorin (**126**)	−92.0	−99.4	−102.1	−110.4	−93.6	−101.1	−101.0	−109.2	−102.0	−110.2	−100.3	−108.4	−95.5	−103.3
Continentalic acid (**127**)	−74.3	−79.6	−91.4	−97.9	−76.7	−82.2	−80.7	−86.4	−82.5	−88.4	−81.9	−87.7	−78.7	−84.3
Crinipellin A (**129**)	−79.7	−82.9	−96.3	−100.1	−99.9	−103.9	−82.0	−85.3	−92.6	−96.3	−84.8	−88.2	−94.9	−98.7
Cryptobeilic acid A (**128**)	−89.8	−94.8	−116.8	−123.2	−106.8	−112.7	−112.3	−118.5	−103.4	−109.1	−102.9	−108.6	−108.4	−114.4
Cryptobeilic acid C (**130**)	−95.2	−95.8	−124.6	−125.5	−105.0	−105.7	−122.0	−122.8	−116.1	−116.9	−125.2	−126.0	−113.5	−114.2
Cryptobeilic acid D (**131**)	−92.2	−98.9	−103.5	−111.1	−100.5	−107.8	−96.4	−103.5	−98.0	−105.2	−100.5	−107.9	−98.6	−105.8
Effusanin A (**132**)	−65.1	−66.5	−83.0	−84.8	−87.7	−89.6	−77.7	−79.4	−89.6	−91.6	−74.6	−76.2	−87.4	−89.3
Effusanin B (**133**)	−80.7	−79.4	−94.3	−92.8	−99.9	−98.3	−91.1	−89.7	−100.9	−99.2	−95.0	−93.5	−89.5	−88.0
Effusanin C (**134**)	−71.5	−69.4	−86.0	−83.5	−91.7	−89.0	−82.0	−79.6	−95.4	−92.6	−91.1	−88.4	−95.0	−92.2
Effusanin D (**135**)	−74.6	−70.0	−101.8	−95.6	−100.7	−94.6	−99.8	−93.8	−105.0	−98.6	−101.4	−95.2	−96.9	−91.0
Effusanin E (**136**)	−73.5	−74.0	−86.6	−87.2	−87.2	−87.8	−79.1	−79.6	−92.2	−92.8	−85.4	−85.9	−89.6	−90.2
Grandiflorenic acid (**137**)	−65.3	−70.1	−81.1	−87.1	−75.8	−81.4	−59.4	−63.8	−70.6	−75.8	−74.9	−80.4	−70.7	−75.9
Haplociliatic acid (**138**)	−92.3	−95.4	−104.2	−107.8	−102.0	−105.4	−95.0	−98.2	−103.9	−107.4	−109.6	−113.3	−100.6	−103.9
Hypargenin A (**139**)	−83.8	−87.1	−95.7	−99.6	−91.5	−95.2	−89.4	−93.0	−97.2	−101.1	−94.1	−97.9	−88.8	−92.4
Hypargenin B (**140**)	−81.8	−86.3	−90.3	−95.3	−81.9	−86.4	−84.3	−88.9	−94.4	−99.6	−87.4	−92.2	−83.5	−88.1
Hypargenin D (**141**)	−72.3	−77.8	−89.2	−96.0	−82.0	−88.2	−79.5	−85.6	−85.3	−91.8	−82.5	−88.7	−82.5	−88.8
Hypargenin F (**142**)	−71.0	−73.9	−86.1	−89.5	−72.4	−75.3	−79.9	−83.1	−79.8	−83.0	−83.3	−86.6	−73.2	−76.1
Isodomedin (**143**)	−66.4	−65.2	−94.5	−92.8	−95.8	−94.1	−78.1	−76.7	−98.8	−97.1	−98.2	−96.4	−76.7	−75.3
Kamebanin (**144**)	−72.7	−75.3	−95.5	−98.9	−81.2	−84.1	−74.3	−77.0	−101.5	−105.2	−79.3	−82.2	−74.2	−76.9
Lasiokaurin (**145**)	−82.0	−79.5	−101.0	−97.9	−100.5	−97.4	−84.3	−81.7	−99.4	−96.3	−91.4	−88.6	−70.6	−68.4
Longikaurin A (**146**)	−67.8	−69.3	−79.4	−81.1	−90.4	−92.3	−76.1	−77.7	−89.0	−91.0	−81.6	−83.3	−70.7	−72.2
Longikaurin B (**147**)	−63.0	−61.1	−83.9	−81.5	−93.2	−90.4	−78.1	−75.8	−94.0	−91.3	−89.0	−86.4	−69.9	−67.8
Longikaurin C (**148**)	−27.9	−27.4	−80.2	−78.9	−88.1	−86.7	−79.2	−78.0	−91.0	−89.5	−90.2	−88.7	−89.2	−87.7
Longikaurin D (**149**)	−51.3	−49.8	−84.3	−81.8	−96.0	−93.2	−84.9	−82.4	−95.8	−93.0	−91.7	−89.0	−96.8	−94.0
Longikaurin E (**150**)	−75.8	−77.4	−84.0	−85.8	−85.9	−87.8	−82.1	−83.9	−88.9	−90.9	−79.3	−81.0	−84.4	−86.3
Longikaurin F (**151**)	−88.2	−82.9	−98.6	−92.7	−111.3	−104.6	−98.8	−92.8	−114.1	−107.1	−102.9	−96.7	−107.7	−101.1
Longikaurin G (**152**)	−74.6	−75.1	−81.2	−81.7	−96.0	−96.6	−80.8	−81.3	−91.5	−92.1	−87.1	−87.6	−75.8	−76.3
Lupulin E (**153**)	−97.3	−86.8	−117.1	−104.5	−112.5	−100.4	−108.4	−96.7	−99.1	−88.5	−102.7	−91.7	−119.9	−107.0
Lupulin F (**154**)	−87.2	−77.8	−116.1	−103.5	−115.2	−102.7	−99.8	−88.9	−103.0	−91.8	−101.8	−90.7	−116.3	−103.7
Methyl seconidoresedate (**155**)	−89.3	−93.0	−106.5	−110.9	−97.2	−101.3	−109.3	−113.9	−97.5	−101.6	−101.4	−105.7	−99.7	−103.9
Pisiferol (**156**)	−70.5	−75.5	−86.2	−92.4	−86.5	−92.6	−75.9	−81.3	−93.3	−100.0	−88.0	−94.2	−82.1	−87.9
Salvic acid (**157**)	−85.1	−89.2	−101.6	−106.5	−89.6	−93.9	−105.0	−110.1	−97.3	−102.0	−95.9	−100.5	−97.3	−102.0
Salvic acid acetate (**158**)	−92.3	−92.9	−114.4	−115.1	−91.3	−91.8	−104.1	−104.7	−103.3	−104.0	−102.8	−103.5	−103.8	−104.5
Shikokianin (**159**)	−63.1	−59.2	−104.1	−97.8	−93.7	−88.0	−93.9	−88.2	−93.0	−87.3	−97.0	−91.1	−84.8	−79.7
Strictic acid (**160**)	−88.5	−93.6	−103.1	−109.0	−94.5	−99.9	−106.8	−112.9	−97.2	−102.8	−98.6	−104.3	−96.5	−102.0
Taxodione (**161**)	−69.8	−73.8	−92.1	−97.4	−87.3	−92.3	−76.9	−81.3	−92.9	−98.2	−90.8	−96.0	−74.2	−78.4
Trichodonin (**162**)	−80.6	−78.3	−91.6	−89.1	−67.4	−65.5	−82.0	−79.7	−80.1	−77.9	−91.9	−89.3	−84.1	−81.7
Umbrosin A (**163**)	−74.7	−77.4	−93.3	−96.6	−83.8	−86.8	−78.7	−81.5	−95.2	−98.7	−85.8	−88.8	−76.2	−78.9
Umbrosin B (**164**)	−76.3	−79.2	−88.8	−92.2	−86.7	−90.0	−78.3	−81.2	−86.6	−89.9	−86.6	−89.9	−73.4	−76.2
Yuexiandajisu A (**165**)	−104.3	−109.8	−114.0	−120.0	−101.1	−106.5	−113.3	−119.3	−109.8	−115.6	−98.4	−103.6	−106.3	−111.9
**Triterpenoids**														
Alisol A 24-acetate (**166**)	−88.7	−78.6	−96.5	−85.6	−104.7	−92.9	−90.7	−80.5	−105.4	−93.5	−100.4	−89.1	−103.2	−91.6
Alisol B 23-acetate (**167**)	−63.6	−57.0	−112.6	−101.0	−107.2	−96.2	−81.1	−72.8	−111.0	−99.6	−98.1	−88.0	−113.2	−101.6
Betulinic acid (**168**)	−98.3	−91.8	−91.4	−85.3	−113.0	−105.5	−88.8	−82.9	−105.7	−98.7	−94.3	−88.0	−102.3	−95.5
Entagenic acid (**169**)	−56.4	−51.5	−101.0	−92.2	−86.3	−78.8	−69.3	−63.3	−85.1	−77.7	−79.1	−72.2	−87.7	−80.0
Lantic acid (**170**)	−38.1	−35.2	−79.1	−73.1	−82.2	−76.0	−74.4	−68.8	−80.1	−74.0	−83.1	−76.8	−88.8	−82.1
Mahmoodin (**171**)	−81.3	−72.4	−97.6	−86.9	−84.8	−75.5	−87.8	−78.1	−86.6	−77.1	−76.8	−68.3	−99.8	−88.9
Maslinic acid (**172**)	−43.6	−40.3	−98.9	−91.3	−87.0	−80.3	−80.1	−73.9	−84.5	−78.0	−82.6	−76.3	−92.1	−85.0
Oleanolic acid (**173**)	−70.3	−65.7	−95.2	−88.9	−81.8	−76.4	−77.9	−72.7	−86.4	−80.7	−82.8	−77.3	−88.4	−82.6
Pristimerin (**174**)	−81.9	−76.0	−103.5	−96.1	−98.1	−91.0	−89.9	−83.5	−96.0	−89.1	−88.3	−81.9	−102.8	−95.5
Rubrinol (**175**)	−70.8	−66.8	−88.0	−83.0	−82.0	−77.4	−79.6	−75.1	−87.1	−82.2	−86.6	−81.7	−95.0	−89.6
Tingenone (**176**)	−71.6	−68.7	−92.0	−88.2	−83.9	−80.5	−80.6	−77.3	−83.2	−79.8	−87.3	−83.8	−94.1	−90.3
**Chalcones**														
1-(2,6-Dihydroxy-4-methoxyphenyl)-3-phenyl-1-propanone (**177**)	−92.7	−102.8	−107.4	−119.1	−93.8	−104.1	−101.8	−112.9	−100.6	−111.6	−98.9	−109.7	−105.3	−116.8
2′-Hydroxy-2,3,4′,6′-tetramethoxychalcone (**178**)	−91.9	−94.2	−110.7	−113.6	−92.8	−95.2	−108.6	−111.4	−103.6	−106.3	−99.7	−102.2	−117.5	−120.5
3′′′′,5′′′,5′′′′′-Tribenzyl-2′′′′,2′′′′′,2′′′′′′-trihydroxyisodiuvaretin (**180**)	−41.6	−32.2	−114.0	−88.2	−141.1	−109.1	−129.7	−100.3	−147.7	−114.2	−126.3	−97.7	−145.6	−112.6
4′-Hydroxychalcone (**179**)	−78.9	−93.3	−94.2	−111.5	−81.1	−96.0	−86.5	−102.3	−90.3	−106.9	−87.3	−103.4	−89.4	−105.9
5″,5′′′′,5′′′′′-Tribenzyl-2′′′′,2′′′′′,2′′′′′′-trihydroxyisodiuvaretin (**181**)	−100.9	−78.0	−114.7	−88.7	−152.4	−117.9	−164.0	−126.9	−145.6	−112.6	−136.0	−105.2	−156.9	−121.4
Angusticornin B (**182**)	−117.5	−112.4	−143.5	−137.2	−134.4	−128.6	−134.7	−128.8	−129.3	−123.7	−131.4	−125.7	−126.7	−121.2
Balsacone A (**183**)	−106.9	−102.6	−127.5	−122.4	−122.3	−117.4	−122.4	−117.5	−127.6	−122.4	−121.9	−117.0	−124.1	−119.1
Balsacone B (**184**)	−109.2	−104.8	−129.0	−123.8	−123.0	−118.0	−124.7	−119.7	−128.3	−123.2	−132.4	−127.0	−119.9	−115.1
Balsacone C (**185**)	−109.0	−107.3	−124.2	−122.1	−121.4	−119.5	−120.0	−118.1	−127.4	−125.3	−132.3	−130.1	−122.4	−120.4
Bartericin C (**186**)	−75.7	−73.3	−123.3	−119.4	−109.6	−106.2	−107.7	−104.3	−102.2	−99.0	−105.6	−102.3	−112.7	−109.2
Bavachalcone (**187**)	−105.6	−109.0	−121.5	−125.3	−114.0	−117.6	−120.0	−123.9	−116.0	−119.7	−112.3	−115.9	−116.9	−120.6
Broussochalcone B (**188**)	−102.3	−107.1	−117.5	−122.9	−109.7	−114.8	−119.4	−124.9	−103.2	−108.0	−109.7	−114.8	−114.5	−119.8
Corylifol B (**189**)	−106.2	−109.3	−121.0	−124.6	−112.9	−116.2	−123.3	−127.0	−113.7	−117.1	−117.7	−121.2	−119.7	−123.3
Erythbidin C (**190**)	−98.5	−104.4	−119.5	−126.6	−103.3	−109.5	−115.5	−122.4	−108.7	−115.1	−113.0	−119.7	−112.2	−118.9
Helichrysone A (**191**)	−91.6	−93.1	−117.8	−119.7	−99.4	−101.0	−112.4	−114.2	−109.2	−110.9	−102.7	−104.3	−104.9	−106.6
Isobavachalcone (**192**)	−104.3	−109.2	−117.8	−123.2	−111.5	−116.7	−114.0	−119.3	−112.4	−117.7	−113.6	−118.9	−115.8	−121.2
Kanzonol C (**193**)	−110.3	−108.4	−133.3	−130.9	−125.2	−123.0	−128.4	−126.1	−130.1	−127.8	−135.9	−133.4	−131.8	−129.4
Kuraridin (**194**)	−117.2	−110.9	−124.0	−117.3	−105.7	−100.0	−127.9	−121.0	−121.1	−114.6	−111.5	−105.6	−136.6	−129.2
Myrigalone G (**195**)	−89.4	−97.5	−105.7	−115.3	−96.9	−105.7	−105.5	−115.0	−104.4	−113.9	−99.7	−108.8	−104.9	−114.5
Piperaduncin A (**196**)	−113.3	−103.3	−141.0	−128.5	−121.0	−110.3	−130.8	−119.2	−132.2	−120.5	−120.8	−110.1	−131.7	−120.1
Piperaduncin B (**197**)	−117.9	−106.3	−139.3	−125.6	−112.0	−101.0	−141.8	−127.9	−144.3	−130.2	−134.3	−121.2	−133.6	−120.5
Piperaduncin C (**198**)	−110.2	−96.3	−155.9	−136.2	−104.1	−91.0	−149.2	−130.4	−127.2	−111.2	−131.8	−115.2	−149.4	−130.6
Psorachalcone A (**199**)	−102.2	−105.2	−118.9	−122.4	−107.2	−110.4	−116.8	−120.3	−112.0	−115.3	−114.5	−117.9	−116.7	−120.2
Xanthoangelol (**200**)	−117.6	−115.4	−135.2	−132.8	−131.9	−129.5	−131.3	−129.0	−132.1	−129.7	−130.6	−128.3	−132.6	−130.2
Xanthoangelol F (**201**)	−115.9	−112.5	−129.8	−126.0	−135.6	−131.6	−127.0	−123.3	−126.2	−122.5	−126.5	−122.8	−127.5	−123.7
**Flavonoids**														
2′,5,5′,7-Tetrahydroxyflavanone (**202**)	−81.2	−88.4	−95.9	−104.4	−84.1	−91.5	−95.4	−103.9	−87.4	−95.2	−87.7	−95.5	−88.2	−96.0
2′,7-Dimethoxyflavone (**203**)	−84.2	−92.3	−94.7	−103.8	−87.5	−95.9	−95.8	−105.0	−98.8	−108.3	−95.9	−105.1	−94.8	−103.8
3′′′′-(2-Hydroxybenzyl)-isouvarinol (**218**)	−77.8	−63.6	−130.5	−106.6	−109.5	−89.5	−133.1	−108.8	−123.5	−100.9	−147.9	−120.9	−149.8	−122.4
3′′′′-(2-Hydroxybenzyl)uvarinol (**217**)	−81.3	−66.5	−129.1	−105.5	−135.5	−110.7	−141.3	−115.5	−117.5	−96.0	−131.1	−107.2	−153.7	−125.6
3′-Methylpelargonidin (**204**)	−90.7	−99.1	−98.9	−108.0	−87.4	−95.5	−101.5	−110.8	−92.0	−100.5	−98.0	−107.1	−92.4	−100.9
3′-*O*-Methyldiplacone (**205**)	−67.4	−63.8	−133.5	−126.4	−120.3	−113.9	−122.7	−116.1	−111.4	−105.5	−109.3	−103.4	−128.9	−122.0
4′,5,7-Trihydroxy-6-methyl-8-prenylflavanone (**207**)	−87.9	−89.3	−102.2	−103.8	−107.1	−108.8	−99.8	−101.4	−98.9	−100.5	−94.2	−95.7	−105.2	−106.9
4′,5,7-Trihydroxy-8-methyl-6-prenylflavanone (**206**)	−84.8	−86.2	−106.0	−107.7	−94.0	−95.5	−105.6	−107.3	−96.9	−98.5	−88.8	−90.2	−105.6	−107.3
4′,5-Dihydroxy-7-methoxy-6-prenylflavanone (**208**)	−92.3	−93.8	−111.3	−113.1	−96.5	−98.0	−112.1	−113.9	−96.6	−98.2	−91.5	−93.0	−106.4	−108.1
4′,6,7-Trihydroxy-3′,5′-dimethoxyflavone (**209**)	−90.8	−94.5	−109.8	−114.2	−99.9	−103.9	−104.2	−108.4	−97.9	−101.9	−97.9	−101.8	−102.6	−106.7
4′,7-Dihydroxy-8-methylflavan (**210**)	−80.2	−90.7	−87.3	−98.8	−80.9	−91.5	−87.8	−99.4	−81.8	−92.6	−85.8	−97.1	−86.7	−98.1
4′-Hydroxy-5,7-dimethoxy-flavone (**211**)	−83.6	−90.0	−98.6	−106.1	−96.0	−103.3	−91.5	−98.4	−88.5	−95.3	−84.9	−91.4	−91.7	−98.7
5″-(2-Hydroxybenzyl)-isouvarinol (**216**)	−113.5	−92.8	−143.5	−117.3	−132.9	−108.6	−137.9	−112.7	−153.3	−125.3	−134.9	−110.2	−156.6	−128.0
5′-(1,1-Dimethyl-2-propenyl)-2′,4′,5,7-tetrahydroxy-6-prenyl-flavanone (**219**)	−102.7	−98.3	−122.9	−117.6	−101.9	−97.5	−132.2	−126.5	−111.6	−106.8	−99.5	−95.2	−117.4	−112.3
5′-(1,1-Dimethyl-2-propenyl)-2′,4′,5,7-tetrahydroxy-8-prenylflavanone (**220**)	−106.9	−102.3	−131.3	−125.6	−112.7	−107.8	−128.4	−122.8	−119.4	−114.2	−115.9	−110.8	−124.1	−118.7
5′-(1,1-Dimethyl-2-propenyl)-4′,5,7-trihydroxy-2′-methoxy-8-prenylflavanone (**221**)	−103.2	−97.6	−132.8	−125.7	−122.3	−115.7	−128.4	−121.6	−111.4	−105.4	−106.7	−101.0	−124.5	−117.8
5,6-Dihydroxy-4′,7,8-trimethoxy-flavone (**212**)	−95.2	−97.6	−110.1	−113.0	−96.1	−98.6	−101.2	−103.8	−100.9	−103.5	−102.4	−105.0	−103.2	−105.8
5-Hydroxy-2′,4′,5′,7-Tetra-methoxyflavone (**213**)	−99.4	−100.6	−110.2	−111.5	−105.0	−106.3	−112.5	−113.9	−95.6	−96.8	−102.3	−103.5	−104.1	−105.4
6,7-Dihydroxyflavone (**214**)	−76.1	−86.3	−87.5	−99.3	−80.8	−91.7	−92.0	−104.4	−88.6	−100.6	−85.4	−97.0	−87.9	−99.8
8-Methoxycirsilineol (**215**)	−93.3	−93.0	−117.9	−117.6	−103.7	−103.5	−118.0	−117.7	−99.6	−99.4	−96.4	−96.2	−107.6	−107.3
9,10-Dihydro-9,10-diacetoxy-3-methoxy-8,8-dimethyl-2-phenyl-4*H*,8*H*-benzo[1,2-*b*:3,4-*b*′]-dipyran-4-one (**222**)	−97.9	−91.7	−102.6	−96.1	−101.4	−95.0	−100.5	−94.2	−99.9	−93.6	−97.2	−91.0	−98.6	−92.4
Abyssinone I (**223**)	−85.5	−89.6	−108.9	−114.2	−87.8	−92.0	−98.8	−103.6	−86.9	−91.1	−87.4	−91.7	−101.1	−106.0
Abyssinone IV (**224**)	−98.6	−96.8	−125.9	−123.6	−107.4	−105.5	−117.3	−115.2	−113.0	−111.0	−116.1	−114.1	−120.3	−118.2
Astragalin (**225**)	−100.1	−94.1	−117.0	−109.9	−95.4	−89.6	−112.8	−106.0	−118.5	−111.3	−105.7	−99.3	−116.3	−109.3
Bavachinin (**226**)	−97.3	−100.4	−111.8	−115.4	−103.4	−106.7	−116.3	−120.0	−101.5	−104.8	−101.5	−104.7	−112.9	−116.5
Betuletol (**227**)	−82.6	−85.9	−102.6	−106.7	−82.1	−85.4	−98.4	−102.4	−87.1	−90.6	−90.9	−94.5	−103.1	−107.3
Bonannione A (**228**)	−81.3	−78.8	−129.0	−125.0	−121.6	−117.8	−120.4	−116.7	−112.2	−108.7	−110.6	−107.2	−116.7	−113.1
Brosimone I (**229**)	−90.4	−86.7	−114.0	−109.4	−100.1	−96.0	−110.2	−105.7	−100.8	−96.8	−97.9	−94.0	−102.0	−97.9
Cassiaflavan (**230**)	−76.1	−87.7	−85.7	−98.9	−75.4	−87.0	−84.3	−97.2	−76.6	−88.3	−82.1	−94.7	−83.3	−96.1
Cerasinone (**231**)	−83.4	−86.7	−101.3	−105.4	−91.2	−94.8	−106.2	−110.4	−88.8	−92.3	−95.3	−99.2	−99.4	−103.4
Chrysin (**233**)	−79.0	−89.6	−85.7	−97.3	−81.5	−92.5	−86.6	−98.2	−82.0	−93.1	−86.4	−98.1	−85.6	−97.2
Chrysoeriol (**232**)	−86.0	−92.4	−101.0	−108.5	−92.1	−98.9	−101.8	−109.3	−90.1	−96.7	−97.6	−104.8	−98.7	−106.0
Corniculatusin (**234**)	−95.1	−98.8	−105.0	−109.0	−103.0	−106.9	−109.1	−113.3	−103.6	−107.6	−97.5	−101.2	−100.0	−103.8
Cudraflavone A (**235**)	−85.8	−82.4	−108.6	−104.4	−97.2	−93.5	−100.2	−96.3	−96.7	−93.0	−85.7	−82.4	−100.4	−96.5
Dihydroquercetin (**236**)	−84.1	−89.9	−98.0	−104.8	−75.8	−81.1	−98.5	−105.3	−85.4	−91.3	−88.2	−94.3	−87.9	−93.9
Eucalyptin (**237**)	−87.4	−91.3	−98.6	−103.0	−90.4	−94.4	−95.8	−100.0	−93.4	−97.5	−94.0	−98.2	−101.9	−106.4
Euchrestaflavanone A (**238**)	−114.5	−111.0	−126.3	−122.4	−118.2	−114.6	−130.4	−126.3	−121.4	−117.6	−128.0	−124.1	−126.9	−123.0
Flavaprenin (**239**)	−99.0	−101.9	−101.7	−104.7	−108.9	−112.2	−107.6	−110.8	−103.2	−106.3	−104.9	−108.0	−112.7	−116.1
Flemiflavanone D (**240**)	−104.6	−100.0	−131.3	−125.6	−118.9	−113.7	−120.0	−114.8	−117.0	−111.9	−111.1	−106.3	−129.3	−123.7
Glabranin (**241**)	−94.8	−99.2	−102.6	−107.4	−99.2	−103.8	−103.5	−108.3	−101.9	−106.6	−102.7	−107.5	−107.0	−112.0
Isoorientin (**243**)	−81.8	−76.8	−111.7	−104.9	−103.4	−97.1	−107.0	−100.5	−101.9	−95.7	−102.1	−95.9	−117.2	−110.1
Isoscoparin (**244**)	−78.4	−72.9	−113.6	−105.7	−113.4	−105.5	−115.2	−107.1	−108.9	−101.2	−106.5	−99.0	−123.0	−114.4
Kaempferol (**242**)	−83.1	−90.7	−94.9	−103.6	−86.6	−94.5	−90.3	−98.5	−84.1	−91.8	−86.4	−94.3	−89.4	−97.5
Kushenol A (**245**)	−105.3	−102.1	−120.0	−116.3	−113.1	−109.6	−117.5	−113.9	−116.8	−113.2	−115.6	−112.0	−110.7	−107.3
Kushenol S (**246**)	−94.7	−97.6	−103.2	−106.3	−100.8	−103.8	−105.3	−108.4	−100.4	−103.4	−105.1	−108.2	−104.7	−107.8
Kushenol U (**247**)	−85.9	−82.3	−121.5	−116.5	−104.7	−100.3	−113.4	−108.7	−112.9	−108.2	−105.5	−101.0	−112.1	−107.4
Kushenol V (**248**)	−99.4	−96.9	−116.3	−113.4	−98.2	−95.8	−126.9	−123.7	−104.5	−101.9	−103.8	−101.2	−113.7	−110.9
Kushenol W (**249**)	−102.6	−101.3	−120.6	−119.0	−109.9	−108.5	−120.6	−119.1	−111.3	−109.8	−114.7	−113.2	−114.4	−112.9
Leachianone A (**250**)	−108.8	−102.9	−121.2	−114.7	−119.4	−113.0	−121.1	−114.6	−117.1	−110.9	−114.7	−108.6	−124.2	−117.6
Leachianone G (**251**)	−99.4	−100.8	−104.5	−106.0	−111.5	−113.1	−106.6	−108.1	−103.9	−105.4	−105.3	−106.8	−109.7	−111.3
Licoflavanone (**252**)	−94.2	−97.0	−111.9	−115.2	−107.2	−110.4	−111.1	−114.4	−99.1	−102.0	−106.2	−109.4	−107.3	−110.5
Licoflavone C (**253**)	−100.9	−104.1	−105.3	−108.7	−109.0	−112.5	−106.3	−109.7	−105.5	−108.9	−107.5	−110.9	−109.4	−112.9
Licoflavonol (**254**)	−85.6	−86.9	−113.1	−114.9	−96.7	−98.3	−113.6	−115.5	−98.0	−99.6	−95.6	−97.2	−104.9	−106.6
Lonchocarpol A (**255**)	−93.8	−90.9	−110.7	−107.3	−113.9	−110.3	−112.0	−108.5	−116.1	−112.5	−111.9	−108.5	−111.5	−108.0
Loranthin (**256**)	−106.0	−96.3	−122.8	−111.6	−113.8	−103.4	−114.0	−103.5	−119.4	−108.4	−122.4	−111.2	−117.2	−106.4
Loxophlebal A (**257**)	−99.3	−91.7	−119.1	−110.0	−100.1	−92.4	−112.6	−104.0	−103.0	−95.1	−98.8	−91.2	−129.2	−119.3
Lucenin 2 (**258**)	−100.5	−85.2	−131.5	−111.4	−116.3	−98.6	−120.8	−102.4	−118.1	−100.1	−101.5	−86.0	−138.6	−117.4
Macarangaflavanone A (**259**)	−105.6	−102.3	−127.0	−123.1	−118.4	−114.7	−123.0	−119.2	−117.4	−113.8	−130.7	−126.6	−113.7	−110.1
Malvidin (**260**)	−89.6	−93.1	−108.4	−112.7	−99.8	−103.7	−111.6	−115.9	−97.7	−101.6	−95.1	−98.8	−98.7	−102.6
Myricetin (**261**)	−86.3	−90.9	−105.2	−110.8	−95.8	−100.9	−103.0	−108.4	−92.8	−97.7	−88.0	−92.7	−98.8	−104.0
Natsudaidain (**262**)	−78.2	−75.2	−118.9	−114.3	−102.9	−99.0	−105.1	−101.0	−99.1	−95.3	−102.4	−98.4	−108.6	−104.4
Nevadensin (**263**)	−85.9	−88.1	−106.9	−109.6	−96.8	−99.3	−110.0	−112.9	−94.8	−97.2	−98.3	−100.9	−106.8	−109.6
*O*-Methylpongaglabol (**264**)	−93.3	−101.0	−97.6	−105.8	−102.5	−111.0	−100.2	−108.5	−96.8	−104.9	−95.7	−103.6	−99.3	−107.6
Paratocarpin L (**265**)	−107.6	−104.2	−127.2	−123.2	−119.5	−115.8	−128.1	−124.1	−117.8	−114.1	−106.1	−102.8	−118.8	−115.1
Persicogenin (**266**)	−89.7	−94.6	−103.1	−108.8	−92.5	−97.7	−98.8	−104.3	−95.2	−100.5	−94.0	−99.2	−100.2	−105.7
Pilosanol A (**267**)	−114.7	−101.2	−124.2	−109.7	−112.9	−99.7	−124.4	−109.8	−111.9	−98.8	−131.6	−116.2	−129.0	−113.9
Pilosanol B (**268**)	−111.7	−99.5	−129.2	−115.0	−97.9	−87.1	−124.4	−110.7	−118.2	−105.3	−124.3	−110.6	−127.4	−113.4
Pilosanol C (**269**)	−115.8	−103.1	−132.5	−118.0	−108.4	−96.5	−123.4	−109.9	−121.1	−107.8	−125.4	−111.7	−130.0	−115.7
Pinocembrin (**270**)	−76.4	−86.5	−86.2	−97.6	−79.4	−89.9	−84.6	−95.8	−79.0	−89.4	−84.1	−95.2	−83.4	−94.4
Pongaflavone (**271**)	−84.1	−87.1	−99.0	−102.6	−92.2	−95.5	−92.1	−95.4	−102.6	−106.3	−93.0	−96.3	−83.3	−86.3
Quercetin (**272**)	−83.6	−89.6	−100.0	−107.2	−92.3	−98.9	−95.5	−102.3	−89.1	−95.5	−88.0	−94.3	−94.4	−101.2
Quercetin 3-methyl ether (**273**)	−91.9	−96.9	−99.5	−105.0	−91.9	−97.0	−97.2	−102.6	−89.4	−94.3	−93.4	−98.5	−95.3	−100.6
Remangiflavanone A (**274**)	−101.5	−97.3	−124.9	−119.7	−113.6	−108.9	−114.2	−109.4	−113.5	−108.7	−103.6	−99.2	−114.4	−109.6
Remangiflavanone B (**275**)	−110.0	−105.2	−117.4	−112.3	−119.1	−113.9	−121.4	−116.2	−119.2	−114.0	−118.9	−113.7	−117.4	−112.3
Sanggenon G (**276**)	−35.5	−28.8	−127.7	−103.7	−108.3	−87.9	−124.4	−101.0	−129.3	−105.0	−131.1	−106.5	−178.3	−144.7
Sigmoidin A (**277**)	−108.2	−103.5	−126.6	−121.1	−117.3	−112.3	−126.1	−120.7	−119.8	−114.6	−114.1	−109.2	−128.5	−122.9
Sigmoidin B (**278**)	−98.1	−99.5	−117.3	−118.9	−111.4	−112.9	−114.3	−115.9	−102.2	−103.6	−100.5	−101.9	−112.4	−114.0
Sigmoidin L (**279**)	−106.5	−106.6	−119.4	−119.5	−117.4	−117.5	−119.7	−119.8	−108.0	−108.1	−106.9	−107.0	−115.3	−115.4
Siraitiflavandiol (**280**)	−96.6	−98.0	−113.0	−114.6	−101.8	−103.2	−102.4	−103.8	−97.6	−99.0	−97.4	−98.8	−114.1	−115.8
Solophenol D (**281**)	−108.3	−102.5	−127.4	−120.5	−119.4	−113.0	−136.5	−129.1	−122.7	−116.1	−122.9	−116.3	−123.8	−117.1
Sophoraflavanone G (**282**)	−102.9	−98.5	−128.2	−122.6	−115.7	−110.7	−120.5	−115.3	−116.5	−111.5	−116.6	−111.6	−116.4	−111.3
Sternbin (**283**)	−86.1	−92.3	−102.8	−110.1	−87.5	−93.7	−98.5	−105.5	−92.2	−98.8	−94.8	−101.6	−97.0	−103.9
Sudachitin (**284**)	−93.4	−94.4	−118.3	−119.5	−102.5	−103.5	−114.8	−116.0	−96.1	−97.1	−97.5	−98.5	−114.0	−115.2
Uvarinol (**285**)	−97.2	−84.1	−135.9	−117.6	−110.7	−95.8	−124.7	−107.8	−135.1	−116.8	−125.2	−108.3	−143.8	−124.4
Vahliabiflavone (**286**)	−95.7	−82.9	−104.3	−90.4	−106.0	−91.9	−104.9	−91.0	−119.0	−103.2	−106.5	−92.3	−110.4	−95.7
Vitexin (**287**)	−111.9	−106.4	−117.8	−112.0	−113.8	−108.2	−120.2	−114.2	−118.1	−112.3	−115.5	−109.8	−123.0	−117.0
Wogonin (**288**)	−87.6	−95.8	−94.1	−102.8	−91.1	−99.6	−96.2	−105.2	−91.0	−99.5	−93.4	−102.2	−94.0	−102.8
**Isoflavonoids**														
2″,3″-Epoxybolusanthol B (**289**)	−104.6	−104.6	−113.5	−113.4	−103.6	−103.5	−112.9	−112.8	−108.7	−108.7	−101.5	−101.4	−116.8	−116.7
3′,5,7-Trihydroxy-4′-methoxy-5′,6-diprenylisoflavanone (**290**)	−87.5	−82.8	−124.9	−118.2	−107.4	−101.6	−124.0	−117.4	−112.6	−106.6	−118.5	−112.1	−130.5	−123.5
4″-Hydroxydiphysolone (**292**)	−98.9	−98.8	−114.3	−114.2	−100.9	−100.8	−94.6	−94.6	−108.7	−108.6	−99.0	−99.0	−106.9	−106.8
5,7-Dihydroxy-2′-methoxy-3′,4′-methylenedioxyisoflavanone (**293**)	−83.4	−86.7	−96.7	−100.6	−86.3	−89.7	−93.0	−96.7	−93.9	−97.7	−89.1	−92.7	−99.6	−103.6
6*a*-Hydroxyphaseollin (**291**)	−76.2	−78.7	−101.8	−105.0	−91.8	−94.7	−92.1	−95.0	−84.4	−87.0	−82.2	−84.8	−103.1	−106.4
Amorphaquinone (**294**)	−80.8	−82.7	−99.5	−101.9	−89.0	−91.1	−98.9	−101.3	−89.5	−91.6	−90.5	−92.7	−93.1	−95.3
Asphodelin A (**295**)	−84.4	−93.9	−94.0	−104.5	−82.7	−91.9	−95.6	−106.3	−86.7	−96.5	−93.4	−103.9	−91.5	−101.7
Bidwillon A (**296**)	−55.7	−54.0	−127.1	−123.2	−110.0	−106.6	−108.6	−105.2	−113.7	−110.1	−105.4	−102.2	−120.3	−116.6
Bolucarpan A (**297**)	−82.8	−83.0	−97.4	−97.7	−92.9	−93.2	−89.8	−90.0	−89.4	−89.7	−102.1	−102.4	−92.6	−92.9
Bolucarpan B (**298**)	−77.9	−78.3	−101.1	−101.6	−91.9	−92.4	−85.8	−86.3	−90.3	−90.7	−104.1	−104.6	−96.0	−96.5
Bolucarpan D (**299**)	−77.9	−80.5	−97.4	−100.7	−89.2	−92.2	−80.6	−83.3	−86.7	−89.7	−100.3	−103.6	−91.1	−94.2
Bolusanthol B (**300**)	−98.2	−99.6	−112.0	−113.6	−101.1	−102.5	−108.3	−109.8	−107.2	−108.8	−100.2	−101.6	−112.5	−114.1
Cajanol (**301**)	−81.1	−85.6	−93.8	−98.9	−86.1	−90.9	−87.2	−92.1	−87.0	−91.8	−85.9	−90.6	−91.8	−96.9
Chandalone (**302**)	−63.0	−61.2	−122.2	−118.8	−113.3	−110.2	−116.7	−113.5	−109.3	−106.2	−109.8	−106.8	−122.2	−118.8
Dalversinol A (**303**)	−108.9	−104.2	−118.9	−113.8	−104.5	−100.0	−113.8	−108.9	−111.8	−106.9	−110.1	−105.4	−114.0	−109.1
Derrisin (**304**)	−88.8	−84.7	−97.1	−92.6	−81.8	−78.0	−82.9	−79.1	−82.4	−78.6	−86.6	−82.6	−87.0	−83.0
Erybraedin A (**305**)	−91.4	−89.8	−120.1	−117.9	−100.8	−99.0	−110.0	−108.1	−100.3	−98.5	−101.8	−100.0	−116.9	−114.8
Erybraedin D (**306**)	−97.9	−96.3	−106.6	−104.9	−102.5	−100.8	−107.0	−105.2	−96.4	−94.8	−99.7	−98.0	−106.0	−104.2
Erypoegin I (**307**)	−101.8	−101.9	−111.1	−111.2	−106.9	−107.0	−108.7	−108.9	−117.0	−117.2	−100.6	−100.8	−112.1	−112.2
Erysubin F (**308**)	−119.6	−117.7	−124.6	−122.5	−124.4	−122.4	−115.0	−113.1	−116.6	−114.7	−126.4	−124.3	−129.8	−127.7
Eryvarin V (**309**)	−91.8	−88.0	−115.0	−110.1	−96.8	−92.7	−114.8	−110.0	−104.3	−99.9	−98.7	−94.6	−99.2	−95.0
Eryvarin W (**310**)	−109.5	−107.8	−122.5	−120.5	−118.8	−116.9	−115.8	−113.9	−122.4	−120.4	−115.4	−113.5	−120.0	−118.0
Eryzerin C (**311**)	−75.0	−73.6	−122.3	−119.9	−108.0	−105.9	−110.1	−108.0	−113.5	−111.3	−106.6	−104.5	−120.3	−117.9
Eryzerin D (**312**)	−76.6	−75.2	−112.1	−110.1	−96.4	−94.6	−108.3	−106.3	−104.7	−102.9	−103.4	−101.6	−103.9	−102.0
Euchretin A (**313**)	−25.3	−22.9	−114.5	−103.6	−115.6	−104.5	−99.2	−89.7	−115.4	−104.3	−114.4	−103.5	−122.2	−110.5
Gancaonin C (**314**)	−105.9	−107.6	−116.7	−118.6	−104.0	−105.7	−113.7	−115.5	−108.7	−110.5	−108.4	−110.2	−115.2	−117.1
Genistein (**315**)	−81.3	−90.4	−92.5	−102.9	−78.6	−87.4	−86.0	−95.6	−75.6	−84.1	−82.5	−91.7	−87.7	−97.6
Glycyrrhisoflavone (**316**)	−98.3	−99.9	−113.9	−115.8	−110.6	−112.4	−109.4	−111.1	−102.1	−103.7	−105.9	−107.6	−116.2	−118.1
Hispaglabridin A (**317**)	−38.2	−37.5	−117.1	−115.0	−107.8	−105.8	−111.6	−109.6	−93.7	−92.0	−106.3	−104.4	−118.1	−115.9
Hispaglabridin B (**318**)	−71.6	−70.4	−113.3	−111.4	−91.8	−90.3	−111.1	−109.3	−79.1	−77.8	−88.5	−87.0	−97.2	−95.6
Hydroxycristacarpone (**319**)	−96.7	−96.8	−112.7	−112.8	−108.2	−108.4	−100.5	−100.6	−113.0	−113.2	−110.3	−110.4	−100.3	−100.4
Isoneorautenol (**320**)	−82.2	−86.2	−96.1	−100.8	−83.2	−87.2	−80.1	−84.0	−83.3	−87.4	−87.9	−92.1	−86.5	−90.7
Lachnoisoflavone A (**321**)	−65.6	−66.8	−118.5	−120.6	−107.3	−109.2	−93.5	−95.1	−105.3	−107.2	−105.5	−107.4	−98.4	−100.2
Licoisoflavanone (**322**)	−76.9	−78.2	−105.5	−107.2	−91.8	−93.2	−103.0	−104.7	−87.4	−88.8	−84.6	−85.9	−100.7	−102.3
Licoricidin (**323**)	−106.3	−101.7	−122.2	−116.9	−114.1	−109.1	−125.1	−119.7	−114.8	−109.8	−114.5	−109.6	−117.0	−111.9
Lupinalbin C (**324**)	−79.3	−79.5	−104.0	−104.3	−94.1	−94.3	−90.1	−90.4	−94.3	−94.5	−92.5	−92.7	−97.1	−97.4
Mucronulatol (**326**)	−83.9	−89.9	−98.8	−105.9	−90.1	−96.5	−96.0	−102.9	−80.9	−86.7	−86.8	−93.0	−94.0	−100.7
Neomillinol (**325**)	−81.1	−84.7	−106.1	−110.8	−96.8	−101.1	−94.9	−99.1	−93.6	−97.7	−85.8	−89.6	−101.5	−106.0
Pendulone (**327**)	−77.9	−82.2	−98.2	−103.6	−85.9	−90.6	−94.6	−99.8	−81.4	−85.9	−84.3	−89.0	−92.1	−97.2
Phyllanone B (**328**)	−96.4	−91.2	−121.7	−115.1	−115.3	−109.2	−116.5	−110.3	−116.0	−109.8	−117.0	−110.7	−122.4	−115.9
Shinpterocarpin (**329**)	−73.3	−76.8	−88.0	−92.3	−84.4	−88.5	−82.2	−86.2	−85.0	−89.2	−82.0	−86.0	−85.9	−90.1
**Neoflavonoids**														
Inophyllum A (**330**)	−98.8	−96.0	−109.9	−106.9	−100.1	−97.3	−102.0	−99.2	−102.2	−99.4	−97.5	−94.8	−105.0	−102.1
Inophyllum C (**331**)	−95.4	−92.9	−104.1	−101.4	−92.0	−89.6	−103.4	−100.7	−102.7	−100.0	−92.6	−90.1	−105.8	−103.0
Mammea A/BA (**332**)	−107.7	−104.5	−123.2	−119.6	−118.6	−115.1	−120.5	−116.9	−119.6	−116.1	−110.5	−107.2	−125.6	−121.9
Mammea A/BB (**333**)	−101.4	−98.4	−120.8	−117.3	−114.7	−111.3	−112.3	−109.0	−117.6	−114.2	−107.2	−104.0	−121.8	−118.3
Mesuol (**334**)	−92.5	−90.8	−118.7	−116.6	−114.1	−112.0	−114.2	−112.2	−107.6	−105.6	−102.7	−100.8	−112.0	−110.0
**Pterocarpans**														
1-Methoxyphaseollidin (**356**)	−92.1	−93.6	−105.6	−107.3	−109.5	−111.2	−112.3	−114.1	−105.1	−106.8	−99.0	−100.5	−104.9	−106.6
Aracarpene 1 (**357**)	−83.2	−89.3	−95.0	−102.0	−88.1	−94.6	−85.7	−92.0	−97.9	−105.1	−88.3	−94.8	−89.7	−96.3
Aracarpene 2 (**358**)	−81.3	−87.3	−98.1	−105.3	−90.1	−96.7	−88.6	−95.2	−94.9	−101.9	−86.0	−92.3	−91.3	−98.1
Calopocarpin (**360**)	−96.7	−101.2	−103.8	−108.7	−108.1	−113.2	−103.3	−108.1	−103.8	−108.7	−103.0	−107.8	−100.8	−105.5
Cristacarpin (**359**)	−82.4	−83.7	−107.2	−108.9	−107.9	−109.7	−102.8	−104.5	−108.1	−109.8	−95.7	−97.2	−102.3	−104.0
Erythbidin D (**362**)	−84.8	−91.1	−92.4	−99.2	−92.7	−99.5	−94.1	−101.1	−90.4	−97.1	−85.8	−92.1	−91.5	−98.2
Eryzerin E (**363**)	−100.9	−96.7	−122.4	−117.2	−103.3	−98.9	−111.1	−106.4	−113.7	−108.9	−114.5	−109.7	−120.6	−115.6
Fuscacarpan B (**364**)	−86.2	−86.3	−111.4	−111.6	−99.6	−99.7	−98.3	−98.4	−102.5	−102.7	−96.5	−96.6	−105.8	−105.9
Fuscacarpan C (**365**)	−84.7	−84.8	−112.7	−112.8	−100.9	−101.0	−101.4	−101.5	−113.2	−113.4	−98.0	−98.1	−102.9	−103.0
Glycyrol (**366**)	−90.2	−90.6	−105.5	−106.0	−105.9	−106.4	−102.2	−102.7	−103.6	−104.1	−101.9	−102.4	−94.9	−95.4
Glycyrrhizol A (**367**)	−90.8	−87.1	−120.0	−115.2	−120.9	−116.0	−109.4	−105.0	−109.5	−105.1	−106.8	−102.5	−117.0	−112.3
Glycyrrhizol B (**368**)	−88.3	−90.1	−104.6	−106.7	−98.7	−100.7	−94.4	−96.3	−103.6	−105.6	−100.1	−102.1	−98.0	−99.9
Sandwicensin (**369**)	−83.2	−85.9	−105.3	−108.6	−104.1	−107.4	−107.0	−110.4	−103.4	−106.7	−96.3	−99.3	−98.9	−102.1
Variabilin (**370**)	−77.0	−82.7	−93.1	−99.9	−94.7	−101.7	−84.6	−90.8	−98.1	−105.3	−89.2	−95.8	−93.7	−100.6
*ent*-Sophoracarpan A (**361**)	−79.2	−85.0	−96.6	−103.7	−90.0	−96.7	−97.6	−104.8	−92.3	−99.1	−92.8	−99.6	−95.4	−102.5
**Chromones**														
3-(3-Hydroxy-4-methoxy-benzylidene)-6,7-dimethoxy-4-chromanone (**371**)	−78.1	−80.2	−113.4	−116.6	−97.5	−100.2	−99.4	−102.2	−94.3	−96.9	−85.2	−87.5	−97.7	−100.5
4′,5,7-Trihydroxy-6,8-dimethyl-homoisoflavanone (**372**)	−83.5	−88.3	−105.0	−111.0	−89.2	−94.4	−98.5	−104.2	−86.6	−91.6	−86.7	−91.7	−98.2	−103.9
4′,5,7-Trihydroxy-6-methyl-homoisoflavanone (**373**)	−79.0	−84.8	−102.7	−110.3	−86.7	−93.1	−98.7	−105.9	−84.3	−90.5	−95.3	−102.3	−91.9	−98.7
7-*O*-Methylbonducellin (**374**)	−75.5	−81.4	−102.1	−110.2	−84.0	−90.6	−92.8	−100.1	−76.9	−83.0	−83.5	−90.0	−89.5	−96.5
8-Methylophiopogonanone B (**375**)	−86.3	−90.0	−108.3	−112.9	−87.7	−91.4	−103.9	−108.3	−94.9	−98.9	−92.6	−96.5	−104.1	−108.4
Bonducellin (**376**)	−75.3	−82.5	−95.5	−104.7	−83.3	−91.2	−90.0	−98.7	−79.6	−87.3	−80.3	−88.0	−90.8	−99.6
Odoratumone A (**377**)	−89.3	−91.6	−114.3	−117.2	−97.6	−100.2	−110.5	−113.3	−98.4	−101.0	−97.6	−100.1	−110.7	−113.6
Sappanone A 3′,4′-methylene ether (**378**)	−81.8	−88.2	−102.1	−110.1	−92.3	−99.5	−94.4	−101.8	−86.4	−93.2	−83.6	−90.2	−94.5	−101.9
Sappanone A 4′-methyl ether (**379**)	−79.9	−86.0	−101.4	−109.1	−81.4	−87.6	−94.1	−101.2	−87.7	−94.4	−82.4	−88.7	−97.0	−104.4
Sappanone A trimethyl ether (**380**)	−82.4	−86.1	−111.8	−116.7	−89.0	−92.9	−107.5	−112.3	−90.2	−94.2	−89.7	−93.6	−94.8	−99.0
**Condensed Tannins**														
GB 1 (**381**)	−121.0	−105.6	−118.3	−103.3	−100.9	−88.1	−127.4	−111.2	−145.4	−127.0	−123.4	−107.8	−116.1	−101.4
Proanthocyanidin A_1_ (**382**)	−95.2	−82.3	−126.7	−109.4	−129.2	−111.6	−105.9	−91.5	−116.3	−100.5	−122.7	−106.0	−117.6	−101.6
Proanthocyanidin A_2_ (**383**)	−94.3	−81.5	−109.1	−94.3	−130.1	−112.4	−109.4	−94.5	−110.2	−95.2	−121.5	−105.0	−113.9	−98.4
Procyanidin B_4_ (**384**)	−97.8	−84.4	−118.8	−102.5	−98.7	−85.1	−115.7	−99.8	−123.1	−106.2	−112.6	−97.2	−113.5	−97.9
Procyanidin B_5_ (**385**)	23.0	19.9	−114.3	−98.7	−111.8	−96.5	−131.1	−113.1	−131.3	−113.3	−115.6	−99.7	−146.4	−126.3
Procyanidin B_6_ (**386**)	−68.6	−59.2	−123.6	−106.6	−101.3	−87.4	−111.5	−96.2	−135.0	−116.5	−121.6	−104.9	−126.1	−108.8
Teatannin (**387**)	−96.6	−91.1	−131.8	−124.4	−103.4	−97.6	−118.4	−111.8	−107.6	−101.6	−104.7	−98.8	−124.7	−117.7
**Coumarins**														
4,5′,8′-Trihydroxy-5-methyl-3,7′-bicoumarin (**344**)	−97.3	−99.0	−104.3	−106.2	−89.6	−91.2	−103.4	−105.3	−95.2	−96.9	−92.9	−94.6	−104.1	−106.0
6-Geranyl-5,7-dihydroxy-8(2-methylbutanoyl)-4-phenyl-coumarin (**345**)	−112.6	−103.8	−127.7	−117.7	−115.8	−106.8	−128.6	−118.5	−122.3	−112.7	−121.3	−111.8	−132.2	−121.9
(–)-Heliettin (**354**)	−70.0	−89.5	−72.8	−93.1	−66.9	−85.5	−73.5	−93.9	−70.0	−89.4	−71.3	−91.1	−70.4	−89.9
Aesculin (**347**)	−99.8	−102.7	−111.0	−114.4	−104.2	−107.3	−103.4	−106.5	−100.9	−103.9	−97.8	−100.7	−102.1	−105.1
Alloimperatorin (**346**)	−95.2	−105.8	−100.7	−112.0	−99.5	−110.7	−101.6	−112.9	−97.1	−107.9	−97.7	−108.6	−98.4	−109.4
Anofinic acid (**348**)	−75.4	−92.0	−80.1	−97.8	−77.9	−95.1	−78.2	−95.4	−77.7	−94.9	−78.1	−95.3	−83.4	−101.8
Calaustralin (**349**)	−90.9	−88.4	−111.9	−108.8	−111.2	−108.1	−107.3	−104.3	−100.5	−97.8	−97.5	−94.7	−114.3	−111.1
Calophyllolide (**350**)	−94.3	−90.8	−120.9	−116.4	−111.0	−106.9	−111.4	−107.3	−113.9	−109.6	−114.6	−110.4	−101.2	−97.4
Dicoumarol (**351**)	−95.2	−98.4	−101.6	−105.1	−80.7	−83.5	−96.8	−100.1	−93.5	−96.6	−90.3	−93.4	−99.6	−103.0
Dipetalolactone (**352**)	−84.9	−90.1	−95.2	−101.1	−101.3	−107.6	−96.3	−102.2	−89.8	−95.3	−90.7	−96.3	−91.2	−96.9
Glycycoumarin (**353**)	−89.1	−89.4	−113.1	−113.5	−106.0	−106.3	−102.7	−103.0	−100.7	−101.0	−95.5	−95.8	−100.3	−100.6
Marmesin (**355**)	−69.8	−80.0	−87.2	−100.0	−80.1	−91.9	−84.0	−96.3	−81.7	−93.8	−83.8	−96.1	−77.9	−89.3
**Stilbenoids**														
2-(2,4-Dihydroxyphenyl-5-(1-propenyl)benzofuran (**388**)	−82.8	−92.6	−96.0	−107.3	−90.3	−100.9	−97.5	−109.0	−85.2	−95.2	−88.9	−99.3	−94.9	−106.0
Albanol A (**389**)	−79.4	−69.2	−116.3	−101.3	−101.2	−88.1	−125.2	−109.1	−111.8	−97.4	−106.7	−92.9	−147.1	−128.1
Albanol B (**390**)	no dock	no dock	−138.1	−120.5	−23.4	−20.4	−109.7	−95.8	−106.7	−93.2	−113.0	−98.7	−114.7	−100.2
Amorfrutin A (**391**)	−100.4	−103.3	−121.8	−125.4	−103.6	−106.7	−117.0	−120.5	−113.0	−116.3	−109.3	−112.5	−118.5	−122.0
Cajaninstilbene acid (**392**)	−97.3	−100.3	−116.3	−120.0	−99.0	−102.1	−112.7	−116.3	−97.3	−100.4	−97.9	−101.0	−118.9	−122.6
Calodenin B (**393**)	−114.8	−102.4	−148.6	−132.5	−135.7	−121.0	−150.0	−133.7	−157.6	−140.6	−143.5	−127.9	−147.0	−131.1
Centrolobofuran (**394**)	−82.1	−93.0	−95.3	−107.9	−83.6	−94.7	−93.4	−105.8	−85.8	−97.1	−84.0	−95.0	−90.6	−102.6
Cochinchinenene A (**395**)	−84.4	−74.5	−113.7	−100.3	−102.5	−90.5	−107.9	−95.2	−134.6	−118.8	−119.9	−105.8	−135.8	−119.9
Cochinchinenene B (**396**)	−101.2	−90.9	−145.2	−130.5	−101.1	−90.9	−124.5	−111.9	−130.4	−117.2	−119.8	−107.6	−156.8	−140.8
Cochinchinenene C (**397**)	−114.8	−104.1	−135.8	−123.1	−133.2	−120.7	−117.1	−106.2	−133.7	−121.2	−116.3	−105.4	−138.2	−125.3
Cochinchinenene D (**398**)	−127.5	−116.7	−140.3	−128.4	−107.9	−98.7	−121.1	−110.9	−124.0	−113.5	−121.1	−110.8	−145.0	−132.8
Egonol (**399**)	−108.1	−112.9	−120.1	−125.4	−103.9	−108.5	−114.9	−119.9	−104.1	−108.7	−105.0	−109.6	−119.2	−124.5
Erypoegin F (**400**)	−104.3	−106.2	−122.4	−124.6	−120.3	−122.4	−121.9	−124.1	−115.1	−117.2	−114.8	−116.9	−117.1	−119.2
Erythbidin E (**401**)	−53.5	−60.6	−97.7	−110.6	−86.9	−98.3	−91.0	−103.0	−87.0	−98.5	−85.5	−96.8	−91.8	−103.9
Eryvarin Q (**402**)	−119.5	−116.0	−140.8	−136.7	−126.3	−122.6	−143.0	−138.8	−130.9	−127.1	−131.6	−127.7	−133.9	−130.0
Gancaonin I (**403**)	−108.9	−110.6	−120.0	−121.9	−103.7	−105.4	−120.9	−122.9	−110.5	−112.2	−108.4	−110.2	−120.8	−122.7
Glyinflanin H (**404**)	−90.5	−96.3	−101.8	−108.4	−88.3	−94.0	−102.3	−108.9	−86.5	−92.0	−88.9	−94.6	−90.9	−96.7
Kuwanol A (**405**)	−74.5	−64.8	−111.1	−96.7	−101.9	−88.7	−129.6	−112.8	−119.2	−103.7	−116.2	−101.1	−155.8	−135.6
Licobenzofuran (**406**)	−106.3	−108.0	−117.4	−119.2	−119.7	−121.6	−112.6	−114.4	−109.1	−110.8	−112.5	−114.3	−113.3	−115.1
Licocoumarone (**407**)	−102.0	−105.0	−120.5	−124.1	−98.7	−101.7	−120.7	−124.3	−104.4	−107.5	−98.6	−101.5	−118.4	−122.0
Mulberrofuran D (**408**)	−122.0	−114.8	−145.5	−136.9	−120.8	−113.6	−136.9	−128.8	−129.7	−122.0	−129.5	−121.8	−134.3	−126.3
Mulberrofuran Y (**409**)	−114.6	−111.0	−134.3	−130.1	−113.6	−110.0	−128.8	−124.8	−126.8	−122.9	−123.2	−119.4	−137.9	−133.6
Pinosylvin (**410**)	−61.5	−74.1	−88.4	−106.6	−77.5	−93.4	−78.8	−95.0	−74.9	−90.3	−76.6	−92.3	−80.0	−96.4
Schweinfurthin A (**411**)	−30.4	−26.7	−82.8	−72.7	−114.6	−100.7	−113.9	−100.1	−116.6	−102.4	−120.7	−106.0	−123.6	−108.5
Shanciguol 3-methyl ether (**412**)	−107.2	−101.1	−125.1	−118.0	−112.9	−106.5	−119.7	−113.0	−122.2	−115.3	−116.9	−110.3	−130.9	−123.5
Stemofuran R (**413**)	−92.6	−96.5	−110.7	−115.3	−95.4	−99.4	−106.3	−110.8	−98.9	−103.0	−100.8	−105.0	−106.3	−110.8
Stilbostemin S (**414**)	−92.1	−100.3	−107.7	−117.2	−93.5	−101.8	−102.0	−111.0	−101.8	−110.8	−101.6	−110.6	−97.8	−106.4
Thunberginol F (**415**)	−85.5	−95.1	−99.1	−110.2	−85.7	−95.3	−96.7	−107.6	−89.3	−99.3	−86.8	−96.6	−96.0	−106.8
(7*E*,7′*R*,8′*R*)-ε-Viniferin (**416**)	−101.7	−95.1	−134.0	−125.3	−118.1	−110.4	−110.8	−103.6	−114.7	−107.3	−115.6	−108.1	−131.7	−123.2
(7*E*,7′*S*,8′*S*)-ε-Viniferin (**417**)	−103.2	−96.5	−134.3	−125.6	−112.6	−105.3	−111.0	−103.8	−119.1	−111.3	−118.8	−111.1	−126.9	−118.7
**Phenylpropanoids and Lignans**														
(*E*)-Cinnamaldehyde (**418**)	−69.5	−98.1	−70.6	−99.6	−66.4	−93.7	−71.3	−100.6	−64.3	−90.7	−66.8	−94.2	−68.2	−96.3
3,4-Dimethylcinnamaldehyde (**419**)	−77.2	−102.2	−81.6	−108.0	−72.2	−95.5	−77.2	−102.3	−77.1	−102.0	−78.7	−104.2	−79.4	−105.1
Methyleugenol (**422**)	−79.9	−102.1	−81.8	−104.5	−74.8	−95.6	−78.1	−99.8	−79.6	−101.7	−80.4	−102.7	−79.8	−101.9
*p*-Coumaraldehyde (**420**)	−73.8	−100.3	−75.9	−103.2	−67.9	−92.3	−72.8	−99.0	−69.8	−94.8	−72.5	−98.6	−74.3	−101.0
*p*-Methoxycinnamaldehyde (**421**)	−79.0	−104.2	−82.1	−108.2	−71.7	−94.5	−78.5	−103.5	−74.1	−97.7	−76.3	−100.6	−81.3	−107.2
(–)-Asarinin (**423**)	−98.1	−99.6	−120.0	−121.9	−96.7	−98.2	−106.8	−108.5	−109.4	−111.1	−110.6	−112.4	−117.3	−119.2
Nordihydroguaiaretic acid (**424**)	−88.6	−94.9	−106.0	−113.6	−103.9	−111.3	−100.1	−107.3	−98.0	−105.0	−93.5	−100.1	−104.6	−112.1
**Xanthones**														
2-Deoxy-4-Hydroxycudratricus-xanthone D (**425**)	−92.1	−90.3	−103.2	−101.2	−110.9	−108.8	−108.2	−106.1	−97.7	−95.8	−96.2	−94.3	−96.4	−94.5
Calozeyloxanthone (**426**)	−86.8	−86.3	−88.3	−87.8	−83.8	−83.3	−86.3	−85.8	−92.6	−92.1	−99.1	−98.5	−92.1	−91.6
Cycloartobiloxanthone (**427**)	−89.3	−84.8	−99.1	−94.0	−101.4	−96.3	−103.8	−98.6	−98.4	−93.5	−96.6	−91.7	−94.4	−89.6
Formoxanthone C (**428**)	−103.0	−100.8	−101.9	−99.8	−107.6	−105.3	−105.2	−103.0	−108.6	−106.3	−104.4	−102.2	−107.6	−105.3
Garciniacowone (**429**)	−71.0	−65.8	−124.5	−115.4	−110.4	−102.3	−106.7	−98.9	−123.0	−114.0	−115.0	−106.6	−119.9	−111.1
Globulixanthone C (**430**)	−81.5	−85.1	−102.8	−107.3	−90.9	−95.0	−94.4	−98.6	−94.5	−98.7	−90.6	−94.6	−89.5	−93.5
Globulixanthone D (**431**)	−90.9	−93.4	−109.6	−112.6	−88.5	−90.9	−99.0	−101.7	−98.4	−101.2	−98.9	−101.7	−99.6	−102.3
Globulixanthone E (**432**)	−68.0	−57.3	−114.7	−96.8	−110.9	−93.6	−89.7	−75.7	−104.9	−88.6	−110.4	−93.2	−124.0	−104.6
Morellin (**433**)	−88.3	−77.7	−92.7	−81.6	−102.7	−90.4	−98.8	−87.0	−107.7	−94.8	−107.7	−94.8	−97.7	−86.0
Nigrolineaxanthone N (**434**)	−98.5	−96.2	−105.1	−102.7	−99.2	−97.0	−103.5	−101.1	−120.9	−118.1	−106.5	−104.1	−99.0	−96.7
Pinselin (**435**)	−94.0	−100.9	−91.2	−97.9	−95.4	−102.4	−99.2	−106.5	−102.7	−110.2	−96.4	−103.5	−98.3	−105.6
Scortechinone B (**436**)	−95.9	−82.1	−123.2	−105.5	−112.5	−96.3	−85.9	−73.5	−113.0	−96.7	−102.3	−87.6	−107.4	−91.9
Symphonin (**437**)	−97.5	−91.3	−107.7	−100.9	−109.3	−102.4	−103.9	−97.3	−106.0	−99.2	−103.0	−96.5	−94.7	−88.7
**Hydrolyzable Tannins**														
1,2,3,4,6-Pentagalloylglucose (**335**)	−115.6	−84.8	−178.0	−130.6	−148.1	−108.7	−161.0	−118.1	−157.5	−115.6	−151.6	−111.3	−184.7	−135.5
Aceritannin (**438**)	−112.5	−104.1	−133.9	−123.9	−133.0	−123.1	−146.6	−135.7	−128.6	−119.0	−139.2	−128.9	−121.8	−112.8
Ginnalin B (**439**)	−92.2	−97.3	−101.3	−106.9	−102.7	−108.4	−104.0	−109.7	−100.2	−105.7	−99.4	−104.9	−106.3	−112.1
Ginnalin C (**440**)	−88.4	−93.3	−106.6	−112.5	−94.6	−99.8	−110.4	−116.5	−100.8	−106.4	−94.1	−99.3	−106.0	−111.9
Panconoside A (**441**)	−79.7	−65.9	−137.5	−113.6	−118.4	−97.8	−124.5	−102.9	−119.3	−98.6	−112.5	−93.0	−134.2	−110.9
**Miscellaneous Phenolics**														
1,3,7,9-Tetrahydroxy-4,6-dimethyl-2,8-bis(2-methyl-propanoyl)-dibenzofuran (**442**)	−93.7	−91.4	−119.9	−117.0	−104.7	−102.1	−106.2	−103.6	−102.4	−99.8	−100.3	−97.9	−108.3	−105.7
2′,4′-Dihydroxy-6′-methoxy-3′-methylacetophenone (**443**)	−76.2	−94.3	−76.7	−94.9	−75.5	−93.4	−77.6	−96.0	−74.5	−92.2	−75.7	−93.7	−74.7	−92.4
3′,4′-Dihydroxyacetophenone (**444**)	−68.6	−92.4	−71.7	−96.6	−67.3	−90.6	−70.5	−95.0	−70.3	−94.7	−70.2	−94.5	−68.1	−91.8
4′-*O*-Methylhonokiol (**446**)	−95.2	−104.6	−107.7	−118.3	−100.8	−110.7	−107.5	−118.1	−97.7	−107.3	−97.0	−106.6	−100.2	−110.1
4-Deoxyadhumulone 2″,3″-epoxide (**445**)	−75.3	−75.9	−114.6	−115.5	−97.6	−98.4	−104.9	−105.8	−103.4	−104.2	−98.7	−99.5	−109.0	−109.9
7-(3,4-Dihydroxy-5-methoxy-phenyl)-1-phenyl-4-hepten-3-one (**447**)	−103.6	−108.2	−124.2	−129.7	−104.9	−109.5	−121.5	−126.9	−111.0	−115.9	−110.6	−115.5	−117.6	−122.8
Agrimol C (**449**)	−102.3	−84.1	−136.6	−112.3	−104.0	−85.5	−127.8	−105.1	−126.9	−104.4	−126.4	−103.9	−119.5	−98.3
Agrimol F (**450**)	−107.7	−89.9	−127.3	−106.1	−106.0	−88.4	−121.5	−101.3	−126.6	−105.6	−122.2	−101.9	−125.8	−104.9
Agrimol G (**451**)	−85.0	−69.9	−128.4	−105.5	−104.6	−86.0	−122.2	−100.5	−132.6	−109.0	−124.6	−102.4	−134.9	−110.9
Arzanol (**452**)	−96.8	−94.3	−118.6	−115.5	−89.1	−86.8	−118.2	−115.1	−102.1	−99.4	−108.1	−105.3	−110.5	−107.6
Aspidinol C (**448**)	−82.6	−97.7	−92.9	−109.9	−85.4	−101.1	−89.8	−106.3	−89.2	−105.5	−93.9	−111.1	−90.1	−106.7
Bruguierol C (**453**)	−80.3	−97.7	−76.2	−92.7	−76.7	−93.4	−80.0	−97.3	−78.7	−95.7	−80.0	−97.4	−76.4	−93.0
Cearoin (**454**)	−87.2	−100.3	−91.7	−105.4	−90.1	−103.6	−88.6	−101.9	−94.3	−108.5	−89.2	−102.6	−87.1	−100.1
Citrusnin A (**455**)	−99.2	−116.1	−101.3	−118.5	−92.4	−108.1	−101.4	−118.6	−99.2	−116.0	−100.4	−117.5	−101.7	−119.0
Cochinchinenin B (**456**)	−126.9	−111.9	−140.4	−123.8	−123.6	−109.0	−132.2	−116.6	−127.4	−112.3	−139.8	−123.2	−139.4	−122.8
Cochinchinenin C (**457**)	−127.2	−112.2	−132.9	−117.1	−123.3	−108.7	−121.7	−107.3	−132.0	−116.3	−130.5	−115.0	−146.7	−129.3
Drummondin D (**458**)	−87.0	−79.0	−125.2	−113.7	−112.3	−102.0	−121.0	−109.9	−112.3	−101.9	−113.2	−102.8	−120.9	−109.7
Drummondin E (**459**)	−112.1	−101.7	−129.4	−117.4	−107.8	−97.7	−125.8	−114.1	−117.4	−106.5	−122.4	−111.0	−126.4	−114.6
Eleutherol (**460**)	−86.3	−99.2	−94.1	−108.3	−87.5	−100.7	−94.4	−108.5	−90.6	−104.2	−90.8	−104.5	−90.6	−104.2
Ellagicacid (**461**)	−91.6	−98.1	−98.4	−105.4	−98.3	−105.3	−98.7	−105.7	−96.3	−103.1	−98.4	−105.5	−96.1	−103.0
Epicoccolide A (**462**)	−81.3	−81.1	−91.8	−91.6	−91.7	−91.5	−84.0	−83.8	−92.5	−92.3	−90.3	−90.1	−93.6	−93.4
Gibbilimbol A (**464**)	−86.3	−100.9	−102.8	−120.2	−91.4	−106.8	−91.9	−107.5	−96.1	−112.4	−91.3	−106.8	−92.8	−108.5
Gibbilimbol B (**465**)	−89.6	−104.8	−101.1	−118.3	−95.2	−111.3	−96.8	−113.3	−95.5	−111.7	−95.2	−111.3	−94.8	−110.8
Grifolin (**466**)	−101.2	−105.5	−124.0	−129.2	−106.3	−110.7	−119.4	−124.4	−109.3	−113.9	−120.1	−125.2	−116.4	−121.3
Hyperbrasilol A (**467**)	−18.6	−16.2	−131.4	−114.0	−113.4	−98.4	−127.0	−110.2	−115.4	−100.1	−87.5	−76.0	−123.3	−107.0
Hyperbrasilol B (**468**)	−104.7	−91.7	−133.0	−116.5	−108.6	−95.1	−125.6	−110.1	−114.7	−100.5	−111.4	−97.6	−127.3	−111.5
Hyperbrasilol C (**469**)	−110.4	−96.6	−131.6	−115.1	−108.6	−95.1	−115.9	−101.4	−117.1	−102.5	−111.8	−97.9	−120.6	−105.5
Isodrummondin D (**470**)	−105.0	−95.4	−122.9	−111.6	−108.6	−98.6	−113.1	−102.7	−116.4	−105.7	−115.6	−105.0	−120.1	−109.0
Isohyperbrasilol B (**471**)	−106.2	−93.0	−122.9	−107.6	−111.7	−97.9	−118.6	−103.9	−123.7	−108.4	−113.7	−99.6	−133.8	−117.2
Isouliginosin B (**472**)	−104.7	−94.9	−119.5	−108.4	−114.8	−104.1	−119.5	−108.4	−117.7	−106.8	−105.4	−95.6	−125.0	−113.3
Italipyrone (**473**)	−104.2	−101.6	−120.5	−117.5	−105.5	−102.9	−115.0	−112.2	−108.1	−105.4	−105.4	−102.8	−112.2	−109.5
Knerachelin A (**474**)	−100.6	−108.0	−115.2	−123.7	−103.7	−111.3	−108.7	−116.7	−101.5	−109.0	−107.3	−115.1	−108.7	−116.7
Knerachelin B (**475**)	−94.7	−105.3	−110.2	−122.6	−98.8	−109.9	−100.0	−111.2	−98.5	−109.5	−102.4	−113.8	−102.7	−114.2
Magnolol (**476**)	−95.7	−106.9	−108.5	−121.3	−93.0	−103.9	−110.6	−123.6	−100.3	−112.1	−99.1	−110.7	−102.0	−114.0
Myrtucommulone A (**477**)	−84.9	−69.8	−115.2	−94.7	−90.7	−74.6	−100.5	−82.6	−121.8	−100.2	−102.6	−84.4	−126.7	−104.1
Myrtucommulone B (**478**)	−103.5	−99.8	−99.8	−96.2	−98.5	−94.9	−111.2	−107.2	−103.3	−99.6	−98.7	−95.2	−98.5	−94.9
Obovatol (**479**)	−89.7	−98.3	−105.7	−115.8	−99.3	−108.8	−104.8	−114.8	−96.6	−105.9	−91.2	−99.9	−97.5	−106.9
Oenostacin (**480**)	−95.4	−106.6	−104.8	−117.2	−94.8	−105.9	−107.6	−120.2	−105.2	−117.6	−101.7	−113.7	−104.0	−116.3
Paeonol (**481**)	−75.7	−99.0	−77.8	−101.8	−71.1	−93.0	−75.4	−98.6	−76.6	−100.1	−76.9	−100.5	−74.7	−97.6
Perlatolic acid (**482**)	−106.6	−100.4	−129.6	−122.1	−116.4	−109.7	−125.7	−118.4	−127.9	−120.5	−132.0	−124.3	−126.8	−119.4
Plicatipyrone (**483**)	−81.3	−78.2	−106.0	−101.9	−96.2	−92.5	−101.4	−97.4	−103.8	−99.8	−98.5	−94.7	−105.4	−101.3
Propterol (**484**)	−85.2	−98.0	−98.1	−112.8	−91.6	−105.3	−87.6	−100.8	−93.5	−107.5	−93.0	−107.0	−91.5	−105.2
Pulverulentone B (**485**)	−80.3	−93.1	−92.6	−107.3	−88.6	−102.8	−91.1	−105.7	−94.1	−109.2	−95.8	−111.1	−90.5	−104.9
Quinquangulin (**486**)	−78.6	−85.7	−86.3	−94.1	−83.1	−90.7	−81.4	−88.8	−86.6	−94.4	−86.7	−94.6	−84.3	−91.9
Rhodomyrtone (**487**)	−94.0	−88.7	−111.4	−105.1	−82.5	−77.9	−100.9	−95.2	−110.7	−104.4	−107.0	−101.0	−109.5	−103.3
Rosmarinic acid (**488**)	−99.0	−100.0	−129.8	−131.2	−111.5	−112.7	−129.9	−131.3	−107.3	−108.4	−106.3	−107.4	−119.8	−121.0
Rubanthrone A (**489**)	−102.1	−101.5	−105.3	−104.6	−108.4	−107.8	−101.3	−100.7	−107.5	−106.9	−105.8	−105.2	−105.1	−104.5
Sampsone A (**490**)	−81.4	−80.5	−98.2	−97.1	−98.5	−97.4	−99.2	−98.1	−97.5	−96.4	−91.8	−90.8	−101.1	−99.9
Sarothralen B (**491**)	−98.2	−85.3	−140.1	−121.7	−106.3	−92.3	−128.7	−111.9	−132.2	−114.9	−122.5	−106.5	−118.2	−102.7
Sarothralen C (**492**)	−93.5	−80.4	−161.4	−138.8	−71.2	−61.2	−127.8	−109.9	−122.5	−105.3	−127.7	−109.8	−128.3	−110.3
Sarothralen D (**493**)	−106.3	−91.4	−146.7	−126.1	−110.6	−95.1	−121.5	−104.5	−135.8	−116.8	−120.5	−103.6	−130.0	−111.7
Shikonofuran C (**494**)	−98.2	−99.3	−130.8	−132.4	−105.6	−106.9	−121.1	−122.6	−111.4	−112.8	−108.0	−109.3	−125.1	−126.6
Shikonofuran D (**495**)	−101.0	−103.6	−125.6	−128.8	−102.0	−104.6	−120.8	−123.9	−109.4	−112.2	−103.9	−106.6	−118.8	−121.9
Shikonofuran E (**496**)	−101.2	−102.6	−130.8	−132.7	−112.7	−114.3	−124.0	−125.8	−108.1	−109.6	−105.6	−107.1	−123.7	−125.5
Sinapic acid (**497**)	−83.4	−98.7	−89.8	−106.2	−84.9	−100.5	−87.2	−103.2	−84.3	−99.8	−84.6	−100.1	−87.3	−103.3
Walrycin A (**498**)	−68.1	−87.7	−73.6	−94.8	−66.9	−86.1	−73.9	−95.1	−71.4	−91.9	−71.5	−92.1	−70.3	−90.5
**Quinones**														
2,6-Dimethoxy-1,4-benzo-quinone (**336**)	−66.9	−87.2	−69.3	−90.2	−67.6	−88.1	−69.2	−90.2	−68.0	−88.5	−66.9	−87.1	−68.3	−89.0
2-Methyl-6-prenyl-1,4-benzo-quinone (**337**)	−80.4	−100.6	−83.4	−104.3	−78.6	−98.3	−84.0	−105.0	−82.9	−103.6	−81.0	−101.3	−85.9	−107.4
Omphalone (**499**)	−88.5	−111.1	−90.1	−113.0	−84.6	−106.1	−91.2	−114.4	−85.7	−107.5	−87.2	−109.5	−90.0	−113.0
Primin (**500**)	−82.1	−99.6	−91.4	−110.9	−80.5	−97.6	−88.5	−107.3	−88.3	−107.1	−87.2	−105.7	−93.6	−113.5
1,4-Naphthoquinone (**338**)	−63.6	−84.5	−67.6	−89.9	−61.4	−81.6	−68.0	−90.4	−64.1	−85.3	−65.4	−86.9	−65.4	−87.0
2-Acetylnaphtho[2,3-*b*]furan-4,9-dione (**339**)	−83.4	−96.5	−90.3	−104.5	−83.7	−96.8	−91.8	−106.2	−89.4	−103.4	−89.3	−103.3	−89.2	−103.2
Alkannin (**340**)	−88.7	−96.6	−104.5	−113.8	−93.5	−101.8	−104.8	−114.1	−95.9	−104.3	−92.8	−101.0	−99.1	−107.8
Isobutyrylshikonin (**341**)	−98.6	−99.8	−112.4	−113.7	−106.2	−107.5	−118.7	−120.2	−105.8	−107.1	−101.8	−103.0	−113.3	−114.6
Lapachol (**501**)	−83.9	−96.8	−91.8	−105.9	−84.5	−97.5	−91.8	−105.8	−88.8	−102.4	−85.4	−98.5	−92.6	−106.8
Mamegakinone (**502**)	−92.8	−92.6	−106.7	−106.5	−91.3	−91.1	−105.7	−105.4	−92.4	−92.2	−87.2	−87.0	−104.1	−103.8
Menadione (**503**)	−69.9	−90.4	−73.0	−94.4	−65.5	−84.7	−72.6	−93.8	−70.9	−91.7	−72.2	−93.3	−69.5	−89.8
Rhinacanthin C (**504**)	−114.9	−111.1	−128.5	−124.3	−112.3	−108.7	−122.9	−118.9	−114.2	−110.4	−114.3	−110.6	−116.1	−112.3
Rhinacanthin D (**505**)	−100.4	−97.3	−128.7	−124.7	−112.8	−109.3	−118.6	−114.9	−118.4	−114.7	−125.1	−121.2	−122.1	−118.3
Rhinacanthin G (**506**)	−115.2	−110.0	−136.0	−129.9	−116.2	−111.0	−123.7	−118.2	−122.9	−117.4	−123.3	−117.7	−118.1	−112.8
Rhinacanthin H (**507**)	−96.7	−92.4	−131.2	−125.3	−98.6	−94.2	−121.2	−115.8	−117.8	−112.6	−119.6	−114.3	−124.3	−118.8
Rhinacanthin I (**508**)	−116.4	−111.2	−130.9	−125.0	−109.9	−105.0	−124.2	−118.6	−128.3	−122.5	−121.9	−116.5	−133.2	−127.2
Rhinacanthin J (**509**)	−124.5	−119.1	−126.5	−121.0	−112.4	−107.6	−127.9	−122.3	−121.1	−115.9	−124.6	−119.2	−119.5	−114.3
Rhinacanthin K (**510**)	−98.9	−93.2	−130.3	−122.8	−121.4	−114.3	−117.3	−110.5	−123.0	−115.9	−118.7	−111.8	−119.1	−112.2
Rhinacanthin L (**511**)	−105.6	−98.3	−134.8	−125.5	−110.6	−103.0	−118.2	−110.1	−118.3	−110.1	−116.8	−108.8	−120.0	−111.7
Rhinacanthin M (**512**)	−110.0	−110.8	−117.2	−118.0	−99.6	−100.3	−110.8	−111.5	−105.3	−106.0	−105.4	−106.1	−115.7	−116.4
Shikonin acetate (**513**)	−95.0	−98.9	−109.3	−113.7	−101.2	−105.3	−114.2	−118.8	−103.7	−107.9	−98.8	−102.8	−108.8	−113.2
β,β-Dimethylacrylshikonin (**514**)	−100.5	−100.6	−121.3	−121.5	−107.0	−107.1	−125.7	−125.9	−109.9	−110.0	−107.7	−107.8	−121.4	−121.5
β-Hydroxyisovaleryshikonin (**515**)	−103.2	−101.7	−126.2	−124.3	−106.6	−105.0	−120.7	−118.9	−110.3	−108.7	−105.1	−103.5	−120.0	−118.3
1-Hydroxy-3-hydroxymethyl-anthraquinone (**516**)	−84.8	−96.2	−93.8	−106.4	−85.2	−96.7	−92.1	−104.5	−96.1	−109.0	−93.7	−106.3	−88.5	−100.4
Aloeemodin (**518**)	−85.6	−95.2	−94.6	−105.2	−88.3	−98.2	−97.4	−108.3	−96.3	−107.1	−95.7	−106.4	−86.9	−96.7
Islandicin (**519**)	−77.5	−86.2	−91.0	−101.2	−84.9	−94.4	−92.9	−103.3	−90.8	−101.0	−93.8	−104.3	−79.3	−88.2
Newbouldiaquinone (**521**)	−82.2	−80.6	−101.6	−99.6	−99.3	−97.4	−93.5	−91.7	−93.8	−92.0	−93.9	−92.1	−95.0	−93.2
Newbouldiaquinone A (**520**)	−105.6	−102.2	−112.2	−108.5	−92.5	−89.5	−105.8	−102.3	−96.7	−93.5	−104.1	−100.7	−114.3	−110.6
Rhein (**522**)	−90.4	−98.9	−100.6	−110.0	−88.7	−97.0	−100.2	−109.6	−96.1	−105.1	−99.4	−108.7	−90.6	−99.0
15,16-Dihydrotanshinone I (**517**)	−81.1	−89.4	−90.7	−99.9	−83.8	−92.3	−89.0	−98.0	−86.3	−95.0	−85.4	−94.0	−86.3	−95.0
**Acetylene, Glucoside, and Other Miscellaneous Phytochemicals**														
1,7-Diphenyl-4-(2-phenylethyl)-1-heptene-3,5-dione (**530**)	−99.6	−98.6	−127.4	−126.1	−115.0	−113.9	−114.4	−113.3	−127.2	−126.0	−114.1	−113.0	−130.7	−129.5
1,7-Diphenyl-5-hepten-3-one (**531**)	−96.6	−108.2	−114.5	−128.2	−98.4	−110.3	−107.9	−120.9	−100.1	−112.1	−95.1	−106.6	−108.0	−120.9
3′-Demothexycyclocurcumin (**532**)	−96.6	−99.7	−112.7	−116.3	−97.1	−100.2	−103.9	−107.2	−113.7	−117.3	−113.3	−116.9	−110.4	−114.0
5,7-Dihydroxyphthalide (**533**)	−68.6	−89.7	−71.2	−93.1	−66.0	−86.4	−70.8	−92.6	−70.4	−92.1	−69.7	−91.1	−72.9	−95.3
6-Methyl-4,5-dithia-2-octene (**534**)	−65.9	−86.8	−65.5	−86.4	−63.6	−83.8	−65.0	−85.7	−64.6	−85.1	−64.8	−85.4	−65.9	−86.8
7-Epiclusianone (**535**)	−100.2	−90.6	−126.3	−114.2	−115.1	−104.1	−125.9	−113.9	−113.0	−102.1	−111.1	−100.5	−127.8	−115.6
Allamandin (**536**)	−82.3	−87.6	−94.9	−101.0	−96.5	−102.7	−86.3	−91.9	−99.6	−106.0	−93.4	−99.4	−88.8	−94.5
Allicin (**537**)	−65.3	−86.1	−65.4	−86.3	−62.6	−82.6	−64.1	−84.5	−64.8	−85.5	−62.9	−83.0	−66.7	−87.9
Amadannulen (**538**)	−92.0	−91.6	−112.5	−112.1	−98.4	−97.9	−111.5	−111.0	−114.0	−113.5	−108.4	−108.0	−111.7	−111.2
Anemonin (**539**)	−72.0	−89.7	−73.9	−92.0	−70.4	−87.7	−76.7	−95.6	−78.2	−97.4	−77.0	−95.9	−81.8	−102.0
Antibiotic CZ 34 (**540**)	−68.6	−81.7	−84.8	−100.9	−89.3	−106.3	−80.7	−96.1	−82.0	−97.7	−78.6	−93.6	−82.5	−98.2
Argutone (**541**)	−75.4	−95.0	−78.9	−99.4	−72.2	−90.9	−74.9	−94.4	−74.0	−93.2	−74.6	−93.9	−76.3	−96.1
Bakuchiol (**542**)	−87.3	−98.8	−106.6	−120.7	−92.0	−104.1	−98.3	−111.3	−99.6	−112.7	−91.8	−103.9	−97.5	−110.4
Brasiliensophyllic acid A (**543**)	−112.9	−98.4	−139.3	−121.5	−123.7	−107.8	−130.4	−113.7	−118.5	−103.3	−118.9	−103.7	−128.9	−112.4
Brasiliensophyllic acid C (**544**)	−114.6	−99.1	−130.8	−113.1	−121.7	−105.3	−137.5	−118.9	−122.0	−105.5	−127.2	−110.0	−126.8	−109.6
Centrolobin (**545**)	−96.7	−102.5	−106.5	−112.8	−102.0	−108.1	−103.4	−109.6	−108.1	−114.6	−102.6	−108.8	−101.9	−108.0
Chamone I (**546**)	−102.5	−88.8	−125.8	−109.0	−110.1	−95.4	−117.3	−101.7	−113.2	−98.1	−115.6	−100.2	−133.6	−115.8
Chamone II (**547**)	−98.9	−85.8	−132.4	−114.9	−115.7	−100.4	−115.7	−100.4	−116.8	−101.3	−110.5	−95.9	−130.6	−113.3
Champanone A (**548**)	−84.1	−95.2	−102.1	−115.5	−85.6	−96.9	−99.5	−112.7	−93.2	−105.5	−92.8	−105.0	−101.7	−115.2
Dhelwangin (**549**)	−87.3	−103.4	−89.4	−105.8	−83.4	−98.7	−86.6	−102.5	−90.7	−107.4	−88.9	−105.2	−89.5	−105.9
Garcinoic acid (**550**)	−108.9	−104.0	−134.6	−128.5	−113.4	−108.3	−129.4	−123.6	−135.3	−129.2	−127.3	−121.5	−132.0	−126.0
Ginkgolide A (**551**)	−69.1	−66.9	−100.9	−97.8	−90.7	−87.9	−74.5	−72.2	−97.6	−94.6	−96.3	−93.3	−90.3	−87.5
Guttiferone E (**552**)	−112.9	−96.1	−160.7	−136.8	−120.6	−102.6	−129.5	−110.3	−130.2	−110.8	−129.9	−110.6	−135.0	−114.9
Helipyrone B (**553**)	−89.4	−95.4	−99.0	−105.5	−89.2	−95.2	−99.3	−105.9	−92.5	−98.6	−89.7	−95.7	−91.1	−97.1
Helipyrone C (**554**)	−84.6	−91.7	−94.2	−102.1	−84.4	−91.4	−94.5	−102.4	−88.7	−96.1	−88.9	−96.3	−88.7	−96.1
Ialibinone A (**555**)	−87.2	−89.4	−97.5	−100.0	−96.2	−98.7	−94.8	−97.2	−99.2	−101.7	−97.9	−100.4	−101.4	−104.0
Ialibinone B (**556**)	−81.9	−84.0	−96.7	−99.2	−85.0	−87.2	−88.1	−90.4	−94.3	−96.8	−91.7	−94.1	−95.5	−97.9
Ialibinone C (**557**)	−86.9	−88.0	−99.4	−100.6	−98.4	−99.6	−96.9	−98.1	−103.1	−104.3	−97.1	−98.2	−100.4	−101.6
Ialibinone D (**558**)	−84.0	−85.0	−93.3	−94.4	−83.5	−84.5	−93.5	−94.7	−93.5	−94.6	−98.7	−99.9	−100.7	−102.0
Isobrasiliensophyllic acid A (**559**)	−99.1	−86.4	−120.1	−104.7	−122.1	−106.5	−122.0	−106.4	−102.3	−89.2	−119.5	−104.2	−110.8	−96.6
Moskachan C (**560**)	−87.4	−103.8	−90.9	−107.9	−87.2	−103.4	−92.3	−109.6	−94.8	−112.5	−91.3	−108.4	−91.3	−108.4
Nimbolide (**561**)	−107.4	−99.5	−123.1	−114.1	−95.1	−88.2	−113.7	−105.4	−125.7	−116.6	−119.8	−111.0	−111.7	−103.5
Pectinolide H (**562**)	−98.6	−109.9	−103.0	−114.8	−98.8	−110.1	−106.4	−118.6	−108.0	−120.3	−102.7	−114.5	−106.3	−118.5
Propolone A (**563**)	−98.9	−89.5	−127.3	−115.1	−108.3	−97.9	−106.5	−96.3	−115.3	−104.3	−102.1	−92.3	−120.4	−108.8
Sellovicine B (**564**)	−82.3	−95.9	−93.2	−108.6	−84.1	−98.1	−92.2	−107.5	−86.0	−100.3	−89.2	−104.0	−87.1	−101.6
Simonin A (**565**)	−84.4	−81.2	−109.6	−105.6	−104.3	−100.4	−105.6	−101.6	−117.0	−112.7	−106.3	−102.4	−111.2	−107.1
Tenulin (**566**)	−83.0	−88.5	−89.7	−95.7	−81.9	−87.3	−89.9	−95.9	−96.6	−103.0	−91.1	−97.2	−93.2	−99.4
Atractylodin (**522**)	−66.5	−84.3	−86.2	−109.3	−76.8	−97.4	−78.0	−98.9	−76.6	−97.2	−78.8	−100.0	−84.0	−106.5
Atractylodinol (**523**)	−75.1	−92.6	−90.3	−111.4	−83.6	−103.1	−82.7	−101.9	−81.2	−100.1	−87.5	−107.8	−89.7	−110.6
Capillene (**342**)	−65.5	−87.8	−71.6	−96.0	−69.3	−92.9	−70.8	−94.9	−72.6	−97.4	−74.9	−100.4	−72.1	−96.7
Peniophorin A (**524**)	−101.4	−111.1	−106.8	−117.1	−96.7	−105.9	−103.7	−113.6	−111.0	−121.6	−109.1	−119.5	−107.7	−118.0
Peniophorin B (**525**)	−84.8	−102.2	−93.2	−112.3	−88.4	−106.6	−94.5	−113.9	−87.2	−105.1	−85.5	−103.0	−89.8	−108.3
Thiarubrin A (**526**)	−70.3	−82.7	−85.5	−100.6	−77.6	−91.2	−76.4	−89.8	−73.7	−86.7	−77.2	−90.8	−74.7	−87.9
Arbutin (**527**)	−90.0	−99.8	−92.0	−102.0	−91.6	−101.7	−94.8	−105.2	−91.9	−101.9	−93.8	−104.1	−88.0	−97.6
Aucubin (**528**)	−99.3	−101.7	−104.7	−107.2	−103.7	−106.2	−111.0	−113.6	−101.2	−103.6	−94.7	−97.0	−103.6	−106.1
Diospyrodin (**529**)	−88.6	−93.7	−97.9	−103.5	−97.4	−103.1	−96.3	−101.8	−95.0	−100.5	−90.4	−95.6	−96.6	−102.2
**Synthetic Inhibitors**														
06-1467 [23] (**567**)	−111.0	−114.0	−122.7	−125.9	−113.7	−116.8	−129.8	−133.2	−107.4	−110.3	−109.5	−112.4	-	-
64-1811 [23] (**569**)	−84.3	−99.3	−86.3	−101.7	−80.8	−95.2	−84.5	−99.6	−83.5	−98.4	−84.5	−99.5	-	-
66-6976 [23] (**568**)	−91.1	−101.5	−101.4	−112.9	−92.8	−103.3	−91.1	−101.4	−99.1	−110.4	−97.6	−108.7	-	-
*N*-Hydroxy-2-(5-methylsulfanyl-1*H*-indol-3-yl)acetamide [86] (**570**)	−89.4	−104.0	−101.6	−118.2	−80.5	−93.7	−91.9	−106.8	−95.7	−111.3	−88.9	−103.4	-	-
2-(1-Benzyl-5-bromoindol-3-yl)-*N*-hydroxyacetamide [86] (**572**)	−105.8	−107.0	−112.6	−113.9	−106.8	−108.0	−106.9	−108.2	−104.4	−105.6	−104.9	−106.1	-	-
2-(3-Benzyl-5-bromoindol-1-yl)-*N*-hydroxyacetamide [86] (**571**)	−101.2	−102.3	−117.5	−118.9	−94.5	−95.6	−110.2	−111.4	−101.7	−102.9	−103.4	−104.5	-	-

^a^ Compounds shown in red font violate Lipinski’s rule-of-five [62].

**Table 3:** MolDock molecular docking energies (E_dock_, kJ/mol) and normalized docking scores (DS_norm_) for antibacterial phytochemical ligands with bacterial topoisomerases and bacterial protein tyrosine phosphatase.

Ligand	EcTopoIV		EcGyrB		MtGyrB		MtPtp		HsPtp	
	E_dock_	DS_norm_	E_dock_	DS_norm_	E_dock_	DS_norm_	E_dock_	DS_norm_	E_dock_	DS_norm_
**Indole Alkaloids**										
1-Hydroxy-6,7-dimethoxy-3-methylcarbazole (**1**)	−96.8	−109.5	−86.9	−98.2	−94.7	−107.0	−82.8	−93.7	−86.9	−98.2
11-Methoxytubotaiwine (**2**)	−75.7	−76.9	−95.3	−96.8	−89.7	−91.2	−92.5	−94.0	−79.2	−80.5
12-Methoxy-4-methylvoachalotine (**3**)	−94.0	−90.9	−76.4	−73.8	−80.1	−77.5	−98.9	−95.6	−38.4	−37.1
3-Prenylindole (**4**)	−83.9	−105.9	−81.5	−102.8	−89.2	−112.5	−75.1	−94.7	−78.6	−99.1
Affinisine (**5**)	−102.0	−108.5	−76.4	−81.3	−103.2	−109.8	−82.9	−88.2	−74.1	−78.8
Apparicine (**6**)	−89.6	−100.3	−80.7	−90.4	−84.7	−94.9	−83.6	−93.6	−70.4	−78.9
Aristolactam I (**7**)	−103.7	−112.2	−93.6	−101.3	−100.5	−108.8	−93.8	−101.5	−88.4	−95.6
Clausenawalline A (**8**) ^a^	−145.2	−126.9	−86.8	−75.9	−114.5	−100.1	−103.2	−90.2	−60.2	−52.6
Cryptoheptine (**9**)	−88.8	−99.7	−72.6	−81.5	−91.4	−102.6	−75.6	−84.9	−92.6	−104.1
Diploceline (**10**)	−105.2	−105.4	−82.8	−83.0	−97.7	−97.9	−90.2	−90.4	−50.8	−50.9
Discarine B (**11**)	−49.3	−42.6	−103.6	−89.7	−118.8	−102.8	−130.1	−112.6	−44.8	−38.8
Ibogamine (**12**)	−85.6	−94.0	−76.6	−84.1	−85.8	−94.2	−78.7	−86.5	−83.1	−91.2
Iboxygaine (**13**)	−91.8	−95.9	−76.2	−79.6	−94.5	−98.7	−81.4	−85.0	−83.7	−87.4
Isovoacangine (**14**)	−98.8	−99.1	−87.3	−87.5	−94.6	−94.9	−94.2	−94.4	−81.2	−81.4
Rugosanine B (**15**)	−118.2	−99.6	−93.1	−78.4	−64.4	−54.3	−137.2	−115.5	−91.8	−77.3
Suaveolindole (**16**)	−111.9	−113.8	−101.6	−103.3	−106.5	−108.3	−98.8	−100.5	−99.0	−100.6
Toussaintine B (**17**)	−99.8	−107.2	−85.1	−91.4	−99.9	−107.4	−87.5	−94.1	−85.4	−91.8
**Isoquinoline Alkaloids**										
8-Acetonyldihydroavicine (**18**)	−117.3	−113.9	−102.1	−99.2	−115.4	−112.1	−111.7	−108.5	−89.6	−87.0
8-Acetonyldihydronitidine (**19**)	−114.9	−113.1	−88.0	−86.7	−113.8	−112.0	−95.3	−93.8	−86.9	−85.6
Antofine (**20**)	−116.6	−117.4	−95.8	−96.5	−114.3	−115.2	−87.6	−88.3	−83.5	−84.1
Berbamine (**24**)	no dock	no dock	−59.9	−50.9	no dock	no dock	−106.8	−90.6	−57.1	−48.5
Berberine (**21**)	−117.4	−121.4	−98.5	−101.9	−109.1	−112.8	−83.6	−86.4	−44.3	−45.8
Bisnorthalphenine (**22**)	−103.0	−107.9	−84.2	−88.2	−102.3	−107.2	−85.5	−89.6	−72.6	−76.1
Cepharanthine (**25**)	no dock	no dock	−91.9	−78.1	no dock	no dock	−130.5	−110.8	−62.9	−53.4
Cryptopleurine (**23**)	−118.7	−118.1	−88.5	−88.1	−113.6	−113.0	−86.4	−86.0	−85.3	−84.8
Emetine (**26**)	−127.8	−117.3	−92.3	−84.7	−119.6	−109.8	−106.9	−98.1	−75.6	−69.4
Hydrastine (**27**)	−127.0	−125.7	−97.5	−96.5	−124.8	−123.5	−96.8	−95.8	−76.1	−75.3
Isotrilobine (**29**)	−77.7	−67.1	−72.7	−62.8	−45.7	−39.5	−121.6	−105.0	−56.9	−49.1
Jatrorrhizine (**28**)	−110.6	−114.1	−97.1	−100.1	−107.4	−110.8	−79.2	−81.7	−60.3	−62.2
Lauroscholtzine (**31**)	−104.5	−107.5	−80.3	−82.6	−106.8	−109.9	−83.0	−85.4	−66.5	−68.4
Methothalistyline (**30**)	−17.3	−13.9	−70.6	−56.8	no dock	no dock	−123.1	−99.1	−50.7	−40.8
*N*-Demethylthalphenine (**32**)	−105.1	−108.6	−83.2	−85.9	−102.2	−105.6	−80.5	−83.2	−42.3	−43.7
Obamegine (**34**)	no dock	no dock	−60.6	−51.8	no dock	no dock	−105.8	−90.5	−59.9	−51.2
Oxyacanthine (**35**)	−42.8	−36.3	−65.6	−55.7	no dock	no dock	−110.2	−93.5	−54.6	−46.3
Pennsylvanine (**36**)	−145.7	−119.0	−113.8	−92.9	no dock	no dock	−116.7	−95.3	−81.9	−66.8
Thaliadanine (**38**)	−127.9	−102.9	−116.0	−93.2	−57.1	−45.9	−131.9	−106.0	−82.4	−66.3
Thalicarpine (**37**)	−141.3	−114.6	−89.6	−72.7	−14.4	−11.6	−131.6	−106.8	−45.1	−36.6
Thalidasine (**39**)	no dock	no dock	−73.1	−60.6	no dock	no dock	−98.4	−81.5	−47.3	−39.2
Thalistyline (**40**)	−74.8	−60.6	−98.4	−79.7	no dock	no dock	−114.6	−92.9	−77.3	−62.6
Thalmelatine (**41**)	−142.7	−116.5	−97.6	−79.7	−45.6	−37.3	−110.6	−90.3	−91.4	−74.6
Thalmirabine (**42**)	no dock	no dock	−93.9	−77.2	no dock	no dock	−97.7	−80.3	−48.5	−39.9
Thalphenine (**33**)	−105.2	−107.1	−80.6	−82.1	−100.3	−102.1	−83.6	−85.1	−48.2	−49.1
Thalrugosidine (**43**)	no dock	no dock	−84.6	−70.6	no dock	no dock	−92.2	−77.0	−58.8	−49.1
Thalrugosine (**44**)	no dock	no dock	−57.5	−48.7	no dock	no dock	−102.8	−87.2	−59.7	−50.7
**Piperidine, Pyrrole, Pyrrolizidine, Quinoline, and Steroidal Alkaloids**										
Aconicaramide (**46**)	−98.8	−117.3	−83.8	−99.5	−101.1	−120.1	−78.1	−92.7	−91.8	−108.9
Lasiocarpine (**47**)	−117.0	−113.1	−92.3	−89.2	−121.7	−117.6	−105.2	−101.7	−87.4	−84.5
Lasiocarpine *N*-oxide (**48**)	−120.3	−114.8	−79.4	−75.8	−123.5	−117.9	−102.5	−97.9	−71.1	−67.9
Piperine (**45**)	−109.9	−120.0	−93.3	−101.9	−100.9	−110.2	−86.5	−94.5	−89.3	−97.6
4-Methoxy-1-methyl-2(1*H*)-quinolinone (**49**)	−76.4	−95.7	−65.4	−81.9	−77.4	−97.0	−61.3	−76.8	−64.6	−80.9
Cryptolepine (**50**)	−91.9	−107.4	−79.4	−92.8	−83.7	−97.9	−77.5	−90.7	−81.9	−95.8
Neocryptolepine (**51**)	−90.7	−106.1	−80.9	−94.6	−83.6	−97.8	−68.2	−79.8	−81.0	−94.7
Pteleine (**52**)	−98.4	−115.6	−82.2	−96.6	−93.1	−109.4	−73.4	−86.3	−79.9	−93.9
Veprisinium (**53**)	−101.5	−105.2	−56.8	−58.9	−97.3	−100.8	−81.9	−84.8	−74.0	−76.7
Conessine (**54**)	−100.0	−101.4	−70.6	−71.5	−90.6	−91.9	−84.6	−85.8	−58.1	−58.9
Irehdiamine A (**55**)	−102.9	−108.6	−86.4	−91.2	−99.9	−105.4	−78.9	−83.3	−25.3	−26.7
Solacassine (**56**)	−89.4	−84.3	−28.5	−26.9	−65.6	−61.9	−87.3	−82.4	−83.2	−78.5
Solanocapsine (**57**)	−95.8	−91.2	−74.5	−70.9	−85.6	−81.5	−83.0	−79.1	−52.8	−50.3
Tomatidine (**58**)	−101.5	−97.8	−70.7	−68.1	−89.2	−86.0	−63.6	−61.3	−61.9	−59.7
**Miscellaneous Alkaloids**										
2-(Methoxyamino)-4*H*-1-benzopyran-3,4,5,7-tetrol (**59**)	−87.0	−100.5	−75.4	−87.0	−89.0	−102.8	−86.9	−100.4	−86.0	−99.4
Abyssenine C (**60**)	−126.9	−119.6	−93.9	−88.5	−99.7	−93.9	−106.0	−99.8	−64.9	−61.2
Amphibine H (**61**)	−95.6	−81.2	−66.1	−56.2	−66.7	−56.7	−132.3	−112.4	−99.4	−84.5
Cepharatine A (**62**)	−88.6	−93.8	−78.0	−82.6	−89.8	−95.0	−75.9	−80.3	−68.5	−72.5
Curcamide (**63**)	−107.4	−123.2	−99.4	−114.0	−107.0	−122.8	−88.9	−102.0	−89.9	−103.2
Drodrenin (**64**)	−154.5	−139.4	−128.6	−116.1	−156.5	−141.2	−120.7	−109.0	−102.5	−92.5
Eschscholtzidine (**65**)	−117.3	−120.9	−81.7	−84.2	−108.4	−111.7	−89.9	−92.7	−83.8	−86.4
Jervine (**66**)	−88.0	−84.1	−95.1	−90.9	−49.8	−47.6	−90.7	−86.7	−31.3	−29.9
Matrine (**67**)	−87.0	−99.5	−76.3	−87.3	−81.9	−93.7	−73.8	−84.4	−75.9	−86.8
Mucronine H (**68**)	−99.1	−91.1	−114.9	−105.6	−98.2	−90.3	−108.2	−99.5	−61.7	−56.7
*N*-Benzoylmescaline (**69**)	−120.9	−127.7	−107.5	−113.6	−122.1	−129.0	−94.6	−99.9	−85.5	−90.4
Nummularine B (**70**)	−83.6	−71.6	−123.6	−105.9	−62.9	−53.9	−133.1	−114.0	−103.8	−88.9
Nummularine S (**71**)	−116.4	−104.1	−111.3	−99.4	−139.9	−125.1	−109.6	−97.9	−95.8	−85.6
Scutianine B (**72**)	−133.8	−116.1	−103.3	−89.6	−55.9	−48.5	−130.4	−113.2	−96.9	−84.1
Shahidine (**73**)	−115.2	−126.7	−94.7	−104.2	−119.6	−131.6	−87.7	−96.5	−88.2	−97.0
Thaliglucinone (**74**)	−118.3	−119.0	−116.5	−117.2	−119.9	−120.5	−98.3	−98.9	−83.1	−83.6
Triisopenylguanidine (**75**)	−109.6	−122.0	−100.4	−111.7	−105.1	−117.0	−89.1	−99.2	−91.9	−102.3
Tuberine (**76**)	−126.0	−116.6	−112.6	−104.1	−114.3	−105.8	−109.6	−101.4	−80.2	−74.2
**Monoterpenoids**										
Linalool (**77**)	−75.4	−101.1	−70.3	−94.3	−74.4	−99.8	−66.2	−88.7	−77.1	−103.3
Thymol (**78**)	−70.3	−95.1	−64.1	−86.7	−69.5	−94.0	−58.9	−79.7	−65.0	−88.0
Thymoquinol (**79**)	−75.1	−98.2	−66.1	−86.5	−73.9	−96.7	−67.7	−88.6	−67.1	−87.7
β-Dolabrin (**80**)	−74.8	−98.7	−66.0	−87.0	−81.8	−107.9	−70.5	−92.9	−78.1	−103.0
β-Thujaplicin (**81**)	−80.3	−105.5	−63.2	−82.9	−81.2	−106.6	−72.0	−94.5	−76.9	−101.0
**Sesquiterpenoids**										
11,13-Dehydroeriolin (**82**)	−83.6	−93.7	−72.6	−81.3	−88.5	−99.1	−79.4	−89.0	−70.7	−79.2
2,10-Bisaboladien-1-one (**83**)	−87.3	−104.0	−91.8	−109.3	−89.1	−106.1	−78.7	−93.6	−78.0	−92.8
2-Hydroxycalamenene (**84**)	−73.5	−87.8	−68.8	−82.2	−68.3	−81.6	−71.6	−85.5	−64.3	−76.7
2-Methoxyfurano-9-guaien-8-one (**85**)	−92.6	−104.2	−86.5	−97.4	−88.3	−99.4	−82.9	−93.3	−79.4	−89.5
4α,10α-Dihydroxy-1,11(13)guaiadien-12,8-olide (**93**)	−86.6	−97.1	−79.9	−89.6	−86.1	−96.5	−80.0	−89.6	−63.9	−71.6
4α,10β-Dihydroxy-1,11(13)guaiadien-12,8-olide (**89**)	−42.4	−47.5	−79.9	−89.5	−75.9	−85.0	−84.0	−94.1	−67.5	−75.6
Alantolactone (**86**)	−80.0	−93.6	−67.5	−78.9	−80.6	−94.3	−71.5	−83.6	−54.3	−63.6
Alliacol A (**87**)	−76.6	−85.8	−72.3	−81.0	−79.6	−89.2	−73.9	−82.8	−56.6	−63.4
Alliacol B (**88**)	−81.2	−91.0	−73.9	−82.8	−86.6	−97.0	−78.0	−87.4	−55.4	−62.0
Artemisinic acid (**113**)	−100.5	−117.1	−75.6	−88.1	−105.8	−123.4	−79.4	−92.6	−76.5	−89.2
Baileyolin (**90**)	−104.9	−105.8	−96.9	−97.7	−90.2	−90.9	−84.1	−84.9	−86.1	−86.9
Bilobalide A (**91**)	−89.9	−93.9	−83.7	−87.4	−82.1	−85.7	−87.6	−91.5	−62.7	−65.5
Confertin (**92**)	−89.3	−102.1	−83.6	−95.7	−96.2	−110.0	−76.3	−87.3	−68.3	−78.1
Cyperenal (**94**)	−59.6	−71.2	−61.1	−72.9	−64.6	−77.1	−63.5	−75.8	−45.2	−54.0
Cyperenol (**95**)	−62.2	−74.1	−61.8	−73.5	−63.5	−75.6	−65.3	−77.7	−47.9	−57.0
Furanodienone (**97**)	−86.5	−101.4	−80.0	−93.9	−85.0	−99.7	−81.0	−95.0	−67.6	−79.2
Ganodermycin (**96**)	−109.3	−120.1	−93.1	−102.3	−111.4	−122.4	−106.3	−116.7	−104.1	−114.4
Helenalin (**98**)	−83.6	−93.9	−75.7	−85.0	−87.3	−98.0	−76.9	−86.4	−67.5	−75.8
Hydrogrammic acid (**99**)	−93.0	−104.7	−77.9	−87.7	−85.0	−95.8	−88.9	−100.1	−51.5	−58.0
Isoalantolactone (**100**)	−73.3	−85.7	−62.6	−73.3	−78.9	−92.3	−70.7	−82.7	−53.3	−62.3
Ivaxillin (**101**)	−83.2	−93.0	−77.4	−86.5	−88.7	−99.2	−79.9	−89.3	−53.1	−59.3
Petrovin A (**102**)	−78.8	−90.1	−78.0	−89.3	−76.8	−87.9	−79.6	−91.0	−54.1	−61.9
Petrovin B (**103**)	−81.7	−93.3	−81.4	−92.9	−96.3	−109.8	−74.4	−84.9	−82.9	−94.6
Polygodial (**104**)	−83.2	−97.0	−79.9	−93.2	−84.0	−98.0	−73.7	−86.0	−22.2	−25.9
Rishitin (**105**)	−81.4	−96.6	−72.7	−86.3	−82.1	−97.5	−76.5	−90.8	−83.0	−98.5
Xanthorrhizol (**106**)	−85.4	−102.0	−87.4	−104.4	−85.6	−102.2	−81.7	−97.6	−82.4	−98.3
α-Amorphene (**107**)	−85.8	−104.8	−66.2	−80.8	−82.0	−100.1	−66.2	−80.9	−50.6	−61.8
α-Cadinene (**108**)	−74.0	−90.4	−68.0	−83.0	−68.2	−83.2	−66.4	−81.1	−76.0	−92.8
α-Copaene (**110**)	−70.2	−87.7	−59.9	−74.9	−70.3	−87.8	−65.8	−82.2	−52.9	−66.1
α-Muurolene (**109**)	−77.1	−94.1	−69.5	−84.9	−71.0	−86.7	−71.7	−87.6	−65.5	−80.0
γ-Cadinene (**112**)	−77.7	−94.8	−67.1	−82.0	−80.6	−98.4	−63.8	−77.9	−59.0	−72.1
**Diterpenoids**										
1,12-Diacetyljativatriol (**114**)	−76.8	−74.6	−82.2	−79.9	−64.7	−62.9	−98.0	−95.3	−36.1	−35.1
12-Oxo-3,13(16)-clerodadien-15-oic acid (**115**)	−97.1	−102.2	−104.6	−110.1	−103.2	−108.7	−93.7	−98.7	−86.1	−90.6
12-Oxo-8,13(16)-clerodadien-15-oic acid (**116**)	−108.4	−114.1	−99.0	−104.2	−104.1	−109.6	−98.8	−104.1	−82.1	−86.4
13-Epimanoyl oxide (**117**)	−71.9	−78.0	−67.1	−72.8	−81.1	−88.0	−72.9	−79.1	−43.0	−46.7
13-Episclareol (**118**)	−92.2	−98.1	−94.2	−100.2	−87.7	−93.3	−87.4	−93.0	−61.2	−65.1
3,4-Seco-4(18)-trachyloben-3-oic acid (**120**)	−87.4	−93.7	−75.1	−80.5	−86.4	−92.5	−79.6	−85.3	−66.5	−71.3
3-Hydroxytotarol (**119**)	−90.5	−96.9	−75.9	−81.3	−87.6	−93.9	−81.5	−87.3	−73.6	−78.8
7,13-Labdadien-15-ol acetate (**121**)	−106.6	−110.6	−94.9	−98.4	−111.9	−116.1	−97.3	−101.0	−86.2	−89.5
7,13-Labdadien-15-ol malonate (**122**)	−122.6	−122.1	−88.7	−88.4	−115.6	−115.1	−106.2	−105.8	−82.4	−82.1
Acetylcrinipellin A (**125**)	−105.4	−105.4	−72.1	−72.0	−74.1	−74.1	−96.4	−96.3	−50.4	−50.4
Aethiopinone (**123**)	−101.0	−108.9	−96.9	−104.5	−106.0	−114.3	−82.1	−88.6	−86.2	−92.9
Andrographolide (**124**)	−100.6	−102.6	−90.9	−92.7	−110.8	−113.0	−98.4	−100.3	−97.9	−99.9
Biflorin (**126**)	−104.5	−113.0	−91.7	−99.1	−100.8	−108.9	−89.2	−96.4	−91.2	−98.6
Continentalic acid (**127**)	−77.1	−82.6	−53.9	−57.7	−85.3	−91.4	−81.8	−87.6	no dock	no dock
Crinipellin A (**129**)	−82.2	−85.5	−81.9	−85.1	−93.9	−97.6	−93.5	−97.2	−69.9	−72.7
Cryptobeilic acid A (**128**)	−118.0	−124.5	−98.9	−104.4	−114.2	−120.5	−97.6	−103.0	−65.6	−69.3
Cryptobeilic acid C (**130**)	−129.4	−130.3	−108.7	−109.5	−128.7	−129.5	−99.4	−100.1	−62.3	−62.8
Cryptobeilic acid D (**131**)	−120.3	−129.2	−100.7	−108.1	−122.0	−130.9	−99.2	−106.5	−89.2	−95.8
Effusanin A (**132**)	−49.0	−50.1	−73.9	−75.6	−47.5	−48.5	−80.5	−82.2	−39.7	−40.5
Effusanin B (**133**)	−73.0	−71.8	−58.5	−57.6	−40.4	−39.8	−84.6	−83.2	no dock	no dock
Effusanin C (**134**)	−64.2	−62.4	−77.6	−75.3	−33.6	−32.6	−95.8	−93.0	−51.2	−49.7
Effusanin D (**135**)	−75.2	−70.6	−73.7	−69.3	−63.7	−59.8	−101.7	−95.5	−51.9	−48.7
Effusanin E (**136**)	−46.4	−46.7	−69.0	−69.5	−31.3	−31.5	−78.0	−78.5	−18.3	−18.4
Grandiflorenic acid (**137**)	−60.0	−64.4	−59.6	−64.0	−73.7	−79.2	−73.4	−78.8	−35.3	−37.8
Haplociliatic acid (**138**)	−99.3	−102.7	−94.1	−97.2	−105.1	−108.6	−110.8	−114.5	−92.2	−95.3
Hypargenin A (**139**)	−84.2	−87.6	−89.5	−93.1	−84.5	−87.9	−80.0	−83.2	−75.5	−78.5
Hypargenin B (**140**)	−82.2	−86.7	−85.9	−90.7	−88.6	−93.4	−82.6	−87.2	−91.9	−96.9
Hypargenin D (**141**)	−91.4	−98.4	−94.0	−101.2	−91.0	−97.9	−77.5	−83.3	−26.9	−29.0
Hypargenin F (**142**)	−96.9	−100.7	−73.0	−76.0	−87.2	−90.7	−76.0	−79.0	−27.4	−28.5
Isodomedin (**143**)	−70.3	−69.1	−64.6	−63.5	−80.5	−79.0	−93.9	−92.2	no dock	no dock
Kamebanin (**144**)	−65.8	−68.1	−67.3	−69.7	−60.0	−62.1	−79.4	−82.2	no dock	no dock
Lasiokaurin (**145**)	−63.1	−61.1	−73.1	−70.8	−52.7	−51.0	−86.0	−83.4	no dock	no dock
Longikaurin A (**146**)	−56.8	−58.1	−56.4	−57.6	−61.1	−62.4	−76.1	−77.8	−36.8	−37.6
Longikaurin B (**147**)	−71.1	−69.0	−72.4	−70.3	−21.4	−20.7	−94.1	−91.3	−57.5	−55.8
Longikaurin C (**148**)	−67.0	−65.9	−76.5	−75.3	−22.4	−22.0	−96.8	−95.2	−51.2	−50.4
Longikaurin D (**149**)	−76.1	−73.8	−77.1	−74.8	−51.8	−50.3	−98.5	−95.6	−58.0	−56.3
Longikaurin E (**150**)	−61.6	−62.9	−62.6	−63.9	−41.2	−42.1	−77.2	−78.9	−53.5	−54.6
Longikaurin F (**151**)	−83.9	−78.8	−88.5	−83.1	−72.8	−68.4	−106.9	−100.4	−73.6	−69.1
Longikaurin G (**152**)	−64.3	−64.8	−49.3	−49.6	−27.9	−28.1	−76.3	−76.8	−17.5	−17.6
Lupulin E (**153**)	−104.1	−92.9	−64.9	−58.0	−94.7	−84.5	−117.7	−105.1	−83.1	−74.2
Lupulin F (**154**)	−75.2	−67.0	−75.4	−67.2	−93.3	−83.2	−117.1	−104.4	−71.0	−63.3
Methyl seconidoresedate (**155**)	−103.9	−108.2	−94.7	−98.7	−107.1	−111.6	−96.7	−100.8	−82.8	−86.3
Pisiferol (**156**)	−60.9	−65.3	−93.6	−100.2	−84.6	−90.6	−76.3	−81.7	−78.5	−84.0
Salvic acid (**157**)	−90.6	−94.9	−84.1	−88.1	−99.2	−104.0	−93.3	−97.9	−90.2	−94.6
Salvic acid acetate (**158**)	−107.8	−108.5	−85.4	−86.0	−113.4	−114.2	−94.9	−95.6	−87.9	−88.4
Shikokianin (**159**)	−72.3	−67.9	−68.7	−64.5	−12.7	−11.9	−95.0	−89.2	−15.9	−15.0
Strictic acid (**160**)	−85.4	−90.3	−75.1	−79.4	−99.8	−105.6	−108.1	−114.3	−67.3	−71.2
Taxodione (**161**)	−93.6	−99.0	−92.8	−98.1	−90.6	−95.8	−82.5	−87.3	−40.1	−42.4
Trichodonin (**162**)	−11.3	−11.0	−75.1	−73.0	−36.5	−35.5	−90.5	−88.0	−60.6	−59.0
Umbrosin A (**163**)	−57.6	−59.6	−68.5	−71.0	−59.6	−61.7	−77.0	−79.7	no dock	no dock
Umbrosin B (**164**)	−69.2	−71.8	−42.6	−44.2	−69.1	−71.7	−78.2	−81.1	no dock	no dock
Yuexiandajisu A (**165**)	−82.8	−87.2	−73.8	−77.8	−65.3	−68.7	−90.4	−95.2	−52.3	−55.1
**Triterpenoids**										
Alisol A 24-acetate (**166**)	−102.3	−90.7	−77.4	−68.6	−45.9	−40.7	−101.7	−90.2	−49.3	−43.7
Alisol B 23-acetate (**167**)	−82.2	−73.8	−102.3	−91.8	−80.5	−72.2	−117.7	−105.6	19.5	17.5
Betulinic acid (**168**)	−26.4	−24.7	−75.5	−70.5	−34.3	−32.1	−101.3	−94.5	−25.6	−23.9
Entagenic acid (**169**)	no dock	no dock	no dock	no dock	no dock	no dock	−99.2	−90.6	33.3	30.4
Lantic acid (**170**)	−21.3	−19.7	−76.7	−70.9	no dock	no dock	−89.7	−82.9	16.4	15.2
Mahmoodin (**171**)	no dock	no dock	−73.9	−65.8	no dock	no dock	−125.8	−112.0	−10.2	−9.1
Maslinic acid (**172**)	−59.5	−54.9	−63.1	−58.2	no dock	no dock	−105.2	−97.1	−32.2	−29.7
Oleanolic acid (**173**)	−60.7	−56.7	−79.9	−74.6	no dock	no dock	−100.3	−93.6	−14.1	−13.2
Pristimerin (**174**)	−35.5	−33.0	−81.1	−75.3	−25.4	−23.6	−82.3	−76.4	−54.2	−50.3
Rubrinol (**175**)	no dock	no dock	−84.3	−79.5	no dock	no dock	−99.2	−93.5	−28.9	−27.2
Tingenone (**176**)	−37.6	−36.0	−68.2	−65.5	−24.1	−23.1	−90.7	−87.1	−57.6	−55.2
**Chalcones**										
1-(2,6-Dihydroxy-4-methoxyphenyl)-3-phenyl-1-propanone (**177**)	−112.2	−124.4	−97.3	−107.9	−112.3	−124.6	−82.8	−91.9	−87.7	−97.3
2′-Hydroxy-2,3,4′,6′-tetramethoxychalcone (**178**)	−127.2	−130.4	−92.7	−95.0	−127.3	−130.6	−91.9	−94.3	−84.8	−87.0
3′′′′,5′′′,5′′′′′-Tribenzyl-2′′′′,2′′′′′,2′′′′′′-trihydroxyisodiuvaretin (**180**)	−84.3	−65.2	−120.6	−93.3	no dock	no dock	−127.4	−98.5	−105.3	−81.5
4′-Hydroxychalcone (**179**)	−92.5	−109.5	−85.8	−101.6	−92.5	−109.4	−82.7	−97.9	−87.9	−104.0
5″,5′′′′,5′′′′′-Tribenzyl-2′′′′,2′′′′′,2′′′′′′-trihydroxyisodiuvaretin (**181**)	−52.4	−40.5	−128.6	−99.5	no dock	no dock	−159.7	−123.5	−116.6	−90.2
Angusticornin B (**182**)	−154.9	−148.2	−79.5	−76.0	−151.8	−145.2	−127.3	−121.8	−97.1	−92.9
Balsacone A (**183**)	−144.0	−138.2	−107.4	−103.1	−143.1	−137.3	−113.8	−109.3	−107.6	−103.3
Balsacone B (**184**)	−139.9	−134.3	−113.4	−108.8	−144.0	−138.2	−116.0	−111.4	−107.8	−103.5
Balsacone C (**185**)	−132.9	−130.7	−107.2	−105.5	−133.6	−131.4	−112.5	−110.7	−109.0	−107.3
Bartericin C (**186**)	−118.5	−114.8	−66.2	−64.1	−104.8	−101.5	−112.8	−109.3	−68.4	−66.3
Bavachalcone (**187**)	−137.5	−141.9	−101.8	−105.1	−136.4	−140.8	−103.1	−106.4	−92.8	−95.8
Broussochalcone B (**188**)	−127.2	−133.1	−107.7	−112.7	−130.5	−136.5	−103.6	−108.4	−105.3	−110.2
Corylifol B (**189**)	−138.2	−142.3	−113.7	−117.0	−140.0	−144.2	−107.5	−110.7	−107.2	−110.4
Erythbidin C (**190**)	−122.4	−129.7	−96.7	−102.5	−121.2	−128.4	−102.6	−108.7	−102.7	−108.9
Helichrysone A (**191**)	−124.8	−126.8	−109.9	−111.7	−125.4	−127.4	−102.7	−104.3	−98.7	−100.3
Isobavachalcone (**192**)	−127.7	−133.7	−110.6	−115.7	−133.8	−140.0	−100.7	−105.3	−103.3	−108.1
Kanzonol C (**193**)	−151.6	−148.8	−119.0	−116.9	−159.8	−156.9	−111.8	−109.8	−88.1	−86.5
Kuraridin (**194**)	−148.2	−140.3	−122.1	−115.5	−145.9	−138.1	−121.3	−114.8	−104.2	−98.7
Myrigalone G (**195**)	−111.4	−121.5	−98.3	−107.3	−115.0	−125.5	−91.9	−100.3	−88.3	−96.3
Piperaduncin A (**196**)	−137.5	−125.3	−113.2	−103.2	−134.3	−122.5	−119.7	−109.1	−82.7	−75.4
Piperaduncin B (**197**)	−134.6	−121.4	−124.5	−112.3	−152.4	−137.5	−122.3	−110.3	−102.4	−92.4
Piperaduncin C (**198**)	−134.1	−117.2	−130.8	−114.3	−112.3	−98.1	−128.3	−112.1	−78.9	−68.9
Psorachalcone A (**199**)	−130.1	−134.0	−112.2	−115.6	−137.6	−141.7	−96.9	−99.8	−99.3	−102.2
Xanthoangelol (**200**)	−143.1	−140.5	−113.3	−111.2	−140.1	−137.6	−110.2	−108.2	−93.1	−91.4
Xanthoangelol F (**201**)	−147.0	−142.7	−123.4	−119.7	−133.1	−129.2	−116.5	−113.1	−101.2	−98.3
**Flavonoids**										
2′,5,5′,7-Tetrahydroxyflavanone (**202**)	−102.5	−111.6	−87.5	−95.2	−96.1	−104.6	−86.1	−93.7	−91.4	−99.5
2′,7-Dimethoxyflavone (**203**)	−105.9	−116.1	−90.8	−99.5	−103.5	−113.5	−79.5	−87.1	−81.0	−88.8
3′′′′-(2-Hydroxybenzyl)isouvarinol (**218**)	−158.3	−129.4	−157.9	−129.0	−115.6	−94.4	−130.8	−106.9	−126.0	−103.0
3′′′′-(2-Hydroxybenzyl)uvarinol (**217**)	−155.5	−127.1	−153.1	−125.1	−125.1	−102.2	−152.8	−124.9	−93.3	−76.3
3′-Methylpelargonidin (**204**)	−97.6	−106.6	−88.0	−96.1	−94.6	−103.3	−87.6	−95.7	−90.2	−98.6
3′-*O*-Methyldiplacone (**205**)	−134.5	−127.3	−107.4	−101.7	−130.8	−123.8	−115.3	−109.1	−104.7	−99.1
4′,5,7-Trihydroxy-6-methyl-8-prenylflavanone (**207**)	−116.4	−118.2	−101.1	−102.7	−110.0	−111.8	−90.9	−92.4	−104.2	−105.9
4′,5,7-Trihydroxy-8-methyl-6-prenylflavanone (**206**)	−110.4	−112.2	−110.1	−111.8	−110.4	−112.1	−94.5	−96.0	−105.6	−107.3
4*'*,5-Dihydroxy-7-methoxy-6-prenylflavanone (**208**)	−126.1	−128.2	−109.3	−111.1	−120.3	−122.3	−96.3	−97.8	−100.4	−102.0
4′,6,7-Trihydroxy-3′,5′-dimethoxyflavone (**209**)	−115.1	−119.7	−109.5	−113.9	−110.4	−114.8	−96.2	−100.1	−99.7	−103.7
4′,7-Dihydroxy-8-methylflavan (**210**)	−88.8	−100.6	−82.8	−93.7	−83.5	−94.5	−77.6	−87.9	−85.2	−96.4
4′-Hydroxy-5,7-dimethoxyflavone (**211**)	−104.1	−112.0	−84.7	−91.2	−109.3	−117.6	−88.6	−95.3	−94.1	−101.2
5″-(2-Hydroxybenzyl)isouvarinol (**216**)	−184.9	−151.1	−136.0	−111.2	−140.6	−114.9	−137.1	−112.0	−143.1	−116.9
5′-(1,1-Dimethyl-2-propenyl)-2′,4′,5,7-tetrahydroxy-6-prenylflavanone (**219**)	−128.0	−122.4	−106.4	−101.8	−116.3	−111.3	−98.5	−94.2	−68.6	−65.6
5′-(1,1-Dimethyl-2-propenyl)-2′,4′,5,7-tetrahydroxy-8-prenylflavanone (**220**)	−127.0	−121.5	−96.0	−91.8	−114.5	−109.5	−99.4	−95.1	−84.4	−80.8
5′-(1,1-Dimethyl-2-propenyl)-4′,5,7-trihydroxy-2′-methoxy-8-prenylflavanone (**221**)	−114.9	−108.8	−75.8	−71.7	−124.0	−117.3	−96.2	−91.0	−96.9	−91.7
5,6-Dihydroxy-4′,7,8-trimethoxyflavone (**212**)	−108.6	−111.4	−92.7	−95.1	−109.6	−112.5	−74.3	−76.2	−33.0	−33.8
5-Hydroxy-2′,4′,5′,7-Tetramethoxyflavone (**213**)	−115.1	−116.5	−103.7	−104.9	−123.3	−124.8	−91.7	−92.8	−56.8	−57.5
6,7-Dihydroxyflavone (**214**)	−98.1	−111.3	−90.2	−102.4	−89.5	−101.6	−88.1	−100.0	−94.5	−107.3
8-Methoxycirsilineol (**215**)	−124.1	−123.8	−111.2	−110.9	−111.1	−110.8	−88.7	−88.5	−99.3	−99.1
9,10-Dihydro-9,10-diacetoxy-3-methoxy-8,8-dimethyl-2-phenyl-4*H*,8*H*-benzo[1,2-*b*:3,4-*b*′]dipyran-4-one (**222**)	−81.8	−76.6	−73.8	−69.1	−76.4	−71.5	−113.3	−106.1	−75.8	−71.0
Abyssinone I (**223**)	−105.9	−111.0	−71.8	−75.3	−100.1	−104.9	−86.2	−90.4	−95.7	−100.4
Abyssinone IV (**224**)	−139.9	−137.4	−96.2	−94.5	−135.9	−133.4	−93.4	−91.7	−117.7	−115.6
Astragalin (**225**)	−136.8	−128.5	−103.9	−97.6	−127.6	−119.8	−103.7	−97.5	−107.4	−100.9
Bavachinin (**226**)	−119.3	−123.1	−111.3	−114.9	−118.8	−122.5	−96.5	−99.5	−100.8	−104.0
Betuletol (**227**)	−110.9	−115.3	−94.6	−98.4	−104.6	−108.8	−83.3	−86.6	−79.5	−82.7
Bonannione A (**228**)	−130.5	−126.4	−96.3	−93.3	−131.5	−127.5	−108.1	−104.7	−107.2	−103.9
Brosimone I (**229**)	−120.0	−115.1	−105.0	−100.7	−128.0	−122.8	−97.2	−93.3	−110.4	−106.0
Cassiaflavan (**230**)	−85.4	−98.4	−80.9	−93.3	−86.0	−99.2	−82.0	−94.6	−85.6	−98.7
Cerasinone (**231**)	−108.4	−112.7	−88.3	−91.8	−111.6	−116.1	−85.6	−89.1	−77.3	−80.4
Chrysin (**233**)	−97.1	−110.2	−87.1	−98.9	−89.5	−101.6	−84.9	−96.3	−88.6	−100.5
Chrysoeriol (**232**)	−105.9	−113.7	−99.9	−107.2	−105.7	−113.5	−95.2	−102.2	−93.4	−100.3
Corniculatusin (**234**)	−109.2	−113.3	−100.2	−104.1	−102.3	−106.2	−93.0	−96.5	−103.3	−107.2
Cudraflavone A (**235**)	−109.6	−105.4	−100.2	−96.3	−109.4	−105.1	−93.8	−90.1	−102.9	−98.9
Dihydroquercetin (**236**)	−103.3	−110.4	−96.7	−103.4	−96.3	−103.0	−94.5	−101.0	−98.0	−104.7
Eucalyptin (**237**)	−109.3	−114.1	−91.8	−95.9	−102.6	−107.2	−75.3	−78.6	−82.7	−86.4
Euchrestaflavanone A (**238**)	−137.0	−132.7	−112.6	−109.1	−137.5	−133.3	−93.2	−90.3	−50.9	−49.3
Flavaprenin (**239**)	−115.7	−119.2	−99.5	−102.5	−112.5	−115.9	−95.0	−97.8	−105.3	−108.4
Flemiflavanone D (**240**)	−128.9	−123.3	−97.0	−92.8	−132.8	−127.0	−108.0	−103.3	−86.7	−83.0
Glabranin (**241**)	−116.1	−121.5	−95.5	−99.9	−108.9	−113.9	−84.3	−88.2	−97.1	−101.6
Isoorientin (**243**)	−126.3	−118.7	−118.7	−111.5	−124.4	−116.8	−103.0	−96.7	−109.4	−102.7
Isoscoparin (**244**)	−117.1	−108.9	−125.0	−116.2	−121.4	−112.8	−98.4	−91.5	−103.4	−96.1
Kaempferol (**242**)	−93.2	−101.7	−90.1	−98.3	−92.5	−100.9	−87.0	−94.9	−95.8	−104.5
Kushenol A (**245**)	−141.0	−136.6	−109.0	−105.6	−139.7	−135.4	−92.6	−89.7	−100.7	−97.6
Kushenol S (**246**)	−118.5	−122.1	−95.5	−98.4	−112.5	−115.8	−84.8	−87.3	−95.4	−98.3
Kushenol U (**247**)	−136.4	−130.7	−114.1	−109.3	−142.9	−136.9	−105.5	−101.1	−105.0	−100.6
Kushenol V (**248**)	−126.9	−123.8	−113.5	−110.7	−123.6	−120.5	−91.5	−89.3	−98.0	−95.6
Kushenol W (**249**)	−126.7	−125.1	−105.8	−104.4	−119.8	−118.3	−90.8	−89.7	−99.2	−97.9
Leachianone A (**250**)	−142.5	−134.9	−100.9	−95.5	−140.0	−132.5	−102.6	−97.1	−105.2	−99.6
Leachianone G (**251**)	−115.9	−117.6	−100.5	−101.9	−120.3	−122.0	−92.2	−93.5	−106.0	−107.5
Licoflavanone (**252**)	−116.0	−119.5	−96.0	−98.9	−121.6	−125.2	−100.7	−103.7	−100.1	−103.1
Licoflavone C (**253**)	−115.0	−118.7	−100.0	−103.2	−117.5	−121.3	−91.8	−94.7	−108.5	−112.0
Licoflavonol (**254**)	−118.6	−120.5	−109.3	−111.1	−118.6	−120.5	−99.0	−100.6	−105.3	−107.0
Lonchocarpol A (**255**)	−121.9	−118.1	−117.9	−114.2	−123.5	−119.6	−102.6	−99.4	−118.0	−114.3
Loranthin (**256**)	−127.5	−115.8	−118.2	−107.3	−128.5	−116.7	−114.6	−104.1	−109.2	−99.2
Loxophlebal A (**257**)	−128.6	−118.7	−104.0	−96.0	−119.5	−110.3	−104.6	−96.5	−92.0	−84.9
Lucenin 2 (**258**)	−86.0	−72.9	−103.5	−87.7	−108.6	−92.0	−134.2	−113.8	−123.9	−105.0
Macarangaflavanone A (**259**)	−138.2	−133.9	−104.3	−101.0	−135.6	−131.4	−107.5	−104.2	−81.3	−78.7
Malvidin (**260**)	−113.5	−117.9	−96.3	−100.0	−107.1	−111.2	−102.2	−106.2	−92.5	−96.2
Myricetin (**261**)	−103.3	−108.8	−99.3	−104.5	−104.1	−109.6	−97.9	−103.1	−99.2	−104.5
Natsudaidain (**262**)	−123.4	−118.6	−112.1	−107.8	−118.4	−113.8	−90.4	−86.9	−45.0	−43.2
Nevadensin (**263**)	−119.8	−122.9	−96.6	−99.1	−113.7	−116.6	−85.7	−87.9	−67.8	−69.6
*O*-Methylpongaglabol (**264**)	−102.3	−110.8	−89.5	−97.0	−99.6	−107.9	−91.1	−98.7	−93.7	−101.6
Paratocarpin L (**265**)	−132.3	−128.2	−111.8	−108.3	−134.4	−130.2	−102.9	−99.7	−105.8	−102.6
Persicogenin (**266**)	−107.9	−113.8	−91.9	−96.9	−110.9	−117.0	−74.9	−79.0	−90.3	−95.2
Pilosanol A (**267**)	−136.9	−120.8	−118.8	−104.8	−128.4	−113.3	−114.8	−101.4	−98.6	−87.0
Pilosanol B (**268**)	−139.4	−124.1	−114.6	−102.1	−114.3	−101.8	−111.3	−99.1	−93.6	−83.4
Pilosanol C (**269**)	−140.8	−125.3	−123.9	−110.3	−126.6	−112.7	−116.7	−103.9	−108.7	−96.8
Pinocembrin (**270**)	−94.5	−107.0	−83.1	−94.1	−88.3	−99.9	−82.7	−93.6	−84.7	−95.9
Pongaflavone (**271**)	−99.7	−103.3	−78.6	−81.4	−103.1	−106.8	−89.6	−92.8	−101.2	−104.9
Quercetin (**272**)	−102.9	−110.3	−94.6	−101.4	−96.7	−103.6	−93.8	−100.5	−99.8	−106.9
Quercetin 3-methyl ether (**273**)	−100.4	−106.0	−91.7	−96.7	−96.5	−101.8	−94.0	−99.2	−104.6	−110.4
Remangiflavanone A (**274**)	−133.0	−127.4	−112.4	−107.6	−133.1	−127.5	−101.1	−96.9	−106.7	−102.2
Remangiflavanone B (**275**)	−136.4	−130.5	−109.2	−104.4	−142.3	−136.2	−105.7	−101.2	−104.0	−99.5
Sanggenon G (**276**)	−107.6	−87.3	−105.6	−85.7	−118.6	−96.3	−129.7	−105.3	−97.5	−79.2
Sigmoidin A (**277**)	−134.3	−128.5	−119.0	−113.8	−134.4	−128.6	−111.0	−106.2	−103.3	−98.8
Sigmoidin B (**278**)	−120.5	−122.2	−97.5	−98.9	−120.3	−122.0	−106.6	−108.1	−78.8	−79.9
Sigmoidin L (**279**)	−120.7	−120.8	−99.4	−99.5	−121.3	−121.4	−109.3	−109.5	−89.0	−89.1
Siraitiflavandiol (**280**)	−118.4	−120.1	−105.6	−107.0	−117.0	−118.6	−113.1	−114.7	−96.9	−98.3
Solophenol D (**281**)	−145.5	−137.7	−118.7	−112.3	−141.7	−134.1	−115.3	−109.1	−85.0	−80.4
Sophoraflavanone G (**282**)	−137.3	−131.3	−110.3	−105.5	−144.7	−138.4	−99.3	−95.0	−106.7	−102.1
Sternbin (**283**)	−101.5	−108.8	−93.7	−100.4	−106.8	−114.4	−95.3	−102.1	−96.1	−103.0
Sudachitin (**284**)	−120.7	−122.0	−112.6	−113.8	−118.2	−119.5	−83.4	−84.3	−101.0	−102.0
Uvarinol (**285**)	−153.4	−132.7	−122.7	−106.1	−149.9	−129.6	−119.7	−103.6	−135.1	−116.9
Vahliabiflavone (**286**)	no dock	no dock	−87.6	−75.9	no dock	no dock	−115.6	−100.2	−57.4	−49.8
Vitexin (**287**)	−129.6	−123.2	−97.8	−93.0	−129.8	−123.4	−99.0	−94.1	−114.9	−109.2
Wogonin (**288**)	−103.2	−112.8	−90.7	−99.2	−96.9	−106.0	−87.5	−95.7	−91.5	−100.0
**Isoflavonoids**										
2″,3″-Epoxybolusanthol B (**289**)	−126.2	−126.1	−94.8	−94.7	−123.8	−123.7	−98.9	−98.9	−95.6	−95.6
3′,5,7-Trihydroxy-4′-methoxy-5′,6-diprenylisoflavanone (**290**)	−131.8	−124.7	−111.5	−105.5	−126.4	−119.7	−109.5	−103.6	−102.4	−96.9
4″-Hydroxydiphysolone (**292**)	−122.8	−122.7	−104.0	−103.9	−120.3	−120.2	−98.7	−98.6	−100.7	−100.6
5,7-Dihydroxy-2′-methoxy-3′,4′-methylenedioxyisoflavanone (**293**)	−119.3	−124.1	−91.5	−95.2	−116.5	−121.2	−77.2	−80.3	−79.8	−83.0
6*a*-Hydroxyphaseollin (**291**)	−103.5	−106.7	−86.7	−89.4	−92.0	−95.0	−91.2	−94.1	−83.2	−85.8
Amorphaquinone (**294**)	−114.3	−117.0	−82.7	−84.7	−112.4	−115.1	−83.7	−85.7	−83.1	−85.1
Asphodelin A (**295**)	−90.7	−100.9	−92.6	−103.0	−95.0	−105.7	−81.1	−90.2	−86.2	−95.9
Bidwillon A (**296**)	−125.7	−121.8	−110.5	−107.0	−126.3	−122.4	−103.4	−100.2	−111.6	−108.1
Bolucarpan A (**297**)	−110.0	−110.4	−96.3	−96.5	−101.1	−101.4	−73.2	−73.4	−71.7	−71.9
Bolucarpan B (**298**)	−106.3	−106.8	−97.9	−98.3	−96.6	−97.1	−77.0	−77.3	−72.4	−72.8
Bolucarpan D (**299**)	−87.5	−90.4	−91.6	−94.7	−79.7	−82.4	−76.7	−79.3	−63.6	−65.8
Bolusanthol B (**300**)	−124.5	−126.3	−99.8	−101.2	−124.6	−126.3	−102.3	−103.7	−102.4	−103.8
Cajanol (**301**)	−109.2	−115.2	−88.8	−93.7	−104.5	−110.3	−85.5	−90.3	−79.4	−83.7
Chandalone (**302**)	−118.7	−115.4	−104.3	−101.4	−119.5	−116.2	−100.4	−97.7	−63.3	−61.6
Dalversinol A (**303**)	−123.2	−117.9	−92.8	−88.8	−123.0	−117.7	−102.2	−97.8	−94.5	−90.4
Derrisin (**304**)	−87.0	−82.9	−73.4	−70.0	−63.7	−60.8	−78.5	−74.8	−18.9	−18.1
Erybraedin A (**305**)	−125.4	−123.1	−98.2	−96.5	−123.2	−121.0	−104.0	−102.1	−89.0	−87.4
Erybraedin D (**306**)	−129.2	−127.1	−94.8	−93.3	−126.3	−124.3	−92.7	−91.1	−80.0	−78.7
Erypoegin I (**307**)	−106.4	−106.5	−93.4	−93.5	−117.6	−117.7	−104.0	−104.2	−76.8	−76.9
Erysubin F (**308**)	−139.5	−137.2	−114.2	−112.3	−132.7	−130.6	−113.7	−111.8	−79.4	−78.1
Eryvarin V (**309**)	−104.7	−100.3	−99.6	−95.5	−96.9	−92.8	−107.9	−103.4	−86.3	−82.7
Eryvarin W (**310**)	−118.1	−116.2	−104.3	−102.6	−115.0	−113.1	−107.6	−105.9	−75.3	−74.1
Eryzerin C (**311**)	−128.1	−125.6	−104.9	−102.8	−127.7	−125.1	−104.6	−102.5	−114.6	−112.4
Eryzerin D (**312**)	−122.6	−120.4	−99.5	−97.7	−124.7	−122.4	−99.3	−97.5	−98.9	−97.1
Euchretin A (**313**)	−128.3	−116.1	−92.3	−83.5	−130.0	−117.6	−113.0	−102.2	−77.2	−69.9
Gancaonin C (**314**)	−113.0	−114.8	−97.2	−98.7	−115.8	−117.6	−102.6	−104.3	−107.4	−109.2
Genistein (**315**)	−95.8	−106.5	−87.7	−97.5	−96.8	−107.6	−84.2	−93.6	−93.6	−104.1
Glycyrrhisoflavone (**316**)	−121.6	−123.6	−98.9	−100.5	−122.2	−124.1	−100.7	−102.3	−95.8	−97.3
Hispaglabridin A (**317**)	−122.9	−120.7	−101.0	−99.2	−122.6	−120.4	−94.1	−92.4	−95.5	−93.8
Hispaglabridin B (**318**)	−112.8	−111.0	−31.1	−30.6	−107.5	−105.8	−84.8	−83.4	−61.9	−60.9
Hydroxycristacarpone (**319**)	−96.2	−96.3	−87.8	−87.9	−91.8	−91.9	−91.6	−91.7	−63.6	−63.7
Isoneorautenol (**320**)	−109.2	−114.5	−75.7	−79.4	−114.0	−119.6	−81.1	−85.0	−72.2	−75.7
Lachnoisoflavone A (**321**)	−112.5	−114.5	−98.9	−100.6	−116.3	−118.3	−94.8	−96.5	−97.4	−99.1
Licoisoflavanone (**322**)	−110.9	−112.7	−64.0	−65.1	−98.6	−100.1	−92.6	−94.1	−83.0	−84.4
Licoricidin (**323**)	−136.7	−130.7	−108.7	−104.0	−138.7	−132.7	−108.4	−103.7	−107.1	−102.4
Lupinalbin C (**324**)	−113.1	−113.4	−104.1	−104.4	−110.9	−111.2	−92.0	−92.3	−93.9	−94.2
Mucronulatol (**326**)	−99.6	−106.7	−80.0	−85.8	−101.4	−108.6	−86.3	−92.5	−92.6	−99.2
Neomillinol (**325**)	−103.3	−107.9	−96.9	−101.1	−113.5	−118.5	−93.8	−98.0	−93.6	−97.8
Pendulone (**327**)	−107.6	−113.6	−79.4	−83.8	−105.6	−111.4	−92.9	−98.1	−74.3	−78.4
Phyllanone B (**328**)	−138.9	−131.4	−98.8	−93.5	−139.5	−132.0	−112.8	−106.8	−101.9	−96.5
Shinpterocarpin (**329**)	−97.7	−102.5	−81.2	−85.1	−95.2	−99.8	−95.3	−99.9	−79.1	−82.9
**Neoflavonoids**										
Inophyllum A (**330**)	−103.7	−100.8	−82.6	−80.3	−101.8	−99.0	−90.4	−87.9	−77.2	−75.1
Inophyllum C (**331**)	−111.3	−108.4	−97.1	−94.6	−107.6	−104.8	−87.4	−85.2	−71.3	−69.5
Mammea A/BA (**332**)	−127.9	−124.1	−110.5	−107.2	−117.2	−113.8	−100.8	−97.8	−112.0	−108.7
Mammea A/BB (**333**)	−123.4	−119.8	−101.6	−98.6	−118.6	−115.1	−95.0	−92.2	−104.6	−101.6
Mesuol (**334**)	−124.3	−122.1	−108.0	−106.1	−122.2	−120.0	−101.2	−99.4	−106.9	−105.0
**Pterocarpans**										
1-Methoxyphaseollidin (**356**)	−117.4	−119.3	−97.6	−99.2	−118.8	−120.7	−97.6	−99.2	−68.5	−69.6
Aracarpene 1 (**357**)	−105.0	−112.7	−84.6	−90.9	−105.1	−112.8	−87.7	−94.2	−89.8	−96.5
Aracarpene 2 (**358**)	−102.9	−110.5	−94.1	−101.1	−108.8	−116.9	−78.7	−84.5	−85.6	−91.9
Calopocarpin (**360**)	−111.9	−117.1	−103.9	−108.7	−113.2	−118.5	−102.7	−107.4	−99.3	−103.9
Cristacarpin (**359**)	−121.4	−123.4	−91.5	−92.9	−119.0	−120.9	−98.3	−99.8	−88.5	−89.9
Erythbidin D (**362**)	−103.0	−110.6	−93.5	−100.4	−112.8	−121.1	−88.4	−95.0	−80.6	−86.6
Eryzerin E (**363**)	−140.8	−135.0	−96.5	−92.4	−129.6	−124.2	−99.4	−95.2	−102.8	−98.5
Fuscacarpan B (**364**)	−116.4	−116.6	−88.4	−88.6	−118.6	−118.7	−99.2	−99.3	−88.9	−89.0
Fuscacarpan C (**365**)	−116.5	−116.7	−90.6	−90.7	−111.3	−111.4	−99.8	−99.9	−86.8	−86.9
Glycyrol (**366**)	−122.3	−122.9	−102.1	−102.6	−119.2	−119.7	−88.8	−89.2	−102.0	−102.4
Glycyrrhizol A (**367**)	−135.5	−130.0	−54.2	−52.0	−124.6	−119.6	−100.1	−96.1	−87.0	−83.5
Glycyrrhizol B (**368**)	−110.1	−112.3	−80.8	−82.4	−117.7	−120.1	−86.6	−88.4	−73.5	−75.0
Sandwicensin (**369**)	−115.6	−119.3	−103.1	−106.3	−116.4	−120.1	−94.8	−97.8	−98.5	−101.6
Variabilin (**370**)	−103.9	−111.5	−85.6	−91.9	−107.1	−115.0	−83.4	−89.5	−73.5	−78.9
*ent*-Sophoracarpan A (**361**)	−106.2	−114.0	−76.3	−82.0	−99.0	−106.2	−89.4	−96.0	−82.8	−88.9
**Chromones**										
3-(3-Hydroxy-4-methoxybenzylidene)-6,7-dimethoxy-4-chromanone (**371**)	−123.1	−126.5	−98.9	−101.6	−116.1	−119.3	−86.7	−89.1	−75.0	−77.1
4′,5,7-Trihydroxy-6,8-dimethylhomoisoflavanone (**372**)	−102.9	−108.9	−93.5	−98.8	−97.9	−103.6	−83.4	−88.2	−84.3	−89.1
4′,5,7-Trihydroxy-6-methylhomoisoflavanone (**373**)	−99.8	−107.1	−93.5	−100.4	−104.1	−111.8	−86.2	−92.6	−82.2	−88.2
7-*O*-Methylbonducellin (**374**)	−108.5	−117.0	−94.7	−102.1	−108.5	−117.0	−83.2	−89.8	−77.7	−83.8
8-Methylophiopogonanone B (**375**)	−113.2	−118.0	−91.8	−95.7	−110.5	−115.1	−82.9	−86.4	−77.3	−80.6
Bonducellin (**376**)	−102.6	−112.4	−88.3	−96.8	−104.4	−114.4	−94.8	−103.9	−85.8	−94.1
Odoratumone A (**377**)	−117.1	−120.2	−95.2	−97.7	−117.5	−120.6	−88.2	−90.5	−74.8	−76.8
Sappanone A 3′,4′-methylene ether (**378**)	−110.6	−119.3	−96.3	−103.9	−109.0	−117.6	−96.1	−103.6	−90.8	−97.9
Sappanone A 4′-methyl ether (**379**)	−108.5	−116.7	−95.2	−102.4	−108.3	−116.6	−96.6	−104.0	−90.5	−97.4
Sappanone A trimethyl ether (**380**)	−123.0	−128.4	−103.8	−108.4	−123.7	−129.1	−87.5	−91.4	−85.4	−89.2
**Condensed Tannins**										
GB 1 (**381**)	no dock	no dock	−35.1	−30.7	−37.2	−32.5	−103.7	−90.5	−79.9	−69.8
Proanthocyanidin A_1_ (**382**)	−80.6	−69.6	−104.7	−90.5	−41.3	−35.7	−111.7	−96.5	−96.1	−83.0
Proanthocyanidin A_2_ (**383**)	−70.8	−61.1	−94.8	−81.9	−21.3	−18.4	−107.4	−92.8	−104.1	−89.9
Procyanidin B_4_ (**384**)	−66.7	−57.6	−97.2	−83.9	no dock	no dock	−119.8	−103.3	−65.5	−56.5
Procyanidin B_5_ (**385**)	−68.4	−59.1	−61.6	−53.1	−41.8	−36.0	−115.2	−99.4	−88.3	−76.2
Procyanidin B_6_ (**386**)	−74.6	−64.4	no dock	no dock	−47.5	−41.0	−119.6	−103.2	−104.0	−89.7
Teatannin (**387**)	−139.2	−131.4	−62.8	−59.3	−143.4	−135.3	−116.0	−109.5	−108.3	−102.2
**Coumarins**										
4,5′,8′-Trihydroxy-5-methyl-3,7′-bicoumarin (**344**)	−109.2	−111.2	−104.3	−106.2	−112.1	−114.1	−94.5	−96.2	−93.1	−94.7
6-Geranyl-5,7-dihydroxy-8(2-methylbutanoyl)-4-phenylcoumarin (**345**)	−154.6	−142.5	−112.3	−103.6	−166.5	−153.4	−113.8	−104.9	−120.1	−110.7
(–)-Heliettin (**354**)	−79.0	−100.9	−66.0	−84.4	−79.6	−101.7	−81.8	−104.6	−77.4	−98.9
Aesculin (**347**)	−115.1	−118.5	−108.7	−112.0	−115.1	−118.5	−89.2	−91.9	−93.3	−96.1
Alloimperatorin (**346**)	−103.5	−115.1	−95.0	−105.7	−103.4	−115.0	−93.1	−103.6	−95.9	−106.6
Anofinic acid (**348**)	−86.1	−105.1	−75.2	−91.8	−90.1	−110.0	−75.7	−92.5	−88.4	−108.0
Calaustralin (**349**)	−125.0	−121.5	−95.8	−93.2	−120.0	−116.6	−92.8	−90.2	−101.5	−98.7
Calophyllolide (**350**)	−112.3	−108.1	−85.2	−82.0	−114.2	−110.0	−99.2	−95.5	−12.5	−12.0
Dicoumarol (**351**)	−114.7	−118.6	−80.0	−82.7	−115.5	−119.4	−88.1	−91.1	−85.1	−88.0
Dipetalolactone (**352**)	−100.0	−106.2	−83.5	−88.7	−89.6	−95.2	−84.0	−89.2	−75.1	−79.8
Glycycoumarin (**353**)	−116.4	−116.7	−110.0	−110.3	−124.0	−124.3	−103.9	−104.2	−101.6	−101.9
Marmesin (**355**)	−96.2	−110.3	−81.6	−93.6	−96.9	−111.1	−94.6	−108.5	−93.4	−107.1
**Stilbenoids**										
2-(2,4-Dihydroxyphenyl-5-(1-propenyl)benzofurans (**388**)	−106.3	−118.8	−98.6	−110.2	−111.2	−124.3	−90.3	−100.9	−94.9	−106.1
Albanol A (**389**)	no dock	no dock	−85.8	−74.8	no dock	no dock	−117.8	−102.6	−97.4	−84.8
Albanol B (**390**)	−124.6	−108.8	−99.3	−86.7	−77.3	−67.5	−114.8	−100.2	−87.8	−76.7
Amorfrutin A (**391**)	−121.4	−125.0	−111.8	−115.1	−117.7	−121.2	−100.0	−103.0	−97.4	−100.3
Cajaninstilbene acid (**392**)	−122.8	−126.7	−117.7	−121.4	−117.5	−121.2	−96.0	−99.0	−96.4	−99.4
Calodenin B (**393**)	−160.9	−143.4	−143.2	−127.7	−143.4	−127.8	−138.4	−123.4	−124.1	−110.6
Centrolobofuran (**394**)	−95.5	−108.1	−100.9	−114.2	−100.1	−113.4	−89.5	−101.4	−92.8	−105.0
Cochinchinenene A (**395**)	−134.0	−118.3	−87.8	−77.5	−97.1	−85.7	−124.2	−109.6	−99.0	−87.3
Cochinchinenene B (**396**)	−147.2	−132.2	−118.2	−106.2	−151.4	−136.0	−126.2	−113.3	−98.9	−88.9
Cochinchinenene C (**397**)	−142.5	−129.2	−104.2	−94.5	−139.6	−126.5	−115.1	−104.4	−90.8	−82.3
Cochinchinenene D (**398**)	−140.2	−128.3	−98.1	−89.8	−134.9	−123.5	−107.7	−98.6	−109.3	−100.1
Egonol (**399**)	−126.3	−131.8	−111.4	−116.3	−123.3	−128.8	−108.9	−113.8	−97.6	−102.0
Erypoegin F (**400**)	−128.1	−130.4	−121.7	−123.9	−133.4	−135.8	−97.8	−99.6	−114.0	−116.0
Erythbidin E (**401**)	−99.8	−112.9	−94.8	−107.3	−99.3	−112.4	−89.2	−101.0	−92.2	−104.3
Eryvarin Q (**402**)	−143.2	−139.0	−121.3	−117.7	−134.3	−130.4	−108.5	−105.4	−65.7	−63.8
Gancaonin I (**403**)	−125.3	−127.3	−105.9	−107.6	−124.1	−126.1	−102.0	−103.6	−104.0	−105.7
Glyinflanin H (**404**)	−110.0	−117.0	−86.0	−91.5	−106.9	−113.7	−93.1	−99.0	−92.1	−98.1
Kuwanol A (**405**)	no dock	no dock	−94.5	−82.2	−33.8	−29.4	−91.3	−79.4	−97.8	−85.0
Licobenzofuran (**406**)	−134.2	−136.3	−111.7	−113.5	−129.4	−131.5	−114.2	−116.1	−86.1	−87.5
Licocoumarone (**407**)	−122.8	−126.4	−102.4	−105.5	−121.4	−125.0	−100.6	−103.6	−103.9	−107.0
Mulberrofuran D (**408**)	−150.0	−141.1	−137.9	−129.8	−151.2	−142.2	−118.1	−111.1	−101.6	−95.5
Mulberrofuran Y (**409**)	−153.9	−149.2	−127.2	−123.2	−146.2	−141.7	−124.5	−120.7	−115.6	−112.0
Pinosylvin (**410**)	−86.0	−103.7	−83.5	−100.6	−84.6	−102.0	−81.6	−98.3	−88.2	−106.3
Schweinfurthin A (**411**)	−48.3	−42.4	no dock	no dock	no dock	no dock	−133.1	−116.9	−76.9	−67.5
Shanciguol 3-methyl ether (**412**)	−130.9	−123.5	−113.4	−107.0	−123.4	−116.4	−112.2	−105.9	−102.5	−96.7
Stemofuran R (**413**)	−117.3	−122.2	−90.5	−94.3	−115.9	−120.8	−84.2	−87.7	−79.7	−83.1
Stilbostemin S (**414**)	−100.6	−109.5	−100.4	−109.3	−106.4	−115.8	−89.5	−97.4	−80.6	−87.7
Thunberginol F (**415**)	−96.1	−106.9	−95.4	−106.1	−101.3	−112.6	−96.9	−107.8	−98.8	−109.8
(7*E*,7′*R*,8′*R*)-ε-Viniferin (**416**)	−121.2	−113.4	−113.6	−106.3	−108.8	−101.8	−108.8	−101.7	−109.2	−102.1
(7*E*,7′*S*,8′*S*)-ε-Viniferin (**417**)	−133.0	−124.4	−95.4	−89.3	−127.6	−119.3	−111.9	−104.6	−114.7	−107.2
**Phenylpropanoids and Lignans**										
(*E*)-Cinnamaldehyde (**418**)	−72.2	−101.8	−60.9	−86.0	−73.9	−104.3	−64.7	−91.3	−65.9	−93.0
3,4-Dimethylcinnamaldehyde (**419**)	−79.6	−105.4	−70.3	−93.1	−82.3	−109.0	−69.9	−92.6	−73.9	−97.8
Methyleugenol (**422**)	−79.9	−102.1	−73.4	−93.7	−82.3	−105.2	−70.5	−90.1	−75.4	−96.3
*p*-Coumaraldehyde (**420**)	−76.9	−104.4	−65.4	−88.9	−77.5	−105.3	−69.2	−94.1	−73.4	−99.8
*p*-Methoxycinnamaldehyde (**421**)	−76.9	−101.4	−72.3	−95.3	−80.3	−105.8	−69.4	−91.5	−73.4	−96.8
(−)-Asarinin (**423**)	−137.5	−139.7	−103.8	−105.4	−134.7	−136.8	−101.8	−103.5	−98.2	−99.8
Nordihydroguaiaretic acid (**424**)	−112.3	−120.3	−94.7	−101.5	−110.9	−118.8	−97.6	−104.5	−95.1	−101.8
**Xanthones**										
2-Deoxy-4-Hydroxycudratricusxanthone D (**425**)	−126.3	−123.8	−93.9	−92.1	−112.0	−109.8	−92.4	−90.6	−96.8	−94.9
Calozeyloxanthone (**426**)	−81.7	−81.2	−87.9	−87.4	−99.7	−99.1	−80.5	−80.0	−72.2	−71.7
Cycloartobiloxanthone (**427**)	−106.3	−100.9	−47.1	−44.7	−107.5	−102.0	−94.5	−89.7	−67.6	−64.2
Formoxanthone C (**428**)	−118.9	−116.4	−103.8	−101.6	−122.4	−119.8	−101.7	−99.5	−102.5	−100.4
Garciniacowone (**429**)	−131.0	−121.5	−85.4	−79.2	−110.7	−102.6	−115.1	−106.7	−103.2	−95.6
Globulixanthone C (**430**)	−100.1	−104.5	−77.5	−80.9	−103.0	−107.6	−79.4	−83.0	−90.1	−94.1
Globulixanthone D (**431**)	−107.6	−110.6	−98.8	−101.5	−109.6	−112.6	−84.6	−87.0	−94.7	−97.3
Globulixanthone E (**432**)	−61.5	−51.9	−96.8	−81.7	−68.4	−57.7	−107.1	−90.4	−91.6	−77.3
Morellin (**433**)	no dock	no dock	no dock	no dock	no dock	no dock	−115.0	−101.2	−48.8	−42.9
Nigrolineaxanthone N (**434**)	−128.2	−125.3	−104.0	−101.6	−126.4	−123.5	−100.6	−98.3	−106.0	−103.6
Pinselin (**435**)	−98.0	−105.2	−87.3	−93.8	−95.7	−102.8	−78.1	−83.8	−83.0	−89.1
Scortechinone B (**436**)	no dock	no dock	−65.1	−55.7	no dock	no dock	−125.7	−107.6	−104.2	−89.2
Symphonin (**437**)	−134.9	−126.4	−112.2	−105.0	−125.9	−118.0	−96.7	−90.6	−93.1	−87.2
**Hydrolyzable Tannins**										
1,2,3,4,6-Pentagalloylglucose (**335**)	−65.6	−48.1	−137.4	−100.9	−16.9	−12.4	−170.4	−125.0	−130.1	−95.4
Aceritannin (**438**)	−151.6	−140.3	−116.8	−108.1	−158.1	−146.4	−118.4	−109.6	−118.4	−109.6
Ginnalin B (**439**)	−108.1	−114.1	−99.8	−105.3	−113.7	−120.0	−97.2	−102.6	−100.9	−106.5
Ginnalin C (**440**)	−105.5	−111.3	−99.0	−104.4	−105.8	−111.7	−94.3	−99.5	−104.0	−109.7
Panconoside A (**441**)	−141.1	−116.6	−118.4	−97.9	−99.7	−82.4	−129.1	−106.7	−85.0	−70.3
**Miscellaneous Phenolics**										
1,3,7,9-Tetrahydroxy-4,6-dimethyl-2,8-bis(2-methyl-propanoyl)-dibenzofuran (**442**)	−114.8	−112.0	−89.7	−87.5	−110.1	−107.4	−97.0	−94.7	−58.8	−57.4
2′,4′-Dihydroxy-6′-methoxy-3′-methylacetophenone (**443**)	−85.3	−105.5	−69.2	−85.6	−83.8	−103.7	−69.5	−85.9	−70.6	−87.3
3′,4′-Dihydroxyacetophenone (**444**)	−71.3	−96.0	−63.9	−86.1	−72.2	−97.2	−74.8	−100.7	−73.4	−98.9
4′-*O*-Methylhonokiol (**446**)	−109.1	−119.9	−99.7	−109.6	−108.3	−119.0	−85.9	−94.4	−94.2	−103.5
4-Deoxyadhumulone 2″,3″-epoxide (**445**)	−115.3	−116.3	−98.6	−99.4	−111.3	−112.3	−93.3	−94.0	−104.3	−105.1
7-(3,4-Dihydroxy-5-methoxyphenyl)-1-phenyl-4-hepten-3-one (**447**)	−123.7	−129.2	−100.1	−104.5	−128.8	−134.5	−99.2	−103.6	−100.6	−105.0
Agrimol C (**449**)	−120.0	−98.6	−101.9	−83.8	−115.8	−95.2	−129.7	−106.7	−84.1	−69.1
Agrimol F (**450**)	−144.8	−120.7	−113.8	−94.9	−123.0	−102.5	−121.3	−101.2	−98.9	−82.5
Agrimol G (**451**)	−153.5	−126.2	−95.4	−78.4	−109.1	−89.7	−123.7	−101.7	−95.6	−78.6
Arzanol (**452**)	−133.2	−129.7	−94.5	−92.0	−132.9	−129.4	−106.5	−103.7	−113.5	−110.5
Aspidinol C (**448**)	−89.1	−105.5	−76.8	−90.9	−92.4	−109.3	−69.0	−81.7	−79.4	−93.9
Bruguierol C (**453**)	−81.4	−99.1	−69.5	−84.6	−81.2	−98.8	−81.3	−98.9	−76.9	−93.6
Cearoin (**454**)	−97.6	−112.3	−89.7	−103.2	−98.3	−113.0	−79.1	−91.0	−87.8	−101.0
Citrusnin A (**455**)	−102.4	−119.7	−89.3	−104.4	−104.3	−122.0	−87.5	−102.4	−93.3	−109.2
Cochinchinenin B (**456**)	−155.4	−137.0	−125.2	−110.3	−140.5	−123.9	−145.8	−128.6	−112.3	−99.0
Cochinchinenin C (**457**)	−155.7	−137.3	−117.3	−103.4	−143.2	−126.3	−135.2	−119.2	−106.1	−93.5
Drummondin D (**458**)	−112.1	−101.8	−102.5	−93.0	−89.6	−81.4	−118.0	−107.2	−91.9	−83.4
Drummondin E (**459**)	−147.2	−133.5	−117.7	−106.8	−112.3	−101.8	−116.4	−105.5	−99.6	−90.3
Eleutherol (**460**)	−90.3	−103.9	−83.8	−96.4	−84.7	−97.4	−76.4	−87.9	−63.0	−72.4
Ellagicacid (**461**)	−103.2	−110.6	−91.7	−98.3	−100.7	−107.9	−85.7	−91.8	−74.6	−80.0
Epicoccolide A (**462**)	−105.3	−105.1	−94.6	−94.3	−111.8	−111.6	−88.6	−88.4	−45.9	−45.8
Gibbilimbol A (**464**)	−99.1	−115.9	−87.7	−102.6	−102.4	−119.8	−85.1	−99.5	−84.9	−99.3
Gibbilimbol B (**465**)	−95.8	−112.0	−87.8	−102.6	−102.3	−119.7	−82.8	−96.8	−87.4	−102.2
Grifolin (**466**)	−127.7	−133.1	−98.6	−102.7	−130.6	−136.0	−106.6	−111.1	−90.8	−94.7
Hyperbrasilol A (**467**)	−126.0	−109.4	−97.7	−84.8	−128.1	−111.1	−113.9	−98.9	−99.3	−86.2
Hyperbrasilol B (**468**)	−129.5	−113.4	−74.5	−65.2	−95.0	−83.3	−105.4	−92.4	−97.0	−85.0
Hyperbrasilol C (**469**)	−97.7	−85.5	−122.7	−107.4	−121.5	−106.3	−110.2	−96.5	−108.5	−94.9
Isodrummondin D (**470**)	−117.5	−106.7	−91.0	−82.6	−106.3	−96.5	−108.8	−98.8	−88.1	−80.0
Isohyperbrasilol B (**471**)	−129.2	−113.2	−110.1	−96.4	−119.5	−104.7	−116.1	−101.7	−83.9	−73.5
Isouliginosin B (**472**)	−117.3	−106.4	−110.7	−100.3	−122.5	−111.1	−118.8	−107.8	−84.6	−76.7
Italipyrone (**473**)	−126.0	−122.9	−100.0	−97.6	−130.7	−127.5	−112.3	−109.6	−91.9	−89.6
Knerachelin A (**474**)	−122.6	−131.6	−108.9	−117.0	−121.2	−130.1	−94.5	−101.5	−92.3	−99.0
Knerachelin B (**475**)	−110.8	−123.2	−99.8	−111.0	−111.1	−123.5	−84.2	−93.6	−90.8	−101.0
Magnolol (**476**)	−101.7	−113.7	−92.5	−103.3	−103.1	−115.2	−87.0	−97.2	−79.1	−88.4
Myrtucommulone A (**477**)	−94.6	−77.7	−82.4	−67.8	no dock	no dock	−129.0	−106.1	−106.7	−87.7
Myrtucommulone B (**478**)	−42.0	−40.5	−77.0	−74.2	−49.4	−47.6	−74.2	−71.6	−76.5	−73.8
Obovatol (**479**)	−111.1	−121.8	−87.1	−95.5	−109.0	−119.4	−93.3	−102.3	−85.5	−93.7
Oenostacin (**480**)	−112.5	−125.7	−101.7	−113.6	−110.6	−123.6	−99.4	−111.1	−106.0	−118.4
Paeonol (**481**)	−80.1	−104.8	−65.8	−86.1	−78.0	−102.0	−74.2	−97.1	−76.4	−99.9
Perlatolic acid (**482**)	−144.5	−136.1	−112.3	−105.8	−140.9	−132.7	−121.1	−114.1	−109.0	−102.7
Plicatipyrone (**483**)	−114.3	−109.9	−93.0	−89.4	−120.6	−116.0	−101.0	−97.1	−79.5	−76.4
Propterol (**484**)	−92.7	−106.6	−85.4	−98.2	−96.4	−110.9	−83.7	−96.2	−87.8	−101.0
Pulverulentone B (**485**)	−99.5	−115.3	−80.5	−93.3	−100.2	−116.2	−73.4	−85.1	−79.1	−91.7
Quinquangulin (**486**)	−103.2	−112.6	−93.4	−101.9	−102.6	−111.9	−78.3	−85.4	−80.9	−88.3
Rhodomyrtone (**487**)	−75.2	−71.0	−94.6	−89.3	−39.1	−36.9	−100.6	−94.9	−96.9	−91.4
Rosmarinic acid (**488**)	−126.0	−127.3	−112.1	−113.3	−136.8	−138.2	−107.2	−108.3	−106.5	−107.6
Rubanthrone A (**489**)	−113.5	−112.8	−96.5	−96.0	−114.8	−114.2	−99.7	−99.1	−88.0	−87.5
Sampsone A (**490**)	−102.4	−101.3	−102.1	−100.9	−71.2	−70.4	−89.3	−88.3	−67.7	−67.0
Sarothralen B (**491**)	−40.1	−34.8	−99.2	−86.2	−96.1	−83.5	−99.6	−86.6	−70.4	−61.2
Sarothralen C (**492**)	−54.5	−46.8	−31.4	−27.0	−115.6	−99.4	−115.3	−99.1	−61.0	−52.4
Sarothralen D (**493**)	−127.3	−109.5	−130.9	−112.6	−119.5	−102.7	−117.1	−100.7	−104.1	−89.5
Shikonofuran C (**494**)	−133.7	−135.3	−103.4	−104.7	−123.6	−125.1	−102.4	−103.7	−109.1	−110.4
Shikonofuran D (**495**)	−126.6	−129.9	−110.3	−113.1	−126.9	−130.1	−103.4	−106.1	−105.3	−108.0
Shikonofuran E (**496**)	−126.6	−128.4	−113.5	−115.1	−134.9	−136.8	−105.2	−106.7	−109.5	−111.1
Sinapic acid (**497**)	−100.7	−119.2	−83.6	−98.9	−106.4	−125.9	−89.9	−106.4	−92.4	−109.4
Walrycin A (**498**)	−75.6	−97.4	−66.0	−85.0	−75.8	−97.5	−64.8	−83.4	−71.6	−92.2
**Quinones**										
2,6-Dimethoxy-1,4-benzoquinone (**336**)	−77.5	−101.0	−66.2	−86.3	−80.9	−105.4	−61.5	−80.1	−63.7	−82.9
2-Methyl-6-prenyl-1,4-benzoquinone (**337**)	−86.8	−108.5	−80.2	−100.3	−90.5	−113.1	−81.8	−102.2	−78.9	−98.7
Omphalone (**499**)	−91.7	−115.1	−79.2	−99.3	−87.4	−109.7	−78.2	−98.2	−81.9	−102.7
Primin (**500**)	−88.9	−107.9	−89.0	−108.0	−92.3	−112.0	−79.0	−95.8	−78.6	−95.4
1,4-Naphthoquinone (**338**)	−71.2	−94.6	−64.6	−85.9	−69.4	−92.2	−57.8	−76.8	−66.9	−88.9
2-Acetylnaphtho[2,3-*b*]furan-4,9-dione (**339**)	−98.4	−113.8	−91.9	−106.3	−97.3	−112.6	−82.4	−95.3	−93.1	−107.7
Alkannin (**340**)	−103.1	−112.2	−94.3	−102.6	−97.0	−105.5	−88.4	−96.2	−60.7	−66.1
Isobutyrylshikonin (**341**)	−122.6	−124.1	−101.9	−103.2	−115.6	−117.0	−90.3	−91.4	−74.4	−75.3
Lapachol (**501**)	−95.5	−110.1	−90.4	−104.3	−91.7	−105.8	−75.2	−86.7	−86.9	−100.3
Mamegakinone (**502**)	−112.2	−111.9	−89.4	−89.2	−111.0	−110.7	−87.8	−87.6	−83.8	−83.6
Menadione (**503**)	−75.5	−97.6	−70.0	−90.4	−72.4	−93.6	−61.6	−79.6	−70.6	−91.3
Rhinacanthin C (**504**)	−145.5	−140.7	−99.2	−96.0	−143.5	−138.8	−104.8	−101.4	−97.3	−94.1
Rhinacanthin D (**505**)	−145.7	−141.2	−98.3	−95.2	−151.8	−147.1	−104.8	−101.6	−107.6	−104.2
Rhinacanthin G (**506**)	−142.4	−136.0	−111.1	−106.2	−147.4	−140.8	−102.9	−98.3	−95.8	−91.5
Rhinacanthin H (**507**)	−155.9	−148.9	−107.4	−102.6	−140.9	−134.6	−104.7	−100.0	−101.1	−96.6
Rhinacanthin I (**508**)	−142.1	−135.7	−119.4	−114.1	−146.1	−139.6	−104.6	−99.9	−108.7	−103.9
Rhinacanthin J (**509**)	−137.0	−131.1	−98.0	−93.7	−153.2	−146.5	−110.6	−105.8	−111.7	−106.9
Rhinacanthin K (**510**)	−145.0	−136.6	−108.8	−102.5	−142.7	−134.4	−113.5	−106.9	−104.8	−98.7
Rhinacanthin L (**511**)	−146.7	−136.6	−102.7	−95.6	−146.1	−136.0	−110.6	−103.0	−97.9	−91.2
Rhinacanthin M (**512**)	−133.5	−134.4	−110.1	−110.8	−131.2	−132.1	−94.2	−94.8	−92.3	−93.0
Shikonin acetate (**513**)	−110.7	−115.2	−91.7	−95.3	−109.2	−113.6	−99.7	−103.7	−89.0	−92.5
β,β-Dimethylacrylshikonin (**514**)	−125.9	−126.0	−106.0	−106.2	−122.4	−122.6	−99.5	−99.7	−77.4	−77.5
β-Hydroxyisovaleryshikonin (**515**)	−123.2	−121.4	−104.1	−102.6	−124.3	−122.5	−101.6	−100.1	−99.3	−97.9
1-Hydroxy-3-hydroxymethylanthraquinone (**516**)	−95.5	−108.4	−84.0	−95.3	−92.7	−105.2	−79.7	−90.5	−84.5	−95.9
Aloeemodin (**518**)	−96.3	−107.1	−85.3	−94.9	−96.2	−107.0	−80.0	−89.0	−85.6	−95.2
Islandicin (**519**)	−91.5	−101.7	−80.1	−89.1	−87.7	−97.6	−75.2	−83.6	−70.8	−78.8
Newbouldiaquinone (**521**)	−95.3	−93.4	−93.4	−91.6	−96.9	−95.0	−93.8	−92.0	−86.7	−85.0
Newbouldiaquinone A (**520**)	−132.6	−128.3	−88.9	−86.0	−133.8	−129.5	−106.6	−103.1	−94.9	−91.9
Rhein (**522**)	−101.3	−110.8	−91.0	−99.5	−101.3	−110.8	−80.8	−88.4	−95.8	−104.8
15,16-Dihydrotanshinone I (**517**)	−96.1	−105.8	−80.7	−88.9	−93.2	−102.6	−74.5	−82.1	−72.5	−79.8
**Acetylene, Glucoside, and Other Miscellaneous Phytochemicals**										
1,7-Diphenyl-4-(2-phenylethyl)-1-heptene-3,5-dione (**530**)	−138.6	−137.3	−108.3	−107.3	−135.2	−133.9	−115.7	−114.6	−103.7	−102.8
1,7-Diphenyl-5-hepten-3-one (**531**)	−107.5	−120.4	−100.8	−112.9	−110.4	−123.7	−88.4	−99.0	−87.3	−97.8
3′-Demothexycyclocurcumin (**532**)	−122.3	−126.2	−91.0	−93.9	−121.0	−124.9	−112.7	−116.2	−99.0	−102.2
5,7-Dihydroxyphthalide (**533**)	−74.6	−97.6	−64.7	−84.6	−75.0	−98.1	−72.3	−94.5	−73.2	−95.7
6-Methyl-4,5-dithia-2-octene (**534**)	−66.5	−87.6	−60.1	−79.2	−67.5	−89.0	−57.5	−75.8	−61.2	−80.7
7-Epiclusianone (**535**)	−105.3	−95.2	−102.8	−92.9	−101.1	−91.4	−105.1	−95.1	−97.4	−88.1
Allamandin (**536**)	−103.4	−110.1	−101.6	−108.1	−99.3	−105.7	−95.2	−101.3	−81.8	−87.1
Allicin (**537**)	−72.4	−95.4	−61.9	−81.6	−69.8	−92.0	−59.7	−78.6	−65.3	−86.0
Amadannulen (**538**)	−110.1	−109.6	−102.9	−102.5	−82.5	−82.1	−99.7	−99.3	−110.5	−110.0
Anemonin (**539**)	−86.9	−108.3	−65.0	−81.0	−90.4	−112.7	−80.5	−100.3	−41.5	−51.7
Antibiotic CZ 34 (**540**)	−85.8	−102.2	−83.0	−98.8	−87.1	−103.7	−79.5	−94.6	−80.2	−95.4
Argutone (**541**)	−83.6	−105.3	−68.8	−86.6	−81.2	−102.3	−77.0	−97.0	−78.7	−99.1
Bakuchiol (**542**)	−103.0	−116.6	−90.8	−102.8	−103.6	−117.2	−89.6	−101.4	−94.5	−107.0
Brasiliensophyllic acid A (**543**)	−101.8	−88.7	−101.7	−88.7	−93.0	−81.1	−121.4	−105.8	−72.6	−63.3
Brasiliensophyllic acid C (**544**)	−110.4	−95.4	−108.3	−93.6	−96.8	−83.7	−129.6	−112.1	−105.9	−91.6
Centrolobin (**545**)	−116.5	−123.4	−94.4	−100.0	−119.7	−126.8	−88.9	−94.2	−93.5	−99.0
Chamone I (**546**)	−102.3	−88.6	−107.6	−93.3	−109.9	−95.3	−111.1	−96.3	−97.7	−84.7
Chamone II (**547**)	−109.3	−94.9	−104.3	−90.5	no dock	no dock	−108.4	−94.1	−66.3	−57.5
Champanone A (**548**)	−105.8	−119.8	−91.8	−104.0	−105.5	−119.5	−86.2	−97.5	−91.8	−103.9
Dhelwangin (**549**)	−94.5	−111.9	−94.5	−111.8	−100.7	−119.2	−78.0	−92.3	−82.8	−98.0
Garcinoic acid (**550**)	−148.8	−142.1	−106.8	−102.0	−151.0	−144.2	−131.6	−125.7	−103.9	−99.3
Ginkgolide A (**551**)	−48.2	−46.7	−91.8	−88.9	−51.4	−49.8	−96.8	−93.8	−36.7	−35.6
Guttiferone E (**552**)	−90.7	−77.2	−119.6	−101.8	−116.8	−99.4	−136.4	−116.1	−102.5	−87.3
Helipyrone B (**553**)	−102.4	−109.2	−94.8	−101.1	−104.1	−111.0	−78.7	−84.0	−77.0	−82.1
Helipyrone C (**554**)	−97.0	−105.1	−91.3	−98.9	−98.0	−106.1	−73.2	−79.3	−64.0	−69.3
Ialibinone A (**555**)	−86.5	−88.8	−84.5	−86.7	−85.0	−87.2	−87.0	−89.2	−77.9	−79.9
Ialibinone B (**556**)	−56.5	−57.9	−81.3	−83.4	−30.3	−31.0	−87.6	−89.9	−70.1	−71.9
Ialibinone C (**557**)	−93.8	−94.9	−89.0	−90.1	−94.0	−95.1	−94.9	−96.0	−87.7	−88.7
Ialibinone D (**558**)	−38.3	−38.8	−91.8	−92.9	−81.6	−82.6	−86.5	−87.6	−79.8	−80.8
Isobrasiliensophyllic acid A (**559**)	−97.3	−84.8	−103.3	−90.0	−96.0	−83.7	−131.0	−114.2	−83.2	−72.6
Moskachan C (**560**)	−92.1	−109.3	−90.5	−107.5	−95.0	−112.8	−86.1	−102.2	−83.4	−99.0
Nimbolide (**561**)	−37.6	−34.9	−77.1	−71.4	−67.9	−63.0	−107.4	−99.5	−82.6	−76.6
Pectinolide H (**562**)	−112.0	−124.8	−98.6	−109.9	−117.0	−130.4	−93.2	−103.9	−97.7	−108.9
Propolone A (**563**)	−107.8	−97.5	−100.7	−91.1	−70.1	−63.4	−108.5	−98.1	−78.0	−70.6
Sellovicine B (**564**)	−98.7	−115.1	−93.4	−108.9	−98.2	−114.5	−79.9	−93.2	−88.5	−103.2
Simonin A (**565**)	−109.7	−105.6	−94.3	−90.8	−102.8	−99.0	−113.8	−109.5	−85.5	−82.3
Tenulin (**566**)	−102.9	−109.7	−74.4	−79.4	−101.6	−108.3	−82.0	−87.5	−66.7	−71.1
Atractylodin (**522**)	−93.5	−118.6	−86.5	−109.6	−92.2	−116.9	−75.8	−96.1	−80.7	−102.4
Atractylodinol (**523**)	−100.8	−124.3	−91.0	−112.2	−97.4	−120.1	−84.5	−104.2	−85.4	−105.3
Capillene (**342**)	−71.8	−96.3	−68.5	−91.8	−70.1	−94.0	−65.1	−87.2	−71.6	−96.0
Peniophorin A (**524**)	−116.7	−127.9	−97.5	−106.9	−112.5	−123.3	−95.5	−104.7	−89.5	−98.1
Peniophorin B (**525**)	−97.9	−118.0	−89.8	−108.3	−104.4	−125.8	−86.9	−104.8	−88.6	−106.8
Thiarubrin A (**526**)	−93.0	−109.4	−92.0	−108.2	−90.0	−105.8	−73.2	−86.1	−75.1	−88.4
Arbutin (**527**)	−93.4	−103.6	−86.0	−95.4	−89.1	−98.9	−87.1	−96.6	−90.3	−100.2
Aucubin (**528**)	−105.2	−107.7	−98.2	−100.5	−110.1	−112.7	−93.1	−95.3	−79.9	−81.8
Diospyrodin (**529**)	−122.2	−129.2	−101.7	−107.5	−105.4	−111.5	−97.3	−102.9	−96.2	−101.8
**Known/Synthetic Inhibitors**										
(−)-Epicatechin	−95.4	−103.6	−90.6	−98.3	−93.5	−101.6	-	-	-	-
(−)-Epicatechin gallate	−140.4	−132.5	−87.6	−82.6	−143.4	−135.3	-	-	-	-
(−)-Epigallocatechin	−95.9	−102.3	−91.8	−97.9	−98.5	−105.0	-	-	-	-
(−)-Epigallocatechin 3-gallate	−141.3	−131.8	−90.8	−84.7	−142.9	−133.3	-	-	-	-
Norfloxacin	−123.6	−130.0	−94.0	−98.9	−112.3	−118.2	-	-	-	-
Novobiocin	−119.7	−101.2	−114.2	−96.6	−120.7	−102.1	-	-	-	-
Quercetin (**272**)	−102.9	−110.3	−94.1	−100.8	−96.8	−103.7	-	-	-	-
3461-2296 [97] (**573**)	-	-	-	-	-	-	−99.3	−102.5	-	-
4236-0754 [97] (**574**)	-	-	-	-	-	-	−102.1	−101.3	-	-
5591-1074 [97] (**575**)	-	-	-	-	-	-	−115.1	−110.7	-	-
C609-0168 [97] (**576**)	-	-	-	-	-	-	−104.2	−98.4	-	-
C609-0177 [97] (**577**)	-	-	-	-	-	-	−110.9	−100.0	-	-
C609-0383 [97] (**578**)	-	-	-	-	-	-	−128.2	−117.5	-	-

^a^ Compounds shown in red font violate Lipinski’s rule-of-five [62].

**Table 4:** MolDock molecular docking energies (E_dock_, kJ/mol) and normalized docking scores (DS_norm_) for the antibacterial phytochemical ligands with *Mycobacterium tuberculosis* UDP-galactopyranose mutase, *M. tuberculosis* cytochrome P450 CYP121, and bacterial DNA ligases.

Ligand	MtUGM		MtCYP121		EcLigA		MtLigA		SaLigA	
	E_dock_	DS_norm_	E_dock_	DS_norm_	E_dock_	DS_norm_	E_dock_	DS_norm_	E_dock_	DS_norm_
**Indole Alkaloids**										
1-Hydroxy-6,7-dimethoxy-3-methylcarbazole (**1**)	−80.2	−90.7	−93.5	−105.7	−91.7	−103.7	−84.7	−95.7	−99.7	−112.7
11-Methoxytubotaiwine (**2**)	−82.4	−83.7	−91.6	−93.0	−113.5	−115.3	−86.8	−88.2	−88.9	−90.3
12-Methoxy-4-methylvoachalotine (**3**)	−98.3	−95.0	−91.6	−88.6	−103.3	−99.8	−68.4	−66.1	−88.4	−85.5
3-Prenylindole (**4**)	−77.8	−98.1	−84.1	−106.1	−78.8	−99.4	−82.0	−103.4	−86.8	−109.5
Affinisine (**5**)	−84.8	−90.2	−83.4	−88.8	−97.0	−103.2	−66.4	−70.7	−89.9	−95.7
Apparicine (**6**)	−82.4	−92.3	−78.8	−88.2	−87.2	−97.7	−78.1	−87.5	−82.9	−92.8
Aristolactam I (**7**)	−95.0	−102.8	−98.9	−107.1	−101.9	−110.3	−91.4	−98.9	−107.1	−115.9
Clausenawalline A ^a^ (**8**)	no dock	no dock	−110.2	−96.3	−103.7	−90.7	−64.2	−56.1	−90.4	−79.0
Cryptoheptine (**9**)	−85.2	−95.7	−82.6	−92.8	−88.4	−99.2	−67.3	−75.6	−95.6	−107.3
Diploceline (**10**)	−82.0	−82.1	−97.4	−97.6	−112.5	−112.8	−96.5	−96.7	−54.6	−54.7
Discarine B (**11**)	−91.2	−78.9	−138.3	−119.7	−55.6	−48.1	−89.1	−77.1	−116.2	−100.5
Ibogamine (**12**)	−84.5	−92.8	−83.5	−91.7	−88.9	−97.6	−87.7	−96.4	−87.5	−96.1
Iboxygaine (**13**)	−101.4	−105.8	−96.1	−100.3	−104.7	−109.3	−98.0	−102.3	−103.3	−107.9
Isovoacangine (**14**)	−94.8	−95.0	−96.3	−96.6	−106.1	−106.4	−82.0	−82.3	−88.9	−89.2
Rugosanine B (**15**)	−45.3	−38.2	−139.7	−117.7	−128.5	−108.2	−111.9	−94.2	−123.4	−104.0
Suaveolindole (**16**)	−103.3	−105.1	−102.6	−104.4	−109.3	−111.2	−99.0	−100.7	−98.4	−100.1
Toussaintine B (**17**)	−92.9	−99.8	−87.6	−94.2	−97.4	−104.7	−94.5	−101.6	−108.3	−116.4
**Isoquinoline Alkaloids**										
8-Acetonyldihydroavicine (**18**)	−103.4	−100.4	−101.8	−98.8	−108.6	−105.5	−99.1	−96.3	−101.6	−98.7
8-Acetonyldihydronitidine (**19**)	−96.2	−94.7	−102.1	−100.5	−116.2	−114.5	−90.8	−89.4	−99.6	−98.0
Antofine (**20**)	−67.2	−67.7	−103.0	−103.7	−112.8	−113.7	−96.1	−96.9	−116.8	−117.7
Berbamine (**24**)	no dock	no dock	−48.9	−41.5	−80.8	−68.6	−58.9	−50.0	−64.0	−54.3
Berberine (**21**)	−100.6	−104.0	−96.1	−99.4	−95.5	−98.8	−97.8	−101.1	−99.0	−102.3
Bisnorthalphenine (**22**)	−84.6	−88.6	−92.6	−97.0	−105.0	−110.0	−87.9	−92.1	−105.9	−111.0
Cepharanthine (**25**)	no dock	no dock	−107.2	−91.0	no dock	no dock	−84.3	−71.6	−80.0	−68.0
Cryptopleurine (**23**)	−71.2	−70.8	−101.3	−100.8	−109.7	−109.2	−91.6	−91.1	−116.1	−115.5
Emetine (**26**)	−99.7	−91.5	−111.8	−102.7	−115.3	−105.8	−97.0	−89.0	−78.6	−72.1
Hydrastine (**27**)	−108.1	−107.0	−108.0	−106.9	−113.9	−112.7	−108.4	−107.2	−106.8	−105.7
Isotrilobine (**29**)	no dock	no dock	−140.7	−121.6	−75.4	−65.1	−66.2	−57.2	−61.9	−53.5
Jatrorrhizine (**28**)	−91.8	−94.8	−98.8	−101.9	−93.9	−96.9	−94.0	−97.0	−100.2	−103.4
Lauroscholtzine (**31**)	−90.5	−93.1	−92.2	−94.8	−102.7	−105.7	−87.3	−89.9	−95.3	−98.1
Methothalistyline (**30**)	−99.6	−80.1	−141.2	−113.7	−110.4	−88.8	−96.0	−77.3	−113.7	−91.5
*N*-Demethylthalphenine (**32**)	−83.8	−86.6	−84.4	−87.1	−106.8	−110.3	−93.1	−96.2	−106.7	−110.2
Obamegine (**34**)	no dock	no dock	−105.4	−90.1	−41.8	−35.8	−82.4	−70.4	−72.2	−61.7
Oxyacanthine (**35**)	no dock	no dock	−125.4	−106.4	−85.8	−72.8	−58.8	−49.9	−88.1	−74.7
Pennsylvanine (**36**)	−76.5	−62.4	−134.2	−109.6	−140.8	−114.9	−101.2	−82.6	−124.9	−102.0
Thaliadanine (**38**)	−98.9	−79.5	−132.1	−106.2	−155.7	−125.2	−103.6	−83.3	−139.6	−112.2
Thalicarpine (**37**)	−75.0	−60.8	−110.1	−89.3	−119.0	−96.5	−96.2	−78.0	−124.3	−100.8
Thalidasine (**39**)	no dock	no dock	−103.1	−85.4	no dock	no dock	−64.0	−53.0	−96.6	−80.1
Thalistyline (**40**)	−58.8	−47.7	−159.7	−129.4	−145.7	−118.1	−101.9	−82.6	−116.1	−94.1
Thalmelatine (**41**)	−44.0	−36.0	−130.7	−106.7	−134.7	−109.9	−102.3	−83.6	−115.7	−94.5
Thalmirabine (**42**)	no dock	no dock	−20.7	−17.0	no dock	no dock	−101.7	−83.6	−70.2	−57.7
Thalphenine (**33**)	−86.5	−88.0	−94.8	−96.5	−110.8	−112.8	−86.7	−88.3	−113.7	−115.8
Thalrugosidine (**43**)	no dock	no dock	−96.5	−80.6	no dock	no dock	−78.1	−65.2	−93.4	−78.0
Thalrugosine (**44**)	no dock	no dock	−119.6	−101.5	−47.1	−40.0	−89.9	−76.3	−73.9	−62.7
**Piperidine, Pyrrole, Pyrrolizidine, Quinoline, and Steroidal Alkaloids**										
Aconicaramide (**46**)	−79.7	−94.6	−83.7	−99.4	−84.2	−99.9	−83.0	−98.6	−95.6	−113.4
Lasiocarpine (**47**)	−119.3	−115.3	−123.0	−118.9	−119.0	−115.0	−95.6	−92.4	−118.7	−114.8
Lasiocarpine *N*-oxide (**48**)	−122.3	−116.7	−106.5	−101.7	−122.5	−116.9	−89.5	−85.4	−114.0	−108.8
Piperine (**45**)	−94.7	−103.4	−105.3	−115.0	−100.4	−109.7	−99.8	−109.0	−105.8	−115.5
4-Methoxy-1-methyl-2(1*H*)-quinolinone (**49**)	−61.8	−77.4	−75.7	−94.8	−70.9	−88.8	−61.3	−76.8	−75.2	−94.1
Cryptolepine (**50**)	−70.3	−82.2	−84.6	−99.0	−89.9	−105.1	−73.6	−86.1	−85.1	−99.5
Neocryptolepine (**51**)	−73.3	−85.7	−84.7	−99.0	−84.0	−98.2	−72.4	−84.6	−87.5	−102.3
Pteleine (**52**)	−75.0	−88.1	−76.8	−90.3	−81.8	−96.1	−78.3	−92.0	−88.1	−103.5
Veprisinium (**53**)	−93.4	−96.7	−79.4	−82.2	−102.1	−105.7	−82.7	−85.6	−92.4	−95.8
Conessine (**54**)	−94.3	−95.6	−92.2	−93.5	−88.0	−89.3	−76.3	−77.4	−78.5	−79.6
Irehdiamine A (**55**)	−87.0	−91.8	−90.3	−95.2	−84.5	−89.1	−79.0	−83.4	−82.6	−87.1
Solacassine (**56**)	−59.2	−55.9	−88.1	−83.1	−105.2	−99.2	−73.6	−69.4	−71.9	−67.8
Solanocapsine (**57**)	−72.0	−68.6	−100.0	−95.2	−104.4	−99.4	−83.9	−79.9	−83.6	−79.6
Tomatidine (**58**)	−63.1	−60.9	−86.8	−83.7	−94.0	−90.6	−84.5	−81.5	−85.2	−82.2
**Miscellaneous Alkaloids**										
2-(Methoxyamino)-4*H*-1-benzopyran-3,4,5,7-tetrol (**59**)	−86.1	−99.4	−91.0	−105.1	−88.4	−102.1	−84.3	−97.4	−92.5	−106.8
Abyssenine C (**60**)	−86.7	−81.7	−108.1	−101.8	−112.6	−106.0	−89.2	−84.0	−98.3	−92.6
Amphibine H (**61**)	−13.3	−11.3	−146.3	−124.3	−99.4	−84.5	−99.4	−84.5	−70.2	−59.7
Cepharatine A (**62**)	−87.5	−92.7	−85.6	−90.6	−93.4	−98.9	−70.2	−74.3	−95.2	−100.7
Curcamide (**63**)	−94.1	−107.9	−97.6	−111.9	−97.1	−111.4	−101.0	−115.8	−108.6	−124.6
Drodrenin (**64**)	−148.0	−133.6	−133.2	−120.2	−144.1	−130.1	−138.5	−125.0	−158.0	−142.6
Eschscholtzidine (**65**)	−88.2	−90.9	−91.0	−93.8	−97.0	−100.0	−86.0	−88.6	−94.4	−97.3
Jervine (**66**)	−32.2	−30.8	−87.8	−84.0	−80.0	−76.4	−94.9	−90.7	−89.5	−85.5
Matrine (**67**)	−75.9	−86.9	−65.2	−74.6	−86.2	−98.6	−71.9	−82.3	−89.5	−102.4
Mucronine H (**68**)	−69.0	−63.4	−118.4	−108.8	−126.1	−115.9	−95.6	−87.9	−119.6	−109.9
*N*-Benzoylmescaline (**69**)	−97.8	−103.3	−100.3	−106.0	−104.6	−110.5	−97.8	−103.3	−115.5	−122.0
Nummularine B (**70**)	no dock	no dock	−148.7	−127.4	−135.6	−116.2	−101.4	−86.9	−81.5	−69.8
Nummularine S (**71**)	−37.6	−33.6	−122.1	−109.2	−131.4	−117.4	−109.9	−98.2	−114.2	−102.1
Scutianine B (**72**)	−20.2	−17.5	−127.6	−110.8	−86.5	−75.1	−95.8	−83.1	−93.0	−80.7
Shahidine (**73**)	−94.7	−104.2	−104.7	−115.2	−104.5	−114.9	−109.1	−120.0	−116.1	−127.7
Thaliglucinone (**74**)	−96.8	−97.3	−106.1	−106.7	−125.1	−125.8	−96.4	−97.0	−123.8	−124.5
Triisopenylguanidine (**75**)	−94.6	−105.4	−91.8	−102.2	−98.7	−109.8	−103.8	−115.5	−107.9	−120.1
Tuberine (**76**)	−133.6	−123.6	−124.6	−115.3	−124.3	−115.0	−122.7	−113.5	−126.7	−117.2
**Monoterpenoids**										
Linalool (**77**)	−64.8	−86.9	−66.7	−89.4	−64.7	−86.7	−72.4	−97.0	−73.5	−98.5
Thymol (**78**)	−60.7	−82.1	−60.7	−82.0	−63.7	−86.2	−63.3	−85.7	−72.7	−98.3
Thymoquinol (**79**)	−65.8	−86.1	−66.8	−87.4	−70.0	−91.6	−65.9	−86.2	−78.1	−102.1
β-Dolabrin (**80**)	−70.7	−93.2	−73.0	−96.2	−73.8	−97.4	−73.3	−96.6	−76.0	−100.2
β-Thujaplicin (**81**)	−70.5	−92.6	−71.8	−94.3	−71.7	−94.1	−73.4	−96.4	−75.1	−98.6
**Sesquiterpenoids**										
11,13-Dehydroeriolin (**82**)	−91.9	−103.0	−77.5	−86.9	−82.3	−92.2	−73.4	−82.3	−81.8	−91.6
2,10-Bisaboladien-1-one (**83**)	−80.7	−96.1	−84.0	−100.0	−84.1	−100.1	−87.4	−104.1	−93.1	−110.9
2-Hydroxycalamenene (**84**)	−71.5	−85.4	−67.2	−80.2	−76.4	−91.2	−66.6	−79.5	−74.3	−88.7
2-Methoxyfurano-9-guaien-8-one (**85**)	−85.7	−96.5	−71.7	−80.8	−94.1	−106.0	−83.7	−94.3	−90.2	−101.6
4α,10α-Dihydroxy-1,11(13)guaiadien-12,8-olide (**93**)	−89.3	−100.1	−80.6	−90.3	−83.3	−93.3	−79.6	−89.1	−82.7	−92.6
4α,10β-Dihydroxy-1,11(13)guaiadien-12,8-olide (**89**)	−91.6	−102.7	−78.9	−88.4	−85.9	−96.2	−77.3	−86.5	−81.0	−90.8
Alantolactone (**86**)	−73.4	−85.9	−65.7	−76.8	−73.9	−86.4	−55.5	−65.0	−46.6	−54.5
Alliacol A (**87**)	−76.5	−85.8	−70.3	−78.8	−78.1	−87.5	−68.3	−76.6	−67.6	−75.8
Alliacol B (**88**)	−76.7	−85.9	−74.6	−83.6	−81.0	−90.8	−67.0	−75.1	−73.5	−82.4
Artemisinic acid (**113**)	−78.3	−91.3	−71.3	−83.1	−76.3	−88.9	−76.2	−88.8	−75.1	−87.6
Baileyolin (**90**)	−99.8	−100.6	−99.9	−100.7	−105.9	−106.8	−96.8	−97.7	−88.2	−88.9
Bilobalide A (**91**)	−90.1	−94.1	−84.9	−88.7	−93.5	−97.6	−59.4	−62.0	−67.4	−70.3
Confertin (**92**)	−88.6	−101.4	−70.8	−81.0	−79.5	−91.0	−60.7	−69.4	−74.3	−85.0
Cyperenal (**94**)	−70.5	−84.2	−56.1	−67.0	−65.5	−78.2	−52.8	−63.0	−48.1	−57.5
Cyperenol (**95**)	−71.6	−85.3	−59.7	−71.0	−68.2	−81.1	−55.3	−65.8	−45.0	−53.6
Furanodienone (**97**)	−88.7	−104.0	−77.1	−90.5	−84.2	−98.8	−67.9	−79.6	−73.5	−86.2
Ganodermycin (**96**)	−101.0	−110.9	−90.2	−99.1	−97.0	−106.6	−97.4	−107.0	−111.3	−122.3
Helenalin (**98**)	−90.4	−101.6	−70.3	−78.9	−84.6	−95.0	−67.2	−75.5	−54.8	−61.5
Hydrogrammic acid (**99**)	−87.2	−98.2	−80.1	−90.2	−88.2	−99.3	−83.5	−94.0	−83.5	−94.0
Isoalantolactone (**100**)	−78.2	−91.4	−65.3	−76.4	−74.7	−87.4	−54.7	−64.0	−70.6	−82.6
Ivaxillin (**101**)	−88.1	−98.4	−76.0	−84.9	−80.6	−90.1	−72.5	−81.1	−77.1	−86.1
Petrovin A (**102**)	−87.4	−100.0	−70.5	−80.6	−71.9	−82.3	−74.8	−85.6	−66.6	−76.2
Petrovin B (**103**)	−81.9	−93.4	−78.5	−89.5	−73.2	−83.5	−76.1	−86.8	−56.3	−64.3
Polygodial (**104**)	−78.2	−91.2	−67.9	−79.2	−76.6	−89.4	−67.8	−79.0	−73.3	−85.5
Rishitin (**105**)	−72.7	−86.3	−79.0	−93.8	−87.2	−103.5	−78.5	−93.1	−86.6	−102.8
Xanthorrhizol (**106**)	−82.0	−97.9	−82.2	−98.1	−82.1	−98.0	−87.1	−104.0	−92.7	−110.7
α-Amorphene (**107**)	−67.1	−81.9	−64.6	−78.8	−68.5	−83.6	−71.7	−87.5	−62.8	−76.7
α-Cadinene (**108**)	−68.9	−84.2	−72.5	−88.5	−73.1	−89.2	−66.9	−81.6	−73.3	−89.5
α-Copaene (**110**)	−61.0	−76.3	−56.4	−70.5	−56.3	−70.4	−56.4	−70.5	−54.4	−68.0
α-Muurolene (**109**)	−68.1	−83.1	−72.2	−88.2	−68.8	−84.0	−66.3	−80.9	−68.9	−84.2
γ-Cadinene (**112**)	−70.6	−86.2	−69.5	−84.8	−74.0	−90.3	−72.1	−88.0	−71.3	−87.1
**Diterpenoids**										
1,12-Diacetyljativatriol (**114**)	−82.4	−80.1	−92.1	−89.5	−112.9	−109.7	−82.5	−80.2	−85.1	−82.8
12-Oxo-3,13(16)-clerodadien-15-oic acid (**115**)	−99.6	−104.9	−87.3	−91.9	−97.4	−102.6	−88.6	−93.3	−89.0	−93.7
12-Oxo-8,13(16)-clerodadien-15-oic acid (**116**)	−98.8	−104.0	−90.9	−95.7	−95.2	−100.2	−94.8	−99.8	−89.6	−94.3
13-Epimanoyl oxide (**117**)	−79.6	−86.4	−68.1	−74.0	−73.9	−80.2	−59.1	−64.1	−55.8	−60.6
13-Episclareol (**118**)	−91.9	−97.8	−80.1	−85.3	−90.2	−96.0	−84.7	−90.1	−39.4	−41.9
3,4-Seco-4(18)-trachyloben-3-oic acid (**120**)	−88.2	−94.5	−88.4	−94.6	−80.7	−86.4	−86.1	−92.3	−80.0	−85.7
3-Hydroxytotarol (**119**)	−78.8	−84.4	−79.9	−85.5	−85.9	−92.0	−64.1	−68.7	−81.2	−87.0
7,13-Labdadien-15-ol acetate (**121**)	−100.2	−104.0	−99.5	−103.3	−105.7	−109.7	−95.8	−99.4	−101.8	−105.7
7,13-Labdadien-15-ol malonate (**122**)	−117.9	−117.4	−109.5	−109.1	−120.5	−120.0	−113.1	−112.6	−96.1	−95.6
Acetylcrinipellin A (**125**)	−103.7	−103.6	−92.8	−92.7	−107.7	−107.7	−78.2	−78.2	−64.8	−64.8
Aethiopinone (**123**)	−94.6	−102.0	−87.8	−94.7	−108.2	−116.7	−93.2	−100.6	−109.1	−117.6
Andrographolide (**124**)	−103.2	−105.3	−95.2	−97.0	−104.8	−106.9	−97.1	−99.0	−99.2	−101.2
Biflorin (**126**)	−94.4	−102.0	−93.1	−100.7	−107.5	−116.2	−90.6	−97.9	−107.8	−116.5
Continentalic acid (**127**)	−78.1	−83.6	−75.9	−81.3	−85.1	−91.1	−61.4	−65.8	−84.7	−90.7
Crinipellin A (**129**)	−108.9	−113.3	−84.7	−88.0	−98.4	−102.4	−71.3	−74.1	−73.5	−76.4
Cryptobeilic acid A (**128**)	−103.4	−109.1	−98.0	−103.4	−103.1	−108.8	−97.9	−103.3	−118.1	−124.6
Cryptobeilic acid C (**130**)	−107.1	−107.8	−114.2	−114.9	−117.9	−118.7	−102.6	−103.3	−120.3	−121.0
Cryptobeilic acid D (**131**)	−98.3	−105.5	−99.7	−107.0	−103.1	−110.7	−102.5	−110.0	−107.0	−114.8
Effusanin A (**132**)	−78.1	−79.8	−73.1	−74.7	−81.6	−83.4	−70.9	−72.5	−43.9	−44.9
Effusanin B (**133**)	−75.8	−74.5	−78.2	−76.9	−92.7	−91.2	−89.9	−88.4	−65.7	−64.6
Effusanin C (**134**)	−95.9	−93.1	−82.0	−79.6	−96.1	−93.2	−44.8	−43.5	−61.5	−59.7
Effusanin D (**135**)	−88.4	−83.0	−96.4	−90.5	−109.0	−102.3	−72.8	−68.4	−71.0	−66.7
Effusanin E (**136**)	−81.2	−81.7	−72.7	−73.2	−83.7	−84.3	−55.0	−55.4	−42.0	−42.2
Grandiflorenic acid (**137**)	−76.5	−82.1	−77.4	−83.1	−77.2	−82.8	−73.2	−78.6	−49.8	−53.5
Haplociliatic acid (**138**)	−99.7	−103.1	−95.2	−98.5	−105.8	−109.3	−88.8	−91.8	−85.9	−88.8
Hypargenin A (**139**)	−82.5	−85.8	−83.6	−87.0	−94.0	−97.7	−77.1	−80.2	−74.1	−77.1
Hypargenin B (**140**)	−84.1	−88.7	−81.6	−86.1	−88.3	−93.2	−75.2	−79.3	−84.8	−89.4
Hypargenin D (**141**)	−79.9	−86.0	−82.1	−88.3	−88.6	−95.3	−76.0	−81.7	−88.5	−95.2
Hypargenin F (**142**)	−88.3	−91.8	−86.1	−89.5	−87.4	−90.9	−70.0	−72.8	−77.2	−80.3
Isodomedin (**143**)	−78.2	−76.8	−84.8	−83.3	−76.2	−74.8	−75.0	−73.7	−89.1	−87.5
Kamebanin (**144**)	−91.3	−94.5	−74.0	−76.7	−79.4	−82.2	−45.6	−47.2	−51.5	−53.3
Lasiokaurin (**145**)	−81.6	−79.1	−84.7	−82.1	−93.6	−90.7	−86.7	−84.0	−52.1	−50.5
Longikaurin A (**146**)	−83.1	−84.9	−71.8	−73.4	−78.1	−79.8	−66.7	−68.2	−62.9	−64.2
Longikaurin B (**147**)	−90.7	−88.1	−89.9	−87.3	−94.5	−91.7	−81.4	−79.0	−46.1	−44.7
Longikaurin C (**148**)	−80.8	−79.5	−87.8	−86.4	−96.6	−95.0	−51.6	−50.8	−72.9	−71.7
Longikaurin D (**149**)	−85.3	−82.8	−88.0	−85.4	−100.5	−97.6	−80.9	−78.6	−55.0	−53.4
Longikaurin E (**150**)	−83.4	−85.2	−72.7	−74.3	−81.0	−82.8	−64.5	−65.9	−33.4	−34.1
Longikaurin F (**151**)	−95.6	−89.8	−92.7	−87.1	−103.8	−97.5	−91.1	−85.6	−82.0	−77.1
Longikaurin G (**152**)	−86.7	−87.3	−75.6	−76.1	−81.9	−82.5	−71.8	−72.3	−62.5	−62.9
Lupulin E (**153**)	−103.9	−92.7	−111.5	−99.5	−124.8	−111.4	−89.3	−79.7	−91.8	−82.0
Lupulin F (**154**)	−44.3	−39.5	−108.0	−96.3	−124.3	−110.8	−77.5	−69.1	−91.1	−81.2
Methyl seconidoresedate (**155**)	−110.4	−115.0	−103.5	−107.8	−110.7	−115.3	−92.0	−95.8	−88.2	−91.9
Pisiferol (**156**)	−83.1	−89.0	−80.9	−86.6	−85.7	−91.7	−81.2	−87.0	−82.2	−88.1
Salvic acid (**157**)	−97.4	−102.1	−88.6	−92.9	−97.3	−102.0	−95.9	−100.6	−86.0	−90.2
Salvic acid acetate (**158**)	−96.2	−96.9	−94.6	−95.2	−109.2	−109.9	−99.3	−100.0	−96.0	−96.7
Shikokianin (**159**)	−59.4	−55.8	−86.5	−81.2	−101.4	−95.2	−83.1	−78.0	−57.1	−53.6
Strictic acid (**160**)	−104.7	−110.7	−98.2	−103.9	−108.4	−114.6	−89.8	−94.9	−88.5	−93.5
Taxodione (**161**)	−85.4	−90.3	−81.4	−86.0	−98.6	−104.3	−80.0	−84.6	−91.9	−97.2
Trichodonin (**162**)	−89.3	−86.8	−92.5	−89.9	−94.2	−91.6	−72.4	−70.4	−60.4	−58.7
Umbrosin A (**163**)	−85.9	−88.9	−76.1	−78.8	−81.7	−84.6	−55.3	−57.3	−61.1	−63.2
Umbrosin B (**164**)	−89.7	−93.1	−75.2	−78.0	−81.6	−84.7	−42.6	−44.3	−44.2	−45.8
Yuexiandajisu A (**165**)	−75.2	−79.2	−86.1	−90.6	−96.9	−102.1	−88.0	−92.6	−77.6	−81.7
**Triterpenoids**										
Alisol A 24-acetate (**166**)	−95.7	−84.9	−109.9	−97.5	−123.5	−109.5	−91.2	−80.9	−111.1	−98.5
Alisol B 23-acetate (**167**)	−79.4	−71.2	−112.2	−100.7	−114.5	−102.7	−99.8	−89.5	−107.9	−96.8
Betulinic acid (**168**)	−65.7	−61.3	−94.9	−88.6	−97.4	−90.9	no dock	no dock	−41.3	−38.6
Entagenic acid (**169**)	−24.8	−22.6	−96.6	−88.2	−82.1	−74.9	−61.4	−56.1	−38.4	−35.1
Lantic acid (**170**)	−31.6	−29.2	−83.6	−77.3	−92.6	−85.6	−57.1	−52.8	−35.8	−33.1
Mahmoodin (**171**)	−51.0	−45.4	−116.5	−103.7	−93.6	−83.4	−79.9	−71.2	−76.3	−67.9
Maslinic acid (**172**)	−54.7	−50.5	−94.9	−87.6	−83.1	−76.7	−64.1	−59.2	−67.9	−62.7
Oleanolic acid (**173**)	−29.6	−27.7	−92.0	−85.9	−75.2	−70.2	−34.9	−32.6	−59.6	−55.7
Pristimerin (**174**)	−72.9	−67.7	−104.7	−97.2	−79.8	−74.1	−36.1	−33.5	−78.1	−72.5
Rubrinol (**175**)	−36.0	−34.0	−86.7	−81.8	−94.3	−89.0	−23.0	−21.7	−37.9	−35.7
Tingenone (**176**)	−50.0	−48.0	−89.1	−85.5	−84.2	−80.8	−21.4	−20.6	−78.8	−75.7
**Chalcones**										
1-(2,6-Dihydroxy-4-methoxyphenyl)-3-phenyl-1-propanone (**177**)	−89.0	−98.7	−85.2	−94.5	−95.8	−106.3	−96.7	−107.3	−111.7	−123.9
2′-Hydroxy-2,3,4′,6′-tetramethoxychalcone (**178**)	−111.5	−114.4	−107.3	−110.0	−123.4	−126.6	−92.2	−94.6	−128.4	−131.7
3′′′′,5′′′,5′′′′′-Tribenzyl-2′′′′,2′′′′′,2′′′′′′-trihydroxyisodiuvaretin (**180**)	−122.3	−94.6	−158.0	−122.3	−70.8	−54.7	−124.0	−95.9	−129.4	−100.1
4'-Hydroxychalcone (**179**)	−80.9	−95.7	−87.4	−103.4	−88.6	−104.8	−90.7	−107.3	−99.2	−117.5
5″,5′′′′,5′′′′′-Tribenzyl-2′′′′,2′′′′′,2′′′′′′-trihydroxyisodiuvaretin (**181**)	−166.5	−128.8	−163.7	−126.6	−150.9	−116.8	−78.4	−60.6	−193.6	−149.8
Angusticornin B (**182**)	−133.8	−128.0	−147.1	−140.7	−149.2	−142.7	−127.8	−122.3	−150.4	−143.8
Balsacone A (**183**)	−126.9	−121.8	−138.3	−132.7	−140.6	−134.9	−123.4	−118.5	−157.2	−150.9
Balsacone B (**184**)	−128.3	−123.1	−144.8	−138.9	−143.3	−137.6	−122.8	−117.8	−155.1	−148.9
Balsacone C (**185**)	−126.1	−124.1	−132.9	−130.7	−134.9	−132.7	−114.8	−113.0	−143.7	−141.4
Bartericin C (**186**)	−111.4	−107.9	−113.1	−109.6	−119.2	−115.5	−106.9	−103.6	−114.2	−110.7
Bavachalcone (**187**)	−117.0	−120.7	−115.7	−119.4	−122.7	−126.6	−120.0	−123.8	−135.9	−140.2
Broussochalcone B (**188**)	−111.7	−116.9	−119.9	−125.5	−120.7	−126.3	−122.2	−127.9	−128.5	−134.4
Corylifol B (**189**)	−114.0	−117.4	−115.5	−118.9	−125.8	−129.6	−124.0	−127.7	−130.9	−134.8
Erythbidin C (**190**)	−108.0	−114.5	−104.4	−110.7	−111.8	−118.5	−109.7	−116.2	−119.5	−126.6
Helichrysone A (**191**)	−119.4	−121.3	−113.1	−114.9	−114.7	−116.5	−119.3	−121.2	−130.6	−132.7
Isobavachalcone (**192**)	−113.7	−119.0	−111.7	−116.9	−118.6	−124.1	−121.3	−126.9	−124.8	−130.6
Kanzonol C (**193**)	−132.3	−129.9	−136.2	−133.8	−140.2	−137.7	−135.3	−132.8	−144.8	−142.2
Kuraridin (**194**)	−132.0	−125.0	−141.7	−134.1	−148.8	−140.8	−129.7	−122.7	−150.7	−142.6
Myrigalone G (**195**)	−91.9	−100.2	−91.9	−100.3	−100.4	−109.5	−90.9	−99.2	−109.9	−119.9
Piperaduncin A (**196**)	−140.3	−127.9	−130.4	−118.8	−146.9	−133.9	−78.6	−71.7	−137.3	−125.2
Piperaduncin B (**197**)	−136.1	−122.7	−136.6	−123.2	−137.8	−124.3	−113.4	−102.3	−138.1	−124.5
Piperaduncin C (**198**)	−128.9	−112.6	−150.2	−131.3	−157.3	−137.5	−106.9	−93.4	−125.1	−109.3
Psorachalcone A (**199**)	−112.7	−116.1	−117.2	−120.7	−121.4	−125.0	−109.4	−112.7	−125.7	−129.4
Xanthoangelol (**200**)	−132.8	−130.4	−130.8	−128.4	−137.8	−135.3	−135.9	−133.4	−153.0	−150.3
Xanthoangelol F (**201**)	−132.6	−128.7	−122.4	−118.8	−143.9	−139.7	−114.5	−111.2	−142.7	−138.5
**Flavonoids**										
2′,5,5′,7-Tetrahydroxyflavanone (**202**)	−89.2	−97.1	−94.0	−102.3	−99.1	−107.9	−86.6	−94.3	−101.8	−110.8
2′,7-Dimethoxyflavone (**203**)	−86.1	−94.4	−94.0	−103.0	−95.3	−104.4	−94.8	−103.9	−100.0	−109.7
3′′′′-(2-Hydroxybenzyl)isouvarinol (**218**)	−151.2	−123.6	−179.4	−146.7	−143.0	−116.9	−130.3	−106.5	−143.3	−117.2
3′′′′-(2-Hydroxybenzyl)uvarinol (**217**)	−152.4	−124.6	−157.7	−128.9	−148.8	−121.6	−135.0	−110.4	−159.7	−130.6
3′-Methylpelargonidin (**204**)	−90.7	−99.1	−99.7	−108.9	−98.5	−107.6	−88.3	−96.5	−103.4	−113.0
3′-*O*-Methyldiplacone (**205**)	−136.8	−129.5	−131.4	−124.3	−139.0	−131.5	−122.3	−115.8	−140.4	−132.9
4′,5,7-Trihydroxy-6-methyl-8-prenylflavanone (**207**)	−102.0	−103.6	−97.2	−98.7	−109.0	−110.7	−100.4	−102.0	−121.6	−123.5
4′,5,7-Trihydroxy-8-methyl-6-prenylflavanone (**206**)	−109.4	−111.1	−98.9	−100.5	−113.4	−115.2	−95.2	−96.7	−119.2	−121.1
4′,5-Dihydroxy-7-methoxy-6-prenylflavanone (**208**)	−111.2	−113.0	−104.8	−106.5	−112.4	−114.2	−101.1	−102.7	−118.5	−120.4
4′,6,7-Trihydroxy-3′,5′-dimethoxyflavone (**209**)	−103.7	−107.8	−108.4	−112.7	−108.5	−112.9	−100.8	−104.8	−110.7	−115.1
4′,7-Dihydroxy-8-methylflavan (**210**)	−81.4	−92.2	−84.4	−95.5	−89.1	−100.9	−83.8	−94.8	−99.0	−112.0
4′-Hydroxy-5,7-dimethoxyflavone (**211**)	−93.2	−100.3	−101.0	−108.7	−100.1	−107.7	−95.9	−103.2	−104.9	−112.9
5″-(2-Hydroxybenzyl)isouvarinol (**216**)	−127.0	−103.8	−161.5	−132.0	−174.9	−142.9	−109.8	−89.7	−164.2	−134.2
5′-(1,1-Dimethyl-2-propenyl)-2′,4′,5,7-tetrahydroxy-6-prenylflavanone (**219**)	−126.8	−121.3	−117.1	−112.1	−115.9	−110.8	−115.3	−110.3	−136.9	−131.0
5′-(1,1-Dimethyl-2-propenyl)-2′,4′,5,7-tetrahydroxy-8-prenylflavanone (**220**)	−116.8	−111.7	−114.4	−109.4	−122.1	−116.8	−96.8	−92.6	−120.9	−115.7
5′-(1,1-Dimethyl-2-propenyl)-4′,5,7-trihydroxy-2′-methoxy-8-prenylflavanone (**221**)	−125.1	−118.4	−109.1	−103.2	−122.9	−116.3	−101.4	−96.0	−115.4	−109.2
5,6-Dihydroxy-4′,7,8-trimethoxyflavone (**212**)	−97.6	−100.1	−95.6	−98.1	−111.0	−113.9	−98.9	−101.4	−112.6	−115.5
5-Hydroxy-2′,4′,5′,7-Tetramethoxyflavone (**213**)	−108.2	−109.5	−111.3	−112.7	−112.5	−113.9	−103.4	−104.6	−109.8	−111.1
6,7-Dihydroxyflavone (**214**)	−80.2	−91.0	−91.6	−103.9	−94.2	−106.9	−86.1	−97.7	−98.0	−111.2
8-Methoxycirsilineol (**215**)	−107.5	−107.2	−98.5	−98.3	−115.2	−114.9	−98.1	−97.9	−111.5	−111.2
9,10-Dihydro-9,10-diacetoxy-3-methoxy-8,8-dimethyl-2-phenyl-4*H*,8*H*-benzo[1,2-*b*:3,4-*b*′]dipyran-4-one (**222**)	−84.0	−78.7	−102.4	−95.9	−118.3	−110.8	−93.6	−87.6	−100.3	−93.9
Abyssinone I (**223**)	−101.6	−106.5	−106.0	−111.1	−105.4	−110.5	−105.2	−110.4	−101.4	−106.3
Abyssinone IV (**224**)	−116.2	−114.1	−128.5	−126.2	−128.3	−126.0	−117.6	−115.5	−135.9	−133.5
Astragalin (**225**)	−118.8	−111.6	−114.5	−107.6	−143.0	−134.3	−118.3	−111.2	−136.1	−127.8
Bavachinin (**226**)	−106.7	−110.1	−104.6	−107.9	−112.1	−115.7	−94.8	−97.8	−117.6	−121.3
Betuletol (**227**)	−94.4	−98.2	−106.6	−110.8	−111.8	−116.3	−97.8	−101.7	−118.6	−123.4
Bonannione A (**228**)	−119.7	−116.0	−125.3	−121.4	−130.6	−126.5	−123.2	−119.4	−132.1	−128.0
Brosimone I (**229**)	−104.1	−99.9	−116.1	−111.4	−117.4	−112.7	−97.0	−93.1	−146.3	−140.4
Cassiaflavan (**230**)	−78.8	−90.9	−83.3	−96.1	−84.0	−96.9	−80.7	−93.0	−90.9	−104.8
Cerasinone (**231**)	−97.3	−101.2	−103.0	−107.1	−112.0	−116.5	−98.2	−102.1	−116.2	−120.9
Chrysin (**233**)	−82.6	−93.8	−91.7	−104.1	−94.7	−107.4	−86.3	−97.9	−95.1	−107.9
Chrysoeriol (**232**)	−95.7	−102.8	−100.3	−107.7	−109.1	−117.2	−97.8	−105.0	−106.7	−114.6
Corniculatusin (**234**)	−103.0	−106.9	−98.9	−102.7	−114.6	−119.0	−96.3	−100.0	−113.5	−117.8
Cudraflavone A (**235**)	−103.0	−99.0	−109.7	−105.5	−107.3	−103.2	−90.8	−87.3	−110.1	−105.8
Dihydroquercetin (**236**)	−99.2	−106.1	−98.4	−105.2	−103.5	−110.7	−92.6	−98.9	−103.6	−110.8
Eucalyptin (**237**)	−92.2	−96.3	−93.7	−97.9	−100.8	−105.3	−95.3	−99.5	−110.5	−115.4
Euchrestaflavanone A (**238**)	−123.0	−119.2	−115.6	−112.0	−139.0	−134.7	−115.0	−111.4	−123.5	−119.7
Flavaprenin (**239**)	−104.9	−108.0	−95.7	−98.6	−114.3	−117.7	−101.9	−104.9	−116.5	−120.0
Flemiflavanone D (**240**)	−129.1	−123.5	−117.3	−112.2	−139.5	−133.5	−112.4	−107.5	−126.6	−121.2
Glabranin (**241**)	−100.5	−105.2	−91.5	−95.7	−114.4	−119.7	−94.1	−98.4	−109.6	−114.7
Isoorientin (**243**)	−97.3	−91.4	−125.8	−118.2	−124.7	−117.2	−101.7	−95.6	−129.4	−121.6
Isoscoparin (**244**)	−103.1	−95.9	−134.5	−125.0	−125.6	−116.8	−98.8	−91.8	−127.7	−118.8
Kaempferol (**242**)	−94.3	−102.8	−97.6	−106.5	−101.8	−111.1	−87.0	−95.0	−101.9	−111.1
Kushenol A (**245**)	−104.3	−101.1	−112.1	−108.6	−127.3	−123.4	−108.5	−105.2	−122.3	−118.5
Kushenol S (**246**)	−105.3	−108.5	−99.3	−102.3	−114.3	−117.7	−96.0	−98.8	−109.2	−112.5
Kushenol U (**247**)	−105.2	−100.8	−113.5	−108.8	−123.2	−118.0	−116.2	−111.4	−136.7	−130.9
Kushenol V (**248**)	−127.2	−124.1	−119.1	−116.2	−126.1	−123.1	−56.4	−55.0	−115.9	−113.1
Kushenol W (**249**)	−115.2	−113.7	−105.6	−104.2	−121.5	−119.9	−107.3	−105.9	−120.3	−118.7
Leachianone A (**250**)	−107.2	−101.4	−116.6	−110.4	−131.1	−124.0	−112.3	−106.3	−128.0	−121.1
Leachianone G (**251**)	−106.5	−108.0	−106.8	−108.3	−116.6	−118.3	−101.6	−103.0	−118.4	−120.1
Licoflavanone (**252**)	−110.8	−114.1	−115.6	−119.0	−117.8	−121.3	−110.4	−113.7	−114.5	−117.9
Licoflavone C (**253**)	−107.5	−110.9	−97.1	−100.2	−117.2	−121.0	−104.9	−108.2	−118.0	−121.7
Licoflavonol (**254**)	−110.0	−111.7	−109.2	−110.9	−119.7	−121.7	−98.1	−99.7	−125.9	−127.9
Lonchocarpol A (**255**)	−126.3	−122.4	−116.9	−113.3	−127.3	−123.4	−107.7	−104.4	−134.0	−129.9
Loranthin (**256**)	−135.7	−123.2	−143.8	−130.6	−128.2	−116.4	−111.7	−101.5	−132.5	−120.3
Loxophlebal A (**257**)	−121.0	−111.7	−107.6	−99.3	−120.3	−111.0	−95.7	−88.3	−96.3	−88.9
Lucenin 2 (**258**)	−96.6	−81.8	−132.8	−112.6	−153.2	−129.8	−133.3	−113.0	−143.1	−121.3
Macarangaflavanone A (**259**)	−131.3	−127.2	−134.3	−130.1	−136.6	−132.3	−121.9	−118.2	−137.5	−133.2
Malvidin (**260**)	−104.1	−108.2	−106.4	−110.6	−106.6	−110.7	−92.8	−96.4	−107.8	−112.0
Myricetin (**261**)	−100.0	−105.3	−101.0	−106.4	−103.9	−109.4	−97.0	−102.1	−104.0	−109.5
Natsudaidain (**262**)	−110.5	−106.2	−95.6	−91.9	−118.7	−114.1	−88.0	−84.6	−116.6	−112.1
Nevadensin (**263**)	−95.0	−97.4	−99.8	−102.4	−110.0	−112.8	−98.7	−101.3	−118.4	−121.4
*O*-Methylpongaglabol (**264**)	−93.6	−101.4	−95.6	−103.5	−104.4	−113.1	−90.0	−97.5	−101.2	−109.7
Paratocarpin L (**265**)	−132.0	−127.9	−131.3	−127.2	−128.6	−124.6	−118.4	−114.7	−139.7	−135.3
Persicogenin (**266**)	−98.0	−103.4	−98.6	−104.0	−103.2	−108.9	−97.2	−102.6	−108.3	−114.3
Pilosanol A (**267**)	−123.9	−109.4	−127.5	−112.6	−147.3	−130.0	−101.4	−89.5	−118.5	−104.6
Pilosanol B (**268**)	−120.6	−107.4	−120.9	−107.6	−143.6	−127.9	−100.7	−89.7	−133.9	−119.2
Pilosanol C (**269**)	−116.3	−103.6	−121.9	−108.6	−141.3	−125.8	−106.8	−95.1	−143.1	−127.4
Pinocembrin (**270**)	−80.9	−91.6	−91.6	−103.6	−93.1	−105.3	−79.9	−90.5	−94.1	−106.5
Pongaflavone (**271**)	−98.7	−102.2	−85.2	−88.2	−95.9	−99.3	−84.8	−87.9	−94.0	−97.4
Quercetin (**272**)	−97.5	−104.4	−98.2	−105.2	−106.8	−114.5	−93.6	−100.2	−106.4	−114.0
Quercetin 3-methyl ether (**273**)	−102.0	−107.6	−95.2	−100.4	−108.0	−114.0	−98.6	−104.0	−107.9	−113.8
Remangiflavanone A (**274**)	−103.2	−98.9	−115.7	−110.9	−130.9	−125.4	−108.9	−104.4	−132.9	−127.3
Remangiflavanone B (**275**)	−106.4	−101.8	−115.6	−110.6	−125.8	−120.4	−120.7	−115.5	−128.9	−123.3
Sanggenon G (**276**)	no dock	no dock	−174.6	−141.8	−126.9	−103.0	−124.0	−100.6	−170.1	−138.1
Sigmoidin A (**277**)	−116.3	−111.3	−120.4	−115.2	−125.9	−120.5	−121.9	−116.6	−135.0	−129.2
Sigmoidin B (**278**)	−114.3	−115.9	−121.1	−122.8	−117.3	−119.0	−115.0	−116.6	−120.2	−121.9
Sigmoidin L (**279**)	−114.4	−114.5	−117.4	−117.5	−126.1	−126.3	−115.8	−116.0	−116.5	−116.6
Siraitiflavandiol (**280**)	−105.8	−107.3	−99.4	−100.8	−110.9	−112.4	−109.9	−111.4	−114.0	−115.6
Solophenol D (**281**)	−131.5	−124.5	−127.8	−121.0	−143.8	−136.1	−127.1	−120.2	−139.4	−132.0
Sophoraflavanone G (**282**)	−104.0	−99.5	−117.4	−112.3	−127.9	−122.3	−116.2	−111.2	−127.6	−122.1
Sternbin (**283**)	−92.5	−99.1	−97.8	−104.8	−98.6	−105.6	−91.9	−98.5	−99.1	−106.1
Sudachitin (**284**)	−108.2	−109.3	−98.7	−99.7	−116.4	−117.6	−102.3	−103.4	−116.1	−117.3
Uvarinol (**285**)	−131.4	−113.6	−135.7	−117.3	−164.7	−142.5	−124.4	−107.6	−148.9	−128.7
Vahliabiflavone (**286**)	−70.2	−60.8	−111.4	−96.6	−77.8	−67.5	−95.0	−82.4	−83.5	−72.4
Vitexin (**287**)	−95.2	−90.5	−114.4	−108.8	−138.3	−131.5	−116.5	−110.7	−123.8	−117.7
Wogonin (**288**)	−88.2	−96.4	−87.6	−95.8	−100.6	−110.0	−87.6	−95.8	−100.5	−109.9
**Isoflavonoids**										
2″,3″-Epoxybolusanthol B (**289**)	−101.0	−100.9	−104.3	−104.2	−114.6	−114.5	−110.1	−110.0	−111.1	−111.1
3′,5,7-Trihydroxy-4′-methoxy-5′,6-diprenylisoflavanone (**290**)	−128.1	−121.3	−122.6	−116.0	−128.6	−121.7	−121.9	−115.4	−134.3	−127.1
4″-Hydroxydiphysolone (**292**)	−118.0	−118.0	−107.9	−107.9	−121.9	−121.8	−93.8	−93.7	−121.7	−121.6
5,7-Dihydroxy-2′-methoxy-3′,4′-methylenedioxyisoflavanone (**293**)	−98.4	−102.4	−98.4	−102.4	−102.7	−106.8	−99.0	−103.0	−115.0	−119.6
6*a*-Hydroxyphaseollin (**291**)	−96.4	−99.5	−90.7	−93.6	−90.3	−93.2	−93.7	−96.6	−91.6	−94.5
Amorphaquinone (**294**)	−99.4	−101.8	−89.3	−91.4	−100.6	−103.0	−69.6	−71.3	−106.4	−108.9
Asphodelin A (**295**)	−87.0	−96.8	−86.4	−96.1	−90.3	−100.5	−83.0	−92.3	−91.9	−102.2
Bidwillon A (**296**)	−115.7	−112.1	−128.3	−124.3	−122.4	−118.6	−120.6	−116.9	−125.8	−121.9
Bolucarpan A (**297**)	−93.9	−94.2	−87.2	−87.5	−97.9	−98.2	−79.5	−79.8	−61.3	−61.5
Bolucarpan B (**298**)	−98.9	−99.4	−88.0	−88.4	−98.3	−98.7	−79.3	−79.6	−76.3	−76.7
Bolucarpan D (**299**)	−88.2	−91.2	−85.6	−88.5	−91.7	−94.8	−67.2	−69.4	−71.1	−73.5
Bolusanthol B (**300**)	−97.2	−98.6	−101.4	−102.8	−112.0	−113.5	−108.2	−109.7	−111.7	−113.2
Cajanol (**301**)	−91.9	−97.0	−87.6	−92.4	−97.6	−103.0	−101.5	−107.1	−107.1	−113.0
Chandalone (**302**)	−119.9	−116.6	−124.1	−120.7	−124.5	−121.1	−113.8	−110.6	−130.9	−127.3
Dalversinol A (**303**)	−120.3	−115.1	−111.4	−106.6	−123.2	−117.9	−79.6	−76.2	−118.2	−113.1
Derrisin (**304**)	−81.0	−77.2	−88.8	−84.7	−98.8	−94.2	−66.7	−63.6	−82.4	−78.6
Erybraedin A (**305**)	−109.4	−107.4	−104.4	−102.5	−116.5	−114.4	−74.9	−73.6	−109.3	−107.3
Erybraedin D (**306**)	−98.5	−96.9	−99.9	−98.3	−105.7	−104.0	−73.3	−72.1	−97.4	−95.9
Erypoegin I (**307**)	−108.7	−108.8	−95.7	−95.8	−110.1	−110.2	−95.9	−96.0	−103.0	−103.2
Erysubin F (**308**)	−123.6	−121.6	−135.4	−133.2	−126.5	−124.4	−125.2	−123.2	−128.9	−126.8
Eryvarin V (**309**)	−105.6	−101.2	−101.7	−97.4	−110.4	−105.7	−110.2	−105.6	−125.3	−120.0
Eryvarin W (**310**)	−120.3	−118.3	−115.2	−113.4	−128.6	−126.5	−106.3	−104.6	−118.6	−116.7
Eryzerin C (**311**)	−115.2	−113.0	−120.4	−118.0	−117.2	−114.9	−112.0	−109.8	−131.7	−129.1
Eryzerin D (**312**)	−106.5	−104.6	−116.4	−114.3	−113.9	−111.8	−98.4	−96.7	−120.7	−118.5
Euchretin A (**313**)	−42.4	−38.3	−127.9	−115.7	−144.2	−130.4	−117.4	−106.2	−141.5	−127.9
Gancaonin C (**314**)	−108.6	−110.3	−107.5	−109.2	−109.9	−111.7	−106.4	−108.1	−116.1	−117.9
Genistein (**315**)	−83.6	−93.0	−87.7	−97.5	−93.6	−104.1	−78.7	−87.6	−100.1	−111.3
Glycyrrhisoflavone (**316**)	−113.9	−115.7	−116.0	−117.8	−113.6	−115.4	−108.8	−110.6	−124.3	−126.3
Hispaglabridin A (**317**)	−103.5	−101.6	−115.5	−113.4	−116.1	−114.0	−108.9	−106.9	−116.0	−113.9
Hispaglabridin B (**318**)	−12.3	−12.1	−88.8	−87.4	−103.6	−101.9	−100.8	−99.1	−107.7	−105.9
Hydroxycristacarpone (**319**)	−101.4	−101.6	−95.9	−96.0	−109.3	−109.4	−89.6	−89.7	−103.1	−103.2
Isoneorautenol (**320**)	−86.4	−90.6	−86.8	−91.0	−96.5	−101.2	−55.5	−58.2	−109.7	−115.1
Lachnoisoflavone A (**321**)	−103.1	−104.9	−101.4	−103.2	−110.5	−112.4	−99.6	−101.4	−123.9	−126.1
Licoisoflavanone (**322**)	−95.8	−97.4	−96.5	−98.1	−114.9	−116.7	−104.0	−105.6	−100.2	−101.8
Licoricidin (**323**)	−119.0	−113.9	−126.1	−120.6	−135.0	−129.1	−118.8	−113.6	−127.4	−121.9
Lupinalbin C (**324**)	−96.8	−97.1	−105.3	−105.6	−104.4	−104.7	−89.3	−89.5	−119.0	−119.3
Mucronulatol (**326**)	−93.3	−99.9	−91.9	−98.4	−98.5	−105.5	−94.5	−101.3	−106.1	−113.7
Neomillinol (**325**)	−95.6	−99.9	−98.4	−102.8	−101.7	−106.2	−99.6	−104.0	−110.3	−115.2
Pendulone (**327**)	−94.6	−99.8	−88.5	−93.4	−98.8	−104.2	−85.9	−90.7	−101.5	−107.1
Phyllanone B (**328**)	−128.0	−121.2	−116.4	−110.2	−130.5	−123.5	−102.9	−97.4	−128.9	−122.0
Shinpterocarpin (**329**)	−92.0	−96.4	−95.6	−100.2	−98.6	−103.4	−80.0	−83.9	−100.8	−105.7
**Neoflavonoids**										
Inophyllum A (**330**)	−83.4	−81.1	−89.4	−86.9	−98.2	−95.5	−65.5	−63.6	−95.6	−93.0
Inophyllum C (**331**)	−69.8	−68.0	−90.5	−88.1	−111.3	−108.4	−82.8	−80.7	−92.6	−90.1
Mammea A/BA (**332**)	−111.4	−108.1	−102.7	−99.6	−125.5	−121.8	−114.4	−111.1	−126.0	−122.3
Mammea A/BB (**333**)	−120.0	−116.5	−104.3	−101.3	−116.1	−112.7	−115.5	−112.1	−124.5	−120.9
Mesuol (**334**)	−104.8	−102.9	−104.6	−102.7	−123.7	−121.5	−109.3	−107.4	−130.8	−128.4
**Pterocarpans**										
1-Methoxyphaseollidin (**356**)	−99.1	−100.7	−101.6	−103.2	−119.3	−121.3	−111.5	−113.3	−117.9	−119.8
Aracarpene 1 (**357**)	−87.3	−93.8	−95.1	−102.1	−105.2	−113.0	−82.9	−89.1	−102.9	−110.5
Aracarpene 2 (**358**)	−88.0	−94.5	−96.0	−103.1	−106.6	−114.4	−88.3	−94.8	−103.1	−110.7
Calopocarpin (**360**)	−99.5	−104.1	−104.3	−109.2	−101.6	−106.3	−104.8	−109.7	−110.9	−116.1
Cristacarpin (**359**)	−109.1	−110.9	−101.9	−103.5	−109.6	−111.3	−100.9	−102.5	−108.3	−110.0
Erythbidin D (**362**)	−94.8	−101.7	−95.5	−102.5	−95.5	−102.5	−91.2	−97.9	−104.1	−111.8
Eryzerin E (**363**)	−107.5	−103.0	−121.3	−116.2	−125.3	−120.0	−118.2	−113.3	−120.5	−115.4
Fuscacarpan B (**364**)	−100.7	−100.8	−101.0	−101.2	−105.8	−105.9	−101.5	−101.6	−101.5	−101.6
Fuscacarpan C (**365**)	−98.5	−98.6	−102.0	−102.1	−109.7	−109.9	−97.3	−97.4	−102.5	−102.7
Glycyrol (**366**)	−108.1	−108.6	−110.1	−110.6	−119.8	−120.4	−94.6	−95.1	−129.1	−129.8
Glycyrrhizol A (**367**)	−125.4	−120.4	−127.8	−122.6	−127.4	−122.3	−89.6	−85.9	−123.9	−118.9
Glycyrrhizol B (**368**)	−96.7	−98.6	−99.8	−101.8	−104.4	−106.4	−82.4	−84.1	−93.4	−95.3
Sandwicensin (**369**)	−97.6	−100.7	−103.9	−107.2	−110.9	−114.4	−97.1	−100.2	−112.0	−115.6
Variabilin (**370**)	−89.5	−96.1	−91.6	−98.4	−92.2	−99.0	−96.5	−103.6	−103.6	−111.2
*ent*-Sophoracarpan A (**361**)	−85.4	−91.7	−82.4	−88.5	−89.5	−96.1	−78.0	−83.7	−101.3	−108.7
**Chromones**										
3-(3-Hydroxy-4-methoxybenzylidene)-6,7-dimethoxy-4-chromanone (**371**)	−96.1	−98.8	−100.2	−102.9	−113.2	−116.4	−94.0	−96.6	−106.8	−109.8
4′,5,7-Trihydroxy-6,8-dimethylhomoisoflavanone (**372**)	−91.4	−96.6	−94.3	−99.7	−102.7	−108.6	−74.9	−79.2	−117.4	−124.1
4′,5,7-Trihydroxy-6-methylhomoisoflavanone (**373**)	−87.9	−94.4	−98.8	−106.1	−101.1	−108.6	−83.4	−89.5	−114.3	−122.7
7-*O*-Methylbonducellin (**374**)	−89.2	−96.2	−92.6	−99.8	−99.0	−106.8	−90.1	−97.2	−99.6	−107.4
8-Methylophiopogonanone B (**375**)	−95.4	−99.5	−95.4	−99.5	−103.5	−107.8	−89.3	−93.1	−123.3	−128.5
Bonducellin (**376**)	−86.9	−95.3	−92.9	−101.8	−101.9	−111.7	−85.9	−94.1	−98.8	−108.3
Odoratumone A (**377**)	−98.7	−101.3	−104.2	−106.8	−105.6	−108.3	−92.4	−94.7	−124.9	−128.1
Sappanone A 3′,4′-methylene ether (**378**)	−94.0	−101.4	−97.7	−105.3	−107.7	−116.1	−90.7	−97.9	−104.7	−112.9
Sappanone A 4′-methyl ether (**379**)	−90.9	−97.9	−95.3	−102.6	−104.9	−112.9	−88.9	−95.7	−96.4	−103.7
Sappanone A trimethyl ether (**380**)	−92.4	−96.5	−101.6	−106.1	−110.3	−115.2	−78.7	−82.2	−102.3	−106.8
**Condensed Tannins**										
GB 1 (**381**)	−76.3	−66.6	−123.2	−107.6	−107.4	−93.8	−92.9	−81.1	−91.9	−80.2
Proanthocyanidin A_1_ (**382**)	−40.8	−35.2	−110.1	−95.1	−92.8	−80.2	−97.6	−84.3	−121.2	−104.7
Proanthocyanidin A_2_ (**383**)	−12.3	−10.6	−105.7	−91.3	−122.7	−106.0	−89.7	−77.5	−123.7	−106.9
Procyanidin B_4_ (**384**)	−111.1	−95.9	−145.7	−125.8	−120.6	−104.0	−120.5	−104.0	−99.6	−86.0
Procyanidin B_5_ (**385**)	−77.8	−67.1	−142.5	−123.0	−153.0	−132.1	−74.1	−63.9	−128.4	−110.8
Procyanidin B_6_ (**386**)	−41.1	−35.5	−148.1	−127.8	−131.9	−113.8	−89.6	−77.4	−161.6	−139.5
Teatannin (**387**)	−119.7	−112.9	−113.6	−107.2	−133.0	−125.5	−115.2	−108.7	−145.4	−137.2
**Coumarins**										
4,5′,8′-Trihydroxy-5-methyl-3,7′-bicoumarin (**344**)	−106.7	−108.6	−103.0	−104.9	−107.6	−109.6	−94.2	−95.9	−105.2	−107.0
6-Geranyl-5,7-dihydroxy-8(2-methylbutanoyl)-4-phenylcoumarin (**345**)	−139.0	−128.2	−124.6	−114.8	−141.0	−130.0	−125.9	−116.0	−149.2	−137.6
(–)-Heliettin (**354**)	−71.0	−90.7	−73.4	−93.8	−74.5	−95.1	−67.3	−86.0	−75.4	−96.3
Aesculin (**347**)	−104.4	−107.6	−99.4	−102.4	−111.8	−115.1	−99.0	−102.0	−121.1	−124.7
Alloimperatorin (**346**)	−97.9	−108.9	−92.2	−102.6	−102.0	−113.4	−84.0	−93.5	−101.6	−113.0
Anofinic acid (**348**)	−71.4	−87.2	−71.4	−87.1	−80.1	−97.7	−82.0	−100.1	−79.9	−97.6
Calaustralin (**349**)	−115.4	−112.2	−104.9	−101.9	−121.1	−117.8	−101.9	−99.1	−109.5	−106.5
Calophyllolide (**350**)	−102.1	−98.3	−102.8	−98.9	−111.2	−107.1	−102.5	−98.7	−108.8	−104.8
Dicoumarol (**351**)	−95.1	−98.3	−93.1	−96.3	−102.9	−106.4	−95.2	−98.4	−118.7	−122.8
Dipetalolactone (**352**)	−84.1	−89.3	−80.4	−85.4	−105.9	−112.4	−86.3	−91.7	−104.6	−111.1
Glycycoumarin (**353**)	−107.2	−107.5	−112.6	−112.9	−118.7	−119.1	−106.0	−106.4	−122.7	−123.1
Marmesin (**355**)	−83.0	−95.2	−83.0	−95.2	−86.7	−99.4	−82.4	−94.6	−93.6	−107.4
**Stilbenoids**										
2-(2,4-Dihydroxyphenyl-5-(1-propenyl)benzofurans (**388**)	−94.3	−105.3	−95.4	−106.6	−102.1	−114.1	−95.9	−107.2	−104.4	−116.7
Albanol A (**389**)	no dock	no dock	−142.0	−123.7	no dock	no dock	−100.1	−87.2	−136.8	−119.2
Albanol B (**390**)	no dock	no dock	−106.6	−93.0	−75.9	−66.3	−110.2	−96.2	−135.2	−118.0
Amorfrutin A (**391**)	−114.0	−117.4	−105.8	−109.0	−113.2	−116.6	−97.0	−99.9	−126.9	−130.7
Cajaninstilbene acid (**392**)	−117.7	−121.4	−107.9	−111.3	−115.8	−119.4	−53.3	−55.0	−113.5	−117.1
Calodenin B (**393**)	−159.1	−141.9	−155.9	−139.0	−158.9	−141.6	−143.8	−128.2	−165.3	−147.4
Centrolobofuran (**394**)	−87.2	−98.7	−92.9	−105.1	−94.2	−106.6	−95.3	−107.9	−106.0	−120.0
Cochinchinenene A (**395**)	−112.8	−99.6	−143.0	−126.2	−123.9	−109.3	−112.9	−99.7	−137.4	−121.3
Cochinchinenene B (**396**)	−122.7	−110.2	−142.7	−128.2	−131.4	−118.1	−127.9	−114.9	−136.1	−122.3
Cochinchinenene C (**397**)	−110.4	−100.1	−137.3	−124.5	−137.0	−124.2	−104.5	−94.8	−127.7	−115.8
Cochinchinenene D (**398**)	−119.6	−109.5	−140.6	−128.7	−135.7	−124.2	−122.6	−112.2	−127.6	−116.8
Egonol (**399**)	−114.9	−120.0	−116.0	−121.2	−116.8	−122.0	−107.2	−112.0	−120.4	−125.8
Erypoegin F (**400**)	−117.7	−119.8	−115.7	−117.8	−126.7	−128.9	−115.8	−117.9	−118.2	−120.3
Erythbidin E (**401**)	−89.9	−101.8	−95.4	−108.0	−98.2	−111.2	−92.7	−104.9	−103.3	−117.0
Eryvarin Q (**402**)	−137.9	−133.9	−122.7	−119.1	−135.8	−131.8	−136.1	−132.1	−140.3	−136.2
Gancaonin I (**403**)	−112.2	−114.0	−112.2	−114.0	−117.2	−119.0	−111.0	−112.7	−118.3	−120.2
Glyinflanin H (**404**)	−98.9	−105.3	−97.7	−104.0	−101.5	−108.1	−103.0	−109.6	−110.3	−117.4
Kuwanol A (**405**)	no dock	no dock	−149.0	−129.6	no dock	no dock	−48.6	−42.3	−142.3	−123.8
Licobenzofuran (**406**)	−115.9	−117.8	−128.7	−130.8	−119.7	−121.6	−119.7	−121.6	−123.9	−125.9
Licocoumarone (**407**)	−110.3	−113.6	−110.1	−113.4	−117.1	−120.6	−104.5	−107.6	−116.9	−120.4
Mulberrofuran D (**408**)	−135.0	−127.0	−142.6	−134.1	−153.3	−144.2	−126.6	−119.1	−148.9	−140.1
Mulberrofuran Y (**409**)	−121.9	−118.1	−129.1	−125.1	−143.6	−139.1	−130.2	−126.2	−134.1	−129.9
Pinosylvin (**410**)	−76.0	−91.6	−81.6	−98.3	−84.8	−102.2	−87.5	−105.5	−86.5	−104.2
Schweinfurthin A (**411**)	−84.9	−74.6	−139.0	−122.1	−123.4	−108.3	−133.1	−116.9	−134.6	−118.2
Shanciguol 3-methyl ether (**412**)	−123.9	−116.9	−130.4	−123.0	−133.7	−126.1	−121.4	−114.5	−129.6	−122.3
Stemofuran R (**413**)	−104.6	−109.0	−105.1	−109.5	−104.8	−109.2	−110.5	−115.2	−109.0	−113.6
Stilbostemin S (**414**)	−98.5	−107.2	−100.0	−108.9	−105.7	−115.1	−89.9	−97.8	−107.4	−116.9
Thunberginol F (**415**)	−90.2	−100.4	−94.5	−105.1	−100.3	−111.6	−98.7	−109.7	−111.0	−123.4
(7*E*,7′*R*,8′*R*)-ε-Viniferin (**416**)	−104.3	−97.5	−134.4	−125.7	−89.7	−83.9	−111.9	−104.7	−121.5	−113.6
(7*E*,7′*S*,8′*S*)-ε-Viniferin (**417**)	−106.1	−99.3	−134.6	−125.9	−134.2	−125.5	−116.2	−108.7	−128.6	−120.3
**Phenylpropanoids and Lignans**										
(*E*)-Cinnamaldehyde (**418**)	−59.6	−84.2	−64.5	−91.0	−69.6	−98.2	−62.4	−88.0	−70.9	−100.0
3,4-Dimethylcinnamaldehyde (**419**)	−68.2	−90.2	−73.0	−96.6	−75.0	−99.3	−72.9	−96.5	−82.0	−108.6
Methyleugenol (**422**)	−66.2	−84.6	−77.6	−99.2	−70.8	−90.4	−74.8	−95.5	−85.3	−109.0
*p*-Coumaraldehyde (**420**)	−66.1	−89.9	−71.4	−97.1	−66.4	−90.2	−67.0	−91.1	−74.8	−101.6
*p*-Methoxycinnamaldehyde (**421**)	−69.2	−91.2	−69.1	−91.1	−81.1	−106.9	−72.8	−96.0	−83.5	−110.1
(−)-Asarinin (**423**)	−107.5	−109.2	−111.5	−113.3	−114.5	−116.3	−115.6	−117.4	−111.4	−113.2
Nordihydroguaiaretic acid (**424**)	−107.9	−115.6	−104.4	−111.8	−103.3	−110.6	−98.0	−105.0	−115.6	−123.9
**Xanthones**										
2-Deoxy-4-Hydroxycudratricusxanthone D (**425**)	−101.5	−99.5	−106.9	−104.8	−111.1	−109.0	−99.0	−97.1	−112.2	−110.0
Calozeyloxanthone (**426**)	−95.4	−94.9	−88.9	−88.4	−101.4	−100.8	−100.6	−100.0	−81.5	−81.0
Cycloartobiloxanthone (**427**)	−122.5	−116.3	−101.7	−96.6	−100.2	−95.1	−83.5	−79.3	−100.7	−95.6
Formoxanthone C (**428**)	−102.4	−100.2	−100.5	−98.3	−121.3	−118.8	−101.7	−99.5	−112.7	−110.3
Garciniacowone (**429**)	−108.3	−100.4	−135.4	−125.5	−139.0	−128.9	−109.6	−101.6	−146.0	−135.3
Globulixanthone C (**430**)	−92.0	−96.1	−91.1	−95.1	−95.4	−99.7	−82.4	−86.1	−101.4	−105.9
Globulixanthone D (**431**)	−104.1	−107.0	−106.5	−109.4	−116.1	−119.3	−91.0	−93.5	−114.5	−117.7
Globulixanthone E (**432**)	−11.6	−9.7	−116.0	−97.9	−140.1	−118.2	−69.0	−58.2	−111.3	−93.9
Morellin (**433**)	−104.3	−91.9	−127.6	−112.3	−118.7	−104.5	−78.8	−69.4	−93.4	−82.3
Nigrolineaxanthone N (**434**)	−110.2	−107.6	−120.6	−117.9	−125.2	−122.4	−104.8	−102.4	−133.5	−130.4
Pinselin (**435**)	−86.6	−93.0	−92.7	−99.6	−94.3	−101.3	−80.8	−86.7	−101.7	−109.2
Scortechinone B (**436**)	−20.9	−17.9	−138.5	−118.6	−85.9	−73.5	−120.0	−102.7	−112.6	−96.4
Symphonin (**437**)	−110.8	−103.7	−105.9	−99.2	−120.7	−113.0	−101.8	−95.3	−110.4	−103.4
**Hydrolyzable Tannins**										
1,2,3,4,6-Pentagalloylglucose (**335**)	−74.0	−54.3	−194.4	−142.7	−122.4	−89.8	−98.8	−72.5	−175.1	−128.5
Aceritannin (**438**)	−142.1	−131.6	−142.0	−131.5	−146.2	−135.3	−133.5	−123.6	−138.3	−128.1
Ginnalin B (**439**)	−103.7	−109.4	−104.5	−110.2	−113.9	−120.2	−107.1	−113.1	−119.6	−126.2
Ginnalin C (**440**)	−104.5	−110.3	−107.2	−113.1	−107.1	−113.0	−101.3	−106.9	−117.4	−123.9
Panconoside A (**441**)	−99.9	−82.6	−140.4	−116.0	−166.3	−137.5	−98.5	−81.4	−164.5	−136.0
**Miscellaneous Phenolics**										
1,3,7,9-Tetrahydroxy-4,6-dimethyl-2,8-bis(2-methyl-propanoyl)dibenzofuran (**442**)	−111.5	−108.8	−111.0	−108.3	−109.4	−106.8	−104.8	−102.3	−128.2	−125.0
2′,4′-Dihydroxy-6′-methoxy-3′-methylacetophenone (**443**)	−69.8	−86.3	−70.2	−86.8	−72.3	−89.4	−67.4	−83.4	−77.1	−95.4
3′,4′-Dihydroxyacetophenone (**444**)	−62.5	−84.1	−62.0	−83.5	−72.1	−97.1	−64.4	−86.8	−72.7	−97.9
4′-*O*-Methylhonokiol (**446**)	−103.6	−113.8	−105.3	−115.7	−101.5	−111.5	−102.9	−113.0	−113.7	−124.9
4-Deoxyadhumulone 2″,3″-epoxide (**445**)	−101.5	−102.4	−100.9	−101.7	−115.7	−116.6	−105.4	−106.3	−125.9	−127.0
7-(3,4-Dihydroxy-5-methoxyphenyl)-1-phenyl-4-hepten-3-one (**447**)	−115.0	−120.1	−112.9	−117.9	−113.5	−118.5	−109.6	−114.5	−122.4	−127.8
Agrimol C (**449**)	−108.6	−89.3	−130.9	−107.6	−128.1	−105.3	−101.8	−83.7	−164.9	−135.6
Agrimol F (**450**)	−105.7	−88.2	−133.1	−111.0	−146.5	−122.2	−91.4	−76.2	−152.2	−126.9
Agrimol G (**451**)	−16.9	−13.9	−146.7	−120.6	−146.0	−120.0	−101.2	−83.2	−153.9	−126.5
Arzanol (**452**)	−110.9	−108.0	−122.3	−119.1	−125.7	−122.4	−112.6	−109.7	−133.3	−129.9
Aspidinol C (**448**)	−81.8	−96.8	−78.0	−92.3	−79.0	−93.5	−77.5	−91.7	−87.3	−103.3
Bruguierol C (**453**)	−76.4	−93.0	−74.1	−90.1	−71.2	−86.7	−67.5	−82.2	−73.2	−89.0
Cearoin (**454**)	−82.7	−95.1	−89.1	−102.5	−86.1	−99.1	−84.4	−97.1	−107.1	−123.1
Citrusnin A (**455**)	−90.1	−105.4	−99.2	−116.1	−89.1	−104.2	−100.0	−117.0	−107.2	−125.4
Cochinchinenin B (**456**)	−128.3	−113.1	−153.5	−135.3	−150.1	−132.3	−120.0	−105.8	−151.3	−133.4
Cochinchinenin C (**457**)	−133.8	−118.0	−155.2	−136.8	−167.6	−147.8	−85.7	−75.5	−151.0	−133.1
Drummondin D (**458**)	−134.4	−122.0	−117.8	−106.9	−129.1	−117.2	−99.6	−90.4	−107.1	−97.3
Drummondin E (**459**)	−138.3	−125.4	−128.9	−116.9	−136.5	−123.8	−96.9	−87.8	−128.4	−116.5
Eleutherol (**460**)	−79.3	−91.2	−89.8	−103.3	−89.0	−102.4	−74.5	−85.7	−91.3	−105.0
Ellagicacid (**461**)	−95.0	−101.7	−82.8	−88.7	−109.3	−117.1	−84.2	−90.2	−103.6	−111.0
Epicoccolide A (**462**)	−87.2	−87.0	−87.0	−86.8	−107.4	−107.2	−73.9	−73.7	−99.3	−99.1
Gibbilimbol A (**464**)	−93.0	−108.7	−91.6	−107.1	−95.0	−111.1	−95.1	−111.2	−105.8	−123.7
Gibbilimbol B (**465**)	−88.3	−103.3	−95.2	−111.3	−93.4	−109.2	−92.8	−108.6	−100.8	−117.9
Grifolin (**466**)	−112.5	−117.2	−115.3	−120.2	−118.4	−123.4	−100.7	−104.9	−128.3	−133.7
Hyperbrasilol A (**467**)	−87.1	−75.6	−137.0	−118.9	−121.2	−105.1	−79.6	−69.1	−88.8	−77.0
Hyperbrasilol B (**468**)	−129.4	−113.4	−127.0	−111.3	−139.0	−121.8	−97.3	−85.3	−115.4	−101.1
Hyperbrasilol C (**469**)	−149.3	−130.6	−129.2	−113.1	−147.7	−129.3	−77.3	−67.6	−143.4	−125.5
Isodrummondin D (**470**)	−107.9	−98.0	−125.7	−114.2	−142.8	−129.7	−77.9	−70.7	−117.5	−106.7
Isohyperbrasilol B (**471**)	−97.3	−85.2	−126.6	−110.9	−134.5	−117.8	−99.3	−87.0	−128.4	−112.5
Isouliginosin B (**472**)	−119.3	−108.2	−123.7	−112.1	−118.7	−107.7	−99.4	−90.1	−121.2	−109.9
Italipyrone (**473**)	−115.0	−112.1	−107.7	−105.0	−125.3	−122.2	−107.7	−105.0	−129.2	−126.0
Knerachelin A (**474**)	−104.9	−112.6	−96.8	−104.0	−106.5	−114.3	−103.3	−111.0	−123.9	−133.0
Knerachelin B (**475**)	−93.2	−103.6	−95.1	−105.7	−101.1	−112.5	−95.2	−105.9	−109.5	−121.8
Magnolol (**476**)	−97.3	−108.8	−102.5	−114.6	−97.0	−108.4	−99.1	−110.8	−107.3	−119.9
Myrtucommulone A (**477**)	−13.8	−11.4	−122.2	−100.5	−36.0	−29.6	−72.1	−59.2	no dock	no dock
Myrtucommulone B (**478**)	−68.2	−65.8	−96.1	−92.6	−106.3	−102.5	−77.3	−74.6	−85.1	−82.1
Obovatol (**479**)	−95.5	−104.7	−104.6	−114.7	−101.7	−111.5	−95.4	−104.6	−110.7	−121.3
Oenostacin (**480**)	−96.0	−107.3	−98.4	−110.0	−106.2	−118.7	−103.6	−115.8	−113.1	−126.4
Paeonol (**481**)	−62.1	−81.2	−66.5	−86.9	−72.6	−95.0	−69.1	−90.4	−83.7	−109.5
Perlatolic acid (**482**)	−137.2	−129.3	−127.1	−119.8	−145.4	−136.9	−119.9	−113.0	−145.6	−137.1
Plicatipyrone (**483**)	−106.6	−102.5	−104.3	−100.3	−118.2	−113.7	−100.8	−96.9	−130.9	−125.8
Propterol (**484**)	−89.5	−102.9	−91.0	−104.6	−91.9	−105.7	−89.7	−103.2	−101.7	−117.0
Pulverulentone B (**485**)	−80.0	−92.8	−83.0	−96.3	−82.9	−96.1	−68.2	−79.0	−93.5	−108.4
Quinquangulin (**486**)	−88.3	−96.3	−87.1	−95.0	−91.4	−99.7	−71.6	−78.1	−97.1	−105.9
Rhodomyrtone (**487**)	−94.4	−89.1	−105.1	−99.2	−96.8	−91.3	−84.8	−80.0	−91.4	−86.2
Rosmarinic acid (**488**)	−123.1	−124.4	−114.5	−115.7	−123.6	−124.9	−120.4	−121.6	−125.9	−127.3
Rubanthrone A (**489**)	−100.2	−99.6	−96.2	−95.6	−115.9	−115.2	−94.3	−93.7	−100.1	−99.5
Sampsone A (**490**)	−103.0	−101.9	−96.6	−95.6	−104.4	−103.2	−85.9	−84.9	−87.7	−86.7
Sarothralen B (**491**)	−131.8	−114.5	−137.7	−119.7	−145.5	−126.4	−115.6	−100.5	−129.2	−112.2
Sarothralen C (**492**)	−130.3	−112.0	−126.7	−108.9	−132.4	−113.9	−46.3	−39.8	−112.5	−96.7
Sarothralen D (**493**)	−104.1	−89.5	−142.6	−122.6	−128.7	−110.7	−110.6	−95.1	−135.2	−116.2
Shikonofuran C (**494**)	−118.9	−120.3	−120.8	−122.3	−120.1	−121.5	−116.3	−117.7	−128.2	−129.7
Shikonofuran D (**495**)	−115.6	−118.6	−119.1	−122.1	−122.3	−125.4	−106.0	−108.8	−130.0	−133.3
Shikonofuran E (**496**)	−123.0	−124.7	−122.9	−124.6	−125.9	−127.6	−104.1	−105.6	−132.3	−134.1
Sinapic acid (**497**)	−77.3	−91.5	−90.1	−106.6	−84.8	−100.3	−88.8	−105.1	−86.5	−102.3
Walrycin A (**498**)	−58.6	−75.5	−71.0	−91.4	−73.8	−95.0	−66.7	−85.9	−71.5	−92.1
**Quinones**										
2,6-Dimethoxy-1,4-benzoquinone (**336**)	−60.2	−78.5	−70.2	−91.5	−70.7	−92.1	−67.4	−87.8	−73.1	−95.2
2-Methyl-6-prenyl-1,4-benzoquinone (**337**)	−71.8	−89.7	−84.2	−105.3	−77.4	−96.7	−83.6	−104.5	−86.9	−108.6
Omphalone (**499**)	−73.3	−92.0	−79.3	−99.4	−84.8	−106.4	−79.5	−99.7	−86.6	−108.7
Primin (**500**)	−75.0	−91.0	−83.3	−101.0	−84.7	−102.8	−84.5	−102.5	−91.1	−110.5
1,4-Naphthoquinone (**338**)	−56.7	−75.4	−65.3	−86.8	−67.6	−89.9	−61.1	−81.3	−69.8	−92.8
2-Acetylnaphtho[2,3-*b*]furan-4,9-dione (**339**)	−78.6	−90.9	−90.9	−105.2	−89.5	−103.5	−85.9	−99.3	−95.6	−110.5
Alkannin (**340**)	−94.7	−103.1	−95.5	−104.0	−103.8	−113.0	−84.1	−91.6	−107.0	−116.5
Isobutyrylshikonin (**341**)	−108.3	−109.6	−99.7	−100.9	−116.8	−118.3	−82.1	−83.1	−120.5	−122.0
Lapachol (**501**)	−86.1	−99.3	−87.0	−100.3	−89.4	−103.1	−88.6	−102.2	−101.5	−117.1
Mamegakinone (**502**)	−86.8	−86.6	−92.1	−91.8	−103.5	−103.2	−82.5	−82.3	−93.5	−93.3
Menadione (**503**)	−61.8	−79.9	−71.4	−92.3	−71.9	−92.9	−64.0	−82.7	−73.0	−94.4
Rhinacanthin C (**504**)	−131.5	−127.2	−124.0	−119.9	−130.8	−126.5	−109.5	−105.9	−131.8	−127.5
Rhinacanthin D (**505**)	−126.1	−122.2	−122.4	−118.6	−127.4	−123.5	−104.4	−101.2	−122.5	−118.7
Rhinacanthin G (**506**)	−130.7	−124.8	−119.6	−114.2	−130.9	−125.1	−109.1	−104.2	−130.4	−124.6
Rhinacanthin H (**507**)	−129.7	−123.9	−128.1	−122.3	−127.5	−121.8	−108.5	−103.7	−128.1	−122.4
Rhinacanthin I (**508**)	−130.7	−124.8	−123.1	−117.6	−133.8	−127.8	−118.0	−112.7	−138.1	−131.9
Rhinacanthin J (**509**)	−127.4	−121.9	−120.2	−115.0	−128.5	−122.9	−107.8	−103.2	−135.3	−129.4
Rhinacanthin K (**510**)	−134.8	−127.0	−117.4	−110.6	−129.1	−121.6	−96.9	−91.3	−142.2	−134.0
Rhinacanthin L (**511**)	−132.7	−123.5	−125.6	−117.0	−129.4	−120.5	−106.6	−99.2	−115.2	−107.2
Rhinacanthin M (**512**)	−118.0	−118.8	−104.0	−104.7	−113.1	−113.8	−100.7	−101.4	−128.3	−129.1
Shikonin acetate (**513**)	−103.6	−107.7	−104.0	−108.1	−112.4	−116.9	−81.5	−84.8	−118.5	−123.2
β,β-Dimethylacrylshikonin (**514**)	−121.4	−121.6	−113.3	−113.4	−125.9	−126.1	−91.0	−91.1	−123.3	−123.4
β-Hydroxyisovaleryshikonin (**515**)	−119.2	−117.4	−113.2	−111.6	−128.3	−126.5	−95.9	−94.5	−124.2	−122.4
1-Hydroxy-3-hydroxymethylanthraquinone (**516**)	−79.2	−89.9	−81.6	−92.6	−89.9	−102.0	−74.5	−84.5	−93.7	−106.3
Aloeemodin (**518**)	−85.2	−94.8	−86.5	−96.2	−95.6	−106.3	−75.5	−83.9	−96.6	−107.4
Islandicin (**519**)	−83.7	−93.1	−85.9	−95.5	−93.2	−103.6	−73.1	−81.3	−92.9	−103.3
Newbouldiaquinone (**521**)	−108.2	−106.1	−97.6	−95.7	−110.9	−108.7	−84.1	−82.5	−74.5	−73.0
Newbouldiaquinone A (**520**)	−104.8	−101.4	−113.2	−109.5	−120.0	−116.1	−82.8	−80.1	−140.9	−136.3
Rhein (**522**)	−86.4	−94.5	−89.6	−98.0	−98.6	−107.8	−77.4	−84.7	−100.0	−109.4
15,16-Dihydrotanshinone I (**517**)	−79.0	−87.0	−84.7	−93.3	−97.5	−107.3	−71.4	−78.6	−93.1	−102.6
**Acetylene, Glucoside, and Other Miscellaneous Phytochemicals**										
1,7-Diphenyl-4-(2-phenylethyl)-1-heptene-3,5-dione (**530**)	−126.7	−125.5	−113.3	−112.3	−129.5	−128.2	−117.8	−116.7	−139.8	−138.5
1,7-Diphenyl-5-hepten-3-one (**531**)	−102.0	−114.3	−94.3	−105.6	−97.9	−109.6	−96.6	−108.2	−108.7	−121.8
3*'*-Demothexycyclocurcumin (**532**)	−109.1	−112.6	−109.9	−113.4	−117.9	−121.7	−95.8	−98.9	−117.4	−121.2
5,7-Dihydroxyphthalide (**533**)	−62.8	−82.1	−69.7	−91.1	−72.8	−95.2	−65.9	−86.2	−71.7	−93.8
6-Methyl-4,5-dithia-2-octene (**534**)	−58.5	−77.1	−65.8	−86.7	−60.1	−79.1	−65.1	−85.7	−67.2	−88.6
7-Epiclusianone (**535**)	−92.4	−83.5	−116.5	−105.4	−113.6	−102.8	−107.3	−97.0	−88.4	−79.9
Allamandin (**536**)	−94.5	−100.6	−95.4	−101.5	−97.3	−103.5	−86.9	−92.5	−91.7	−97.6
Allicin (**537**)	−56.7	−74.8	−63.3	−83.4	−57.0	−75.1	−59.1	−77.8	−66.2	−87.3
Amadannulen (**538**)	−107.7	−107.3	−111.7	−111.2	−123.0	−122.5	−105.4	−105.0	−113.7	−113.2
Anemonin (**539**)	−71.7	−89.3	−76.0	−94.7	−70.8	−88.3	−64.7	−80.6	−65.9	−82.1
Antibiotic CZ 34 (**540**)	−83.7	−99.7	−75.6	−90.0	−77.9	−92.8	−85.9	−102.2	−84.8	−101.0
Argutone (**541**)	−70.1	−88.3	−74.7	−94.1	−76.8	−96.7	−76.6	−96.5	−79.7	−100.4
Bakuchiol (**542**)	−94.0	−106.3	−89.6	−101.4	−100.5	−113.7	−96.7	−109.4	−104.0	−117.7
Brasiliensophyllic acid A (**543**)	−110.8	−96.6	−124.8	−108.8	−127.0	−110.8	−94.8	−82.7	−122.3	−106.6
Brasiliensophyllic acid C (**544**)	−109.1	−94.4	−140.6	−121.6	−119.0	−102.9	−101.5	−87.8	−95.4	−82.5
Centrolobin (**545**)	−102.6	−108.8	−103.0	−109.2	−110.6	−117.2	−106.3	−112.7	−116.5	−123.5
Chamone I (**546**)	−107.6	−93.3	−125.2	−108.5	−106.2	−92.1	−106.2	−92.1	−103.3	−89.5
Chamone II (**547**)	−68.0	−59.0	−130.2	−113.0	−109.2	−94.8	−80.5	−69.9	−97.2	−84.3
Champanone A (**548**)	−87.4	−98.9	−93.3	−105.6	−95.2	−107.8	−97.3	−110.1	−103.7	−117.4
Dhelwangin (**549**)	−84.1	−99.5	−82.9	−98.1	−82.9	−98.1	−85.1	−100.7	−95.1	−112.6
Garcinoic acid (**550**)	−130.3	−124.5	−128.9	−123.1	−141.3	−134.9	−107.3	−102.5	−147.6	−141.0
Ginkgolide A (**551**)	−94.7	−91.8	−101.4	−98.3	−94.5	−91.5	−73.9	−71.6	−73.9	−71.6
Guttiferone E (**552**)	−119.4	−101.6	−139.9	−119.0	−117.5	−100.0	−109.1	−92.8	−97.4	−82.9
Helipyrone B (**553**)	−97.6	−104.1	−81.6	−87.1	−97.7	−104.2	−92.2	−98.3	−110.0	−117.3
Helipyrone C (**554**)	−90.2	−97.8	−78.8	−85.4	−92.2	−99.9	−84.7	−91.8	−105.0	−113.7
Ialibinone A (**555**)	−86.4	−88.6	−90.7	−93.0	−96.1	−98.6	−85.7	−87.9	−80.7	−82.8
Ialibinone B (**556**)	−82.0	−84.1	−85.4	−87.6	−96.0	−98.5	−81.4	−83.5	−78.5	−80.5
Ialibinone C (**557**)	−85.8	−86.8	−94.3	−95.4	−100.6	−101.8	−89.2	−90.3	−86.9	−88.0
Ialibinone D (**558**)	−87.2	−88.3	−94.0	−95.2	−100.4	−101.6	−84.3	−85.3	−82.1	−83.1
Isobrasiliensophyllic acid A (**559**)	−109.8	−95.7	−130.4	−113.7	−123.7	−107.9	−87.2	−76.1	−102.2	−89.1
Moskachan C (**560**)	−82.1	−97.5	−88.7	−105.3	−87.5	−103.9	−89.0	−105.6	−98.3	−116.6
Nimbolide (**561**)	−94.3	−87.5	−111.7	−103.5	−126.4	−117.2	−95.0	−88.0	−82.3	−76.3
Pectinolide H (**562**)	−98.3	−109.5	−89.0	−99.2	−98.5	−109.9	−102.4	−114.1	−109.9	−122.5
Propolone A (**563**)	−61.3	−55.4	−111.8	−101.1	−94.9	−85.8	−104.3	−94.3	−91.0	−82.3
Sellovicine B (**564**)	−84.5	−98.5	−86.5	−100.9	−85.2	−99.3	−86.9	−101.4	−99.8	−116.4
Simonin A (**565**)	−128.9	−124.1	−106.9	−103.0	−116.1	−111.8	−102.6	−98.8	−105.0	−101.1
Tenulin (**566**)	−87.5	−93.3	−82.7	−88.2	−85.7	−91.4	−74.8	−79.7	−76.7	−81.8
Atractylodin (**522**)	−78.7	−99.9	−83.4	−105.7	−80.7	−102.3	−83.6	−106.0	−87.2	−110.6
Atractylodinol (**523**)	−87.6	−108.1	−91.0	−112.2	−85.5	−105.4	−88.0	−108.5	−94.9	−117.0
Capillene (**342**)	−67.4	−90.4	−74.2	−99.4	−69.6	−93.3	−72.6	−97.3	−79.5	−106.6
Peniophorin A (**524**)	−99.3	−108.8	−104.0	−114.0	−98.8	−108.3	−103.7	−113.7	−117.1	−128.4
Peniophorin B (**525**)	−80.7	−97.2	−88.8	−107.0	−87.4	−105.4	−83.9	−101.1	−95.6	−115.3
Thiarubrin A (**526**)	−76.3	−89.8	−87.1	−102.5	−80.3	−94.5	−80.2	−94.4	−87.1	−102.4
Arbutin (**527**)	−90.2	−100.0	−92.1	−102.1	−95.5	−105.9	−88.1	−97.7	−98.7	−109.5
Aucubin (**528**)	−108.5	−111.1	−93.7	−96.0	−105.7	−108.2	−92.5	−94.7	−110.9	−113.6
Diospyrodin (**529**)	−108.2	−114.4	−91.4	−96.7	−94.4	−99.8	−89.7	−94.8	−116.6	−123.3
**Synthetic Inhibitors**										
(4-chlorophenyl)-[1-(4-chlorophenyl)-3-hydroxy-5-methyl-1*H*-pyrazol-4-yl]-methanone [99] (**579**)	−103.7	−106.1								
3-(4-iodophenyl)-2-[4-(3,4-dichlorophenyl)-thiazol-2-ylamino]-propionic acid [99] (**580**)	−112.3	−100.5								
3-phenyl-2-[5-(3-chlorobenzylidene)-2-thioxo-4-thiazolidinone]-propionic acid [100] (**581**)	−120.5	−117.2								
4-[3-amino-4-(4-hydroxyphenyl)-1*H*-pyrazol-5-yl]benzene-1,3-diol [53] (**582**)			−92.3	−101.1						
4,4′-{3-[(4-hydroxyphenyl)amino]-1*H*-pyrazole-4,5-diyl}diphenol [53] (**583**)			−119.2	−120.5						
4-(3′-Amino-[1,1′-biphenyl]-3-yl)-1*H*-pyrazol-5-amine [32] (**584**)			−92.1	−105.1						
4-(5-Amino-4-(3'-amino-[1,1′-biphenyl]-3-yl)-1*H*-pyrazol-3-yl)-phenol [32] (**585**)			−124.2	−127.6						
Chloroquine					−101.2	−106.4	−93.5	−98.3	−101.8	−107.0
Doxorubicin					−119.6	−105.4	−103.8	−91.5	−144.2	−127.0
2-((2-(1*H*-pyrazolo[3,4-*c*]pyridin-3-yl)-6-(trifluoromethyl)pyrimidin-4-yl)-amino)-ethanol [56] (**586**)					−113.0	−118.2	−105.2	−110.1	−123.4	−129.2
2-cyclobutylmethoxy-5′-fluoroadenosine [33] (**587**)					−119.4	−121.4	−115.8	−117.8	−132.3	−134.5
2-phenoxy-5′-deoxyadenosine [34] (**588**)					−118.4	−121.6	−112.9	−115.9	−120.9	−124.2
2-amino-6-bromo-7-(trifluoromethyl)-[1,8]-naphthyridine-3-carboxamide [54] (**589**)					−99.6	−103.1	−78.3	−81.0	−90.1	−93.3
2-amino-6-bromo-7-(2-benzyloxy-2-methylethyl)-[1,8]-naphthyridine-3-carboxamide [54] (**590**)					−122.1	−117.7	−91.1	−87.8	−117.4	−113.1
4-amino-2-(2-fluoroethoxy)-*N*-(2-hydroxyethyl)-purido[2,3-*d*]pyrimidin-5(8*H*)-one [103] (**591**)					−98.3	−109.6	−81.3	−90.6	−93.5	−104.3
4-amino-2-(2-ethylcyclohexoxy)-*N*-(2-hydroxycyclopentyl)-purido[2,3-d]-pyrimidin-5(8*H*)-one [103] (**592**)					−120.6	−122.1	−97.0	−98.2	−115.7	−117.1

^a^ Compounds shown in red font violate Lipinski’s rule-of-five [62].
